# Jewelled spider flies of North America: a revision and phylogeny of *Eulonchus* Gerstaecker (Diptera, Acroceridae)

**DOI:** 10.3897/zookeys.619.8249

**Published:** 2016-09-27

**Authors:** Christopher J. Borkent, Jessica P. Gillung, Shaun L. Winterton

**Affiliations:** 1 California State Collection of Arthropods, California Department of Food and Agriculture, 3294 Meadowview Road, Sacramento, CA 95832, USA

**Keywords:** Antrodiaetidae, Euctenizidae, spider parasitoid, phylogeny, small-headed fly, tarantula, biodiversity, cybertaxonomy, Lucid

## Abstract

The spider fly genus *Eulonchus* Gerstaecker is found throughout the Nearctic Region. Six species are recognized and intraspecific morphological variation is documented in several species. A phylogeny of *Eulonchus* based on DNA sequence data of three molecular markers (COI, CAD, and 16S) is presented and relationships of species are discussed in the light of biogeography and host usage. All six species of *Eulonchus* are redescribed using natural language descriptions exported from a character matrix, and a key to species is presented. Lectotypes are designated for *Eulonchus sapphirinus* Osten Sacken, *Eulonchus smaragdinus* Gerstaecker, and *Eulonchus tristis* Loew.

## Introduction

Acroceridae are a small group of flies commonly known as spider flies or small-headed flies. The family comprises a morphologically heterogeneous assemblage of taxa, with approximately 550 species in 55 genera (Schlinger et al. 2013) distributed in all major biogeographic regions. Acroceridae is divided into three subfamilies: Philopotinae, Panopinae and the polyphyletic Acrocerinae (Schiner 1868; Cole 1919; Schlinger 1981; Winterton et al. 2007; Gillung and Nihei 2016). Adults are remarkably diverse in morphology, sometimes with elongated mouthparts for nectar feeding or with a strongly arched thorax, while the larvae are specialised endoparasitoids of spiders. First instar acrocerid larvae are free-living planidia that either actively seek out a spider host or sit and wait for the host to pass by (Cole 1919; Schlinger 1987; Nielsen et al. 1999).

Panopinae (tarantula flies) represent some of the smallest and largest spider flies, with a body size ranging from 4.0 mm (*Corononcodes* Speiser) to 19.0 mm (*Exetasis* Walker). Winterton (2012) recently diagnosed the subfamily, which is characterised by the antennal flagellum cylindrical or paddle shaped (never stylate), lacking an arista, postpronotal lobes widely separated, wing venation relatively conserved with cells m_3_, d, bm and basal r_4+5_ normally present, tibial spines present (absent only in *Apsona* Westwood), and parasitism on Mygalomorphae. Only *Corononcodes* deviates significantly from this diagnosis, due to reduction in wing venation associated with the very small body size of most species. All species of Panopinae with known life histories are exclusive parasitoids of mygalomorph spiders and have been recorded from 5 families, including Antrodiaetidae, Ctenizidae, Dipluridae, Euctenizidae, Migidae, and Theraphosidae (Schlinger 1987; Winterton et al. 2007; Barneche et al. 2013). Panopinae contains ~135 species divided into 23 genera. The fauna of the Palaearctic, Oriental, and Afrotropical Regions contains seven genera, including *Corononcodes*, *Astomella* Latreille, *Astomelloides* Schlinger, *Physegasterella* Brunetti, *Psilodera* Gray, *Rhysogaster* Aldrich, and *Stenopialea* Speiser. The panopine fauna of the Australasian Region was recently revised by Winterton (2012) to contain only four genera: *Apsona*, *Leucopsina* Westwood, *Mesophysa* Macquart and *Panops* Lamarck. The Neotropical Region is rich in genera, containing *Apelleia* Bellardi, *Archipialea* Schlinger, *Arrhynchus* Philippi, *Camposella* Cole, *Coquena* Schlinger, *Eulonchus* Gerstaecker, *Exetasis*, *Lasia* Wiedemann, *Lasioides* Collado, *Ocnaea* Erichson, *Pialea* Erichson, and *Pteropexus* Macquart, although the group is in need of revision, with several generic synonyms likely (Schlinger et al. 2013). *Pterodontia* Lamarck was considered by some authors as part of Panopinae, but was conclusively shown to be a derived member of Acrocerinae by Winterton et al. (2007) based on molecular data. The Nearctic Region is faunistically depauperate in panopine taxa, with only three genera: *Eulonchus*, *Lasia*, and *Ocnaea*.

Species of *Eulonchus*, *Lasia* and the Australasian genera *Apsona* and *Panops* are commonly called ‘jewelled spider flies’, while species of the New World genus *Ocnaea* are known as ‘hairy tarantula flies’. *Lasia* and *Ocnaea* are relatively diverse in the Neotropical Region but are represented in the Nearctic Region only by a few extralimital species. In contrast, *Eulonchus* is endemic to the Nearctic Region. Six species of *Eulonchus* are present, distributed from Canada to Mexico. *Eulonchus marialiciae* Brimley is known only from the eastern USA, while all other species are recorded from the western part of the continent, from Baja California, Mexico, north to British Columbia, Canada, and east to Utah and Arizona. The greatest number of species is known from California, although there are records from Arizona, Idaho, Montana, Nevada, Oregon, Utah, and Washington. *Eulonchus marginatus* Osten Sacken, *Eulonchus sapphirinus* Osten Sacken, and *Eulonchus tristis* Loew are known from Northern California, the latter two species extending northwards to Oregon, Idaho, Washington and British Columbia. *Eulonchus halli* Schlinger and *Eulonchus smaragdinus* Gerstaecker are found in California south to Baja California, Mexico. Brunetti (1926) recorded a specimen of *Eulonchus* from Uruguay, but this record was subsequently dismissed as a likely misidentification of a species of *Lasia* (Schlinger 1960). Some species of *Eulonchus* were previously revised by Cole (1919) and Schlinger (1960), and a total of six species are described (as well as two subspecies of *Eulonchus smaragdinus*). Considerable sexual dimorphism and variation in pile density and cuticle colouration is evident in several species. Schlinger indicated at several times (1960, 1966, 1969) that there were a large number (>15) of undescribed species, and that the genus should be divided into species groups, but he only defined a single species group comprising *Eulonchus smaragdinus* and *Eulonchus halli*.

*Eulonchus* attack spiders in the families Euctenizidae and Antrodiaetidae (Coyle 1971; Vincent 1986; Schlinger 1987), although hosts are only known conclusively for *Eulonchus marialiciae*, *Eulonchus smaragdinus*, and *Eulonchus tristis*. Little is known of the biology of the parasitoid larvae, though one of us (C. Borkent) has observed that the newly hatched first instars are active in searching for a host. The larvae crawl in the manner of geometrid larvae, inching along and occasionally rearing up and wiggling their body side to side, presumably hoping to attach to a passing spider leg.

Several studies also demonstrate the importance of species of *Eulonchus* as pollinators of several plant families in montane areas of the Nearctic Region, including Boraginaceae, Geraniaceae, Themidaceae, and Iridaceae (Borkent and Schlinger 2008a, b). They have also been previously recorded visiting flowers of species in the Phrymaceae, Polemoniaceae, Primulaceae, and Ericaceae. Schlinger (1960) suggested that *Eulonchus halli* was a mutualist pollinator of *Cryptantha intermedia* Greene (Boraginaceae) as the flowering time of this plant appeared to coincide closely with the flight period of *Eulonchus halli*. Based on the behaviour observed by Borkent and Schlinger (2008a, b) this seems unlikely.

We revise the jewelled spider flies of North America (*Eulonchus*) and redescribe all valid species using cybertaxonomic methods of natural language description. A molecular phylogeny of the genus is presented along with a discussion of its evolution in a biogeographical context.

## Materials and methods

### Generic revision

Terminology for general morphology follows Cumming and Wood (2009), with wing venation as in Gillung and Winterton (2011) and Winterton (2012). Modifications of wing terminology as proposed by Saigusa (2006) were used, in which the dipteran vein A_1_ (as used in Cumming and Wood 2009) was homologized with CuP of Mecoptera. Consequently, the following wing venation terms are used here: CuA_1_ (of Cumming and Wood) = M_4_, CuA_2_ = CuA, and anal vein (A_1_) = CuP.

Descriptions were constructed with Lucid Builder 3.5, using a matrix database of character states, which were then exported using the natural language function into a text document (for further editing) and accompanying XML. Specimen images were taken using a digital camera at different focal points and combined into a montage image using Helicon Focus software. Distribution maps were generated using ArcGIS 10.1 software (ESRI 2012). When GPS data were not available on the label, latitude and longitude were obtained by consulting online gazetteers and Google Earth™. Any plants listed as visited by *Eulonchus*, but without citation, are new records based on label data and collection records from this study.

### Material examined

The specimens examined during the course of this study (Table 3) were principally part of the collection amassed by Dr. Evert Schlinger over more than 60 years. This collection now resides at the California Academy of Sciences
(CAS). While most belonged to the Schlinger collection, a small minority of these specimens were loaned from other collections, but do not have that information clearly associated with them (either physically or in the incomplete specimen database). Therefore we cannot determine whether the specimen was loaned versus gifted; here we use a conservative approach and list only what is noted in the database or associated labels in the collection.

Annotations of collection label data are included where appropriate in brackets. The following collection codes are cited in the Material Examined: CAS – California Academy of Sciences, San Francisco, USA; EMEC – Essig Museum of Entomology, University of California Berkeley, California, USA; MCZ – Museum of Comparative Zoology, Harvard University, Cambridge, Massachusetts, USA; OSUC – Oregon State University, Oregon, Corvallis, USA; DEI – Senckenberg Deutsches Entomologisches Institut, Müncheberg, Germany; TCAC – Insect Collection of the Tulare County Agricultural Commissioner/Sealer, Tulare, California, USA; TAIU – Texas A&M University, Kingsville, Texas, USA; UCDC – Bohart Museum of Entomology, University of California Davis, California, USA; USNM – National Museum of Natural History, Washington D.C., USA; ZMB – Museum für Naturkunde, Berlin, Germany. Museum acronyms follow Evenhuis (2015).

### Phylogenetic analysis

Five species of *Eulonchus* and two outgroup species (*Apsona muscaria* Westwood and *Lasia corvina* Erichson (given as *Lasia carbonanicus* in Winterton et al. 2007, a misspelling of *Lasia carbonarius* Philippi = *Lasia corvina* (Edwards 1930))) were included in the analyses. Multiple DNA loci, as used previously in the phylogenetic study of spider fly evolution by Winterton et al. (2007), were included here. The dataset comprises sequences of two mitochondrial genes, 16S rDNA and cytochrome oxidase I (COI), as well as a single nuclear gene, the carbamoyl phosphate synthetase (CPSase) active site region of carbamoyl-phosphate synthetase-aspartate transcarbamoylase-dihydroorotase (CAD). Sequences from *Apsona muscaria*, *Lasia corvina*, *Eulonchus marialiciae*, *Eulonchus sapphirinus*, and *Eulonchus smaragdinus* were downloaded from Genbank, and new sequences for *Eulonchus marginatus*, *Eulonchus sapphirinus*, and *Eulonchus tristis* were added. DNA sequencing for these additional taxa was carried out following the protocol outlined by Winterton et al. (2007). Genbank accession and specimen voucher numbers are presented in Table 1. Sequences were aligned manually, with CAD and COI aligned with reference to translated amino acid sequences using Mesquite 2.75 (Maddison and Maddison 2014). Parsimony analyses were conducted using PAUP* 4.0b10 (Swofford 2003) using a branch and bound search protocol. Bootstrap support values were calculated from 200 heuristic search (TBR) pseudoreplicates of re-sampled data sets, each with 30 random additions (constant characters excluded).

**Table 1.** Specimens used for sequencing in this study, with associated Genbank accession numbers. EIS = E.I. Schlinger Collection specimen numbers; NCSU = North Carolina State University collection specimen number.

	Collection #	Genbank Accession No.			Collection Data
		16S	COI	CAD	
*Apsona muscaria Westwood*	*EIS 009*956	AY140851	—	AF539866	New Zealand: Otira Valley; January, 1999; LJ Boutin
*Lasia corvina Erichson*	*EIS 0*10914	AY140856	DQ631969	AF539865	Chile: Nuble Prov.: Las Trancas; January, 2000; DK Yeates
*Eulonchus marginatus Osten Sacken*	*EIS 02*0189	KU215393	KU215390	—	USA: California: Mendocino County: Angelo Coast Range Reserve; May, 2005; CJ Borkent
*Eulonchus marialiciae Brimley*	*EIS 009955*	AY140853	DQ631979	AF539887	USA: North Carolina: Great Smoky Mountains N.P.; June, 1999; D Dafoe
*Eulonchus sapphirinus Osten Sacken*	*NCSU 99*-07-21-48	AY140852	—	AF539877	USA: Washington: South Fork: Wenatachee N.F; July, 2000; KC Holston
*Eulonchus sapphirinus Osten Sacken*	*CSCA15L30*3-16V350	—	KU215391	—	USA: California: Calaveras County: Calaveras Big Trees State Park; June, 2010; AR Cline
*Eulonchus smaragdinus Gerstaecker*	*NCSU 99-07*-09-76	AY140854	—	AF539867	USA: California: San Diego County: San Diego; June 2000; SL Winterton
*Eulonchus smaragdinus Gerstaecker*	*CSCA15L304*-16V351	—	KU215392	—	USA: California: Alameda County: Pleasanton Ridge Regional Park: June, 2012; JM Ayala
*Eulonchus tristis Loew*	*CSCA15L302-16*V349	KU215394	—	—	USA: Oregon: Lane County: Trail Creek; July 2000; JK Moulton

## Taxonomy

### Eulonchus

Taxon classificationAnimaliaDipteraAcroceridae

Gerstaecker, 1856

Figs 1 , 2 , 3 , 4 , 5 , 6 , 7 , 8 , 9 , 10 , 11 , 12 , 13 , 14 , 15 , 16 , 17 , 18 , 19

Eulonchus Gerstaecker, 1856: 359

#### References.

Westwood 1876: 517 (generic checklist); Williston 1888: 40 (key), 1896: 71 (key), 1908: 185 (key); Bigot 1890: 315 (key); Coquillett 1910: 541 (type checklist); Cole 1919: 31 (description, key); Sabrosky 1948 (revision, key); Paramonov 1955: 20 (comments); Schlinger 1960 (revision), 1966: 112 (distribution), 1981: 583 (key), 1987 (host records); Coyle and Icenogle 1994 (host records); Poole 1996: 36 (checklist).

#### Type species.

*Eulonchus smaragdinus* Gerstaecker, 1856 by monotypy.

#### Common name.

North American jewelled spider flies.

#### Diagnosis.

*Eulonchus* can be immediately identified from other Panopinae in the New World by the presence of elongate mouthparts, metallic colouration, and pilose eyes. The only other genus in the New World with metallic colouration is *Lasia*, which is distinguished from *Eulonchus* in the eyes being separated below the antennae, and in lacking the palpus and alula. *Eulonchus* is remarkably similar to the endemic New Zealand genus *Apsona* and is distinguished from it by the presence of tibial spines (lacking in *Apsona*), the wing medial veins reaching the wing margin (attenuated in *Apsona*) and the antennae placed in the middle of the frons (higher in *Apsona*).

#### Description.

Body length: 7.2–12.8 mm; wing length: 5.2–12.6 mm. Body colouration metallic green, blue or purple, rarely non-metallic (likely due to poor preservation or collection methods). *Head* hemispherical, width slightly smaller than thorax width; ocellar tubercle slightly to strongly raised, shape variable and either rounded, bifid or trifid, two or three ocelli present; postocular ridge and occiput rounded; eye densely pilose, setae relatively long (2x pedicel length), posterior margin of eye rounded (*i.e.* not emarginate); antenna located on middle of frons; eye contiguous above and below antennal base; palpus present; proboscis length greater than head length; antennal flagellum elongate, cylindrical to laterally compressed, slightly to strongly tapered apically; in western species male flagellum cylindrical, broader distally, female flagellum more tapered distally; flagellum apex with or without terminal setae; scapes separate (*i.e.* not fused medially). *Thorax* with postpronotal lobes not enlarged or contiguous medially; antenotum narrow; subscutellum barely visible beneath scutellum; legs not greatly elongated; tibial spines present apically; pulvilli present; wing hyaline, markings absent; costal vein circumambient around wing, costal margin straight along entire length, rounded apically; humeral crossvein present; vein R_1_ not inflated distally; R_4_ and R_5_ present as forked petiolate veins; radial veins curved towards wing anterior margin; crossvein 2r-m present between M_1_ and R_4+5_, bisecting cell r_4+5_; R_4_ with or without spur vein; veins M_1_, M_2_ and M_3_ present and reaching wing margin; discal cell closed completely; M_4_ joining M_3_, cell m_3_ petiolate; CuA fused to CuP before wing margin, cell CuA petiolate; wing microtrichia absent; anal lobe well developed; alula weakly developed; abdominal tergites smooth, rounded. *Abdomen* shape conical to turbinate. *Male genitalia* (Figs 17–19). Cerci smooth, densely pilose; dorsal surface of epandrium covered with dense, long pile, posterior margin of epandrium usually concave (straight in *Eulonchus marialiciae* and *Eulonchus smaragdinus*); gonocoxite variable in shape and size, with distal apex thinned; aedeagus broad at apex (slightly thinned in *Eulonchus halli*).

**Figure 1.**
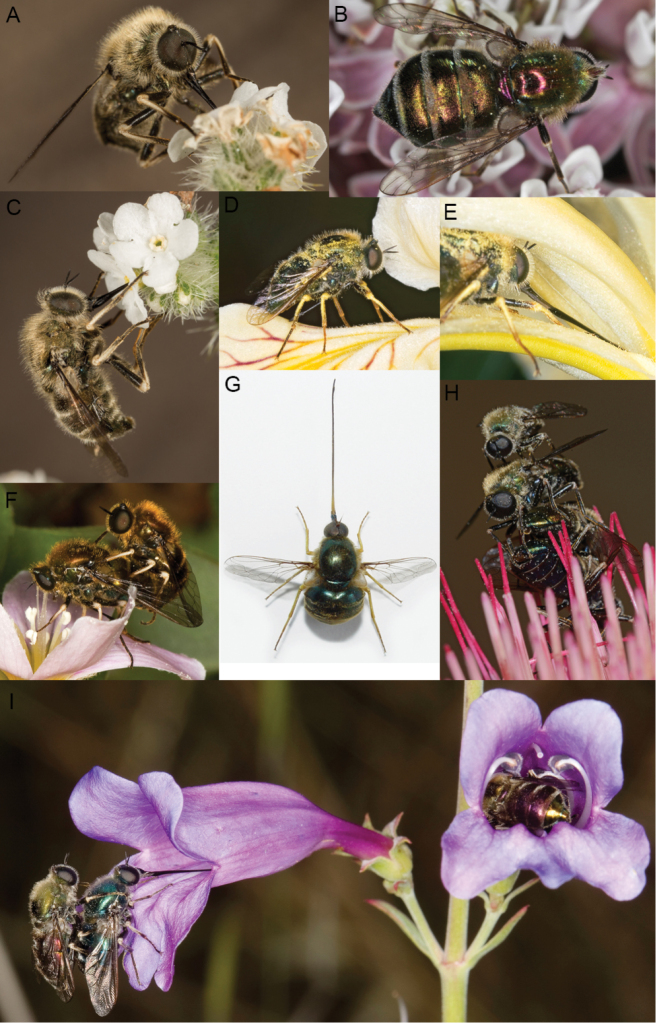
*Eulonchus* spp. on various flowers. **A**, **C** Male *Eulonchus tristis* Loew on *Cryptantha intermedia* **B** Male *Eulonchus marginatus* Osten Sacken trapped on flower of *Asclepias* sp **D, E** Male *Eulonchus tristis* approaching and feeding from *Iris bracteata* **F** Mated pair of *Eulonchus tristis* on *Oxalis oregana* **G** *Eulonchus smaragdinus* Gerstaecker **H** Three male *Eulonchus marginatus* attempting to mount a single female feeding on thistle **I** Mated pairs of *Eulonchus tristis* on *Penstemon heterophyllus*, with one pair resting at length inside the flower. Photo credits: A. Schusteff (**D, E, F, I**); A. Abela (**A, C**); G. McDonald (**B, H**); R. Waayers (**G**).

**Figure 2.**
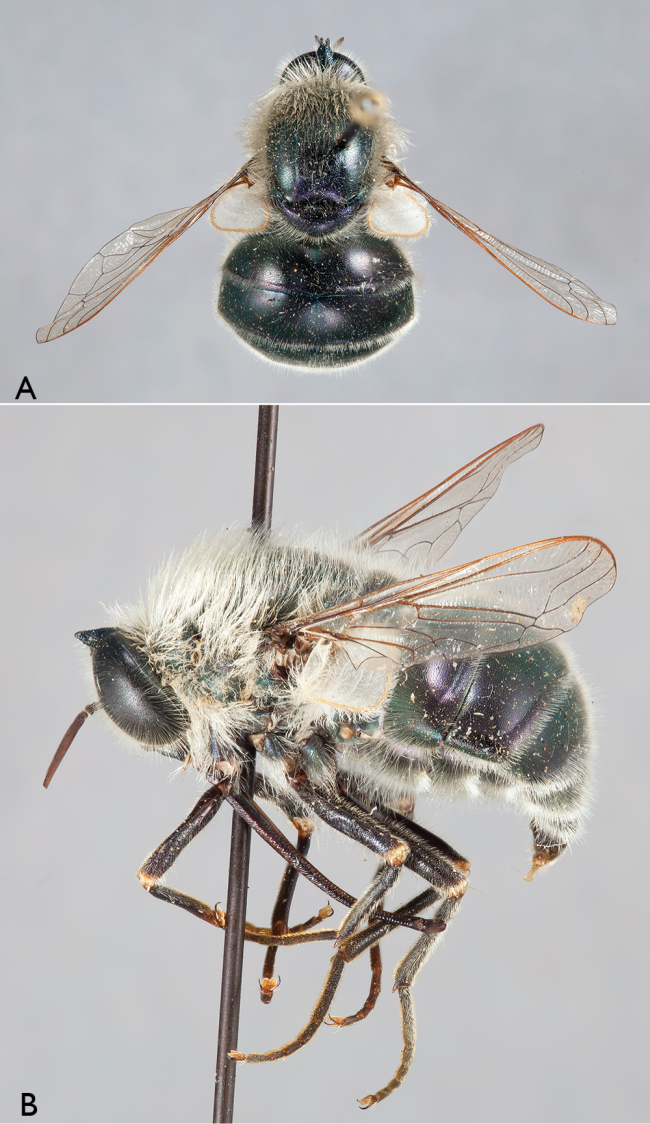
*Eulonchus halli* Schlinger, male (EIS 003122). **A** dorsal view **B** lateral view. Body length: 11.4 mm.

**Figure 3.**
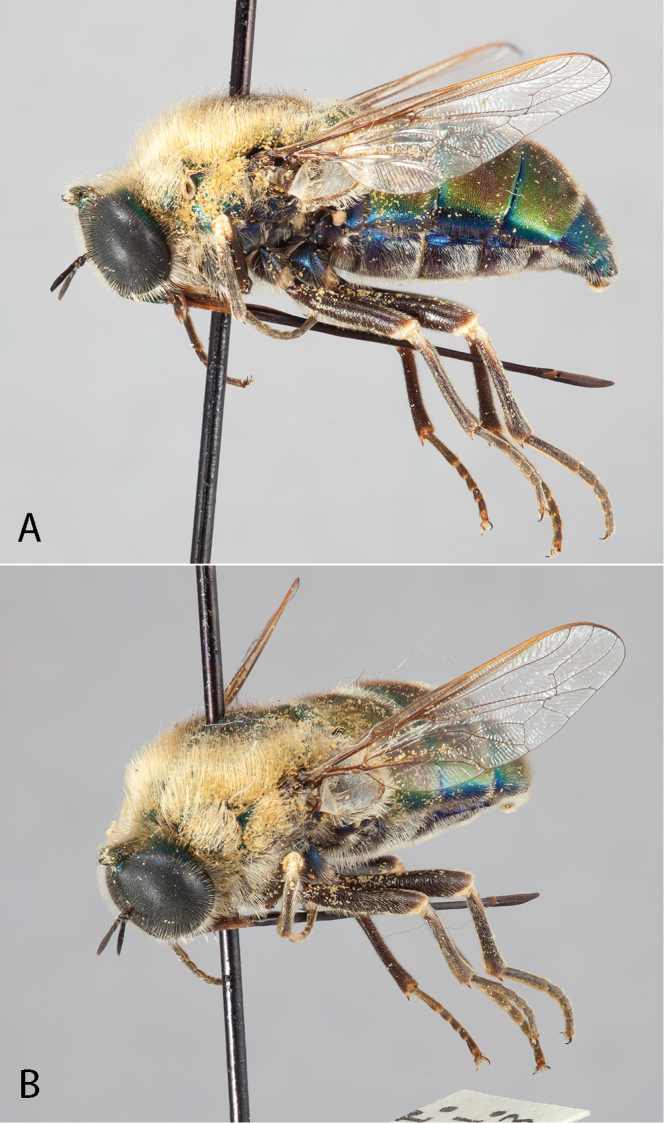
*Eulonchus marginatus* Osten Sacken, male (EIS 000290). **A** lateral view **B** oblique view. Body length: 12.2 mm.

**Figure 4.**
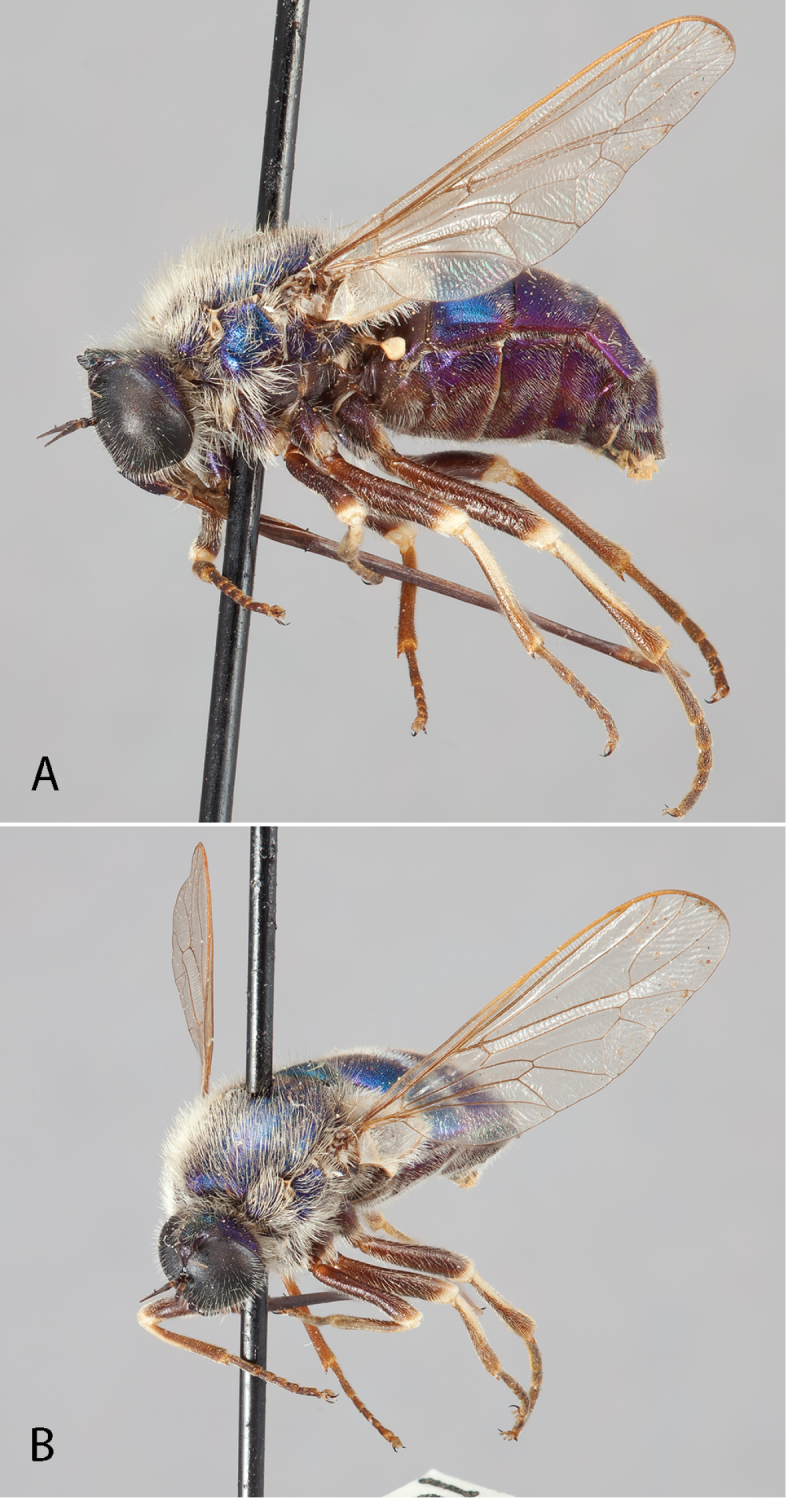
*Eulonchus marginatus* Osten Sacken, female (EIS 000370). **A** lateral view **B** oblique view. Body length: 8.7 mm.

**Figure 5.**
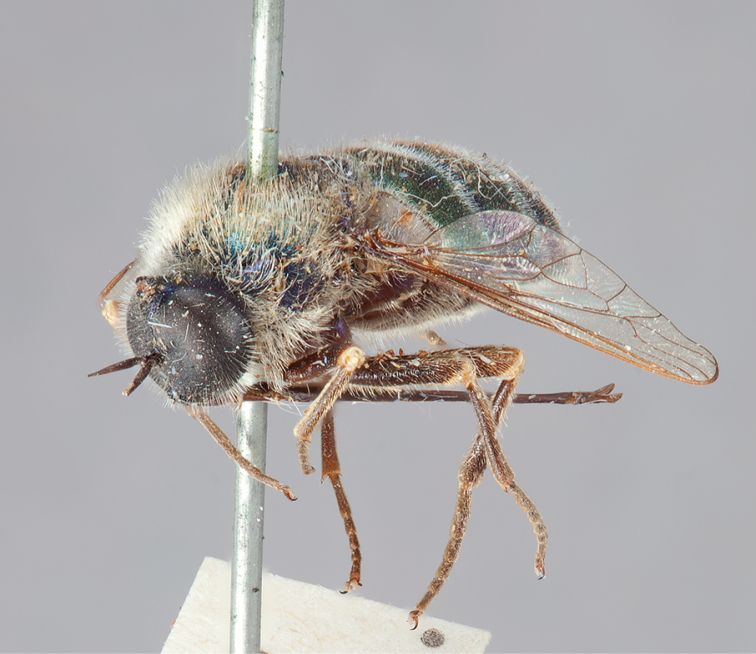
*Eulonchus marginatus* Osten Sacken, holotype, male, oblique view. Body length: 8.9 mm.

**Figure 6.**
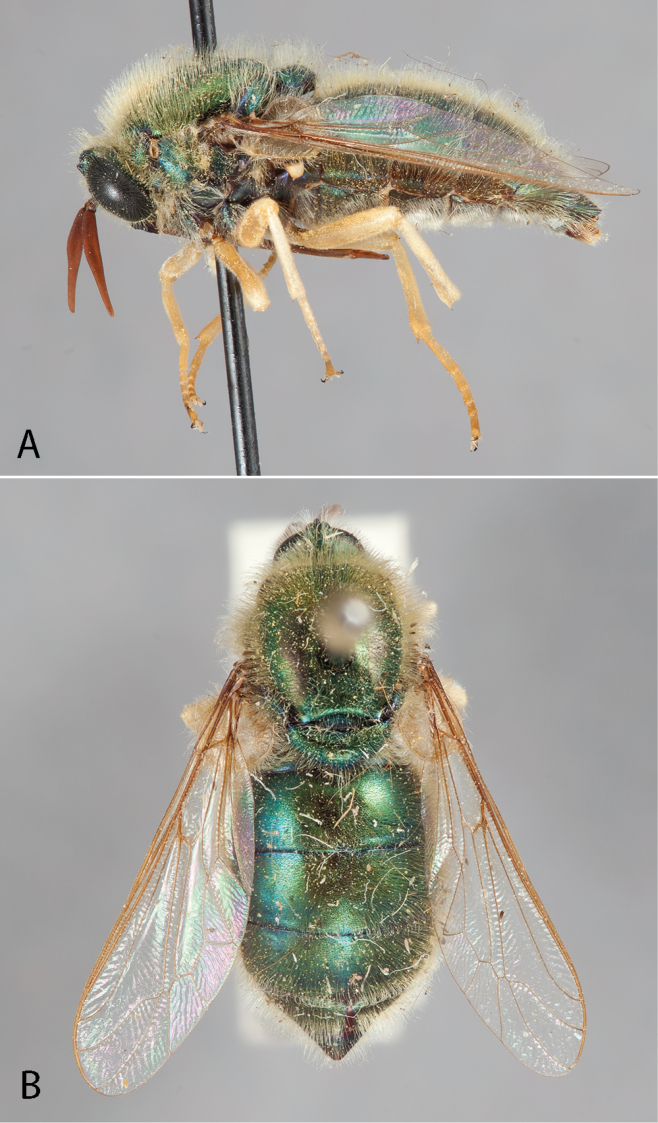
*Eulonchus marialiciae* Brimley, male (EIS 014839). **A** lateral view **B** dorsal view. Body length: 11.0 mm.

**Figure 7.**
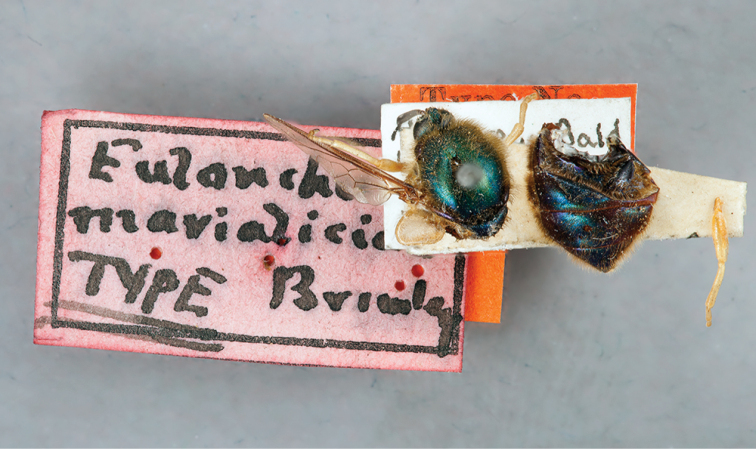
*Eulonchus marialiciae* Brimley, holotype, male. Body length: ~12.0 mm.

**Figure 8.**
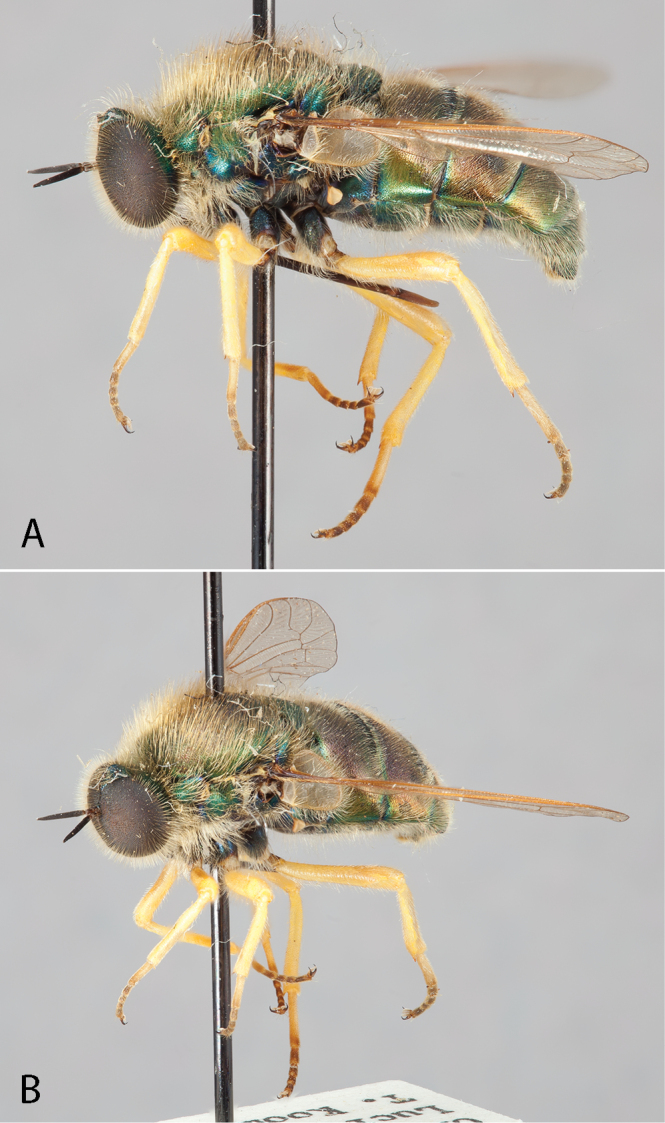
*Eulonchus sapphirinus* Osten Sacken, male (EIS 013101). **A** lateral view **B** oblique view. Body length: 10.2 mm.

**Figure 9.**
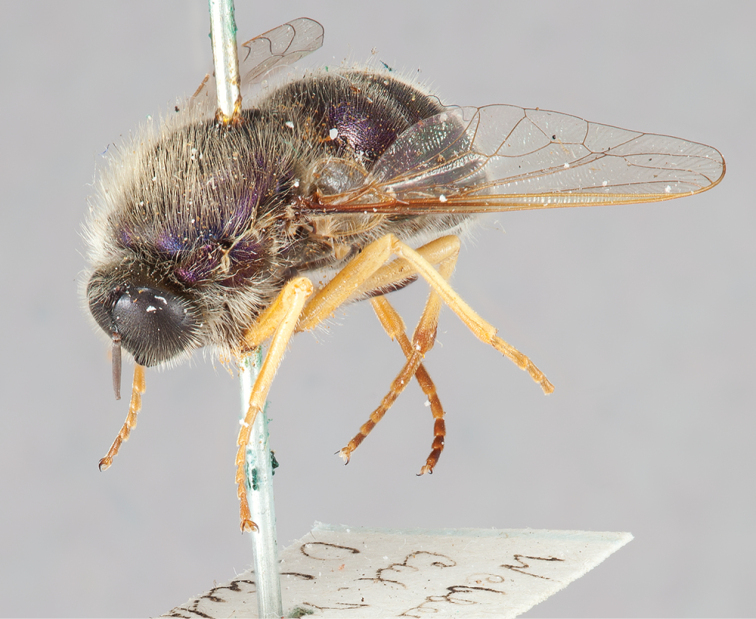
*Eulonchus sapphirinus* Osten Sacken. Paralectotype male. Body length: 9.0 mm.

**Figure 10.**
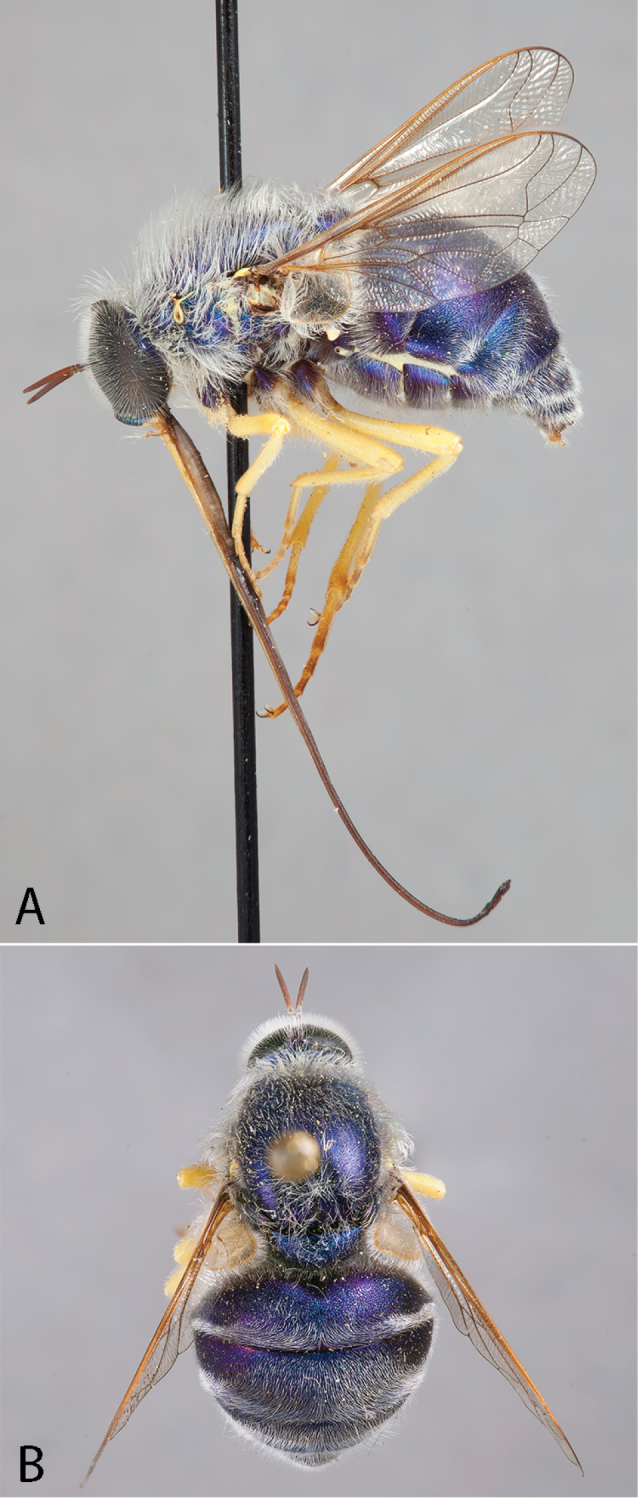
*Eulonchus smaragdinus* Gerstaecker, male (EIS 000465). **A** lateral view **B** dorsal view. Body length: 11.4 mm.

**Figure 11.**
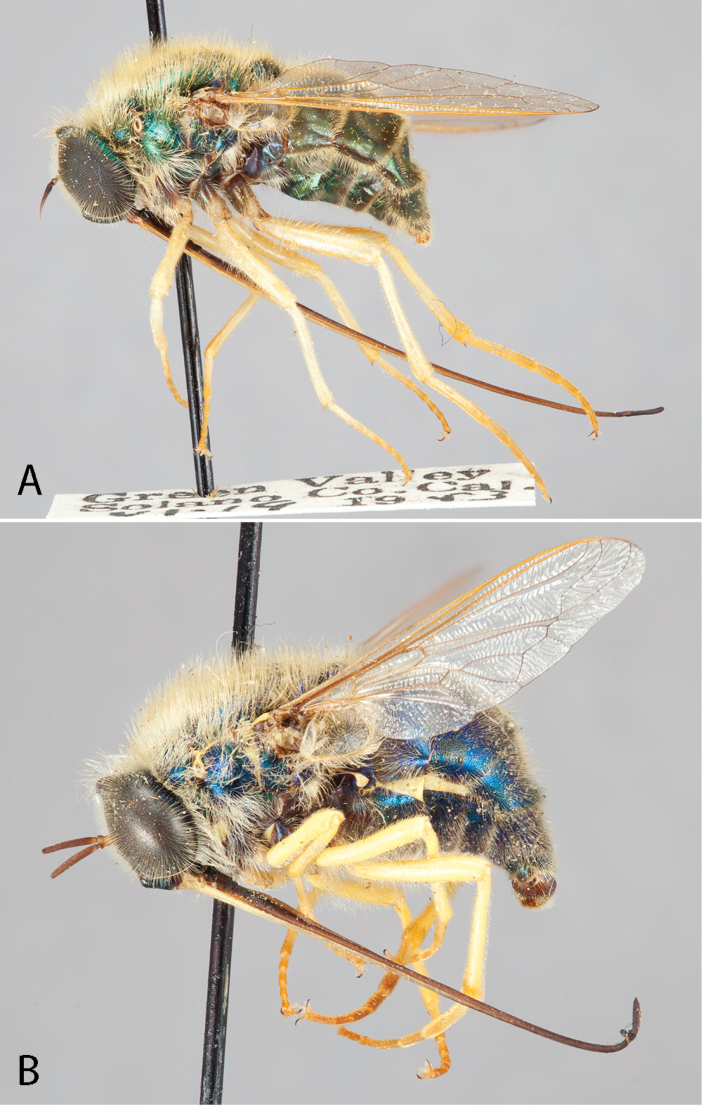
*Eulonchus smaragdinus* Gerstaecker. **A** Female (EIS 000191), lateral view. Body length: 11.1 mm **B** Male (EIS 000027), lateral view. Body length: 10.1 mm.

**Figure 12.**
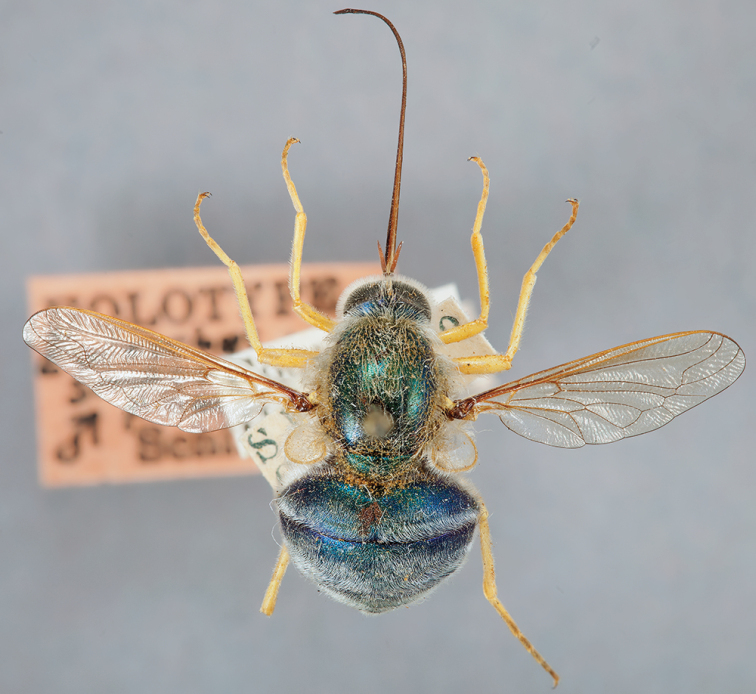
*Eulonchus smaragdinus* Gerstaecker (*Eulonchus smaragdinus pilosus* Schlinger holotype), dorsal view. Body length: 11.9 mm.

**Figure 13.**
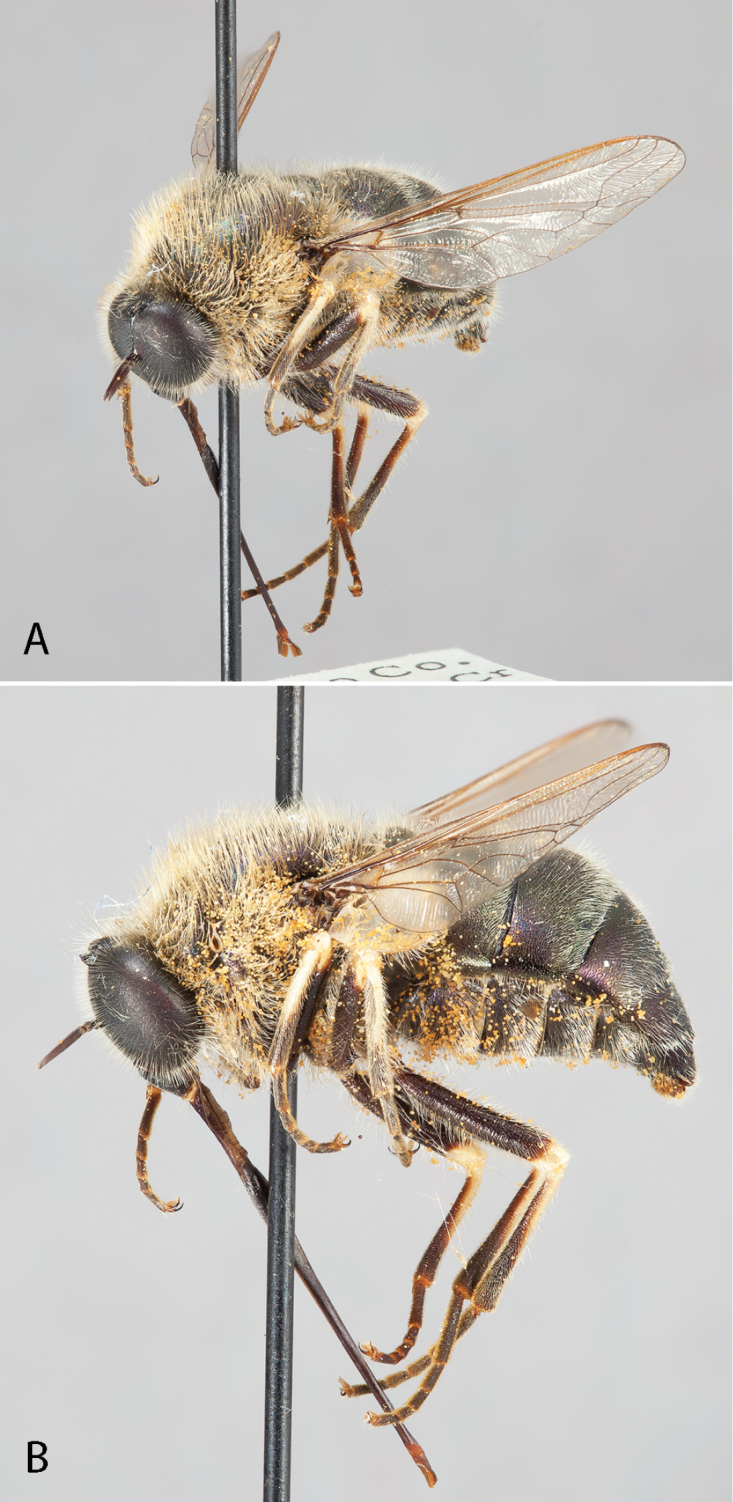
*Eulonchus tristis* Loew, male (EIS 008947). **A** oblique view **B** lateral view. Body length: 10.6 mm.

**Figure 14.**
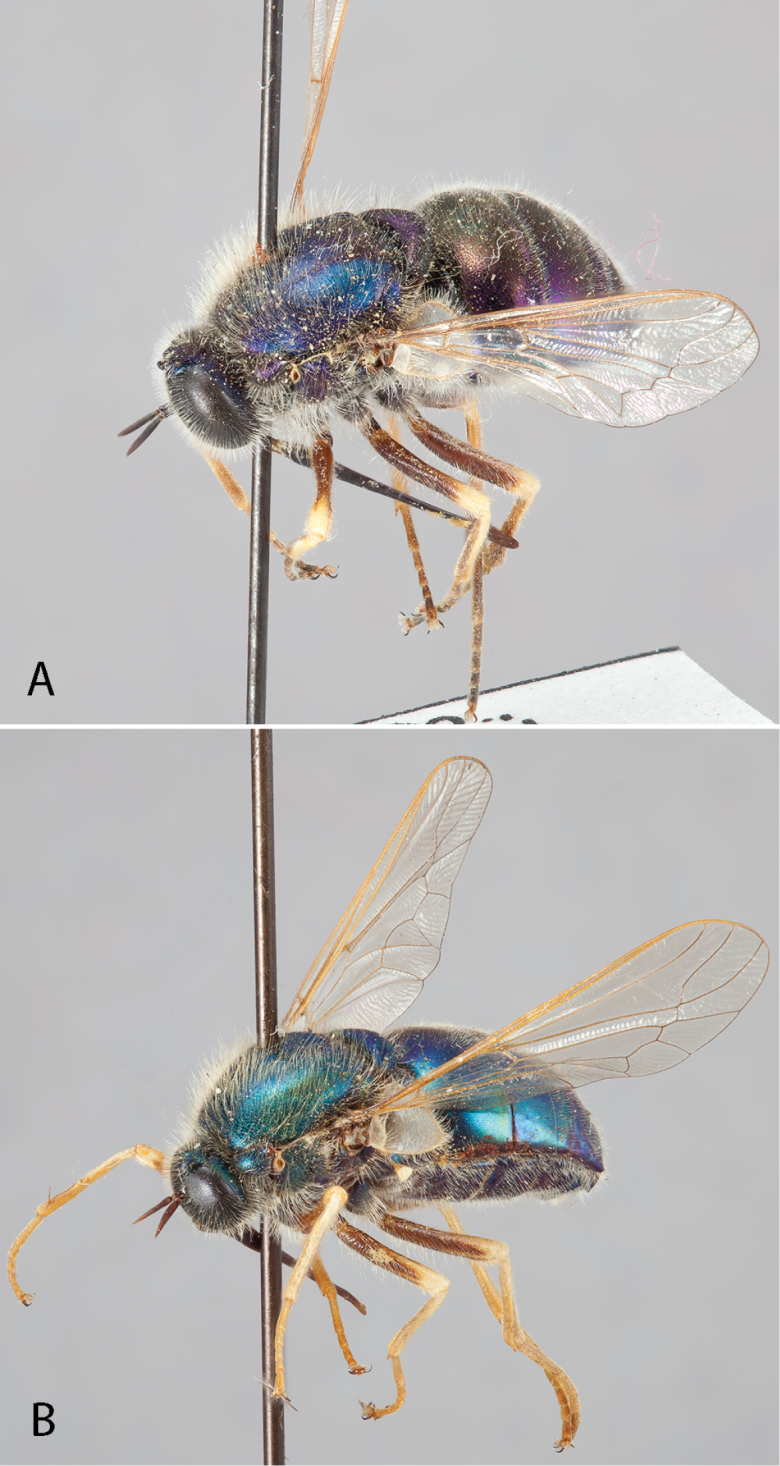
*Eulonchus tristis* Loew. **A** oblique view, male (EIS 009592), body length: 10.0 mm **B** oblique view, female (EIS 017865), body length: 10.8 mm.

**Figure 15.**
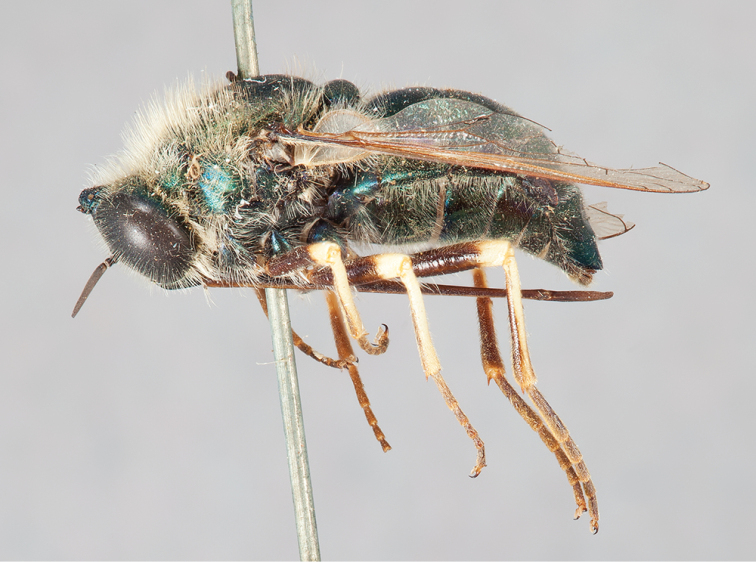
*Eulonchus tristis* Loew, lectotype male, lateral view. Body length: 9.8 mm.

**Figure 16.**
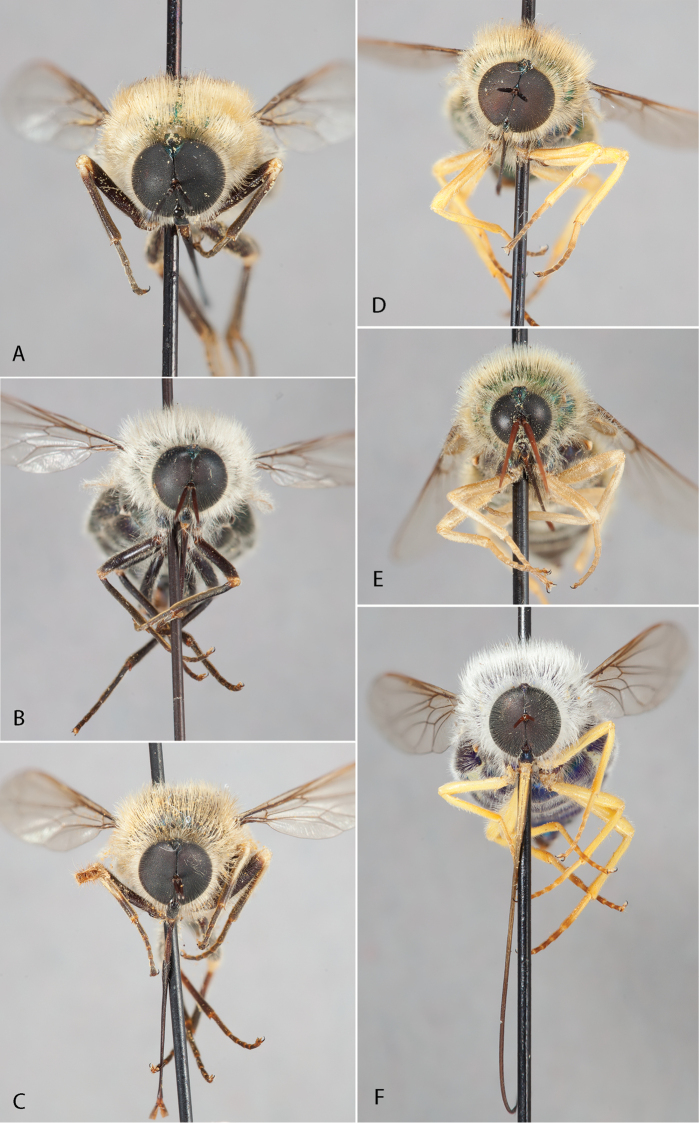
*Eulonchus* species, anterior view. **A** *Eulonchus marginatus* **B** *Eulonchus halli* **C** *Eulonchus tristis* **D** *Eulonchus sapphirinus* **E** *Eulonchus marialicae* **F** *Eulonchus smaragdinus*.

**Figure 17.**
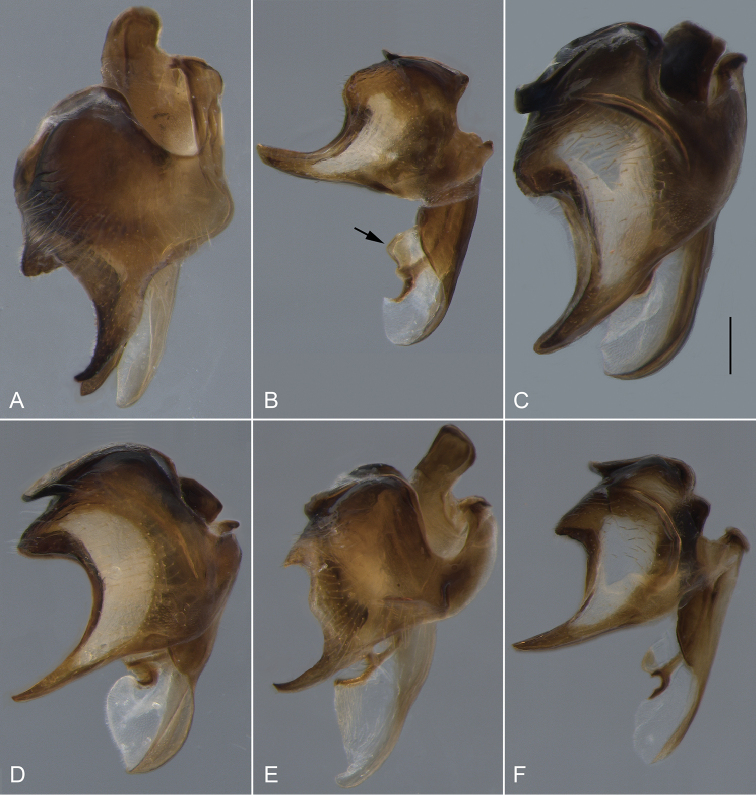
Gonocoxite and aedeagus, lateral view. **A** *Eulonchus halli* (EIS 004397) **B** *Eulonchus marginatus* (EIS 009172) **C** *Eulonchus marialiciae* (EIS 017824) **D** *Eulonchus sapphirinus* (EIS 017824) **E** *Eulonchus smaragdinus* **F** *Eulonchus tristis* (EIS 014098). Scale bar: 0.5 mm.

**Figure 18.**
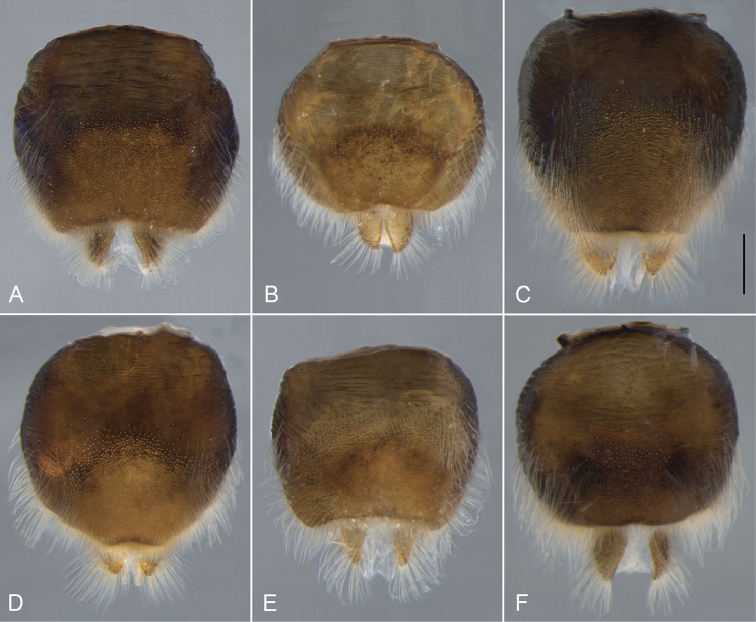
Epandrium, lateral view. **A** *Eulonchus halli* (EIS 004397) **B** *Eulonchus marginatus* (EIS 009172) **C** *Eulonchus marialiciae* (EIS 017824) **D** *Eulonchus sapphirinus* (EIS 017824) **E** *Eulonchus smaragdinus* **F** *Eulonchus tristis* (EIS 014098). Scale bar: 0.5 mm.

**Figure 19.**
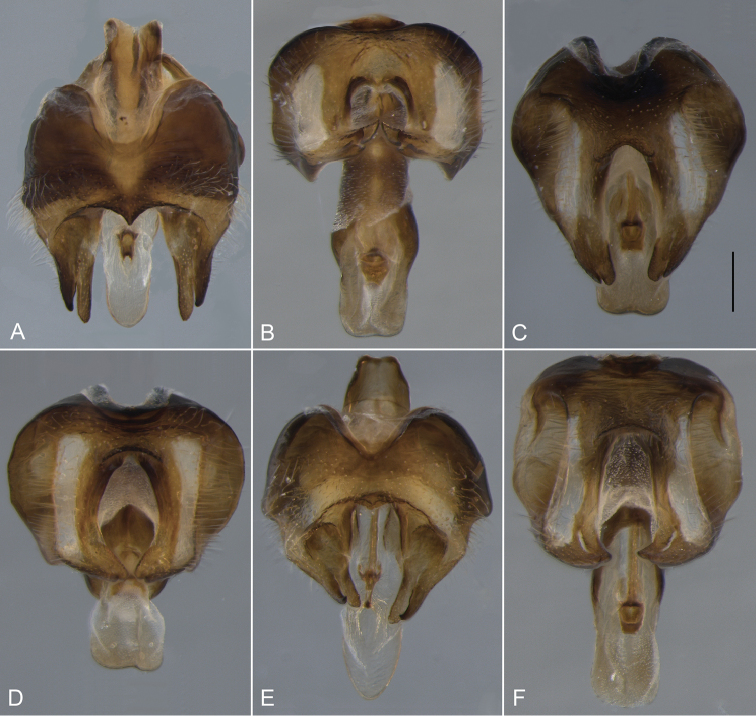
Gonocoxite, ventral view. **A** *Eulonchus halli* (EIS 004397) **B** *Eulonchus marginatus* (EIS 009172) **C** *Eulonchus marialiciae* (EIS 017824) **D** *Eulonchus sapphirinus* (EIS 017824) **E** *Eulonchus smaragdinus* **F** *Eulonchus tristis* (EIS 014098). Scale bar: 0.5 mm.

#### Included species.

*Eulonchus halli* Schlinger, 1960: 418; *Eulonchus marginatus* Osten Sacken, 1877: 277; *Eulonchus marialiciae* Brimley, 1925: 77; *Eulonchus sapphirinus* Osten Sacken, 1877: 276; *Eulonchus smaragdinus* Gerstaecker, 1856: 360 (including *Eulonchus smaragdinus smaragdinus* Gerstaecker, 1856 and *Eulonchus smaragdinus pilosus* Schlinger, 1960); *Eulonchus tristis* Loew, 1872: 60.

#### Distribution

**Figure 20.**
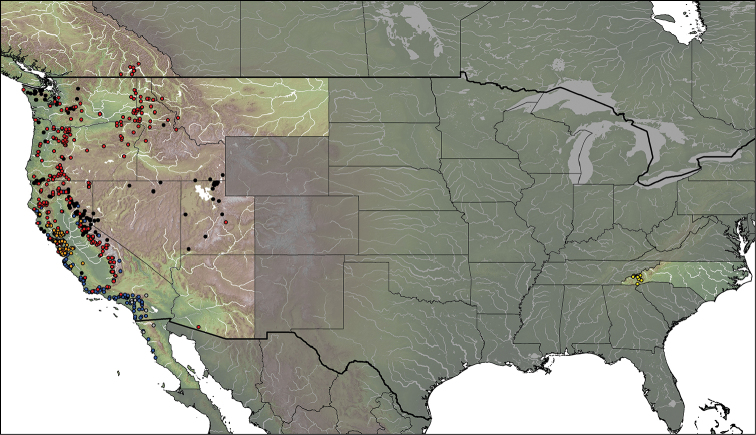
Distribution of *Eulonchus* species. Grey circles: *Eulonchus halli*; orange circles: *Eulonchus marginatus*; yellow circles: *Eulonchus marialiciae*; black circles: *Eulonchus sapphirinus*; blue circles: *Eulonchus smaragdinus*; red circles: *Eulonchus tristis*.

**Figure 21.**
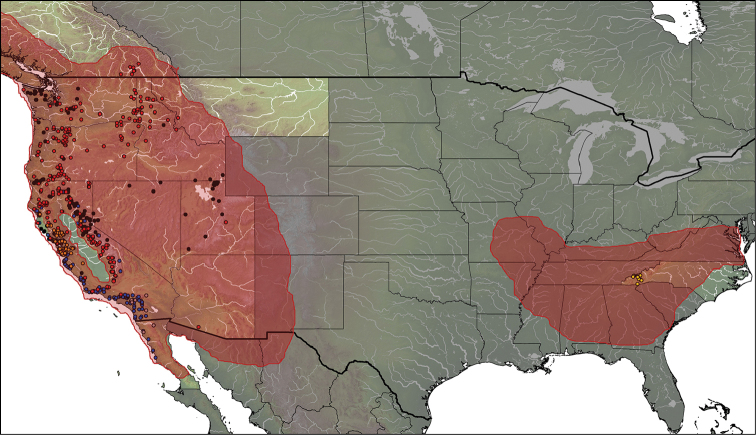
Distribution of species of *Eulonchus* and their hosts. Coloured circles: *Eulonchus* spp.; red shaded area: potential Antrodiaetidae spider fly host distribution inferred from Coyle 1971, Hendrixson and Bond 2006, and Bond 2012.

(Fig. 20). *Eulonchus* is distributed throughout the Nearctic, with *Eulonchus marialiciae* found in the eastern state of North Carolina and the remaining species distributed throughout the USA west of the Rocky Mountains, northwards to Canada and south to Baja California, Mexico (Fig. 20). There is significant overlap in the distribution of species of *Eulonchus* with that of the host (or presumed host) spiders in the families Antrodiaetidae (*Aliatypus* sp., *Antrodiaetus riversi* Cambridge, *Antrodiaetus unicolor* Hentz) and Euctenizidae (*Aptostichus stanfordianus* Smith) (Fig. 21) (Schlinger 1987), with the range of *Eulonchus* being clearly inside the range of its hosts. Antrodiaetidae comprise two genera, *Aliatypus* Smith, and *Antrodiaetus* Ausserer, with a disjunct distribution in Japan and North America (Coyle 1971; Hendrixson and Bond 2006). Nearctic antrodiaetids inhabit three primary geographic regions: the deciduous forests of the eastern United States (USA), the forested ‘sky islands’ of the southwestern USA, and various habitats throughout the northwestern USA and adjacent portions of southwestern Canada (Hendrixson and Bond 2006). The euctenizid genus *Aptostichus* Simon is a genus of trapdoor spiders found predominantly in southern California; *Aptostichus stanfordianus* is commonly called the Stanford Hills Trapdoor Spider, and is distributed throughout California (Bond 2012).

#### Conservation.

Most species of *Eulonchus* are relatively widely distributed, and locally abundant, except the sole eastern North American species *Eulonchus marialiciae*. This species is known from only a few specimens, all found within a small contiguous area in the Great Smoky Mountains, with collections from Haywood, Jackson, Macon, Transylvania, and Swain counties in North Carolina. Even though the spider host and floral food source of *Eulonchus marialiciae* are distributed over much of western North Carolina, southwestern Virginia, and eastern Tennessee, the species is only found in five mountainous counties of western North Carolina, at elevations of 1250m or higher. Considering the apparent limited and very isolated distribution of this species, the conservation status of *Eulonchus marialiciae* should be further evaluated. Future studies should focus on identifying potential extralimital populations of this species outside of the presently known distribution, as well as understanding potential biotic (*e.g.* spider host and plant food source range and distribution) and abiotic (*e.g.* elevation, climate, vegetation and soil type) factors associated with the apparent limited distribution of this species.

#### Biology.

Adult *Eulonchus* are locally abundant on flowers (Cole 1919; Schlinger 1960) and exhibit behaviours that show constancy to particular plant species (Borkent and Schlinger 2008a, b). Thus, North American jewelled spider flies may form a large and important component of the pollinator fauna for some plant species. *Eulonchus* visit species from more than 30 plant families (see Table 2), and in multiple habitats (Borkent and Schlinger 2008a, b). However, the ability of *Eulonchus* species to serve as pollinators needs to be further studied.

**Table 2.** Specimen label and literature records of plants visited by *Eulonchus* species. The genus has also been recorded visiting flowers of Brassicaceae (*Cakile*), Fabaceae (*Lotus nevadensis*), Hydrophyllaceae (*Nama parryi*), Ranunculaceae (*Delphinium*, *Ranunculus*), and Saxifragaceae (*Lithophragma*).

Plant family	Plant visited	*Eulonchus s*pecies visiting					
		*Eulonchus halli*	*Eulonchus marginatus*	*Eulonchus marialiciae*	*Eulonchus sapphirinus*	*Eulonchus smaragdinus*	*Eulonchus tristis*
Asteraceae	*Cirsium*		X			X	X
	*Cirsium cymosum*						X
	*Erigeron*				X		
	*Wyethia*						X
Boraginaceae	*Cryptantha intermedia*	X					
	*Eriodictyon californicum*		X			X	X
	*Hackelia bella*						X
	*Hackelia floribunda*				X		
	*Myosotis*				X		
	*Myosotis sylvatica*				X		
	*Phacelia*				X		
	*Phacelia heterophylla*					X	
Caprifoliaceae	*Linnaea borealis*				X		
Caryophyllaceae	*Stellaria crispa*				X		X
Convolvulaceae	*Convolvulus*				X	X	
Crassulaceae	*Dudleya cultrata*					X	
Ericaceae	*Azalea occidentalis*						X
	*Rhododendron*						X
	*Rhododendron occidentale*				X		
	*Vaccinium*						X
	*Vaccinium ovatum*				X		X
Gentianaceae	*Frasera tubulosa*						X
Geraniaceae	*Geranium robertianum*				X		
Grossulariaceae	*Ribes cereum*						X
	*Ribes roezlii*						X
Iridaceae	*Iris douglasiana*		X		X		X
	*Iris hartwegii*				X		X
Iridaceae	*Iris macrosiphon*						X
	*Iris purdyi*					X	X
Lamiaceae	*Lepechinia calycina*		X				
	*Monardella*				X		
	*Monardella lanceolata*						X
	*Monardella odoratissima*						X
	*Salvia carduacea*				X		
	*Salvia clevelandi*						X
Liliaceae	*Clintonia uniflora*				X		X
Melanthiaceae	*Veratrum*				X		
Montiaceae	*Calyptridium umbellatum*				X		
	*Lewisia cotyledon*				X		
	*Montia*						
Onagraceae	*Clarkia*						X
	*Clarkia breweri*		X				
	*Clarkia concinna*		X			X	X
Orchidaceae	*Corallorhiza maculata*				X		
Oxalidaceae	*Oxalis oregana*						X
Papaveraceae	*Eschscholzia californica*					X	
Phrymaceae	*Mimulus aurantiacus*		X			X	X
	*Mimulus glutinosus*				X	X	
	*Mimulus guttatus*		X				
	*Mimulus longiflorus*				X		
Plantaginaceae	*Penstemon*		X			X	
	*Penstemon cardwelli*				X		
	*Penstemon heterophyllus*		X			X	
	*Veronica americana*						X
Polemoniaceae	*Gilia capitata*					X	
	*Gilia splendens*					X	
	*Leptosiphon*						X
Polemoniaceae	*Leptosiphon bicolor*					X	
	*Linanthus*						X
	*Linanthus androsaceus*					X	
	*Microsteris gracilis*				X		
	*Navarretia capitatus*		X				
Polygonaceae	*Eriogonum*						X
Primulaceae	*Trientalis borealis*				X		
Rhamnaceae	*Ceanothus cordulatus*						X
	*Ceanothus integerrimus*				X		
Rosaceae	*Chamaebatia foliolosa*				X		
	*Fragaria vesca*				X		
	*Prunus emarginata*				X		
	*Rubus*			X			
	*Rubus canadensis*			X			
	*Rubus parviflorus*						X
	*Rubus ursinus*				X		
Salicaceae	*Salix*						X
Themidaceae	*Brodiaea*					X	X
	*Brodiaea bridgesii*						X
	*Brodiaea congesta*		X				
	*Brodiaea elegans*		X			X	X
	*Brodiaea lutea*						X
	*Brodiaea pulchella*						X
	*Dichelostomma multiflora*						X
	*Triteleia ixioides*						X
Verbenaceae	*Verbena*		X				

Coyle (1971) reported a large aggregation of *Eulonchus marialiciae* forming as he was excavating burrows of *Antrodiaetus unicolor* in North Carolina. A relatively large number of flies (~12 individuals) approached very quickly, hovering close to the ground where the burrows were being excavated, apparently attracted by some chemical released during the process. The author observed multiple adults hovering over and landing near closed burrow entrances. As mentioned above, one of us (C. Borkent) has observed that the newly hatched first instar acrocerid larvae actively search for their hosts, rearing up in search of a spider. Once successful they subsequently penetrate their cuticle and develop as an endoparasitoid (Schlinger 1987). Additionally, Coyle (1971) observed several instances in which the larva, after feeding on the spider in the bottom end of the burrow, climbed up the burrow wall, attached somewhere above the bottom end and completed development within the burrow.

#### Comments.

Species of *Eulonchus* are very similar to species of *Lasia* and *Apsona*, and these three genera, as wells as some species of *Panops*, are commonly known as jewelled spider flies due to their metallic body colouration. *Eulonchus* species are commonly called Emeralds or Sapphires, depending on the body colour. Winterton (2012) described the thoracic pile in the Australian genus *Panops* as being reflective, thus brighter when the individual was viewed anteriorly, a characteristic of many Old World panopine species. This character is absent in *Eulonchus* and most New World panopine genera, with the thoracic pile being of similar brightness regardless of which angle it is viewed. Phylogenetic relationships among Panopinae genera are still poorly known. Based on DNA sequence data, Winterton et al. (2007) found *Eulonchus* to be placed between *Lasia* and more derived genera such as *Ocnaea*, *Archipialea*, and *Arrhynchus*.

#### Phylogenetic relationships

**Figure 22.**
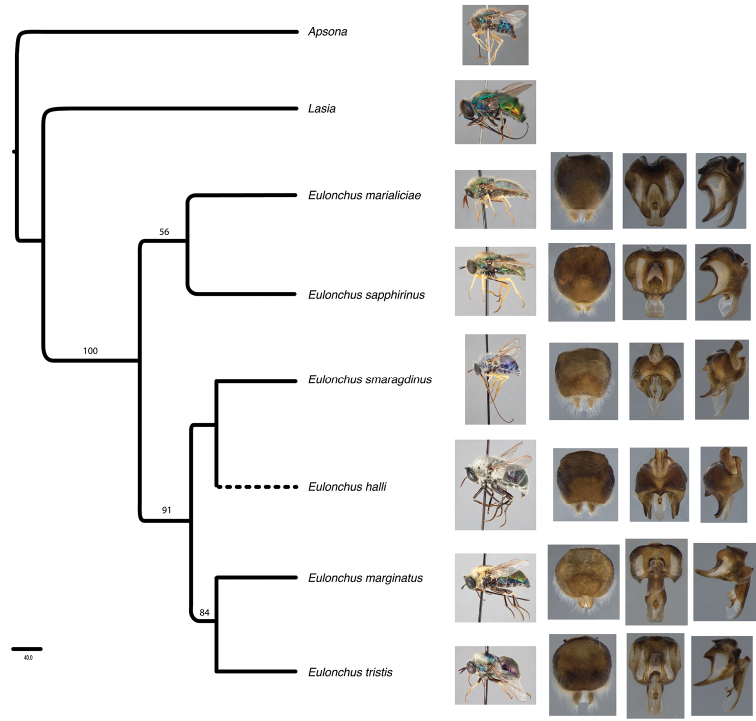
Phylogeny of *Eulonchus* based on DNA sequence data (16S, COI, CAD). Single tree resulting from branch and bound parsimony search. Bootstrap support values are shown above the branches.

(Fig. 22). The phylogeny performed in this study is based on DNA sequence data and includes five of the six species of *Eulonchus*. The parsimony analysis resulted in a single most parsimonious tree with length = 904, consistency index (CI) = 0.92, and retention index (RI) = 0.62. The eastern species *Eulonchus marialiciae* (Fig. 6) and the northwestern *Eulonchus sapphirinus* (Figs 8–9) were recovered as sister taxa in a clade that is sister to the remaining species of the genus. Even though the support for the branch was low (Fig. 22) we are confident in this relationship as *Eulonchus marialiciae* and *Eulonchus sapphirinus* share multiple characters, such as the ovate epandrium (which is thinner at the apex; Fig. 18C–D), the gonocoxite taller than wide (Fig. 19C–D), and the aedeagus broad at the apex and not heavily laterally sclerotized (Fig. 17C–D). These two species are typically bright metallic green or blue with yellow legs. This feature is shared with some individuals of the highly variable *Eulonchus smaragdinus* from southern California, USA and Baja California, Mexico. *Eulonchus smaragdinus* (Figs 10–11) was recovered as an intermediate species that subtends the clade comprising the more northern species, *Eulonchus tristis* and *Eulonchus marginatus*. *Eulonchus halli* was not included in the phylogenetic analysis due to lack of fresh material for DNA extraction. However, placement of the species in the *Eulonchus* tree was postulated based on morphology (see dashed lines on Fig. 20). We hypothesize that *Eulonchus halli* is more closely related to *Eulonchus smaragdinus* based on their bifid ocellar tubercles, epandrium somewhat rectangle shaped and wide at the apex, gonocoxite deeply emarginate along anterior margin, fenestrae lacking (Fig. 19A, E) and aedeagus thin at the apex (Fig. 17A, E). The sister-grouping was already suggested by Schlinger (1960). *Eulonchus tristis* (Figs 13–14) and *Eulonchus marginatus* (Figs 3–4) have dark brown coloured legs and a thorax and abdomen with dark metallic to brownish-black colouration. *Eulonchus tristis* is also a highly morphologically variable species with body colour ranging from metallic light blue (Fig. 14) to dark brown (Fig. 13). These two species share multiple genitalia characters such as a somewhat round epandrium, gonocoxite with anterior margin almost straight, with large fenestrae, and aedeagus heavily sclerotized laterally.

#### Key to species of *Eulonchus*

**Table:** 

1	Femora and tibiae completely bright yellow	**2**
–	Femora completely dark brown, or light to dark brown with dark yellow or cream portion distally; tibiae completely dark brown, or brown with dark yellow to cream basally	**4**
2	Flagellum laterally compressed, thicker than scape and pedicel, pendulous, and ~twice length of head; proboscis length rarely extending beyond midpoint of abdomen, typically extending to end of thorax (eastern North America)	***Eulonchus marialiciae* Brimley**
–	Flagellum not laterally compressed, basal portion ~equal to thickness of scape and pedicel, erect, and equal to length of head; proboscis extending beyond midpoint of abdomen, often longer than body (western North America)	**3**
3	Proboscis approximately equal to body length, straight; ocellar tubercle typically taller than wide and apex irregularly trifid; median ocellus often weakly developed	***Eulonchus sapphirinus* Osten Sacken**
–	Proboscis distinctly longer than body length, curved at tip; ocellar tubercle wider than tall and apex weakly bifid; median ocellus clearly defined	***Eulonchus smaragdinus* Gerstaecker**
4	Ocellar tubercle bifid in anterior view; flagellum as long as the head	***Eulonchus halli* Schlinger**
–	Ocellar tubercle trifid; flagellum half as long as the head	**5**
5	Margin of calypter light yellow to light brown; femur and tibia light to dark brown with variable amounts of yellow or ivory patterning; pile on the abdomen yellow to light yellow (highly variable species); aedeagus in lateral view with a single rounded point dorsally just before opening of the aedeagus (Fig. 17F)	***Eulonchus tristis* Loew**
–	Margin of calypter dark brown with brown bleeding into calypter membrane; femur completely dark brown, tibia usually dark brown, occasionally with cream yellow on dorsal surface basally (joint between femur and tibia always yellow); pile on abdomen white; aedeagus in lateral view with two rounded points prior to the opening of the aedeagus (Fig. 17B)	***Eulonchus marginatus* Osten Sacken**

### Eulonchus halli

Taxon classificationAnimaliaDipteraAcroceridae

Schlinger, 1960

Figs 2 , 16B , 17A , 18A , 19A

Eulonchus halli Schlinger, 1960: 418

#### References.

Arnaud 1979: 203 (holotype depository); Schlinger 1981: 576 (figs); Poole 1996: 36 (checklist).

#### Common name.

Hall’s Sapphire.

#### Diagnosis.

Proboscis curved and extending beyond abdomen apex (but shorter than wing length), ocellar tubercle bifurcate; calypter margin pale; legs dark brown-black; abdomen dark brown with reddish metallic hue; extensive white pile on thorax and as bands on abdomen.

#### Redescription.

Body length: 9.8–12.0 mm, Wing length 8.0–10.6 mm. *Head*. Flagellum dark brown, scape and pedicel brown; male flagellum cylindrical, shorter than head height; clypeus elongate, extending beyond oral cavity, shape rounded with raised ridge dorsally, surface glossy black-brown with sparse pubescence; labial palp brown, extending anteriorly beyond proboscis at point of attachment; margin of oral cavity (parafacial) finely pubescent, lacking pile; proboscis length extending to middle of abdomen; ocellar tubercle bifurcate, apices narrowly digitate and sub-parallel, tubercle height taller than width, median ocellus greatly reduced; occiput metallic blue or metallic purple, occipital pile densely white. *Thorax*. Metallic green, metallic blue or metallic purple in colour, setal pile white; coxae black with metallic blue sheen; femora dark brown, apices white; tibia brown; tarsi brown; calypter margin yellow or light brown, membrane translucent; haltere entirely dark brown. *Abdomen*. Metallic green, metallic blue violet or black with metallic green sheen, vestiture white, dominant setae erect, other pile posteriorly directed, marginal band of denser setae on T3–4. *Male genitalia* (Figs 17A, 18A, 19A). Epandrium rectangular, wide at the apex, with posterior margin concave; gonocoxite deeply emarginate along anterior margin, fenestrae lacking; aedeagus thinned at the apex, only slightly sclerotized.

#### Type material examined.

**Holotype** male, CAS, “Riverside/ Cal. *Mar 7, 34*” [white]; “Timberlake/ Coll.” [white]; “*on Cryptantha*/ *intermedia*” [white]; “HOLOTYPE/ *Eulonchus*/ *halli*/ ♂ Schlinger” [orange]; “Genitalia *51-3-id*/ Dissection No./ by E.I. Schlinger” [white]; “California Academy/ of Sciences/ Type No. *6392*” [white]; “IMAGE” [green]; “CAS/ Dec-07” [green]; specimen condition: good, proboscis, right flagellum and right front leg broken off and glued to a paper triangle on pin with specimen. Body length: 10.2 mm, Wing length: 9.2 mm.

#### Other material examined.

Listed in Table 3 (Suppl. material 1).

#### Distribution

(Fig. 20). Nearctic: Southern California (USA) to Baja California (Mexico).

#### Ecology.

Schlinger (1960) notes that this species has a relatively short adult flight period (*ca.* six weeks) during spring and has a feeding preference on *Cryptantha intermedia* flowers (Table 2).

#### Biology.

The larval host for this species is unknown.

#### Comments.

*Eulonchus halli* is a distinctive species that can be distinguished by the dark leg and body colouration, erect white thoracic and abdominal pile, raised bifurcate ocellar tubercle, and a curved proboscis that is shorter than the body length.

### Eulonchus marginatus

Taxon classificationAnimaliaDipteraAcroceridae

Osten Sacken, 1877

Figs 3 , 4 , 5 , 16A , 17B , 18B , 19B

Eulonchus marginatus Osten Sacken, 1877: 277

#### References.

Osten Sacken 1878: 99 (catalogue); Aldrich 1905: 221 (catalogue); Kertész 1909: 12 (catalogue); Cole 1919: 38 (key notes, figs), 1923: 46 (California); Sabrosky 1948: 388 (key); Cole 1969: 221 (notes); Poole 1996: 36 (checklist).

#### Common name.

Sombre Sapphire or Emerald.

#### Diagnosis.

Proboscis straight, approximately reaching apex of abdomen; ocellar tubercle trifurcate; legs dark brown (pale ‘knee’ joint); calypter margin black or brown.

#### Redescription.

Body length: 7.2–11.4 mm, wing length: 5.2–9.5 mm. *Head*. Flagellum dark brown; scape and pedicel brown, male flagellum cylindrical, shorter than head height; clypeus elongate, extending beyond oral cavity, shape rounded with flat area dorsally, clypeus black-brown, glossy with sparse pubescence; labial palp brown, extending anteriorly beyond proboscis at point of attachment; margin of oral cavity (parafacial) pilose, admixed with short pubescence; ocellar tubercle trifurcate, processes narrow (anteromedial process taller), height taller than width; median ocellus greatly reduced or absent; occiput metallic green-blue, metallic blue or metallic purple, pile densely white or yellow. *Thorax*. Metallic green, blue or purple, setal pile erect, white or yellow; coxae black with metallic blue sheen; femora dark brown, apices white; tibiae brown (whitish basally on dorsal surface); tarsi brown; calypter margin light to dark brown, membrane translucent, with suffused brown marginally; haltere stem dark brown, knob lighter brown. *Abdomen*. Metallic olive green, green or blue-violet, vestiture white or yellow, dominant setae erect, pile posteriorly directed, marginal band of laterally directed pile on T2–4. *Male genitalia*. Epandrium round, with posterior margin concave; gonocoxite with anterior margin almost straight, with large fenestrae; aedeagus heavily sclerotized laterally, with a secondary dorsal point just prior to the opening of the aedeagus.

#### Type material examined.

**Holotype** male, MCZ, “*Napa Co*/ California.” [white]; “*O. Sacken.*/ *West Dipt.*” [white]; “Type/ *1078*” [red and white]; “*Eulonchus*/ *marginatus*/ *O.S.*” [white]”; “Eug-Dec 2006/ MCZ Image/ Database” [white]; “MCZ-ENT/ 00001078” [white]; “HOLOTYPE ♂/ Eulonchus marginatus/ Osten Sacken/ Det. C.J. Borkent 2015” [red]; specimen condition: excellent, no parts missing. Body length: 8.9 mm, Wing length: 7.0 mm.

#### Other material examined.

Listed in Table 3 (Suppl. material 1).

#### Distribution

(Fig. 20). Nearctic: Northern California (USA).

#### Ecology.

*Eulonchus marginatus* has been recorded visiting the flowers of 10 different plant families and 14 different species (Table 2).

#### Biology.

Host unknown.

#### Comments.

*Eulonchus marginatus* is closely related to *Eulonchus tristis*, sharing features such as extensive white thoracic pile and dark colouration on the legs. *Eulonchus marginatus* is easily distinguished from other species in the genus by the leg colour, trifurcate ocellar tubercle and dark margin of the calypter. This species displays considerable variation in body colour, ranging from metallic green, blue to purple.

### Eulonchus marialiciae

Taxon classificationAnimaliaDipteraAcroceridae

Brimley, 1925

Figs 6 , 7 , 16E , 17C , 18C , 19C

Eulonchus marialiciae Brimley, 1925: 77

#### References.

Brimley 1938: 335 (North Carolina); Sabrosky 1948: 388 (key ref.); Schlinger 1965: 404 (catalogue); Coyle 1971: 281 (host *Antrodiaetus unicolor*, distr.); Poole 1996: 36 (checklist); Adler et al. 1997: 190 (biology, abundance).

#### Common name.

Mary-Alice’s Emerald.

#### Diagnosis.

Antennal flagellum elongate, basally broad and flattened laterally; proboscis straight, relatively short (~half body length); ocellar tubercle trifurcate; legs yellow; calypter margin brown; body colour metallic green.

#### Redescription.

Body length: 9.9–12.0 mm, Wing length: 9.1–10.2 mm. *Head*. Flagellum dark brown, male flagellum laterally compressed and variable in amount of distal tapering, longer than head height (pendulous in pinned specimen); scape and pedicel light brown to yellow; clypeus elongate, extending beyond oral cavity, rounded with raised ridge dorsally, surface black-brown, glossy with sparse pubescence; labial palp brown or yellow, length not extending beyond proboscis at point of attachment; margin of oral cavity (parafacial) glabrous or pilose, admixed with pubescence; proboscis straight, shorter than thorax or reaching middle of abdomen; ocellar tubercle trifurcate with processes relatively short and subequal (posterolateral processes often rounded), tubercle height shorter than width; median ocellus present; occiput metallic green-blue or blue, pile densely white or yellow. *Thorax*. Metallic green, blue or purple, setal pile yellow; coxae black with metallic blue and/or green sheen; femora yellow; tibiae dark yellow; tarsi dark yellow; calypter margin brown, membrane translucent, with suffused brown marginally; haltere stem dark brown, knob lighter brown. *Abdomen*. Metallic green or blue-violet, vestiture yellow, dominant setae erect. *Male genitalia* (Figs 17C, 18C, 19C). Epandrium ovate, thinned at the apex, with posterior margin straight; gonocoxite taller than wide, with broad fenestrae; aedeagus broad at the apex, bilobate in posterior view, not heavily sclerotized laterally.

#### Type material examined.

**Holotype** male, USNM, “*Andrews Bald*/ *IVIT.5 700ft*/ *Swain Co N.C.*/ *VI.26.23*” [white]; “JC Crawford/ Coll” [white]; “Type No./ *55797*/ U.S.N.M.” [red]; “*Eulonchus*/ *marialiciae*/ *TYPE Brimley*” [pink]; specimen condition: poor, all legs missing except left front and right mid leg, right wing missing, abdomen and a leg glued to a paper triangle on pin. Body length: ~12.0, (this is an approximation due to the disarticulation of the specimen), wing length: 10.2 mm.

#### Other material examined.

Listed in Table 3 (Suppl. material 1).

#### Distribution

(Fig. 20). Nearctic: North Carolina: Great Smoky Mountains: Macon, Swain and Hayward Counties (USA).

#### Ecology.

Flowers visited: Rosaceae: *Rubus canadensis* L., *Rubus* sp. (Table 2).

#### Biology.

Host: *Antrodiaetus unicolor* (Antrodiaetidae) (Schlinger 1987).

#### Comments.

*Eulonchus marialiciae* is the only disjunct species in the genus, with a relatively small distribution in the Great Smoky Mountains of North Carolina (USA); all other species are found in contiguous distributions in the far western part of the continent. This species is the sister species to the north-western *Eulonchus sapphirinus* and both have characteristic bright green metallic colouration, short proboscis, yellow legs and similarities in the male genitalia shape. *Eulonchus marialiciae* has the shortest proboscis of any species in the genus, as well as a much more elongated and laterally compressed flagellum.

### Eulonchus sapphirinus

Taxon classificationAnimaliaDipteraAcroceridae

Osten Sacken, 1877

Figs 8 , 9 , 16D , 17D , 18D , 19D

Eulonchus sapphirinus Osten Sacken, 1877: 276

#### References.

Osten Sacken 1878: 99 (catalogue); Melander 1902: 181 (California); Aldrich 1905: 221 (catalogue); Kertész 1909: 12 (catalogue); Woodworth 1913: 152 (California); Cole 1919: 36 (key, notes, figs, California, Utah, Oregon), 1927: 422 (male genitalia); Cole and Lovett 1921: 238 (Oregon); Brunetti 1926: 582 (Washington); Essig 1926: 559 (notes, California and Oregon); Knowlton et al. 1939: 6 (Utah); Sabrosky 1948: 389 (key, notes); Schlinger 1965: 404 (catalogue); Cole 1969: 221 (notes); Poole 1996: 36 (checklist).

#### Common name.

Northern Sapphire or Emerald.

#### Diagnosis.

Antennal flagellum relatively short, cylindrical or tapered; proboscis straight, shorter than length of body; ocellar tubercle trifurcate; legs yellow; calypter margin pale; body colour metallic green, blue or purple.

#### Redescription.

Body length: 8.3–11.9 mm, Wing length: 7.1–12.0 mm. *Head*. Flagellum red-brown or dark brown, male flagellum cylindrical, shorter than head height; scape and pedicel brown; clypeus elongate, extending beyond oral cavity, rounded with flat area dorsally, surface glossy, glabrous, black-brown; labial palp brown, length extending anteriorly beyond proboscis at point of attachment; margin of oral cavity (parafacial) pilose, proboscis length extending to middle of abdomen or equal to abdomen length; ocellar tubercle trifurcate, processes relatively short, subequal (posteromedial processes rounded), height equal to width; median ocellus present, greatly reduced or absent; occiput metallic green-blue, blue or purple, pile densely white or yellow. *Thorax*. Metallic green, blue or purple, setal pile white or yellow; coxae brown or black with metallic blue (and purple) sheen; femora yellow; tibiae dark yellow; tarsi dark yellow (distal tarsomeres often darker); calypter margin yellow to light brown, membrane transparent or translucent; haltere entirely light brown to yellow. *Abdomen*. Colour highly variable, metallic olive green, bright green or blue violet, vestiture white or yellow, dominant setae appressed or erect, pile posteriorly directed, marginal band of dense thicker setae on T3–4, or laterally directed pile on T2–4. *Male genitalia* (Figs 17D, 18D, 19D). Epandrium ovate, thinned at the apex, with posterior margin slightly concave; gonocoxite taller than wide, with broad fenestrae; aedeagus broad at the apex, bilobate in posterior view, not heavily sclerotized laterally.

#### Type material examined.

**Lectotype** male (designated here), MCZ, “*Webber Lake, Cal*/ *July 23. O Sacken*” [white]; “*West. Dipt.*/ *O. Sacken.*” [white]; “TYPE/ *4*/ *1076*” [red and white]; “MC-ENT/ 00303277” [white]; “LECTOTYPE ♂/ Eulonchus sapphirinus/ Osten Sacken/ Des. C.J. Borkent 2015” [red]; specimen condition: excellent, no parts missing. Body length: 9.2 mm, wing length: 8.7 mm. **Paralectotype** female, MCZ, “*Webber Lake, Cal.*/ *July 23. O. Sack.*” [white]; “O. Sacken./ *West. Dipt.*” [white]; “Type/ *5*/ *1076*” [red and white]; “MCZ-ENT/ 00303278” [white]; “PARALECTOTYPE ♀/ *Eulonchus sapphirinus*/ Osten Sacken/ Det. C.J. Borkent 2015” [yellow]; specimen condition: excellent, no parts missing. **Paralectotype** male, MCZ, “*Webber Lake*,/ *Cal. July 26.*/ *O. Sacken*” [white]; “*O. Sacken.*/ *West. Dipt.*” [white]; “Type/ *3*/ *1076*” [red and white]; “MCZ-ENT/ 00303276” [white]; “PARALECTOTYPE ♂/ *Eulonchus sapphirinus*/ Osten Sacken/ Det. C.J. Borkent 2015” [yellow]; specimen condition: very good, tarsi of both mid legs missing, genetic anomaly with only one antenna present. **Paralectotype** male, **paralectotype** female [mating pair on same pin], MCZ, “*Webber Lake, Cal./ July 26 O. Sacken*” [white]; “*O. Sacken.*/ *West. Dipt.*” [white]; “Aug-Dec 2006/ MCZ Image/ Database” [white]; “*Eulonchus*/ *sapphirinus*/ *O.S.*” [white]; “MCZ-ENT/ 00001076” [white]; “PARALECTOTYPE ♂/ Eulonchus sapphirinus/ Osten Sacken/ Det. C.J. Borkent 2015” [yellow]; specimen condition: male, very good, tarsi of hind legs missing, left flagellum missing; female, excellent, no parts missing.

#### Other material examined.

Listed in Table 3 (Suppl. material 1).

#### Distribution

(Fig. 20). Nearctic: California, Idaho, Nevada, Oregon, Utah, Washington (USA); British Columbia (Canada).

#### Ecology.

*Eulonchus sapphirinus* has been recorded visiting the flowers of 19 different plant families and 30 different species (Table 2). *Eulonchus sapphirinus* adults have been observed exhibiting strong fidelity to a single flowering plant species, suggesting their role as important pollinators (Borkent and Schlinger 2008a).

#### Biology.

Host unknown.

#### Comments.

*Eulonchus sapphirinus* is the sister species to the eastern *Eulonchus marialiciae* as both have characteristic bright green metallic colouration, short proboscis, yellow legs and similar shaped male genitalia. The shape of the antennal flagellum and colour of calypter separate the two species.

### Eulonchus smaragdinus

Taxon classificationAnimaliaDipteraAcroceridae

Gerstaecker, 1856

Figs 10 , 11 , 12 , 16F , 17E , 18E , 19E

Eulonchus smaragdinus Gerstaecker, 1856: 360.
Eulonchus smaragdinus pilosus Schlinger, 1960: 418, **syn. n.**

#### References.

Osten Sacken 1877: 276 (California, notes), 1878: 99 (catalogue); Melander 1902: 181 (California); Aldrich 1905: 221 (catalogue); Kertész 1909: 12 (catalogue); Verrall 1909a: 451 (fig wing); Cole 1919: 34 (key, notes, figs, California); Essig 1926: 559 (descr. note, California); Brunetti 1926: 583 (Uruguay [misidentification]); Sabrosky 1948: 388 (key ref., notes); Schlinger 1953: 220 (LT designation), 1960: 417 (description, distr., figs), 1965: 404 (catalogue), 1987: 320 (host *Aptostichus standfordianus*); Paramonov 1955: 20 (comparison with *Apsona muscaria*); Cole 1969: 221 (notes); Poole 1996: 36 (checklist).

#### Common name.

Southern Emerald or Sapphire.

#### Diagnosis.

Proboscis curved and longer than abdomen apex (as long or longer than wing length); ocellar tubercle nearly flat, weakly bifurcated; legs bright yellow; body colour metallic green, blue or purple; thorax covered in yellowish pile.

#### Redescription.

Body length: 8.3–12.9 mm, Wing length: 6.9–12.6 mm. *Head*. Flagellum red-brown or dark brown, male flagellum cylindrical, shorter than head height; scape and pedicel brown; clypeus elongate, length equal to oral cavity; rounded with flat area dorsally, black-brown, surface glossy, glabrous; labial palp brown or yellow, extending anteriorly beyond proboscis at point of attachment; margin of oral cavity (parafacial) glabrous, admixed with pubescence; proboscis length extending beyond abdomen; ocellar tubercle bifurcate (processes short and rounded), tubercle height shorter than width; median ocellus present or greatly reduced; occiput metallic green-blue or blue, pile densely white or yellow. *Thorax*. Metallic green, blue or purple, pile white or yellow; coxae brown or black with metallic blue (and green) sheen; femora yellow; tibiae dark yellow; tarsi dark yellow (distal tarsomeres often darker); calypter margin yellow or light brown; calypter membrane transparent; haltere entirely light brown to yellow. *Abdomen*. Metallic green or blue-violet, vestiture white or yellow, dominant setae appressed or erect, pile posteriorly directed, marginal band of laterally directed pile on T2–4. *Male genitalia* (Figs 17A, 18A, 19A). Epandrium rectangular, wide at the apex, with posterior margin slightly concave; gonocoxite deeply emarginate along anterior margin, fenestrae lacking; aedeagus thinned at the apex, only slightly sclerotized.

#### Type material examined.

**Lectotype** male, ZMB, “*Californien*/ *von Müller*” [green]; “1251” [white]; “Type” [orange]; “*smaragdinus*/ Gerst.*” [green]; “*Californ. v. Müller*” [green]; “*LECTOTYPE*/ *Eulonchus*/ *smaragdinus*/ *Gerst.*/ *Designation of. E.I. Schlinger-1952*” [blue]; specimen condition: very good, tarsi of both mid legs missing. Body length: 10.0 mm, wing length: 8.6 mm. **Paralectotype** female, ZMB, “*Californien*/ *von Müller S.*” [green]; “Type” [orange]; “PARALECTOTYPE ♀/ Eulonchus smaragdinus/ Gerstaecker/ Det. C.J. Borkent 2015” [yellow]; specimen condition: fair, head crushed, antennae broken off, tarsi of left mid leg and hind right leg missing.

**Figure 23.**
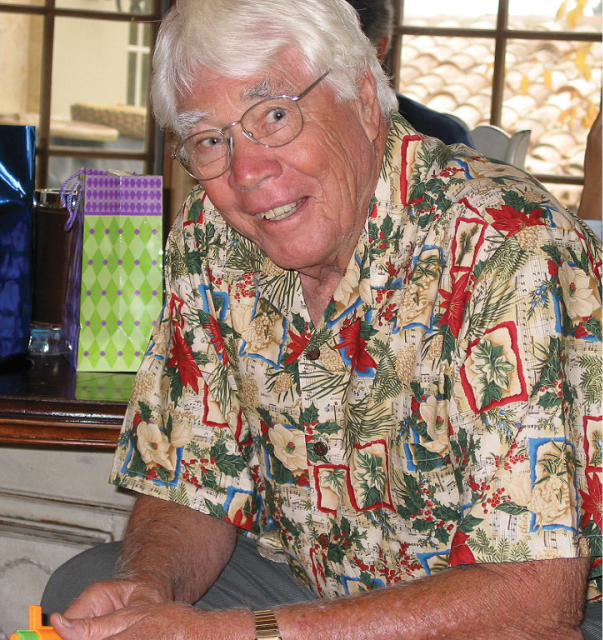
Evert I. Schlinger (1928–2014) was a world renowned expert on spider fly taxonomy and biology. This paper is dedicated to him and his legacy.

*Eulonchus smaragdinus pilosus* Schlinger, 1960: 418; **Holotype** male, USNM, “S Bernadino/ Co. CAL.”[white]; “Coquillet/ Collector” [white]; “Insect Book./ Pl.*18* fig *23*” [white]; “HOLOTYPE/ *Eulonchus*/ *smaragdinus*/ *pilosus*/ ♂ Schlinger” [orange]; specimen condition: excellent, tarsi of left hind leg missing. Body length: 10.1 mm, Wing length: 9.0 mm.

#### Other material examined.

Listed in Table 3 (Suppl. material 1).

#### Distribution

(Fig. 20). Nearctic: northern California (USA) to Baja California (Mexico). Erroneous record of Uruguay, see discussion by Schlinger 1960.

#### Ecology.

*Eulonchus smaragdinus* has been recorded visiting the flowers of 11 different plant families and 18 different species (Table 2, Borkent and Schlinger 2008b).

#### Biology.

Host: *Aptostichus standfordianus* (Euctenizidae) (Schlinger 1987).

#### Comments.

*Eulonchus smaragdinus* is highly variable in size and colour, and is superficially morphologically similar to *Eulonchus sapphirinus*, most notably in the bright yellow legs. However, it can be easily distinguished from the latter in having a proboscis that is curved (rather than straight) that extends beyond the abdomen, and is often longer than body. Male genitalic characters otherwise indicate a closer relationship to *Eulonchus halli*, as suggested by Schlinger (1960) (see Fig. 22). Schlinger (1960) erected the subspecies *Eulonchus smaragdinus pilosus* due to the lighter coloured pile of the individuals he collected. In our study we found that these lighter individuals were just one end of the colouration spectrum (golden pile changing progressively to white pile when moving north to south) of *Eulonchus smaragdinus*, and therefore do not recognize it as a distinct subspecies.

### Eulonchus tristis

Taxon classificationAnimaliaDipteraAcroceridae

Loew, 1872

Figs 13 , 14 , 15 , 16C , 17F , 18F , 19F

Eulonchus tristis Loew, 1872: 60.

#### References.

Osten Sacken 1877: 276 (California), 1878: 99 (catalogue); Melander 1902: 181 (California, Idaho, notes); Howard 1902: pl. 18, fig. 23 (habitus); Aldrich 1905: 221 (catalogue); Kertész 1909: 12 (catalogue); Woodworth 1913: 152 (California); Peterson 1916: 181, Figs 284a, 364a, 425a, 425b and 543 (head capsule, mouthparts); Cole 1919: 34, pl. 5, fig. 18 (habitus) (key, notes, Idaho, British Columbia, Oregon, California), 1927: 422, fig. 86 (male genitalia); Cole and Lovett 1921: 239 (Oregon); Essig 1926: 559 (descr. note, Idaho, Oregon, California); Brunetti 1926: 583 (Idaho); Sabrosky 1948: 390 (key ref., notes); Schlinger 1965: 404 (catalogue), 1969: 221, fig. 134 (notes), 1987: 320 (host); Poole 1996: 36 (checklist); Coyle and Icenogle 1994 (larval host).

#### Common name.

Dusky Sapphire.

#### Diagnosis.

Proboscis reaching apex of abdomen; ocellar tubercle trifurcate with three ocelli present (median smaller than laterals); legs mostly dark brown (‘knee’ pale), calypter margin dark and membrane white or light yellow.

#### Redescription.

Body length: 7.9–12.8 mm, Wing length: 6.0–11.2 mm. *Head*. Flagellum dark brown, scape and pedicel brown, male flagellum cylindrical, shorter than head height; clypeus elongate, extending beyond oral cavity, rounded with flat area dorsally, black-brown, surface glossy, glabrous; labial palp brown, length extending anteriorly beyond proboscis at point of attachment; margin of oral cavity (parafacial) pilose admixed with pubescence (faint); proboscis length from middle of abdomen or extending beyond abdomen; ocellar tubercle trifurcate, processes subequal (narrowly digitate), height equal to or shorter than width; median ocellus present, greatly reduced or absent; occiput metallic green-blue, blue or purple, pile densely white or yellow. *Thorax*. Metallic green, blue or purple, setal pile white or yellow; coxae brown or black with metallic blue sheen; femora dark brown, apicies white; tibiae dark yellow or brown; tarsi dark yellow; calypter margin dark brown or light brown, membrane translucent; haltere entirely dark brown. *Abdomen*. Metallic olive green, bright green to blue-violet, vestiture white or yellow, dominant setae appressed or erect, pile posteriorly directed, marginal band of dense thicker setae on T3-4, or posteriorly directed, marginal band of laterally directed pile on T2-4. *Male genitalia*. Epandrium round, with posterior margin almost straight; gonocoxite as tall as wide, with anterior margin almost straight, with large fenestrae; aedeagus heavily sclerotized laterally.

#### Type material examined.

**Lectotype** male (designated here), MCZ, “*S. Francisco*/ *H. Edw*” [white]; “Loew” [white]; “Type/ *3*/ *1077*” [red and white]; “MCZ-ENT/ 00303280” [white]; “LECTOTYPE ♂/ Eulonchus tristis/ Loew/ Des. C.J. Borkent 2015” [red]; specimen condition: tarsi of right mid leg and left flagellum missing, membrane of both wings slightly damaged on posterior portion. Body length: 9.8 mm, wing length: 9.2 mm. **Paralectotype** female, MCZ, “*S. Francisco*/ *H. Edw.*” [white]; “Loew” [white]; “Type/ *2*/ *1077*” [red and white]; “MCZ-ENT/ 00303279” [white]; “PARALECTOTYPE ♀/ Eulonchus tristis/ Loew/ Det. C.J. Borkent 2015” [yellow]; specimen condition: very good, tarsi of right hind leg missing. **Paralectotype female**, MCZ, “*S. Francisco*/ *H. Edw*” [white]; “Loew” [white]; “Type/ *4*/ *1077*” [red and white]; “MCZ-ENT/ 00303281” [white]; “PARALECTOTYPE ♀/ Eulonchus tristis/ Loew/ Det. C.J. Borkent 2015” [yellow]; specimen condition: very good, left mid and hind legs missing. **Paralectotype male**, MCZ, “*Californ*/ *Edwards*” [white]; “Loew” [white]; “*Eulonchus*/ *tristis Lw.*/ *Cant. X*” [white]; “Type/ *1077*” [red and white]; “MCZ-ENT/ 00001077” [red and white]; “Aug-Dec 2006/ MCZ Image/ Database” [white]; “PARALECTOTYPE ♂/ Eulonchus tristis/ Loew/ Det. C.J. Borkent 2015” [yellow]; specimen condition: very good, flagella missing.

#### Other material examined.

Listed in Table 3 (Suppl. material 1).

#### Distribution

(Fig. 20). Nearctic: Northern California, Oregon, Washington, Arizona.

#### Ecology.

Pollen loads and diversity from individual *Eulonchus tristis* visiting flowers of *Brodiaea elegans* (*Themidaceae*) and *Iris douglasiana* (Iridaceae) in California has been studied, showing high levels of constancy to a single species (Borkent and Schlinger 2008b). *Eulonchus tristis* has been recorded visiting the flowers of 19 different plant families and 38 different species (Table 2).

#### Biology.

Host: *Aliatypus* sp. (Antrodiaetidae) (Schlinger 1987).

#### Comments.

*Eulonchus tristis* is most similar to *Eulonchus marginatus*, with which it shares the ocellar tubercle trifid and the flagellum half as long as the head. *Eulonchus tristis* can be easily distinguished from *Eulonchus marginatus* by its femur and tibia with yellow markings and the pile on abdomen yellow.

## Supplementary Material

XML Treatment for Eulonchus

XML Treatment for Eulonchus halli

XML Treatment for Eulonchus marginatus

XML Treatment for Eulonchus marialiciae

XML Treatment for Eulonchus sapphirinus

XML Treatment for Eulonchus smaragdinus

XML Treatment for Eulonchus tristis

## Supplementary materials

Supplementary material 1 Table 3 Data type: material examined Explanation note: Non-type material examined (EIS # = Evert I. Schlinger collection database specimen accession number). See Materials and methods section for notes on depositories. This dataset is made available under the Open Database License (http://opendatacommons.org/licenses/odbl/1.0/). The Open Database License (ODbL) is a license agreement intended to allow users to freely share, modify, and use this Dataset while maintaining this same freedom for others, provided that the original source and author(s) are credited.Christopher J. Borkent, Jessica P. Gillung, Shaun L. Winterton
