# Supplementary material for: Jewelled spider flies of North America: a revision and phylogeny of Eulonchus Gerstaecker (Diptera, Acroceridae)
Source: Zookeys. 2016 Sep 27;(619):103–46. doi: 10.3897/zookeys.619.8249 (PMC5090163; doi:10.3897/zookeys.619.8249)
Supplement: Supplementary material 1 — Table 3 [file zookeys-619-103-s001.docx]

**Table 2**. Non-type material examined (EIS # = Evert I. Schlinger collection database specimen accession number). See Materials and Methods section for notes on depositories.

| **Species** | **EIS #** | **Gender** | **Depositories** | **Locality Data** |
| --- | --- | --- | --- | --- |
| *Eulonchus halli* | 000196 | male | CAS | USA, California, 8.1 km southwest Riverside, [33.908, -117.43], 9.IV.1954, J. C. Hall |
| *Eulonchus halli* | 000197 | male | CAS | USA, California, 8.1 km southwest Riverside, [33.908, -117.43], 9.IV.1954, J. C. Hall |
| *Eulonchus halli* | 000198 | male | CAS | USA, California, 8.1 km southwest Riverside, [33.908, -117.43], 4.IV.1954, D. W. Ricker |
| *Eulonchus halli* | 000199 | male | CAS | USA, California, Riverside, [33.948, -117.396], 27.III.1957, E. I. Schlinger |
| *Eulonchus halli* | 000200 | male | CAS | USA, California, Riverside, [33.948, -117.396], 26.III.1956, J. C. Hall |
| *Eulonchus halli* | 000201 | male | CAS | USA, California, 8.1 km southwest Riverside, [33.908, -117.43], 4.IV.1954, D. W. Ricker |
| *Eulonchus halli* | 000202 | male | CAS | USA, California, Riverside, [33.948, -117.396], 4.IV.1954, J. C. Hall |
| *Eulonchus halli* | 000203 | male | CAS | USA, California, Riverside, [33.948, -117.396], 4.IV.1954, J. C. Hall |
| *Eulonchus halli* | 000204 | male | CAS | USA, California, Riverside County, 12.9 km east Sunnymead, [33.947, -117.142], 26.III.1958, H. R. Moffitt |
| *Eulonchus halli* | 000205 | male | CAS | USA, California, Riverside, [33.948, -117.396], 8.III.1958, R. van den Bosch |
| *Eulonchus halli* | 000206 | male | CAS | USA, California, Riverside, [33.948, -117.396], 8.III.1958, R. van den Bosch |
| *Eulonchus halli* | 000207 | male | CAS | USA, California, Riverside, [33.948, -117.396], 24.III.1957, E. I. Schlinger |
| *Eulonchus halli* | 000208 | male | CAS | USA, California, Riverside, [33.948, -117.396], 8.III.1958, R. van den Bosch |
| *Eulonchus halli* | 000209 | male | CAS | USA, California, Riverside, [33.948, -117.396], 8.III.1958, E. I. Schlinger |
| *Eulonchus halli* | 000210 | male | CAS | USA, California, Riverside, [33.948, -117.396], 8.III.1958, E. I. Schlinger |
| *Eulonchus halli* | 000211 | male | CAS | USA, California, Riverside, [33.948, -117.396], 8.III.1958, E. I. Schlinger |
| *Eulonchus halli* | 000212 | male | CAS | USA, California, Riverside, [33.948, -117.396], 8.III.1958, E. I. Schlinger |
| *Eulonchus halli* | 000213 | male | CAS | USA, California, Riverside County, 12.9 km east Sunnymead, [33.947, -117.142], 12.IV.1958, E. I. Schlinger |
| *Eulonchus halli* | 000214 | male | CAS | USA, California, Riverside, [33.948, -117.396], 8.III.1958, J. C. Hall |
| *Eulonchus halli* | 000215 | male | CAS | USA, California, Riverside, [33.948, -117.396], 24.III.1957, E. I. Schlinger |
| *Eulonchus halli* | 000216 | male | CAS | USA, California, Riverside, [33.948, -117.396], 24.III.1957, E. I. Schlinger |
| *Eulonchus halli* | 000217 | male | CAS | USA, California, Riverside, [33.948, -117.396], 24.III.1957, E. I. Schlinger |
| *Eulonchus halli* | 000218 | male | CAS | USA, California, 8.1 km southwest Riverside, [33.908, -117.43], 4.IV.1954, D. W. Ricker |
| *Eulonchus halli* | 003122 | male | CAS | USA, California, Riverside County, 12.9 km east Sunnymead, [33.947, -117.142], 12.IV.1958, E. I. Schlinger |
| *Eulonchus halli* | 003123 | female | CAS | USA, California, Riverside County, 12.9 km east Sunnymead, [33.947, -117.142], 12.IV.1958, E. I. Schlinger |
| *Eulonchus halli* | 004397 | male | CAS | MEXICO, Baja California, Valle de la Trinidad, [31.787, -116.573], 16.III.1936, C. F. Harbison |
| *Eulonchus halli* | 004398 | male | CAS | MEXICO, Baja California Norte, Mesa west of Cantillas Canyon, [32.233, -115.9], 18.IX.1967, P. A. Opler |
| *Eulonchus halli* | 004399 | male | CAS | USA, California, Riverside County, 12.9 km east Sunnymead, [33.947, -117.142], 12.IV.1958, E. I. Schlinger |
| *Eulonchus halli* | 004400 | male | CAS | USA, California, Riverside County, 12.9 km east Sunnymead, [33.947, -117.142], 12.IV.1958, E. I. Schlinger |
| *Eulonchus halli* | 004401 | male | CAS | USA, California, Riverside County, 12.9 km east Sunnymead, [33.947, -117.142], 12.IV.1958, E. I. Schlinger |
| *Eulonchus halli* | 004402 | male | CAS | USA, California, Riverside County, 12.9 km east Sunnymead, [33.947, -117.142], 12.IV.1958, E. I. Schlinger |
| *Eulonchus halli* | 004403 | male | CAS | USA, California, Riverside County, 12.9 km east Sunnymead, [33.947, -117.142], 12.IV.1958, E. I. Schlinger |
| *Eulonchus halli* | 004404 | male | CAS | USA, California, Riverside County, 12.9 km east Sunnymead, [33.947, -117.142], 12.IV.1958, E. I. Schlinger |
| *Eulonchus halli* | 004405 | male | CAS | USA, California, Riverside County, 12.9 km east Sunnymead, [33.947, -117.142], 22.IV.1958, J. C. Hall |
| *Eulonchus halli* | 004406 | male | CAS | USA, California, Riverside County, 12.9 km east Sunnymead, [33.947, -117.142], 22.IV.1958, J. C. Hall |
| *Eulonchus halli* | 004407 | male | CAS | USA, California, Riverside County, 12.9 km east Sunnymead, [33.947, -117.142], 22.IV.1958, J. C. Hall |
| *Eulonchus halli* | 004408 | male | CAS | USA, California, Riverside County, 12.9 km east Sunnymead, [33.947, -117.142], 27.III.1959, J. C. Hall |
| *Eulonchus halli* | 004409 | male | CAS | USA, California, Riverside County, 12.9 km east Sunnymead, [33.947, -117.142], 27.III.1959, J. C. Hall |
| *Eulonchus halli* | 004410 | male | CAS | USA, California, Riverside County, 12.9 km east Sunnymead, [33.947, -117.142], 12.IV.1958, J. C. Hall |
| *Eulonchus halli* | 004411 | male | CAS | USA, California, Riverside County, 12.9 km east Sunnymead, [33.947, -117.142], 28.III.1959, E. I. Schlinger |
| *Eulonchus halli* | 004412 | male | CAS | USA, California, Riverside County, 12.9 km east Sunnymead, [33.947, -117.142], 26.III.1958, H. R. Moffitt |
| *Eulonchus halli* | 004413 | male | CAS | USA, California, Riverside County, 12.9 km east Sunnymead, [33.947, -117.142], 30.III.1958, H. R. Moffitt |
| *Eulonchus halli* | 004414 | male | CAS | USA, California, Riverside County, 12.9 km east Sunnymead, [33.947, -117.142], 28.III.1959, E. I. Schlinger |
| *Eulonchus halli* | 004415 | female | CAS | USA, California, 8.1 km southwest Riverside, [33.908, -117.43], 9.IV.1954, J. C. Hall |
| *Eulonchus halli* | 004416 | female | CAS | USA, California, 8.1 km southwest Riverside, [33.908, -117.43], 9.IV.1954, J. C. Hall |
| *Eulonchus halli* | 004417 | female | CAS | USA, California, 8.1 km southwest Riverside, [33.908, -117.43], 4.IV.1954, D. W. Ricker |
| *Eulonchus halli* | 004418 | female | CAS | USA, California, Riverside County, 12.9 km east Sunnymead, [33.947, -117.142], 12.IV.1958, E. I. Schlinger |
| *Eulonchus halli* | 004419 | female | CAS | USA, California, Riverside County, 12.9 km east Sunnymead, [33.947, -117.142], 12.IV.1958, J. C. Hall |
| *Eulonchus halli* | 004420 | female | CAS | USA, California, Riverside, [33.948, -117.396], 9.IV.1958, E. I. Schlinger |
| *Eulonchus halli* | 004421 | female | CAS | USA, California, Riverside, [33.948, -117.396], 8.III.1958, R. van den Bosch |
| *Eulonchus halli* | 004422 | female | CAS | USA, California, Riverside, [33.948, -117.396], 9.IV.1958, E. I. Schlinger |
| *Eulonchus halli* | 004423 | female | CAS | USA, California, Riverside, [33.948, -117.396], 27.III.1957, E. I. Schlinger |
| *Eulonchus halli* | 004424 | female | CAS | USA, California, Riverside County, 12.9 km east Sunnymead, [33.947, -117.142], 26.III.1958, H. R. Moffitt |
| *Eulonchus halli* | 004425 | male | CAS | USA, California, Riverside County, 12.9 km east Sunnymead, [33.947, -117.142], 12.IV.1958, J. C. Hall |
| *Eulonchus halli* | 004426 | female | CAS | USA, California, Riverside County, 12.9 km east Sunnymead, [33.947, -117.142], 12.IV.1958, J. C. Hall |
| *Eulonchus halli* | 004427 | unknown | CAS | USA, California, Landers, [34.266, -116.393], 8.IV.1966, A. E. Michelbacher |
| *Eulonchus halli* | 004428 | unknown | CAS | USA, California, San Diego County, 3.2 km west Borrego Valley, [33.255, -116.409], 28.II.1969, J. Wilcox |
| *Eulonchus halli* | 008334 | male | CAS | USA, California, Riverside, [33.948, -117.396], 9.IV.1958, E. I. Schlinger |
| *Eulonchus halli* | 009199 | female | CAS | USA, California, Riverside County, Gavilan Hills, [33.804, -117.376], 20.III.1988, R. Rogers |
| *Eulonchus halli* | 018928 | unknown | CAS | USA, California, Humboldt County, Humboldt Redwoods State Park [Rockerfeller National Park], [40.366, -123.929], 19.IV.1986, Bourandas |
| *Eulonchus marginatus* | 000274 | male | CAS | USA, California, Napa County, Samuel Springs, [38.604, -122.311], 30.V.1953, E. I. Schlinger |
| *Eulonchus marginatus* | 000275 | male | CAS | USA, California, Napa County, Samuel Springs, [38.604, -122.311], 30.V.1953, E. I. Schlinger |
| *Eulonchus marginatus* | 000276 | male | CAS | USA, California, Napa County, Samuel Springs, [38.604, -122.311], 30.V.1953, R. C. Bechtel |
| *Eulonchus marginatus* | 000277 | male | CAS | USA, California, Napa County, Samuel Springs, [38.604, -122.311], 30.V.1953, E. I. Schlinger |
| *Eulonchus marginatus* | 000278 | male | CAS | USA, California, Napa County, Samuel Springs, [38.604, -122.311], 30.V.1953, R. C. Bechtel |
| *Eulonchus marginatus* | 000279 | male | CAS | USA, California, Napa County, Samuel Springs, [38.604, -122.311], 30.V.1953, E. I. Schlinger |
| *Eulonchus marginatus* | 000280 | male | CAS | USA, California, Napa County, Samuel Springs, [38.604, -122.311], 30.V.1953, R. C. Bechtel |
| *Eulonchus marginatus* | 000281 | male | CAS | USA, California, Napa County, Samuel Springs, [38.604, -122.311], 30.V.1953, R. C. Bechtel |
| *Eulonchus marginatus* | 000282 | male | CAS | USA, California, Solano County, Green Valley, [38.252, -122.163], 19.VI.1953, R. C. Bechtel |
| *Eulonchus marginatus* | 000283 | male | CAS | USA, California, Marin County, Mill Valley, =, [37.906, -122.545], 31.V.1954, F. X. Williams |
| *Eulonchus marginatus* | 000284 | male | CAS | USA, California, Napa County, Samuel Springs, [38.604, -122.311], 30.V.1953, R. C. Bechtel |
| *Eulonchus marginatus* | 000285 | male | CAS | USA, California, Solano County, Green Valley, [38.252, -122.163], 19.VI.1953, E. I. Schlinger |
| *Eulonchus marginatus* | 000286 | male | CAS | USA, California, Solano County, Green Valley, [38.252, -122.163], 19.VI.1953, R. C. Bechtel |
| *Eulonchus marginatus* | 000287 | male | CAS | USA, California, Solano County, Green Valley, [38.252, -122.163], 19.VI.1953, E. I. Schlinger |
| *Eulonchus marginatus* | 000288 | male | CAS | USA, California, Solano County, Green Valley, [38.252, -122.163], 19.VI.1953, R. C. Bechtel |
| *Eulonchus marginatus* | 000289 | male | CAS | USA, California, Solano County, Green Valley, [38.252, -122.163], 19.VI.1953, R. C. Bechtel |
| *Eulonchus marginatus* | 000290 | male | CAS | USA, California, Napa County, Samuel Springs, [38.604, -122.311], 30.V.1953, E. I. Schlinger |
| *Eulonchus marginatus* | 000291 | male | UCDC | USA, California, Napa County, Samuel Springs, [38.604, -122.311], 7.VI.1953, R. M. Bohart |
| *Eulonchus marginatus* | 000292 | male | CAS | USA, California, Napa County, Samuel Springs, [38.604, -122.311], 30.V.1953, E. I. Schlinger |
| *Eulonchus marginatus* | 000293 | male | CAS | USA, California, Napa County, Samuel Springs, [38.604, -122.311], 7.VI.1953, R. C. Bechtel |
| *Eulonchus marginatus* | 000294 | male | CAS | USA, California, Napa County, Samuel Springs, [38.604, -122.311], 30.V.1953, R. C. Bechtel |
| *Eulonchus marginatus* | 000295 | male | CAS | USA, California, Napa County, Samuel Springs, [38.604, -122.311], 7.VI.1953, R. C. Bechtel |
| *Eulonchus marginatus* | 000296 | male | CAS | USA, California, Napa County, Samuel Springs, [38.604, -122.311], 30.V.1953, R. C. Bechtel |
| *Eulonchus marginatus* | 000297 | male | CAS | USA, California, Lake County, Hopland Grade, [38.997, -122.949], 29.V.1958, S. M. Fidel |
| *Eulonchus marginatus* | 000298 | male | CAS | USA, California, Mount Diablo, [37.905, -121.949], 29.V.1951, E. J. Taylor |
| *Eulonchus marginatus* | 000300 | male | CAS | USA, California, Napa County, Mount St. Helena, [38.669, -122.633], 9.VI.1918, E. P. Van Duzee |
| *Eulonchus marginatus* | 000301 | male | CAS | USA, California, Napa County, mountains west of St. Helena, [38.49, -122.495], 23.V.1956, H. H. Keifer |
| *Eulonchus marginatus* | 000302 | male | CAS | USA, California, Santa Cruz County, Santa Cruz Mountains, [37.091, -121.844], 20.VI.1956, D. Ribble |
| *Eulonchus marginatus* | 000303 | male | CAS | USA, California, Santa Clara County, San Antonio Valley, [37.355, -121.92], 3.VI.1954, C. D. MacNeill |
| *Eulonchus marginatus* | 000304 | male | CAS | USA, California, Mount Diablo, [37.905, -121.949], 30.V.1951, E. I. Schlinger |
| *Eulonchus marginatus* | 000305 | male | CAS | USA, California, Marin County, Mill Valley, =, [37.906, -122.545], 28.V.1965, P. H. Arnaud, Jr. |
| *Eulonchus marginatus* | 000306 | male | CAS | USA, California, Contra Costa County, Mount Diablo State Park, Juniper Camp, [37.879, -121.913], 884 m, 27.VI.1964, D. C. Rents, K. A. Rents |
| *Eulonchus marginatus* | 000307 | male | CAS | USA, California, Yolo County, Putah Canyon, [38.513, -122.101], 2.VI.1962, M. E. Irwin |
| *Eulonchus marginatus* | 000308 | male | CAS | USA, California, Yolo County, Putah Canyon, [38.513, -122.101], 2.VI.1962, M. E. Irwin |
| *Eulonchus marginatus* | 000309 | male | CAS | USA, California, Marin County, Mill Valley, =, [37.906, -122.545], 14.V.1961, D. Q. Cavagnaro |
| *Eulonchus marginatus* | 000310 | male | CAS | USA, California, Lake County, Hopland Grade, [38.997, -122.949], 7.VI.1960, S. M. Fidel |
| *Eulonchus marginatus* | 000311 | male | CAS | USA, California, Santa Clara County, San Antonio Valley, [37.355, -121.92], 3.VI.1954, C. D. MacNeill |
| *Eulonchus marginatus* | 000312 | male | CAS | USA, California, Napa County, Samuel Springs, [38.604, -122.311], 30.V.1955, A. A.  Grigarick |
| *Eulonchus marginatus* | 000313 | male | CAS | USA, California, Napa County, Samuel Springs, [38.604, -122.311], 29.V.1955, R. C. Bechtel |
| *Eulonchus marginatus* | 000314 | male | CAS | USA, California, Santa Clara County, San Antonio Valley, [37.355, -121.92], 3.VI.1954, C. D. MacNeill |
| *Eulonchus marginatus* | 000315 | male | CAS | USA, California, Napa County, Samuel Springs, [38.604, -122.311], 30.V.1955, A. D. Telford |
| *Eulonchus marginatus* | 000316 | male | CAS | USA, California, Napa County, Samuel Springs, [38.604, -122.311], 30.V.1955, R. C. Bechtel |
| *Eulonchus marginatus* | 000317 | male | CAS | USA, California, Santa Cruz County, 14.5 km northeast Soquel, [36.988, -121.957], 16.VI.1956, S. M. Fidel |
| *Eulonchus marginatus* | 000318 | male | CAS | USA, California, Santa Cruz County, 14.5 km northeast Soquel, [36.988, -121.957], 16.VI.1956, S. M. Fidel |
| *Eulonchus marginatus* | 000319 | male | CAS | USA, California, Santa Cruz County, 14.5 km northeast Soquel, [36.988, -121.957], 16.VI.1956, S. M. Fidel |
| *Eulonchus marginatus* | 000320 | male | CAS | USA, California, Santa Cruz County, 14.5 km northeast Soquel, [36.988, -121.957], 16.VI.1956, S. M. Fidel |
| *Eulonchus marginatus* | 000321 | male | CAS | USA, California, Napa County, 22.5 km east Rutherford, near Hennesey Dam, [38.457, -122.169], 22.V.1955, J. C. Downey |
| *Eulonchus marginatus* | 000322 | male | CAS | USA, California, Napa County, Samuel Springs, [38.604, -122.311], 18.V.1955, E. I. Schlinger |
| *Eulonchus marginatus* | 000323 | male | CAS | USA, California, Santa Cruz County, 14.5 km northeast Soquel, [36.988, -121.957], 16.VI.1956, S. M. Fidel |
| *Eulonchus marginatus* | 000324 | male | CAS | USA, California, Napa County, Samuel Springs, [38.604, -122.311], 30.V.1955, R. C. Bechtel |
| *Eulonchus marginatus* | 000325 | male | UCDC | USA, California, Napa County, Samuel Springs, [38.604, -122.311], 18.V.1955, R. M. Bohart |
| *Eulonchus marginatus* | 000326 | male | CAS | USA, California, Mount Diablo, [37.905, -121.949], 29.V.1951, W. J. Wall |
| *Eulonchus marginatus* | 000327 | male | CAS | USA, California, Mount Diablo, [37.905, -121.949], 30.V.1951, E. I. Schlinger |
| *Eulonchus marginatus* | 000328 | male | CAS | USA, California, Mount Diablo, [37.905, -121.949], 29.V.1951, W. J. Wall |
| *Eulonchus marginatus* | 000329 | male | CAS | USA, California, Napa County, Samuel Springs, [38.604, -122.311], 18.V.1955, R. C. Bechtel |
| *Eulonchus marginatus* | 000330 | male | CAS | USA, California, Mount Diablo, [37.905, -121.949], 29.V.1951, W. J. Wall |
| *Eulonchus marginatus* | 000331 | male | CAS | USA, California, Mount Diablo, [37.905, -121.949], 30.V.1951, E. I. Schlinger |
| *Eulonchus marginatus* | 000332 | male | CAS | USA, California, Mount Diablo, [37.905, -121.949], 29.V.1951, E. J. Taylor |
| *Eulonchus marginatus* | 000333 | male | CAS | USA, California, Mount Diablo, [37.905, -121.949], 29.V.1951, W. J. Wall |
| *Eulonchus marginatus* | 000334 | male | CAS | USA, California, Mount Diablo, [37.905, -121.949], 30.V.1951, E. I. Schlinger |
| *Eulonchus marginatus* | 000335 | male | CAS | USA, California, Mount Diablo, [37.905, -121.949], 29.V.1951, W. J. Wall |
| *Eulonchus marginatus* | 000336 | male | CAS | USA, California, Solano County, Green Valley, [38.252, -122.163], 19.VI.1953, R. C. Bechtel |
| *Eulonchus marginatus* | 000337 | male | CAS | USA, California, Solano County, Green Valley, [38.252, -122.163], 19.VI.1953, R. C. Bechtel |
| *Eulonchus marginatus* | 000338 | male | CAS | USA, California, Santa Clara County, Stevens Creek Area, [37.431, -122.066], 28.V.1952, D. J. Burdick |
| *Eulonchus marginatus* | 000339 | male | CAS | USA, California, Santa Clara County, Palo Alto, [37.442, -122.143], 7.VI.1922, S. E. Flanders |
| *Eulonchus marginatus* | 000340 | male | CAS | USA, California, Santa Cruz County, Santa Cruz Mountains, [37.091, -121.844], 15.VI.1922 |
| *Eulonchus marginatus* | 000341 | male | CAS | USA, California, Mount Diablo, [37.905, -121.949], 29.V.1951, W. J. Wall |
| *Eulonchus marginatus* | 000342 | male | CAS | USA, California, Mount Diablo, [37.905, -121.949], 30.V.1951, E. I. Schlinger |
| *Eulonchus marginatus* | 000343 | male | CAS | USA, California, Mount Diablo, [37.905, -121.949], 29.V.1951, E. J. Taylor |
| *Eulonchus marginatus* | 000344 | male | CAS | USA, California, Lake County, Hopland Grade, [38.997, -122.949], 10.VI.1959, S. M. Fidel |
| *Eulonchus marginatus* | 000345 | male | CAS | USA, California, Lake County, Hopland Grade, [38.997, -122.949], 10.VI.1959, S. M. Fidel |
| *Eulonchus marginatus* | 000346 | male | CAS | USA, California, Lake County, Kelseyville, [38.978, -122.84], 4.VI.1958, S. M. Fidel |
| *Eulonchus marginatus* | 000347 | male | CAS | USA, California, Mount Diablo, [37.905, -121.949], 30.V.1951, E. I. Schlinger |
| *Eulonchus marginatus* | 000348 | male | CAS | USA, California, Sonoma County, Sobre Vista, [38.333, -122.511], 8.V.1910, J. A. Kusche |
| *Eulonchus marginatus* | 000349 | male | CAS | USA, California, Lake County, Hopland Grade, [38.997, -122.949], 10.VI.1959 |
| *Eulonchus marginatus* | 000350 | male | CAS | USA, California, Mount Tamalpais, [37.924, -122.596], .VI.1950, E. S. Ross |
| *Eulonchus marginatus* | 000351 | male | CAS | USA, California, Marin County, Mill Valley, =, [37.906, -122.545], 2.VI.1950, F. X. Williams |
| *Eulonchus marginatus* | 000352 | male | CAS | USA, California, Gilroy Hot Springs, [37.108, -121.478], 28.V.1925, L. S. Slevin |
| *Eulonchus marginatus* | 000353 | male | CAS | USA, California, Lake County, Hopland Grade, [38.997, -122.949], 10.VI.1959, S. M. Fidel |
| *Eulonchus marginatus* | 000354 | male | CAS | USA, California, Lake County, Hopland Grade, [38.997, -122.949], 6.V.1959, S. M. Fidel |
| *Eulonchus marginatus* | 000355 | male | CAS | USA, California, Mount Diablo, [37.905, -121.949], 30.V.1951, E. I. Schlinger |
| *Eulonchus marginatus* | 000356 | male | CAS | USA, California, Solano County, Vacaville, [38.357, -121.988], 14.VI.1947 |
| *Eulonchus marginatus* | 000357 | female | CAS | USA, California, Santa Cruz County, Mount Hermon, [37.051, -122.059], 21.IX.1947, P. Williams |
| *Eulonchus marginatus* | 000358 | female | CAS | USA, California, San Benito County, Idria (gem mine), [36.414, -120.675], 15.VI.1955, C. D. MacNeill |
| *Eulonchus marginatus* | 000359 | female | CAS | USA, California, Lake County, Hopland Grade, [38.997, -122.949], 10.VI.1959, S. M. Fidel |
| *Eulonchus marginatus* | 000360 | female | CAS | USA, California, Lake County, Hopland Grade, [38.997, -122.949], 29.V.1958, S. M. Fidel |
| *Eulonchus marginatus* | 000361 | female | UCDC | USA, California, Napa County, Samuel Springs, [38.604, -122.311], 7.VI.1953, R. M. Bohart |
| *Eulonchus marginatus* | 000362 | female | CAS | USA, California, Mount Diablo, [37.905, -121.949], 29.V.1951, W. J. Wall |
| *Eulonchus marginatus* | 000363 | female | CAS | USA, California, Lake County, Hopland Grade, [38.997, -122.949], 29.V.1958, S. M. Fidel |
| *Eulonchus marginatus* | 000364 | female | CAS | USA, California, Santa Clara County, San Antonio Valley, [37.355, -121.92], 3.VI.1954, C. D. MacNeill |
| *Eulonchus marginatus* | 000365 | female | CAS | USA, California, Napa County, Samuel Springs, [38.604, -122.311], 7.VI.1953, R. C. Bechtel |
| *Eulonchus marginatus* | 000366 | female | CAS | USA, California, Napa County, Samuel Springs, [38.604, -122.311], 29.V.1955, A. D. Telford |
| *Eulonchus marginatus* | 000367 | female | CAS | USA, California, Napa County, Samuel Springs, [38.604, -122.311], 30.V.1953, R. C. Bechtel |
| *Eulonchus marginatus* | 000368 | female | CAS | USA, California, Gilroy Hot Springs, [37.108, -121.478], 28.V.1925, L. S. Slevin |
| *Eulonchus marginatus* | 000369 | female | CAS | USA, California, Santa Cruz County, Santa Cruz Mountains, [37.091, -121.844], 1.VII.1955, Ribble |
| *Eulonchus marginatus* | 000370 | female | CAS | USA, California, Mount Diablo, [37.905, -121.949], 29.V.1951, E. J. Taylor |
| *Eulonchus marginatus* | 000371 | female | CAS | USA, California, Lake County, Hopland Grade, [38.997, -122.949], 7.VI.1960, S. M. Fidel |
| *Eulonchus marginatus* | 000372 | female | CAS | USA, California, Napa County, Samuel Springs, [38.604, -122.311], 7.VI.1953, R. C. Bechtel |
| *Eulonchus marginatus* | 000373 | female | CAS | USA, California, Mount Diablo, [37.905, -121.949], 30.V.1951, E. I. Schlinger |
| *Eulonchus marginatus* | 000374 | female | CAS | USA, California, Lake County, Hopland Grade, [38.997, -122.949], 10.VI.1959, S. M. Fidel |
| *Eulonchus marginatus* | 000375 | female | CAS | USA, California, Napa County, Samuel Springs, [38.604, -122.311], 28.V.1953, J. C. Hall |
| *Eulonchus marginatus* | 000376 | female | CAS | USA, California, Lake County, Hopland Grade, [38.997, -122.949], 10.VI.1959, S. M. Fidel |
| *Eulonchus marginatus* | 000377 | female | CAS | USA, California, Napa County, Samuel Springs, [38.604, -122.311], 7.VI.1953, R. C. Bechtel |
| *Eulonchus marginatus* | 000378 | female | CAS | USA, California, Napa County, Samuel Springs, [38.604, -122.311], 30.V.1955, R. C. Bechtel |
| *Eulonchus marginatus* | 000379 | male | CAS | USA, California, Mount Diablo, [37.905, -121.949], 30.V.1951, E. I. Schlinger |
| *Eulonchus marginatus* | 000380 | female | CAS | USA, California, Mount Diablo, [37.905, -121.949], 30.V.1951, E. I. Schlinger |
| *Eulonchus marginatus* | 000383 | male | CAS | USA, California, Napa County, Samuel Springs, [38.604, -122.311], 30.V.1955, A. A.  Grigarick |
| *Eulonchus marginatus* | 000384 | female | CAS | USA, California, Napa County, Samuel Springs, [38.604, -122.311], 30.V.1955, A. A.  Grigarick |
| *Eulonchus marginatus* | 000385 | male | CAS | USA, California, Napa County, Samuel Springs, [38.604, -122.311], 7.VI.1953, R. C. Bechtel |
| *Eulonchus marginatus* | 000386 | female | CAS | USA, California, Napa County, Samuel Springs, [38.604, -122.311], 7.VI.1953, R. C. Bechtel |
| *Eulonchus marginatus* | 000418 | male | CAS | USA, California, Lake County, Hopland Grade, [38.997, -122.949], 17.VI.1959, S. M. Fidel |
| *Eulonchus marginatus* | 000419 | male | CAS | USA, California, Lake County, Hopland Grade, [38.997, -122.949], 17.VI.1959, S. M. Fidel |
| *Eulonchus marginatus* | 002216 | male | CAS | USA, California, Alameda County, Redwood Park, [37.184, -122.166], 10.VI.1949, C. D. MacNeill |
| *Eulonchus marginatus* | 002217 | male | CAS | USA, California |
| *Eulonchus marginatus* | 002218 | male | UCDC | USA, California, Mount Diablo, [37.905, -121.949], 10.V.1941, G. E. Bohart |
| *Eulonchus marginatus* | 002219 | male | UCDC | USA, California, Mount Diablo, [37.905, -121.949], 10.V.1941, G. E. Bohart |
| *Eulonchus marginatus* | 002220 | male | UCDC | USA, California, Santa Clara County, Palo Alto, [37.442, -122.143], 7.VI.1936, G. E. Bohart |
| *Eulonchus marginatus* | 002221 | male | UCDC | USA, California, Mount Diablo, [37.905, -121.949], 10.V.1941, G. E. Bohart |
| *Eulonchus marginatus* | 002222 | male | UCDC | USA, California, Mount Diablo, [37.905, -121.949], 10.V.1941, G. E. Bohart |
| *Eulonchus marginatus* | 002223 | male | UCDC | USA, California, Mount Diablo, [37.905, -121.949], 10.V.1941, G. E. Bohart |
| *Eulonchus marginatus* | 002224 | male | UCDC | USA, California, Mount Diablo, [37.905, -121.949], 10.V.1941, G. E. Bohart |
| *Eulonchus marginatus* | 002225 | male | UCDC | USA, California, Mount Diablo, [37.905, -121.949], 10.V.1941, G. E. Bohart |
| *Eulonchus marginatus* | 002226 | male | UCDC | USA, California, Mount Diablo, [37.905, -121.949], 10.V.1941, G. E. Bohart |
| *Eulonchus marginatus* | 003124 | unknown | CAS | USA, California, Mill Valley, Ross Lane, [37.878, -122.53], .VI.1975, E. S. Ross |
| *Eulonchus marginatus* | 003125 | female | CAS | USA, California, Contra Costa County, Mount Diablo, [37.882, -121.914], 10.VI.1952, E. C. Van Dyke |
| *Eulonchus marginatus* | 003126 | female | CAS | USA, California, Mount Diablo, [37.882, -121.914], 610 m, 18.VI.1951, F. X. Williams |
| *Eulonchus marginatus* | 003127 | female | CAS | USA, California, Gilroy Hot Springs, [37.108, -121.478], 29.V.1925 |
| *Eulonchus marginatus* | 003128 | female | CAS | USA, California, Contra Costa County, 8.VI.1932, E. R. Leach |
| *Eulonchus marginatus* | 003129 | female | CAS | USA, California, Contra Costa County, 8.VI.1932, E. R. Leach |
| *Eulonchus marginatus* | 003130 | female | CAS | USA, California, Mount Diablo, [37.882, -121.914], 610 m, 26.VI.1951, F. X. Williams |
| *Eulonchus marginatus* | 003131 | female | CAS | USA, California, Mount Diablo, [37.905, -121.949], 10.VI.1951, J. R. Helfer |
| *Eulonchus marginatus* | 003132 | female | CAS | USA, California, Mount Diablo, [37.882, -121.914], 610 m, 14.VI.1949, F. X. Williams |
| *Eulonchus marginatus* | 003133 | female | CAS | USA, California, Mount Diablo, [37.882, -121.914], 610 m, 10.VI.1949, F. X. Williams |
| *Eulonchus marginatus* | 003134 | female | CAS | USA, California, Mount Diablo, [37.905, -121.949], 10.VI.1951, J. R. Helfer |
| *Eulonchus marginatus* | 003135 | female | CAS | USA, California, Mount Diablo, [37.882, -121.914], 610 m, 26.VI.1951, F. X. Williams |
| *Eulonchus marginatus* | 003136 | female | CAS | USA, California, Contra Costa County, Mount Diablo, [37.882, -121.914], 10.VI.1952, F. X. Williams |
| *Eulonchus marginatus* | 003137 | female | CAS | USA, California, Mount Diablo, [37.882, -121.914], 610 m, 18.VI.1951, F. X. Williams |
| *Eulonchus marginatus* | 003138 | female | CAS | USA, California, Mount Diablo, [37.882, -121.914], 610 m, 18.VI.1951, F. X. Williams |
| *Eulonchus marginatus* | 003139 | female | CAS | USA, California, Mount Diablo, [37.882, -121.914], 610 m, 18.VI.1951, F. X. Williams |
| *Eulonchus marginatus* | 003140 | female | CAS | USA, California, Mount Diablo, [37.882, -121.914], 610 m, 18.VI.1951, F. X. Williams |
| *Eulonchus marginatus* | 003141 | male | CAS | USA, California, Mount Diablo, [37.882, -121.914], 610 m, 18.VI.1951, F. X. Williams |
| *Eulonchus marginatus* | 003142 | male | CAS | USA, California, Mount Diablo, [37.882, -121.914], 610 m, 18.VI.1951, F. X. Williams |
| *Eulonchus marginatus* | 003143 | male | CAS | USA, California, Mount Diablo, [37.882, -121.914], 610 m, 18.VI.1951, F. X. Williams |
| *Eulonchus marginatus* | 003144 | male | CAS | USA, California, Mount Diablo, [37.882, -121.914], 610 m, 18.VI.1951, F. X. Williams |
| *Eulonchus marginatus* | 003145 | male | CAS | USA, California, Mount Diablo, [37.882, -121.914], 610 m, 18.VI.1951, F. X. Williams |
| *Eulonchus marginatus* | 003146 | male | CAS | USA, California, Mount Diablo, [37.882, -121.914], 610 m, 18.VI.1951, F. X. Williams |
| *Eulonchus marginatus* | 003147 | female | CAS | USA, California, San Mateo County, Woodside, [37.43, -122.254], 19.V.1951, P. H. Arnaud, Jr. |
| *Eulonchus marginatus* | 003148 | male | CAS | USA, California, Mount Diablo, [37.882, -121.914], 610 m, 18.VI.1951, F. X. Williams |
| *Eulonchus marginatus* | 003149 | male | CAS | USA, California, Mount Diablo, [37.882, -121.914], 610 m, 18.VI.1951, F. X. Williams |
| *Eulonchus marginatus* | 003150 | male | CAS | USA, California, Mount Diablo, [37.882, -121.914], 610 m, 18.VI.1951, F. X. Williams |
| *Eulonchus marginatus* | 003151 | male | CAS | USA, California, Mount Diablo, [37.882, -121.914], 610 m, 18.VI.1951, F. X. Williams |
| *Eulonchus marginatus* | 003152 | male | CAS | USA, California, Mount Diablo, [37.882, -121.914], 610 m, 18.VI.1951, F. X. Williams |
| *Eulonchus marginatus* | 003153 | male | CAS | USA, California, Mount Diablo, [37.882, -121.914], 610 m, 18.VI.1951, F. X. Williams |
| *Eulonchus marginatus* | 003154 | male | CAS | USA, California, Mount Diablo, [37.882, -121.914], 610 m, 18.VI.1951, F. X. Williams |
| *Eulonchus marginatus* | 003155 | male | CAS | USA, California, Marin County, Pine Mountain, 5.6 km west Fairfax, 1.6 km N Alpine Lake, Truck Road, [37.95, -122.693], 366 m, 18.V.1968, T. W. Davies |
| *Eulonchus marginatus* | 003156 | female | CAS | USA, California, Mount Diablo, [37.905, -121.949], 18.V.1951, J. R. Helfer |
| *Eulonchus marginatus* | 003157 | male | CAS | USA, California, Contra Costa County, Mount Diablo, [37.882, -121.914], 10.VI.1952, F. X. Williams |
| *Eulonchus marginatus* | 003158 | female | CAS | USA, California, Contra Costa County, Mount Diablo, [37.882, -121.914], 10.VI.1952, F. X. Williams |
| *Eulonchus marginatus* | 003159 | male | CAS | USA, California, Mount Diablo, [37.882, -121.914], 610 m, 26.VI.1951, F. X. Williams |
| *Eulonchus marginatus* | 003160 | male | CAS | USA, California, Contra Costa County, Mount Diablo, [37.882, -121.914], 19.VI.1952, F. X. Williams |
| *Eulonchus marginatus* | 003161 | male | CAS | USA, California, Mount Tamalpais, [37.924, -122.596], .VI.1950, E. S. Ross |
| *Eulonchus marginatus* | 003162 | male | CAS | USA, California, Mount Diablo, [37.905, -121.949], 10.VI.1951, J. R. Helfer |
| *Eulonchus marginatus* | 003163 | male | CAS | USA, California, Mount Diablo, [37.905, -121.949], 10.VI.1951, J. R. Helfer |
| *Eulonchus marginatus* | 003164 | male | CAS | USA, California, Marin County, Mill Valley, =, [37.906, -122.545], 28.V.1965, P. H. Arnaud, Jr. |
| *Eulonchus marginatus* | 003165 | male | CAS | USA, California, Contra Costa County, Mount Diablo State Park, Juniper Camp, [37.879, -121.913], 884 m, 27.VI.1964, D. C. Rents, K. A. Rents |
| *Eulonchus marginatus* | 003166 | male | CAS | USA, California, Contra Costa County, Mount Diablo State Park, Juniper Camp, [37.879, -121.913], 884 m, 27.VI.1964, D. C. Rents, K. A. Rents |
| *Eulonchus marginatus* | 003167 | male | CAS | USA, California, Marin County, Mill Valley, =, [37.906, -122.545], 28.V.1965, P. H. Arnaud, Jr. |
| *Eulonchus marginatus* | 003168 | male | CAS | USA, California, Marin County, Mill Valley, [37.906, -122.545], 110 m, 9.VII.1965 to 12.VII.1965, P. H. Arnaud, Jr. |
| *Eulonchus marginatus* | 003169 | male | CAS | USA, California, Marin County, Mill Valley, [37.906, -122.545], 110 m, 9.VII.1965 to 12.VII.1965, P. H. Arnaud, Jr. |
| *Eulonchus marginatus* | 003170 | male | CAS | USA, California, Sonoma County, [38.292, -122.458], 23.IV.1928, E. R. Leach |
| *Eulonchus marginatus* | 003171 | female | CAS | USA, California, Napa County, near Pope Valley, Pope Creek, [38.645, -122.368], 12.V.1968, J. D. Birchim |
| *Eulonchus marginatus* | 003172 | unknown | CAS | USA, California, Sonoma County, [38.292, -122.458], 23.IV.1928, E. R. Leach |
| *Eulonchus marginatus* | 003173 | male | CAS | USA, California, Sonoma County, [38.292, -122.458] |
| *Eulonchus marginatus* | 003174 | male | CAS | USA, California, Sonoma County, Sobre Vista, [38.333, -122.511], 8.V.1910, J. A. Kusche |
| *Eulonchus marginatus* | 003175 | male | CAS | USA, California, Contra Costa County, 8.VI.1932, E. R. Leach |
| *Eulonchus marginatus* | 003176 | male | CAS | USA, California, Mount Diablo, [37.882, -121.914], 610 m, 21.V.1949, F. X. Williams |
| *Eulonchus marginatus* | 003177 | male | CAS | USA, California, Mount Diablo, [37.882, -121.914], 610 m, 10.VI.1949, F. X. Williams |
| *Eulonchus marginatus* | 003178 | male | CAS | USA, California, Contra Costa County, 8.VI.1932, E. R. Leach |
| *Eulonchus marginatus* | 003179 | male | CAS | USA, California, Contra Costa County, 8.VI.1932, E. R. Leach |
| *Eulonchus marginatus* | 003180 | male | CAS | USA, California, Mill Valley, [37.906, -122.545], 7.VI.1915, E. P. Van Duzee |
| *Eulonchus marginatus* | 003181 | male | CAS | USA, California, Tamalpais, [37.905, -122.604], 28.V.1922, C. L. Fox |
| *Eulonchus marginatus* | 008919 | unknown | CAS | USA, California, Monterey County, Arroyo Seco, [36.232, -121.485], 30.V.1972, P. Torchio |
| *Eulonchus marginatus* | 008944 | unknown | CAS | USA, California, Santa Clara County, Mount Hamilton, [37.342, -121.643], 29.V.1972, P. F. Torchio |
| *Eulonchus marginatus* | 008945 | unknown | CAS | USA, California, Santa Clara County, Mount Hamilton, [37.342, -121.643], 29.V.1972, P. F. Torchio |
| *Eulonchus marginatus* | 008951 | unknown | CAS | USA, California, Santa Clara County, Mount Hamilton, [37.342, -121.643], 29.V.1972, P. F. Torchio |
| *Eulonchus marginatus* | 008953 | unknown | CAS | USA, California, Santa Clara County, Mount Hamilton, [37.342, -121.643], 29.V.1972, P. F. Torchio |
| *Eulonchus marginatus* | 008954 | unknown | CAS | USA, California, Santa Clara County, Mount Hamilton, [37.342, -121.643], 29.V.1972, P. F. Torchio |
| *Eulonchus marginatus* | 008955 | unknown | CAS | USA, California, Santa Clara County, Mount Hamilton, [37.342, -121.643], 29.V.1972, P. F. Torchio |
| *Eulonchus marginatus* | 008956 | unknown | CAS | USA, California, Santa Clara County, Mount Hamilton, [37.342, -121.643], 29.V.1972, P. F. Torchio |
| *Eulonchus marginatus* | 008957 | unknown | CAS | USA, California, Santa Clara County, Mount Hamilton, [37.342, -121.643], 29.V.1972, P. F. Torchio |
| *Eulonchus marginatus* | 008958 | unknown | CAS | USA, California, Santa Clara County, Mount Hamilton, [37.342, -121.643], 29.V.1972, P. F. Torchio |
| *Eulonchus marginatus* | 008959 | unknown | CAS | USA, California, Santa Clara County, Mount Hamilton, [37.342, -121.643], 29.V.1972, P. F. Torchio |
| *Eulonchus marginatus* | 008960 | unknown | CAS | USA, California, Santa Clara County, Mount Hamilton, [37.342, -121.643], 29.V.1972, P. F. Torchio |
| *Eulonchus marginatus* | 008961 | unknown | CAS | USA, California, Santa Clara County, Mount Hamilton, [37.342, -121.643], 29.V.1972, P. F. Torchio |
| *Eulonchus marginatus* | 008962 | unknown | CAS | USA, California, Santa Clara County, Mount Hamilton, [37.342, -121.643], 29.V.1972, P. F. Torchio |
| *Eulonchus marginatus* | 008963 | unknown | CAS | USA, California, Santa Clara County, Mount Hamilton, [37.342, -121.643], 29.V.1972, P. F. Torchio |
| *Eulonchus marginatus* | 008964 | unknown | CAS | USA, California, Santa Clara County, Mount Hamilton, [37.342, -121.643], 29.V.1972, P. F. Torchio |
| *Eulonchus marginatus* | 008965 | unknown | CAS | USA, California, Santa Clara County, Mount Hamilton, [37.342, -121.643], 29.V.1972, P. F. Torchio |
| *Eulonchus marginatus* | 008966 | unknown | CAS | USA, California, Santa Clara County, Mount Hamilton, [37.342, -121.643], 29.V.1972, P. F. Torchio |
| *Eulonchus marginatus* | 008967 | unknown | CAS | USA, California, Santa Clara County, Mount Hamilton, [37.342, -121.643], 29.V.1972, P. F. Torchio |
| *Eulonchus marginatus* | 008968 | unknown | CAS | USA, California, Santa Clara County, Mount Hamilton, [37.342, -121.643], 29.V.1972, P. F. Torchio |
| *Eulonchus marginatus* | 008969 | unknown | CAS | USA, California, Santa Clara County, Mount Hamilton, [37.342, -121.643], 29.V.1972, P. F. Torchio |
| *Eulonchus marginatus* | 008970 | unknown | CAS | USA, California, Santa Clara County, Mount Hamilton, [37.342, -121.643], 29.V.1972, P. F. Torchio |
| *Eulonchus marginatus* | 008974 | unknown | CAS | USA, California, Mt. Diablo, [37.882, -121.914], 7.V.1939 |
| *Eulonchus marginatus* | 008977 | unknown | CAS | USA, California, Santa Clara County, Mount Hamilton, [37.342, -121.643], 29.V.1972, P. F. Torchio |
| *Eulonchus marginatus* | 008978 | unknown | CAS | USA, California, Santa Clara County, Mount Hamilton, [37.342, -121.643], 29.V.1972, P. F. Torchio |
| *Eulonchus marginatus* | 009306 | unknown | CAS | USA, California, Marin County, Carson Ridge, [37.967, -122.633], 30.V.1959, J. R. Powers |
| *Eulonchus marginatus* | 009308 | unknown | CAS | USA, California, Contra Costa County, near Clayton, Mitchell Canyon, [37.919, -121.941], 27.V.1961, J. F. Lawrence |
| *Eulonchus marginatus* | 009309 | unknown | CAS | USA, California, Contra Costa County, Mt. Diablo, [37.881, -121.914], 5.VI.1960, J.A. Goodwin |
| *Eulonchus marginatus* | 009310 | unknown | CAS | USA, California, 29 km W. of Patterson, Del Puerto Canyon, [37.473, -121.457], 14.V.1963, J. W. MacSwain |
| *Eulonchus marginatus* | 009311 | unknown | CAS | USA, California, Santa Clara County, Stevens Creek Area, [37.431, -122.066], 26.V.1954, J. W. Tilden |
| *Eulonchus marginatus* | 009312 | unknown | CAS | USA, California, Contra Costa County, Mt. Diablo, [37.881, -121.914], 5.VI.1960, J.A. Goodwin |
| *Eulonchus marginatus* | 009313 | unknown | CAS | USA, California, Santa Clara County, Herbert Creek, [37.158, -121.843], 30.V.1965, P. A. Opler |
| *Eulonchus marginatus* | 009314 | unknown | CAS | USA, California, Marin County, Carson Ridge, [37.967, -122.633], 30.V.1959, J. R. Powers |
| *Eulonchus marginatus* | 009383 | unknown | CAS | USA, California, Marin County, near Woodacre, Carson Ridge, 8.VI.1966, J. Slater |
| *Eulonchus marginatus* | 011699 | unknown | UCDC | USA, California, Santa Cruz County, Santa Cruz, [36.974, -122.03], 1.VI.1936, G. E. Bohart |
| *Eulonchus marginatus* | 011700 | unknown | CAS | USA, California, Santa Cruz County, Highland District, [37.081, -122.081], 17.VI.1956, S. M. Fidel |
| *Eulonchus marginatus* | 011702 | unknown | CAS | USA, California, Santa Cruz County, 8.1 km east Glenwood, [37.109, -121.896], 2.VII.1956, D. J. Burdick |
| *Eulonchus marginatus* | 011873 | female | CAS | USA, California, Contra Costa Co., Briones Regional Park [North Briones Reservation], Hampton Road Canyon, [37.943, -122.141], 28.VI.1977, M. E. Buegler |
| *Eulonchus marginatus* | 011874 | female | CAS | USA, California, Contra Costa Co., Briones Regional Park [North Briones Reservation], Hampton Road Canyon, [37.943, -122.141], 28.VI.1977, M. E. Buegler |
| *Eulonchus marginatus* | 011879 | female | CAS | USA, California, Alameda Co., Strawberry Canyon, Berkeley, fire trail, [37.874, -122.228], 27.VI.1977, M. E. Buegler |
| *Eulonchus marginatus* | 011879 | unknown | CAS | USA, California, Nevada County, 5 miles south of Washington, [39.287, -120.801], 31.V.1977, E. I. Schlinger |
| *Eulonchus marginatus* | 011884 | female | CAS | USA, California, Alameda Co., Strawberry Canyon, Berkeley, fire trail, [37.874, -122.228], 27.VI.1977, M. E. Buegler |
| *Eulonchus marginatus* | 011886 | unknown | CAS | USA, California, Contra Costa Co., Briones Regional Park [North Briones Reservation], Hampton Road Canyon, [37.943, -122.141], 16.VI.1977, M. E. Buegler |
| *Eulonchus marginatus* | 012174 | female | CAS | USA, California, Santa Clara County, Mt. Hamilton Site, [37.341, -121.643], 17.VI.1969, A. R. Moldenke |
| *Eulonchus marginatus* | 012175 | female | CAS | USA, California, San Mateo Co., Campus Experimental Area, [37.405, -122.242], 26.IV.1969, A. R. Moldenke |
| *Eulonchus marginatus* | 012176 | female | CAS | USA, California, San Mateo Co., Campus Experimental Area, [37.405, -122.242], 26.IV.1969, A. R. Moldenke |
| *Eulonchus marginatus* | 012178 | female | CAS | USA, California, San Mateo Co., Campus Experimental Area, [37.405, -122.242], 26.IV.1969, A. R. Moldenke |
| *Eulonchus marginatus* | 012179 | female | CAS | USA, California, San Mateo Co., Campus Experimental Area, [37.405, -122.242], 26.IV.1969, A. R. Moldenke |
| *Eulonchus marginatus* | 012180 | female | CAS | USA, California, San Mateo Co., Campus Experimental Area, [37.405, -122.242], 26.IV.1969, A. R. Moldenke |
| *Eulonchus marginatus* | 012181 | female | CAS | USA, California, San Mateo Co., Campus Experimental Area, [37.405, -122.242], 26.IV.1969, A. R. Moldenke |
| *Eulonchus marginatus* | 012182 | female | CAS | USA, California, San Mateo Co., Campus Experimental Area, [37.405, -122.242], 26.IV.1969, A. R. Moldenke |
| *Eulonchus marginatus* | 012210 | male | CAS | USA, California, San Mateo Co., Campus Experimental Area, [37.405, -122.242], 26.IV.1969, A. R. Moldenke |
| *Eulonchus marginatus* | 012211 | male | CAS | USA, California, San Mateo Co., Campus Experimental Area, [37.405, -122.242], 26.IV.1969, A. R. Moldenke |
| *Eulonchus marginatus* | 012212 | male | CAS | USA, California, San Mateo Co., Campus Experimental Area, [37.405, -122.242], 26.IV.1969, A. R. Moldenke |
| *Eulonchus marginatus* | 012213 | male | CAS | USA, California, San Mateo Co., Campus Experimental Area, [37.405, -122.242], 26.IV.1969, A. R. Moldenke |
| *Eulonchus marginatus* | 012214 | male | CAS | USA, California, San Mateo Co., Campus Experimental Area, [37.405, -122.242], 26.IV.1969, A. R. Moldenke |
| *Eulonchus marginatus* | 012215 | male | CAS | USA, California, San Mateo Co., Campus Experimental Area, [37.405, -122.242], 26.IV.1969, A. R. Moldenke |
| *Eulonchus marginatus* | 012216 | male | CAS | USA, California, San Mateo Co., Campus Experimental Area, [37.405, -122.242], 26.IV.1969, A. R. Moldenke |
| *Eulonchus marginatus* | 012217 | male | CAS | USA, California, San Mateo Co., Campus Experimental Area, [37.405, -122.242], 26.IV.1969, A. R. Moldenke |
| *Eulonchus marginatus* | 012218 | male | CAS | USA, California, San Mateo Co., Campus Experimental Area, [37.405, -122.242], 26.IV.1969, A. R. Moldenke |
| *Eulonchus marginatus* | 012219 | male | CAS | USA, California, San Mateo Co., Campus Experimental Area, [37.405, -122.242], 26.IV.1969, A. R. Moldenke |
| *Eulonchus marginatus* | 012220 | male | CAS | USA, California, San Mateo Co., Campus Experimental Area, [37.405, -122.242], 26.IV.1969, A. R. Moldenke |
| *Eulonchus marginatus* | 012221 | male | CAS | USA, California, San Mateo Co., Campus Experimental Area, [37.405, -122.242], 26.IV.1969, A. R. Moldenke |
| *Eulonchus marginatus* | 012222 | male | CAS | USA, California, San Mateo Co., Campus Experimental Area, [37.405, -122.242], 26.IV.1969, A. R. Moldenke |
| *Eulonchus marginatus* | 012223 | male | CAS | USA, California, San Mateo Co., Campus Experimental Area, [37.405, -122.242], 26.IV.1969, A. R. Moldenke |
| *Eulonchus marginatus* | 012224 | male | CAS | USA, California, San Mateo Co., Campus Experimental Area, [37.405, -122.242], 26.IV.1969, A. R. Moldenke |
| *Eulonchus marginatus* | 012225 | male | CAS | USA, California, San Mateo Co., Campus Experimental Area, [37.405, -122.242], 26.IV.1969, A. R. Moldenke |
| *Eulonchus marginatus* | 012226 | male | CAS | USA, California, San Mateo Co., Campus Experimental Area, [37.405, -122.242], 26.IV.1969, A. R. Moldenke |
| *Eulonchus marginatus* | 012227 | male | CAS | USA, California, San Mateo Co., Campus Experimental Area, [37.405, -122.242], 26.IV.1969, A. R. Moldenke |
| *Eulonchus marginatus* | 012228 | male | CAS | USA, California, San Mateo Co., Campus Experimental Area, [37.405, -122.242], 26.IV.1969, A. R. Moldenke |
| *Eulonchus marginatus* | 012229 | male | CAS | USA, California, San Mateo Co., Campus Experimental Area, [37.405, -122.242], 26.IV.1969, A. R. Moldenke |
| *Eulonchus marginatus* | 012230 | male | CAS | USA, California, San Mateo Co., Campus Experimental Area, [37.405, -122.242], 26.IV.1969, A. R. Moldenke |
| *Eulonchus marginatus* | 012231 | male | CAS | USA, California, San Mateo Co., Campus Experimental Area, [37.405, -122.242], 26.IV.1969, A. R. Moldenke |
| *Eulonchus marginatus* | 012232 | male | CAS | USA, California, San Mateo Co., Campus Experimental Area, [37.405, -122.242], 26.IV.1969, A. R. Moldenke |
| *Eulonchus marginatus* | 012747 | both | CAS | USA, California, Napa County, 11 miles east of Rutherford, [38.459, -122.412], 30.V.1968, P. Welies |
| *Eulonchus marginatus* | 012748 | female | CAS | USA, California, Napa County, 11 miles east of Rutherford, [38.459, -122.412], 30.V.1968, P. Welies |
| *Eulonchus marginatus* | 012749 | male | CAS | USA, California, Napa County, 11 miles east of Rutherford, [38.459, -122.412], 30.V.1968, P. Welies |
| *Eulonchus marginatus* | 012766 | male | CAS | USA, California, Napa County, Conn Dam, 30.V.1968, P. Welies |
| *Eulonchus marginatus* | 012975 | male | CAS | USA, California, Napa County, 7 miles east of Conn Dam, [38.489, -122.376], 12.VI.1964, R. W. Thorp |
| *Eulonchus marginatus* | 012976 | male | CAS | USA, California, Napa County, 7 miles east of Conn Dam, [38.489, -122.376], 12.VI.1964, R. W. Thorp |
| *Eulonchus marginatus* | 012977 | male | CAS | USA, California, Napa County, 7 miles east of Conn Dam, [38.489, -122.376], 12.VI.1964, R. W. Thorp |
| *Eulonchus marginatus* | 012982 | female | CAS | USA, California, Stanislaus, Del Puerto Canyon, [37.469, -121.457], 9.V.1964, J. W. MacSwain |
| *Eulonchus marginatus* | 012983 | female | CAS | USA, California, Stanislaus, Del Puerto Canyon, [37.469, -121.457], 9.V.1964, J. W. MacSwain |
| *Eulonchus marginatus* | 012984 | female | CAS | USA, California, Stanislaus, Del Puerto Canyon, [37.469, -121.457], 9.V.1964, J. W. MacSwain |
| *Eulonchus marginatus* | 012995 | female | CAS | USA, California, Napa County, 15 miles South of Monticello, [38.362, -122.207], 427 m, 16.VI.1953, R. C. Bechtel |
| *Eulonchus marginatus* | 012996 | male | CAS | USA, California, Napa County, 15 miles South of Monticello, [38.362, -122.207], 427 m, 16.VI.1953, R. C. Bechtel |
| *Eulonchus marginatus* | 013946 | unknown | CAS | USA, California, Mendocino County, Hopland, [38.973, -123.116], 853 m, 29.VI.1971, aerial net, R.S. Lane |
| *Eulonchus marginatus* | 013947 | unknown | CAS | USA, California, Mendocino County, Hopland, [38.973, -123.116], 853 m, 29.VI.1971, aerial net, R.S. Lane |
| *Eulonchus marginatus* | 013948 | unknown | CAS | USA, California, Contra Costa County, Orinda Village, below Eagle Peak, San Pablo Ridge, [37.935, -122.271], 305 to 366 m, 21.VI.1971, Swimming Pool, E. I. Schlinger |
| *Eulonchus marginatus* | 014109 | unknown | CAS | USA, California, Marin County, Mill Valley, [37.906, -122.545], 110 m, 26.V.1965 to 27.V.1965, flight trapP. H. Arnaud, Jr. |
| *Eulonchus marginatus* | 014545 | unknown | CAS | USA, California, Solano County, 8 Km WNW Vacaville, Gates Cyn, nr. merge of Alamo Creek & S. Fork Alamo Creek, [38.382, -122.038], 335 m, 14.VI.1994 to 20.VI.1994, Malaise trap, S. D. Gaimari |
| *Eulonchus marginatus* | 014683 | unknown | CAS | USA, California, San Mateo County, Portola Valley, Alpine Road, Corte Madera Creek, [37.401, -122.238], 7.V.1960, P. H. Arnaud, Jr. |
| *Eulonchus marginatus* | 014686 | unknown | CAS | USA, California, Alameda County, Sunol Valley Regional Park, [37.511, -121.828], 120 m, 1.VI.1968, P. H. Arnaud, Jr. |
| *Eulonchus marginatus* | 014687 | unknown | CAS | USA, California, Alameda County, Sunol Valley Regional Park, [37.511, -121.828], 120 m, 1.VI.1968, P. H. Arnaud, Jr. |
| *Eulonchus marginatus* | 014745 | unknown | CAS | USA, California, Santa Clara County, Alum Rock Park, [37.388, -121.788], 14.V.1962 |
| *Eulonchus marginatus* | 014771 | unknown | CAS | USA, California, Marin County, Mill Valley, [37.906, -122.545], 110 m, 24.V.1965 to 25.V.1965, Malaise trap, P. H. Arnaud, Jr. |
| *Eulonchus marginatus* | 014774 | unknown | CAS | USA, California, Marin County, Mill Valley, [37.906, -122.545], 110 m, 24.V.1965 to 25.V.1965, Malaise trap, P. H. Arnaud, Jr. |
| *Eulonchus marginatus* | 014785 | unknown | CAS | USA, California, San Mateo County, Searsville Lake, [37.404, -122.238], 21.V.1953, P. H. Arnaud, Jr. |
| *Eulonchus marginatus* | 014787 | unknown | CAS | USA, California, Marin County, SW Fairfax, Fairfax-Bolinas Rd, Pine Mountain Truck Road, [37.977, -122.597], 26.VI.1999, P. H. Arnaud, Jr., M. M. Arnaud |
| *Eulonchus marginatus* | 015487 | unknown | CAS | USA, California, Mendocino County, Hopland Grade, [38.98, -123.009], 20.V.1989, R. Robertson, J. K. Robertson |
| *Eulonchus marginatus* | 016995 | female | CAS | USA, California, Contra Costa County, Orinda Village, below Eureka Peak, San Pablo Ridge, [37.935, -122.271], 305 to 365 m, 25.VI.1977, E. I. Schlinger |
| *Eulonchus marginatus* | 016996 | female | CAS | USA, California, Contra Costa County, Orinda Village, below Eureka Peak, San Pablo Ridge, [37.935, -122.271], 305 to 365 m, 25.VI.1977, E. I. Schlinger |
| *Eulonchus marginatus* | 016997 | female | CAS | USA, California, Contra Costa County, Orinda Village, below Eureka Peak, San Pablo Ridge, [37.935, -122.271], 305 to 365 m, 25.VI.1977, E. I. Schlinger |
| *Eulonchus marginatus* | 016998 | female | CAS | USA, California, Contra Costa County, Orinda Village, below Eureka Peak, San Pablo Ridge, [37.935, -122.271], 305 to 365 m, 25.VI.1977, E. I. Schlinger |
| *Eulonchus marginatus* | 016999 | female | CAS | USA, California, Contra Costa County, Orinda Village, below Eureka Peak, San Pablo Ridge, [37.935, -122.271], 305 to 365 m, 25.VI.1977, E. I. Schlinger |
| *Eulonchus marginatus* | 017000 | female | CAS | USA, California, Contra Costa County, Orinda Village, below Eureka Peak, San Pablo Ridge, [37.935, -122.271], 305 to 365 m, 25.VI.1977, E. I. Schlinger |
| *Eulonchus marginatus* | 017001 | female | CAS | USA, California, Contra Costa County, Orinda Village, below Eureka Peak, San Pablo Ridge, [37.935, -122.271], 305 to 365 m, 25.VI.1977, E. I. Schlinger |
| *Eulonchus marginatus* | 017002 | female | CAS | USA, California, Contra Costa County, Orinda Village, below Eureka Peak, San Pablo Ridge, [37.935, -122.271], 305 to 365 m, 25.VI.1977, E. I. Schlinger |
| *Eulonchus marginatus* | 017003 | female | CAS | USA, California, Contra Costa County, Orinda Village, below Eureka Peak, San Pablo Ridge, [37.935, -122.271], 305 to 365 m, 27.VI.1977, hand netted, E. I. Schlinger |
| *Eulonchus marginatus* | 017004 | female | CAS | USA, California, Contra Costa County, Orinda Village, below Eureka Peak, San Pablo Ridge, [37.935, -122.271], 305 to 365 m, 27.VI.1977, hand netted, E. I. Schlinger |
| *Eulonchus marginatus* | 017005 | female | CAS | USA, California, Contra Costa County, Orinda Village, below Eureka Peak, San Pablo Ridge, [37.935, -122.271], 305 to 365 m, 27.VI.1977, hand netted, E. I. Schlinger |
| *Eulonchus marginatus* | 017006 | male | CAS | USA, California, Contra Costa County, Orinda Village, below Eureka Peak, San Pablo Ridge, [37.935, -122.271], 305 to 365 m, 25.VI.1977, E. I. Schlinger |
| *Eulonchus marginatus* | 017007 | male | CAS | USA, California, Contra Costa County, Orinda Village, below Eureka Peak, San Pablo Ridge, [37.935, -122.271], 305 to 365 m, 27.VI.1977, hand netted, E. I. Schlinger |
| *Eulonchus marginatus* | 017008 | male | CAS | USA, California, Contra Costa County, Orinda Village, below Eureka Peak, San Pablo Ridge, [37.935, -122.271], 305 to 365 m, 27.VI.1977, hand netted, E. I. Schlinger |
| *Eulonchus marginatus* | 017009 | male | CAS | USA, California, Contra Costa County, Orinda Village, below Eureka Peak, San Pablo Ridge, [37.935, -122.271], 305 to 365 m, 27.VI.1977, hand netted, E. I. Schlinger |
| *Eulonchus marginatus* | 018797 | female | CAS | USA, California, Santa Clara County, San Antonio Valley, [37.355, -121.92], 13.VI.1950, W. F. Barr |
| *Eulonchus marginatus* | 018807 | male | CAS | USA, California, H. Edwards |
| *Eulonchus marginatus* | 019049 | unknown | CAS | USA, California, Marin County, Carson Ridge, [37.967, -122.633], 30.V.1959, J. R. Powers |
| *Eulonchus marginatus* | 019075 | unknown | CAS | USA, California, Stanislaus County, Frank Raines Park, Del Puerto Canyon, [37.423, -121.375], 366 m, 24.V.1980, E. I. Schlinger |
| *Eulonchus marginatus* | 019076 | unknown | CAS | USA, California, Stanislaus County, Frank Raines Park, Del Puerto Canyon, [37.423, -121.375], 366 m, 24.V.1980, E. I. Schlinger |
| *Eulonchus marginatus* | 019086 | unknown | CAS | USA, California, Alameda County, Tilden Park, Berkeley, 8.VI.1973, hand netted, D. S. Green |
| *Eulonchus marginatus* | 019097 | unknown | CAS | USA, California, Alameda County, Tilden Park, Lake Anza, [37.896, -122.252], 5.VI.1977, hand netted, M. E. Buegler |
| *Eulonchus marginatus* | 019098 | unknown | CAS | USA, California, Mendocino County, Hopland Field Station, [39.001, -123.083], 30.V.1982, D. Young |
| *Eulonchus marginatus* | 019099 | unknown | CAS | USA, California, Mendocino County, Hopland Field Station, [39.001, -123.083], 30.V.1982, D. Young |
| *Eulonchus marginatus* | 019122 | unknown | CAS | USA, California, Contra Costa County, Orinda Village, below Eureka Peak, San Pablo Ridge, [37.935, -122.271], 305 to 365 m, 27.VI.1977, hand netted, E. I. Schlinger |
| *Eulonchus marginatus* | 019123 | unknown | CAS | USA, California, Contra Costa County, Orinda Village, below Eureka Peak, San Pablo Ridge, [37.935, -122.271], 305 to 365 m, 27.VI.1977, hand netted, E. I. Schlinger |
| *Eulonchus marginatus* | 019125 | unknown | CAS | USA, California, Contra Costa County, Orinda Village, below Eagle Peak, San Pablo Ridge, [37.935, -122.271], 305 to 366 m, 28.VI.1977, hand netted, E. I. Schlinger |
| *Eulonchus marginatus* | 019443 | female | CAS | USA, California, Contra Costa County, Orinda Village, below Eureka Peak, San Pablo Ridge, [37.935, -122.271], 305 to 365 m, 31.V.1976, E. I. Schlinger |
| *Eulonchus marginatus* | 019444 | unknown | CAS | USA, California, Contra Costa County, Orinda Village, below Eureka Peak, San Pablo Ridge, [37.935, -122.271], 305 to 365 m, 31.V.1976, E. I. Schlinger |
| *Eulonchus marginatus* | 019454 | unknown | CAS | USA, California, Solano County, Cold Creek, 0.8 km Monticello Damn, [38.513, -122.09], 30.V.1974, M. K. Sears |
| *Eulonchus marginatus* | 019455 | unknown | CAS | USA, California, Solano County, Cold Creek, 0.8 km Monticello Damn, [38.513, -122.09], 30.V.1974, M. K. Sears |
| *Eulonchus marginatus* | 019456 | unknown | CAS | USA, California, Contra Costa County, Lafayette, Diablo View Road, [37.927, -122.091], time of day: 6:30 pm, 14.V.1974, hand netted, D. S. Green |
| *Eulonchus marginatus* | 019480 | unknown | CAS | USA, California, Marin County, Mill Valley, [37.906, -122.545], 110 m, 14.VI.1965 to 16.VI.1965, P. H. Arnaud, Jr. |
| *Eulonchus marginatus* | 019481 | unknown | CAS | USA, California, Marin County, Mill Valley, [37.906, -122.545], 110 m, 14.VI.1965 to 16.VI.1965, P. H. Arnaud, Jr. |
| *Eulonchus marginatus* | 019484 | unknown | CAS | USA, California, Marin County, Mill Valley, [37.906, -122.545], 110 m, 14.VI.1965 to 16.VI.1965, P. H. Arnaud, Jr. |
| *Eulonchus marginatus* | 019485 | unknown | CAS | USA, California, Marin County, Mill Valley, [37.906, -122.545], 110 m, 14.VI.1965 to 16.VI.1965, P. H. Arnaud, Jr. |
| *Eulonchus marginatus* | 019557 | unknown | CAS | USA, California, Marin County, Mill Valley, [37.906, -122.545], 110 m, 14.VI.1965 to 16.VI.1965, P. H. Arnaud, Jr. |
| *Eulonchus marginatus* | 019561 | unknown | CAS | USA, California, Marin County, Mill Valley, [37.906, -122.545], 110 m, 14.VI.1965 to 16.VI.1965, P. H. Arnaud, Jr. |
| *Eulonchus marginatus* | 019563 | unknown | CAS | USA, California, Marin County, Mill Valley, [37.906, -122.545], 110 m, 14.VI.1965 to 16.VI.1965, P. H. Arnaud, Jr. |
| *Eulonchus marginatus* | 019569 | unknown | CAS | USA, California, Marin County, Mill Valley, [37.906, -122.545], 110 m, 14.VI.1965 to 16.VI.1965, P. H. Arnaud, Jr. |
| *Eulonchus marginatus* | 019570 | unknown | CAS | USA, California, Marin County, Mill Valley, [37.906, -122.545], 110 m, 14.VI.1965 to 16.VI.1965, P. H. Arnaud, Jr. |
| *Eulonchus marginatus* | 019576 | unknown | CAS | USA, California, Marin County, Mill Valley, [37.906, -122.545], 110 m, 14.VI.1965 to 16.VI.1965, P. H. Arnaud, Jr. |
| *Eulonchus marginatus* | 019579 | unknown | CAS | USA, California, Marin County, Mill Valley, [37.906, -122.545], 110 m, 14.VI.1965 to 16.VI.1965, P. H. Arnaud, Jr. |
| *Eulonchus marginatus* | 019580 | unknown | CAS | USA, California, Marin County, Mill Valley, [37.906, -122.545], 110 m, 14.VI.1965 to 16.VI.1965, P. H. Arnaud, Jr. |
| *Eulonchus marginatus* | 019581 | unknown | CAS | USA, California, Marin County, Mill Valley, [37.906, -122.545], 110 m, 14.VI.1965 to 16.VI.1965, P. H. Arnaud, Jr. |
| *Eulonchus marginatus* | 019611 | unknown | CAS | USA, California, Marin County, Mill Valley, Blithedale Ridge, Lee Street, [37.921, -122.553], 110 m, 24.VI.1965 to 28.VI.1965, Malaise trap, P. H. Arnaud, Jr. |
| *Eulonchus marginatus* | 019625 | unknown | CAS | USA, California, Marin County, Mill Valley, Blithedale Ridge, Lee Street, [37.921, -122.553], 110 m, 24.VI.1965 to 28.VI.1965, Malaise trap, P. H. Arnaud, Jr. |
| *Eulonchus marginatus* | 019786 | unknown | CAS | USA, California, Contra Costa County, Orinda Village, below Eureka Peak, San Pablo Ridge, [37.935, -122.271], 305 to 365 m, 5.VI.1973, E. I. Schlinger |
| *Eulonchus marginatus* | 019787 | unknown | CAS | USA, California, Contra Costa County, Orinda Village, below Eureka Peak, San Pablo Ridge, [37.935, -122.271], 305 to 365 m, 4.VI.1973, E. I. Schlinger |
| *Eulonchus marginatus* | 019788 | unknown | CAS | USA, California, Contra Costa County, Orinda Village, below Eureka Peak, San Pablo Ridge, [37.935, -122.271], 305 to 365 m, 4.VI.1973, E. I. Schlinger |
| *Eulonchus marginatus* | 019789 | unknown | CAS | USA, California, Contra Costa County, Orinda Village, below Eureka Peak, San Pablo Ridge, [37.935, -122.271], 305 to 365 m, 4.VI.1973, E. I. Schlinger |
| *Eulonchus marginatus* | 019790 | unknown | CAS | USA, California, Contra Costa County, Orinda Village, below Eureka Peak, San Pablo Ridge, [37.935, -122.271], 305 to 365 m, 4.VI.1973, E. I. Schlinger |
| *Eulonchus marginatus* | 019816 | unknown | CAS | USA, California, Santa Clara County, Alum Rock Park, [37.388, -121.788], 7.VI.1962, A. Edwards, J. G. Edwards |
| *Eulonchus marginatus* | 019821 | unknown | CAS | USA, California, Contra Costa County, Orinda Village, below Eureka Peak, San Pablo Ridge, [37.935, -122.271], 305 to 365 m, 21.VI.1975, E. I. Schlinger |
| *Eulonchus marginatus* | 019823 | unknown | CAS | USA, California, Contra Costa County, Orinda Village, below Eureka Peak, San Pablo Ridge, [37.935, -122.271], 305 to 365 m, 21.VI.1975, E. I. Schlinger |
| *Eulonchus marginatus* | 019824 | unknown | CAS | USA, California, Contra Costa County, Orinda Village, below Eureka Peak, San Pablo Ridge, [37.935, -122.271], 305 to 365 m, 21.VI.1975, E. I. Schlinger |
| *Eulonchus marginatus* | 019825 | unknown | CAS | USA, California, Contra Costa County, Orinda Village, below Eureka Peak, San Pablo Ridge, [37.935, -122.271], 305 to 365 m, 29.VI.1975, E. I. Schlinger |
| *Eulonchus marginatus* | 019826 | unknown | CAS | USA, California, Contra Costa County, Orinda Village, below Eureka Peak, San Pablo Ridge, [37.935, -122.271], 305 to 365 m, 7.VI.1976, Swimming Pool, E. I. Schlinger |
| *Eulonchus marginatus* | 019829 | unknown | CAS | USA, California, Contra Costa County, Orinda Village, below Eureka Peak, San Pablo Ridge, [37.935, -122.271], 305 to 365 m, 4.VI.1972, Swimming Pool, E. I. Schlinger |
| *Eulonchus marginatus* | 019830 | unknown | CAS | USA, California, Contra Costa County, Orinda Village, below Eureka Peak, San Pablo Ridge, [37.935, -122.271], 305 to 365 m, 4.VI.1972, Swimming Pool, E. I. Schlinger |
| *Eulonchus marginatus* | 019839 | unknown | CAS | USA, California, Santa Clara County, Alum Rock Park, near San Jose, by Penitencia Creek, [37.395, -121.823], 21.V.1972, R. W. Garrison |
| *Eulonchus marginatus* | 019840 | unknown | CAS | USA, California, Santa Clara County, Alum Rock Park, near San Jose, by Penitencia Creek, [37.395, -121.823], 21.V.1972, R. W. Garrison |
| *Eulonchus marginatus* | 019844 | unknown | CAS | USA, California, Contra Costa County, Orinda Village, below Eureka Peak, San Pablo Ridge, [37.935, -122.271], 305 to 365 m, 21.VI.1972, E. I. Schlinger |
| *Eulonchus marginatus* | 019845 | unknown | CAS | USA, California, Contra Costa County, Orinda Village, below Eureka Peak, San Pablo Ridge, [37.935, -122.271], 305 to 365 m, 21.VI.1972, E. I. Schlinger |
| *Eulonchus marginatus* | 019887 | unknown | CAS | USA, California, Alameda County, Arroyo Mocho Canyon, [37.667, -121.765], 24.V.1972, hand netted, W. H. Tyson |
| *Eulonchus marginatus* | 019888 | unknown | CAS | USA, California, Alameda County, Arroyo Mocho Canyon, [37.667, -121.765], 24.V.1972, hand netted, W. H. Tyson |
| *Eulonchus marginatus* | 019892 | unknown | CAS | USA, California, Santa Clara County, Stevens Creek Area, [37.431, -122.066], 29.VI.1965, T. L. Erwin |
| *Eulonchus marginatus* | 019915 | unknown | CAS | USA, California, Marin County, Mill Valley, Blithedale Ridge, Lee Street, [37.921, -122.553], 110 m, 24.VI.1965 to 28.VI.1965, Malaise trap, P. H. Arnaud, Jr. |
| *Eulonchus marginatus* | 019917 | unknown | CAS | USA, California, Marin County, Mill Valley, Blithedale Ridge, Lee Street, [37.921, -122.553], 110 m, 24.VI.1965 to 28.VI.1965, Malaise trap, P. H. Arnaud, Jr. |
| *Eulonchus marginatus* | 019934 | unknown | CAS | USA, California, Marin County, Mill Valley, Blithedale Ridge, Lee Street, [37.921, -122.553], 110 m, 24.VI.1965 to 28.VI.1965, Malaise trap, P. H. Arnaud, Jr. |
| *Eulonchus marginatus* | 019952 | unknown | CAS | USA, California, Marin County, Mill Valley, Blithedale Ridge, Lee Street, [37.921, -122.553], 110 m, 24.VI.1965 to 28.VI.1965, Malaise trap, P. H. Arnaud, Jr. |
| *Eulonchus marginatus* | 019958 | unknown | CAS | USA, California, Marin County, Mill Valley, Blithedale Ridge, Lee Street, [37.921, -122.553], 110 m, 24.VI.1965 to 28.VI.1965, Malaise trap, P. H. Arnaud, Jr. |
| *Eulonchus marginatus* | 019964 | unknown | CAS | USA, California, Marin County, Mill Valley, Blithedale Ridge, Lee Street, [37.921, -122.553], 110 m, 24.VI.1965 to 28.VI.1965, Malaise trap, P. H. Arnaud, Jr. |
| *Eulonchus marginatus* | 019970 | unknown | CAS | USA, California, Napa County, 5.5 km NW Moskowite Corner, Capell Creek, [38.475, -122.24], 200 m, 12.VI.1977, Malaise trap, P. H. Arnaud, Jr. |
| *Eulonchus marginatus* | 019971 | unknown | CAS | USA, California, Napa County, 5.5 km NW Moskowite Corner, Capell Creek, [38.475, -122.24], 200 m, 12.VI.1977, Malaise trap, P. H. Arnaud, Jr. |
| *Eulonchus marginatus* | 020053 | unknown | USNM | USA, California, Seavsville Lake, Searsville Lake, [37.404, -122.238], 12.V.1953 |
| *Eulonchus marginatus* | 020054 | unknown | USNM | USA, California, Marin County, Mill Valley, [37.906, -122.55], 17.VI.1965 to 19.VI.1965, flight trap |
| *Eulonchus marginatus* | 020055 | unknown | USNM | USA, California, Marin County, Mill Valley, [37.906, -122.55], 17.VI.1965 to 19.VI.1965, flight trap |
| *Eulonchus marginatus* | 020056 | unknown | USNM | USA, California, Marin County, Mill Valley, [37.906, -122.55], 17.VI.1965 to 19.VI.1965, flight trap |
| *Eulonchus marginatus* | 020057 | unknown | USNM | USA, California, Marin County, Mill Valley, [37.906, -122.55], 17.VI.1965 to 19.VI.1965, flight trap |
| *Eulonchus marginatus* | 020058 | unknown | USNM | USA, California, Marin County, Mill Valley, [37.906, -122.55], 17.VI.1965 to 19.VI.1965, flight trap |
| *Eulonchus marginatus* | 020059 | unknown | USNM | USA, California, Marin County, Mill Valley, [37.906, -122.55], 17.VI.1965 to 19.VI.1965, flight trap |
| *Eulonchus marginatus* | 020060 | unknown | USNM | USA, California, Marin County, Mill Valley, [37.906, -122.55], 17.VI.1965 to 19.VI.1965, flight trap |
| *Eulonchus marginatus* | 020061 | unknown | USNM | USA, California, Marin County, Mill Valley, [37.906, -122.55], 17.VI.1965 to 19.VI.1965, flight trap |
| *Eulonchus marginatus* | 020062 | unknown | USNM | USA, California, Marin County, Mill Valley, [37.906, -122.55], 17.VI.1965 to 19.VI.1965, flight trap |
| *Eulonchus marginatus* | 020170 | unknown | CAS | USA, California, Napa County, 12.9 Southwest of Monticello Dam, [38.505, -122.115], 27.V.1978 |
| *Eulonchus marginatus* | 020171 | unknown | CAS | USA, California, Napa County, 12.9 Southwest of Monticello Dam, [38.505, -122.115], 27.V.1978 |
| *Eulonchus marginatus* | 020172 | unknown | CAS | USA, California, Napa County, 12.9 Southwest of Monticello Dam, [38.505, -122.115], 22.V.1978 |
| *Eulonchus marginatus* | 020173 | unknown | CAS | USA, California, Napa County, mountains west of St. Helena, [38.49, -122.495], 23.V.1956, H. H. Keifer |
| *Eulonchus marginatus* | 020189 | unknown | CAS | USA, California, Mendocino County, Angelo Coast Range Reserve, 10.6 km ENE of Branscomb, Trail to Ahiquist pond, chaparral, [39.748, -123.601], 780 m, 14.V.2005, C.J. Borkent |
| *Eulonchus marginatus* | – | male | TCAC | USA, California, San Benito Co., New Idria Road, [36.412, -120.671], Criswold Cyn, 1800 feet, 1.VII.2007, R.D. Haines. |
| *Eulonchus marginatus* | – | male | TCAC | USA, California, San Benito Co., New Idria Road, [36.412, -120.671], Criswold Cyn, 1800 feet, 1.VII.2007, R.D. Haines. |
| *Eulonchus marginatus* | – | male | TCAC | USA, California, San Benito Co., New Idria Road, [36.412, -120.671], Criswold Cyn, 1800 feet, 1.VII.2007, R.D. Haines. |
| *Eulonchus marginatus* | – | male | TCAC | USA, California, San Benito Co., Criswold Cyn, 1500 feet, 23.V.2009, R.D. Haines. |
| *Eulonchus marginatus* | – | male | TCAC | USA, California, San Benito Co., New Idria Road, [36.412, -120.671], Criswold Cyn, 1800 feet, 1.VII.2007, R.D. Haines. |
| *Eulonchus marginatus* | – | male | TCAC | USA, California, San Benito Co., Willow Springs, [36.667, -121.035], 20.V.2013, G. McDonald. |
| *Eulonchus marginatus* | – | male | TCAC | USA, California, San Benito Co., Coalinga Road, 7.4 mi. E Highway 25, [36.360, -120.851], 30.V.2009, R.D. Haines. |
| *Eulonchus marginatus* | – | male | TCAC | USA, California, San Benito Co., New Idria Road, [36.412, -120.671], Criswold Cyn, 1800 feet, 1.VII.2007, R.D. Haines. |
| *Eulonchus marginatus* | – | male | TCAC | USA, California, San Benito Co., Willow Springs, [36.667, -121.035], 27.V.2013, G. McDonald. |
| *Eulonchus marginatus* | – | male | TCAC | USA, California, San Benito Co., Willow Springs, [36.667, -121.035], 27.V.2013, G. McDonald. |
| *Eulonchus marginatus* | – | male | TCAC | USA, California, San Benito Co., Willow Springs, [36.667, -121.035], 20.V.2013, G. McDonald. |
| *Eulonchus marginatus* | – | male | TCAC | USA, California, San Benito Co., Willow Springs, [36.667, -121.035], 20.V.2013, G. McDonald. |
| *Eulonchus marginatus* | – | male | TCAC | USA, California, San Benito Co., Willow Springs, [36.667, -121.035], 1.VI.2012, G. McDonald. |
| *Eulonchus marginatus* | – | male | TCAC | USA, California, San Benito Co., Willow Springs, [36.667, -121.035], 15.V.2013, G. McDonald. |
| *Eulonchus marginatus* | – | male | TCAC | USA, California, San Benito Co., Willow Springs, [36.667, -121.035], 15.V.2013, G. McDonald. |
| *Eulonchus marginatus* | – | male | TCAC | USA, California, San Benito Co., Willow Springs, [36.667, -121.035], 15.V.2013, G. McDonald. |
| *Eulonchus marginatus* | – | male | TCAC | USA, California, San Benito Co., Willow Springs, [36.667, -121.035], 15.V.2013, G. McDonald. |
| *Eulonchus marginatus* | – | male | TCAC | USA, California, San Benito Co., Willow Springs, [36.667, -121.035], 15.V.2013, G. McDonald. |
| *Eulonchus marginatus* | – | male | TCAC | USA, California, San Benito Co., Willow Springs, [36.667, -121.035], 15.V.2013, G. McDonald. |
| *Eulonchus marginatus* | – | male | TCAC | USA, California, San Benito Co., Willow Springs, [36.667, -121.035], 15.V.2013, G. McDonald. |
| *Eulonchus marginatus* | – | male | TCAC | USA, California, San Benito Co., New Idria Road, [36.412, -120.671], Criswold Cyn, 1800 feet, 1.VII.2007, R.D. Haines. |
| *Eulonchus marginatus* | – | male | TCAC | USA, California, San Benito Co., New Idria Road, [36.412, -120.671], Criswold Cyn, 1800 feet, 1.VII.2007, R.D. Haines. |
| *Eulonchus marginatus* | – | male | TCAC | USA, California, San Benito Co., Criswold Cyn, 1500 feet, 23.V.2009, R.D. Haines. |
| *Eulonchus marialiciae* | 009955 | male | CAS | USA, North Carolina, Swain County, Newfound Gap, [35.611, -83.426], 1463 m, 7.VI.1999, D. Defoe |
| *Eulonchus marialiciae* | 014838 | male | CAS | USA, North Carolina, Haywood County, [35.63, -82.99], 16.VI.1956, hand netted, H. V. Weems, Jr. |
| *Eulonchus marialiciae* | 014839 | unknown | CAS | USA, North Carolina, Haywood County, [35.63, -82.99], 12.VI.1956, hand netted, H. V. Weems, Jr. |
| *Eulonchus marialiciae* | 014840 | male | CAS | USA, North Carolina, Swain County, Great Smoky Mountains National Park, 2.2 mi S. of Newfound Gap, [35.6, -83.44], 1463 m, 7.VI.1999, hand netted, D. Defoe |
| *Eulonchus marialiciae* | 018961 | unknown | CAS | USA, North Carolina, Macon County, Highlands Biological Station, [35.05, -83.19], 1.VIII.1966 to 3.VIII.1966, F. A. Coyle |
| *Eulonchus marialiciae* | 018962 | unknown | CAS | USA, North Carolina, Macon County, Highlands Biological Station, [35.05, -83.19], 1.VIII.1966 to 3.VIII.1966, F. A. Coyle |
| *Eulonchus marialiciae* | 018963 | unknown | CAS | USA, North Carolina, Macon County, Highlands Biological Station, [35.05, -83.19], 1.VIII.1966 to 3.VIII.1966, F. A. Coyle |
| *Eulonchus sapphirinus* | 000221 | male | CAS | USA, California, Chester, [40.307, -121.232], 7.VII.1948, D. J. Knull, J. N. Knull |
| *Eulonchus sapphirinus* | 000222 | male | CAS | USA, California, Chester, [40.307, -121.232], 7.VII.1948, D. J. Knull, J. N. Knull |
| *Eulonchus sapphirinus* | 000223 | male | CAS | USA, California, Plumas County, Buck’s Lake, [39.868, -121.174], 23.VI.1949, J. W. MacSwain |
| *Eulonchus sapphirinus* | 000224 | male | CAS | USA, California, Plumas County, Buck’s Lake, [39.868, -121.174], 23.VI.1949, J. W. MacSwain |
| *Eulonchus sapphirinus* | 000225 | male | CAS | USA, California, Plumas County, Buck’s Lake, [39.868, -121.174], 23.VI.1949, J. W. MacSwain |
| *Eulonchus sapphirinus* | 000226 | male | CAS | USA, California, Truckee, [39.328, -120.184], 17.VI.1927, E. P. Van Duzee |
| *Eulonchus sapphirinus* | 000227 | male | CAS | USA, California, Plumas County, Blairsden, [39.781, -120.617], 4.VI.1958, J. S. Buckett |
| *Eulonchus sapphirinus* | 000228 | male | CAS | USA, California, Tehama County, Mineral, [40.349, -121.595], 6.VII.1952, M. A. Cazier, W. J. Gertsch, R. Schrammel |
| *Eulonchus sapphirinus* | 000229 | male | CAS | USA, California, Tehama County, Mineral, [40.349, -121.595], 6.VII.1952, M. A. Cazier, W. J. Gertsch, R. Schrammel |
| *Eulonchus sapphirinus* | 000230 | male | CAS | USA, California, Tehama County, Mineral, [40.349, -121.595], 6.VII.1952, M. A. Cazier, W. J. Gertsch, R. Schrammel |
| *Eulonchus sapphirinus* | 000231 | male | CAS | USA, California, Tehama County, Mineral, [40.349, -121.595], 6.VII.1952, M. A. Cazier, W. J. Gertsch, R. Schrammel |
| *Eulonchus sapphirinus* | 000232 | male | CAS | USA, California, Tehama County, Deer Creek, [39.937, -122.069], 6.VII.1952, M. A. Cazier, W. J. Gertsch, R. Schrammel |
| *Eulonchus sapphirinus* | 000233 | male | CAS | USA, California, Nevada County, near Hobart Mills, Sagehen Creek, [39.407, -120.19], 1.VII.1964, M. E. Irwin |
| *Eulonchus sapphirinus* | 000234 | male | CAS | USA, California, Nevada County, near Hobart Mills, Sagehen Creek, [39.407, -120.19], 1.VII.1964, M. E. Irwin |
| *Eulonchus sapphirinus* | 000235 | male | CAS | USA, California, Sierra County, Yuba Pass, [39.506, -120.419], 7.VII.1964, K. Lorenzen |
| *Eulonchus sapphirinus* | 000236 | male | CAS | USA, California, Nevada County, near Hobart Mills, Sagehen Creek, [39.407, -120.19], 24.VI.1964, M. E. Irwin |
| *Eulonchus sapphirinus* | 000237 | male | CAS | USA, California, Nevada County, near Hobart Mills, Sagehen Creek, [39.407, -120.19], 24.VI.1964, M. E. Irwin |
| *Eulonchus sapphirinus* | 000238 | male | CAS | USA, California, Nevada County, near Hobart Mills, Sagehen Creek, [39.407, -120.19], 24.VI.1964, M. E. Irwin |
| *Eulonchus sapphirinus* | 000239 | male | CAS | USA, California, Chester, [40.307, -121.232], 7.VII.1948, D. J. Knull, J. N. Knull |
| *Eulonchus sapphirinus* | 000240 | male | CAS | USA, California, Nevada County, near Hobart Mills, Sagehen Creek, [39.407, -120.19], 24.VI.1964, S. G. Seminoff |
| *Eulonchus sapphirinus* | 000241 | male | CAS | USA, California, Nevada County, near Hobart Mills, Sagehen Creek, [39.407, -120.19], 24.VI.1964, M. E. Irwin |
| *Eulonchus sapphirinus* | 000242 | male | CAS | USA, California, Nevada County, near Hobart Mills, Sagehen Creek, [39.407, -120.19], 8.VII.1964, A. R. Gillogly |
| *Eulonchus sapphirinus* | 000243 | male | CAS | USA, California, Nevada County, Sagehen, near Hobart Mills, [39.407, -120.19], 25.VI.1954, R. H. Goodwin |
| *Eulonchus sapphirinus* | 000244 | male | CAS | USA, California, Nevada County, Sagehen, near Hobart Mills, [39.407, -120.19], 25.VI.1954, R. H. Goodwin |
| *Eulonchus sapphirinus* | 000245 | male | CAS | USA, California, Plumas County, Meadow Valley, [39.929, -121.061] |
| *Eulonchus sapphirinus* | 000246 | male | CAS | USA, California, Truckee, [39.328, -120.184], 5.VII.1927, E. P. Van Duzee |
| *Eulonchus sapphirinus* | 000247 | male | CAS | USA, California, Chester, [40.307, -121.232], 12.VI.1941, D. J. Knull, J. N. Knull |
| *Eulonchus sapphirinus* | 000248 | male | CAS | USA, California, Chester, [40.307, -121.232], 25.VI.1951, D. J. Knull, J. N. Knull |
| *Eulonchus sapphirinus* | 000249 | male | CAS | USA, California, Chester, [40.307, -121.232], 30.VI.1960, D. J. Knull, J. N. Knull |
| *Eulonchus sapphirinus* | 000250 | male | CAS | USA, California, Nevada County, near Hobart Mills, Sagehen Creek, [39.407, -120.19], 24.VI.1964, M. E. Irwin |
| *Eulonchus sapphirinus* | 000251 | male | CAS | USA, California, Nevada County, near Hobart Mills, Sagehen Creek, [39.407, -120.19], 1.VII.1964, M. E. Irwin |
| *Eulonchus sapphirinus* | 000252 | female | CAS | USA, California, Mono County, Mono Lake, [38.009, -119.013], 20.VI.1951 |
| *Eulonchus sapphirinus* | 000253 | male | CAS | USA, California, Mono County, Mono Lake, [38.009, -119.013], 20.VI.1951 |
| *Eulonchus sapphirinus* | 000254 | female | CAS | USA, California, Chester, [40.307, -121.232], 25.VI.1951, D. J. Knull, J. N. Knull |
| *Eulonchus sapphirinus* | 000255 | female | CAS | USA, California, Plumas County, Blairsden, [39.781, -120.617], 4.VI.1958, J. S. Buckett |
| *Eulonchus sapphirinus* | 000256 | female | CAS | USA, California, Chester, [40.307, -121.232], 12.VII.1958, D. J. Knull, J. N. Knull |
| *Eulonchus sapphirinus* | 000257 | female | CAS | USA, California, Chester, [40.307, -121.232], 12.VI.1941, D. J. Knull, J. N. Knull |
| *Eulonchus sapphirinus* | 000258 | female | CAS | USA, California, Tehama County, Mineral, [40.349, -121.595], 6.VII.1952, M. A. Cazier, W. J. Gertsch, R. Schrammel |
| *Eulonchus sapphirinus* | 000259 | female | CAS | USA, California, Tehama County, Mineral, [40.349, -121.595], 6.VII.1952, M. A. Cazier, W. J. Gertsch, R. Schrammel |
| *Eulonchus sapphirinus* | 000260 | female | CAS | USA, California, Lassen National Park, 6.4 km east Manzanita Lake, [40.529, -121.487], 26.VI.1963, V. L. Vesterby |
| *Eulonchus sapphirinus* | 000261 | female | CAS | USA, Nevada, Washoe County, Mount Rose summit, [39.314, -119.897], 9.VII.1964, F. D. Parker |
| *Eulonchus sapphirinus* | 000262 | female | CAS | USA, California, Plumas County, Buck’s Lake, [39.868, -121.174], 1.VII.1949, J. W. MacSwain |
| *Eulonchus sapphirinus* | 000263 | female | CAS | USA, California, Plumas County, Buck’s Lake, [39.868, -121.174], 23.VI.1949, J. W. MacSwain |
| *Eulonchus sapphirinus* | 000264 | female | CAS | USA, California, Lassen County, Susan River Camp, [40.494, -121.093], 10.VII.1949, E. L. Atkinson |
| *Eulonchus sapphirinus* | 000265 | female | CAS | USA, California, Plumas County, Buck’s Lake, [39.868, -121.174], 23.VI.1949, C. I. Smith |
| *Eulonchus sapphirinus* | 000266 | female | CAS | USA, California, Nevada County, Sagehen, near Hobart Mills, [39.407, -120.19], 25.VI.1954, R. H. Goodwin |
| *Eulonchus sapphirinus* | 000267 | female | CAS | USA, California, Truckee, [39.328, -120.184], 4.VII.1927, E. P. Van Duzee |
| *Eulonchus sapphirinus* | 000268 | female | CAS | USA, California, Plumas County, Buck’s Lake, [39.868, -121.174], 1.VII.1949, J. W. MacSwain |
| *Eulonchus sapphirinus* | 000269 | female | CAS | USA, California, Plumas County, Buck’s Lake, [39.868, -121.174], 1.VII.1949, J. W. MacSwain |
| *Eulonchus sapphirinus* | 000270 | female | CAS | USA, California, Nevada County, Sagehen, near Hobart Mills, [39.407, -120.19], 25.VI.1954, R. H. Goodwin |
| *Eulonchus sapphirinus* | 000271 | female | CAS | USA, California, Plumas County, Buck’s Lake, [39.868, -121.174], 23.VI.1949, J. W. MacSwain |
| *Eulonchus sapphirinus* | 000407 | male | CAS | USA, California, Lake County, Hopland Grade, [38.997, -122.949], 7.VI.1960, S. M. Fidel |
| *Eulonchus sapphirinus* | 000424 | male | CAS | USA, California, Lake County, Blue Lakes, [39.175, -123.015], 9.VI.1959, S. M. Fidel |
| *Eulonchus sapphirinus* | 004106 | unknown | CAS | USA, Washington, Pierce County, Clover Creek, [47.142, -122.452], 7.VII.1980, T. L. Whitworth |
| *Eulonchus sapphirinus* | 004107 | unknown | CAS | USA, Utah, Cache County, Green Canyon, [41.851, -111.8], 30.V.1997, pan trap, F. D. Parker |
| *Eulonchus sapphirinus* | 004108 | unknown | CAS | USA, Utah, Cache County, Green Canyon, [41.851, -111.8], 30.V.1997, pan trap, F. D. Parker |
| *Eulonchus sapphirinus* | 004109 | unknown | CAS | USA, Utah, Cache County, Green Canyon, [41.851, -111.8], 6.VI.1997, yellow pan trap, F. D. Parker |
| *Eulonchus sapphirinus* | 004110 | unknown | CAS | USA, Utah, Cache County, Green Canyon, [41.851, -111.8], 27.V.1997, pan trap, F. D. Parker |
| *Eulonchus sapphirinus* | 004111 | male | CAS | USA, Utah, Cache County, north of Tony Grove Lake, Naomi Peak Trail, [41.895, -111.642], 2499 to 2621 m, 17.VII.1995, T. L. Griswold |
| *Eulonchus sapphirinus* | 004112 | female | CAS | USA, Utah, Cache County, north of Tony Grove Lake, Naomi Peak Trail, [41.895, -111.642], 2499 to 2621 m, 17.VII.1995, T. L. Griswold |
| *Eulonchus sapphirinus* | 004113 | unknown | CAS | USA, Utah, Sanpete County, head of Mill Stream, [39.749, -111.27], 3239 m, time of day: 12 pm, 18.VI.1989, S. M. Geer |
| *Eulonchus sapphirinus* | 004114 | unknown | CAS | USA, Utah, Cache County, north of Tony Grove Lake, Naomi Peak Trail, [41.895, -111.642], 2499 to 2621 m, 17.VII.1995, T. L. Griswold |
| *Eulonchus sapphirinus* | 004115 | unknown | CAS | USA, Utah, Cache County, north of Tony Grove Lake, Naomi Peak Trail, [41.895, -111.642], 2499 to 2621 m, 17.VII.1995, T. L. Griswold |
| *Eulonchus sapphirinus* | 004116 | unknown | CAS | USA, Utah, Cache County, north of Tony Grove Lake, Naomi Peak Trail, [41.895, -111.642], 2499 to 2621 m, 17.VII.1995, T. L. Griswold |
| *Eulonchus sapphirinus* | 004117 | unknown | CAS | USA, Utah, Cache County, north of Tony Grove Lake, Naomi Peak Trail, [41.895, -111.642], 2499 to 2621 m, 17.VII.1995, T. L. Griswold |
| *Eulonchus sapphirinus* | 004118 | unknown | CAS | USA, Utah, Cache County, Green Canyon, [41.851, -111.8], 2.VI.1997, decoy pan trap, F. D. Parker |
| *Eulonchus sapphirinus* | 004119 | unknown | CAS | USA, Idaho, Franklin County, Weston Canyon, [42.093, -112.077], 17.VI.1997, white pan trap, F. D. Parker |
| *Eulonchus sapphirinus* | 004120 | unknown | CAS | USA, Utah, Cache County, Logan Canyon, [41.74, -111.794], 1829 m, 21.VI.1997, pan trap, F. D. Parker |
| *Eulonchus sapphirinus* | 004121 | unknown | CAS | USA, Utah, Cache County, Cowley Canyon, [41.777, -111.62], 17.VI.1989 to 27.VI.1989, W. J. Hanson |
| *Eulonchus sapphirinus* | 004122 | unknown | CAS | USA, Washington, Pierce County, Pleasant Valley, 15.VII.1977, T. L. Whitworth |
| *Eulonchus sapphirinus* | 004123 | unknown | CAS | USA, Washington, Pierce County, Clover Creek, [47.142, -122.452], 7.VII.1979, T. L. Whitworth |
| *Eulonchus sapphirinus* | 004124 | unknown | CAS | USA, Washington, Pierce County, Pleasant Valley, 10.VII.1978, T. L. Whitworth |
| *Eulonchus sapphirinus* | 004125 | unknown | CAS | USA, Oregon, Josephine County, Lake Mountain, [42.091, -123.36], 1676 m, 1.VIII.1995, T. L. Griswold |
| *Eulonchus sapphirinus* | 004126 | unknown | CAS | USA, Oregon, Josephine County, 19.6 km southwest Williams, [42.083, -123.427], 1402 m, 1.VIII.1995, T. Griswold |
| *Eulonchus sapphirinus* | 004127 | unknown | CAS | USA, Oregon, Josephine County, Mount Elijah, [42.085, -123.371], 1707 m, 1.VIII.1995, T. L. Griswold |
| *Eulonchus sapphirinus* | 004128 | unknown | CAS | USA, Oregon, Josephine County, Siskiyou Mountains, 1.1 km SE of Pepper Camp, 1536 m, 1.VIII.1995, T. Griswold |
| *Eulonchus sapphirinus* | 004130 | unknown | CAS | USA, California, Amador County, northwest of Cooks Station, [38.541, -120.451], 29.V.1996, T. Griswold |
| *Eulonchus sapphirinus* | 004136 | unknown | CAS | USA, Oregon, Josephine County, Mount Elijah, [42.085, -123.371], 1707 m, 1.VIII.1995, T. L. Griswold |
| *Eulonchus sapphirinus* | 004137 | unknown | CAS | USA, Oregon, Josephine County, Lake Mountain, [42.091, -123.36], 1676 m, 1.VIII.1995, T. L. Griswold |
| *Eulonchus sapphirinus* | 004138 | unknown | CAS | USA, Oregon, Josephine County, Mount Elijah, [42.085, -123.371], 1707 m, 1.VIII.1995, T. L. Griswold |
| *Eulonchus sapphirinus* | 004143 | unknown | CAS | USA, Utah, Cache County, Green Canyon, [41.851, -111.8], 27.V.1997, malaise trap, F. D. Parker |
| *Eulonchus sapphirinus* | 006721 | female | CAS | USA, California, Sierra County, Gold Lake, [39.678, -120.657], 31.VII.1921, C. L. Fox |
| *Eulonchus sapphirinus* | 006722 | female | CAS | USA, California, Plumas County, Clover Valley, [39.975, -120.551], 17.VI.1923, J. O. Martin |
| *Eulonchus sapphirinus* | 006723 | female | CAS | USA, California, Manzanita Lake, [40.533, -121.567], .VI.1940, I. McCracken |
| *Eulonchus sapphirinus* | 006724 | female | CAS | USA, California, Sierra County, Gold Lake, [39.678, -120.657], 12.VII.1921, C. L. Fox |
| *Eulonchus sapphirinus* | 006725 | female | CAS | USA, California, Plumas County, Meadow Valley, [39.93, -121.061], 1067 to 1219 m, 5.VI.1924, E. C. Van Dyke |
| *Eulonchus sapphirinus* | 006726 | female | CAS | USA, California, Sierra County, Gold Lake, [39.678, -120.657], 12.VII.1921, C. L. Fox |
| *Eulonchus sapphirinus* | 006727 | female | CAS | USA, California, Plumas County, Meadow Valley, [39.93, -121.061], 1067 to 1219 m, 11.VI.1924, E. C. Van Dyke |
| *Eulonchus sapphirinus* | 006728 | female | CAS | USA, California, Plumas County, Meadow Valley, [39.93, -121.061], 1067 to 1219 m, 3.VI.1924, E. C. Van Dyke |
| *Eulonchus sapphirinus* | 006729 | female | CAS | USA, California, Sierra County, Gold Lake, [39.678, -120.657], 21.VII.1921, C. L. Fox |
| *Eulonchus sapphirinus* | 006730 | female | CAS | USA, California, Sierra County, Gold Lake, [39.678, -120.657], 10.VII.1921, C. L. Fox |
| *Eulonchus sapphirinus* | 006731 | female | CAS | USA, California, Sierra County, Gold Lake, [39.678, -120.657], 14.VII.1921, C. L. Fox |
| *Eulonchus sapphirinus* | 006732 | female | CAS | USA, California, Plumas County, Meadow Valley, [39.93, -121.061], 1067 to 1219 m, 16.VI.1924, E. C. Van Dyke |
| *Eulonchus sapphirinus* | 006733 | female | CAS | USA, California, Marin County, Mill Valley, =, [37.906, -122.545], 6.V.1968, H. B. Leech |
| *Eulonchus sapphirinus* | 006734 | male | CAS | USA, California, Marin County, Mill Valley, =, [37.906, -122.545], 6.V.1968, H. B. Leech |
| *Eulonchus sapphirinus* | 006735 | male | CAS | USA, California, Nevada County, near Hobart Mills, Sagehen Creek, [39.407, -120.19], 1.VII.1964, C. N. Slobodchikoff |
| *Eulonchus sapphirinus* | 006736 | male | CAS | USA, California, Sierra County, Gold Lake, [39.678, -120.657], 12.VII.1921, C. L. Fox |
| *Eulonchus sapphirinus* | 006737 | male | CAS | USA, California, Nevada County, near Hobart Mills, Sagehen Creek, [39.407, -120.19], 1.VII.1964, C. N. Slobodchikoff |
| *Eulonchus sapphirinus* | 006738 | male | CAS | USA, California, Lassen National Park, Kelly’s Ranch, 14.VI.1931, E. C. Van Dyke |
| *Eulonchus sapphirinus* | 006739 | male | CAS | USA, California, Nevada County, near Hobart Mills, Sagehen Creek, [39.407, -120.19], 1.VII.1964, C. N. Slobodchikoff |
| *Eulonchus sapphirinus* | 006740 | male | CAS | USA, California, Sierra County, Gold Lake, [39.678, -120.657], 12.VII.1921 |
| *Eulonchus sapphirinus* | 006741 | male | CAS | USA, California, Plumas County, Blairsden, [39.781, -120.617], 4.VI.1958, W. R. Bauer, J. S. Buckett |
| *Eulonchus sapphirinus* | 006742 | female | CAS | USA, California, Plumas County, Blairsden, [39.781, -120.617], 4.VI.1958, W. R. Bauer, J. S. Buckett |
| *Eulonchus sapphirinus* | 006743 | female | CAS | USA, California, Plumas County, near La Porte, Table Mountain, [39.773, -121.077], 1707 to 1859 m, 30.VI.1963, E. E. Ball, Jr. |
| *Eulonchus sapphirinus* | 008335 | female | CAS | USA, California, Plumas County, Buck’s Lake, [39.868, -121.174], 1.VII.1949, J. W. MacSwain |
| *Eulonchus sapphirinus* | 008717 | unknown | CAS | USA, Oregon, Benton County, nr. Adair Village, [44.671, -123.227], 26.VI.1977, R. L. Westcott |
| *Eulonchus sapphirinus* | 008734 | unknown | CAS | USA, Oregon, Linn County, 9.7 km. SE. of Gates, [44.698, -122.326], 3.VII.1971, R. L. Westcott |
| *Eulonchus sapphirinus* | 008757 | unknown | CAS | USA, Oregon, Hood River County, 6.8 km. S. of Mt. Hood P.O., [45.313, -121.697], 13.VII.1971, R. L. Westcott |
| *Eulonchus sapphirinus* | 008758 | unknown | CAS | USA, Oregon, Hood River County, 6.8 km. S. of Mt. Hood P.O., [45.313, -121.697], 13.VII.1971, R. L. Westcott |
| *Eulonchus sapphirinus* | 008760 | unknown | CAS | USA, Oregon, Yamhill County, 14.5 km NW of MacMinnville, [45.288, -123.347], 27.V.1972, R. L. Westcott |
| *Eulonchus sapphirinus* | 008763 | unknown | CAS | USA, Oregon, Linn County, 1.6 km S of Marion Forks, [44.601, -121.946], 21.VII.1969, K. Goeden |
| *Eulonchus sapphirinus* | 008771 | unknown | CAS | USA, Washington, Olympic National Park, 11.3 - 16.1 km. S of Heart-O’-the-Hills, [47.846, -123.502], 10.VIII.1970, R. L. Westcott |
| *Eulonchus sapphirinus* | 008772 | unknown | CAS | USA, Washington, Olympic National Park, 11.3 - 16.1 km. S of Heart-O’-the-Hills, [47.846, -123.502], 10.VIII.1970, R. L. Westcott |
| *Eulonchus sapphirinus* | 008903 | unknown | CAS | USA, Utah, Bow Elder County, Mantua, Devil’s Gate, [41.45, -111.92], 12.VI.1969, T. L. Whitworth |
| *Eulonchus sapphirinus* | 008904 | unknown | CAS | USA, Utah, Bow Elder County, Mantua, Devil’s Gate, [41.45, -111.92], 9.VI.1969, T. L. Whitworth |
| *Eulonchus sapphirinus* | 008905 | unknown | CAS | USA, Utah, Bow Elder County, Mantua, Devil’s Gate, [41.45, -111.92], 9.VI.1969, T. L. Whitworth |
| *Eulonchus sapphirinus* | 008908 | unknown | CAS | USA, Utah, Piute County, 16.1 km W Junction, [38.238, -112.404], 23.VI.1971, G. Bohart, P. Torchio |
| *Eulonchus sapphirinus* | 008910 | unknown | CAS | USA, Utah, Cache County, Logan, [41.737, -111.834], 3.VI.1974, Parker |
| *Eulonchus sapphirinus* | 008911 | unknown | CAS | USA, Utah, Cache County, Logan, [41.737, -111.834], 3.VI.1974, Parker |
| *Eulonchus sapphirinus* | 008912 | unknown | CAS | USA, Utah, Piute County, 16.1 km W Junction, [38.238, -112.404], 23.VI.1971, G. Bohart, P. Torchio |
| *Eulonchus sapphirinus* | 008913 | unknown | CAS | USA, Utah, Weber County, South of Monte Crisco, 2.VI.1979, G. F. Knowlton |
| *Eulonchus sapphirinus* | 008914 | unknown | CAS | USA, California, Siskiyou County, McCloud, [41.256, -122.14], 14.VI.1961, S. L. Wood, J. B. Karren, D.E. Bright |
| *Eulonchus sapphirinus* | 008915 | unknown | CAS | USA, Utah, Washington County, Pine Valley, [37.391, -113.514], 14.VI.1961, D. Davis |
| *Eulonchus sapphirinus* | 008920 | unknown | CAS | USA, Utah, Piute County, 16.1 km W Junction, [38.238, -112.404], 23.VI.1971, G. Bohart, P. Torchio |
| *Eulonchus sapphirinus* | 008923 | unknown | CAS | USA, California, Siskiyou County, McCloud, [41.256, -122.14], 14.VI.1961, S. L. Wood, J. B. Karren, D.E. Bright |
| *Eulonchus sapphirinus* | 008924 | unknown | CAS | USA, Utah, Cache County, Logan, Tony Grove, [41.896, -111.554], 3.VII.1974 |
| *Eulonchus sapphirinus* | 008925 | unknown | CAS | USA, Utah, Cache County, USU School Forest, [41.748, -111.808], 15.VI.1977 |
| *Eulonchus sapphirinus* | 008927 | unknown | CAS | USA, California, Siskiyou County, McCloud, [41.256, -122.14], 14.VI.1961, S. L. Wood, J. B. Karren, D.E. Bright |
| *Eulonchus sapphirinus* | 008928 | unknown | CAS | USA, California, Siskiyou County, McCloud, [41.256, -122.14], 14.VI.1961, S. L. Wood, J. B. Karren, D.E. Bright |
| *Eulonchus sapphirinus* | 008930 | unknown | CAS | USA, Washington, Pierce County, Tacoma, [47.253, -122.445], 15.VII.1976, T. L. Whitworth |
| *Eulonchus sapphirinus* | 008931 | unknown | CAS | USA, Washington, Pierce County, Tacoma, [47.253, -122.445], 15.VII.1976, T. L. Whitworth |
| *Eulonchus sapphirinus* | 008932 | unknown | CAS | USA, Washington, Pierce County, Tacoma, [47.253, -122.445], 15.VII.1976, T. L. Whitworth |
| *Eulonchus sapphirinus* | 008933 | unknown | CAS | USA, Washington, Pierce County, Tacoma, [47.253, -122.445], 15.VII.1976, T. L. Whitworth |
| *Eulonchus sapphirinus* | 008934 | unknown | CAS | USA, Idaho, Oneida County, Black Pine Canyon, [42.021, -113.093], 1768 m, 12.VI.1974 to 25.VI.1974, malaise trap, |
| *Eulonchus sapphirinus* | 008935 | unknown | CAS | USA, Idaho, Oneida County, Black Pine Canyon, [42.021, -113.093], 1768 m, 4.VI.1974 to 12.VI.1974, malaise trap, |
| *Eulonchus sapphirinus* | 008936 | unknown | CAS | USA, Idaho, Oneida County, Black Pine Canyon, [42.021, -113.093], 1768 m, 25.VI.1974 to 5.VII.1974, malaise trap, |
| *Eulonchus sapphirinus* | 008937 | unknown | CAS | USA, Washington, Pierce County, Tacoma, [47.253, -122.445], 15.VII.1976, T. L. Whitworth |
| *Eulonchus sapphirinus* | 008938 | unknown | CAS | USA, Washington, Pierce County, Tacoma, [47.253, -122.445], 15.VII.1976, T. L. Whitworth |
| *Eulonchus sapphirinus* | 008939 | unknown | CAS | USA, Washington, Pierce County, Tacoma, [47.253, -122.445], 15.VII.1976, T. L. Whitworth |
| *Eulonchus sapphirinus* | 008940 | unknown | CAS | USA, Washington, Pierce County, Tacoma, [47.253, -122.445], 15.VII.1976, T. L. Whitworth |
| *Eulonchus sapphirinus* | 008941 | unknown | CAS | USA, Washington, Pierce County, Tacoma, [47.253, -122.445], 15.VII.1976, T. L. Whitworth |
| *Eulonchus sapphirinus* | 008942 | unknown | CAS | USA, Washington, Pierce County, Tacoma, [47.253, -122.445], 15.VII.1976, T. L. Whitworth |
| *Eulonchus sapphirinus* | 008943 | unknown | CAS | USA, Washington, Pierce County, Tacoma, [47.253, -122.445], 15.VII.1976, T. L. Whitworth |
| *Eulonchus sapphirinus* | 008948 | unknown | CAS | USA, Utah, Cache County, Logan, Tony Grove, [41.896, -111.554], 12.VII.1972, D. Vincent, F. Parker |
| *Eulonchus sapphirinus* | 008949 | unknown | CAS | USA, Idaho, Oneida County, Black Pine Canyon, [42.021, -113.093], 1768 m, 4.VI.1974 to 12.VI.1974, malaise trap, |
| *Eulonchus sapphirinus* | 008950 | unknown | CAS | USA, Idaho, Oneida County, Black Pine Canyon, [42.021, -113.093], 1768 m, 4.VI.1974 to 12.VI.1974, malaise trap, |
| *Eulonchus sapphirinus* | 008952 | unknown | CAS | USA, Idaho, Oneida County, Black Pine Canyon, [42.021, -113.093], 1768 m, 25.VI.1974 to 5.VII.1974, malaise trap, |
| *Eulonchus sapphirinus* | 008975 | unknown | CAS | USA, Utah, Cache County, Logan, Grass Canyon, [41.737, -111.834], 15.VI.1957 |
| *Eulonchus sapphirinus* | 008976 | unknown | UCDC | USA, Utah, Cache County, Providence Canyon, [41.693, -111.796], 2438 m, 20.VI.1969, G. E. Bohart |
| *Eulonchus sapphirinus* | 009074 | unknown | CAS | USA, California, Mendocino County, NCCRP (Northern California Coast Range Preserve), 4.8 km N of Branscomb, [39.697, -123.626], 427 m, time of day: midafternoon, 21.V.1982 to 23.V.1982, E. I. Schlinger |
| *Eulonchus sapphirinus* | 009076 | unknown | CAS | USA, California, Mendocino County, NCCRP (Northern California Coast Range Preserve), 4.8 km N of Branscomb, [39.697, -123.626], 427 m, time of day: midafternoon, 21.V.1982 to 23.V.1982, E. I. Schlinger |
| *Eulonchus sapphirinus* | 009078 | unknown | CAS | USA, California, Mendocino County, NCCRP (Northern California Coast Range Preserve), 4.8 km N of Branscomb, [39.697, -123.626], 427 m, time of day: midafternoon, 21.V.1982 to 23.V.1982, E. I. Schlinger |
| *Eulonchus sapphirinus* | 009079 | unknown | CAS | USA, California, Mendocino County, NCCRP (Northern California Coast Range Preserve), 4.8 km N of Branscomb, [39.697, -123.626], 427 m, time of day: midafternoon, 21.V.1982 to 23.V.1982, E. I. Schlinger |
| *Eulonchus sapphirinus* | 009080 | unknown | CAS | USA, California, Mendocino County, NCCRP (Northern California Coast Range Preserve), 4.8 km N of Branscomb, [39.697, -123.626], 427 m, time of day: midafternoon, 21.V.1982 to 23.V.1982, E. I. Schlinger |
| *Eulonchus sapphirinus* | 009082 | unknown | CAS | USA, California, Mendocino County, NCCRP (Northern California Coast Range Preserve), 4.8 km N of Branscomb, [39.697, -123.626], 427 m, time of day: midafternoon, 21.V.1982 to 23.V.1982, E. I. Schlinger |
| *Eulonchus sapphirinus* | 009325 | unknown | CAS | USA, California, Santa Clara County, San Antonio Valley, [37.355, -121.92], 3.VI.1954, J. G. Rozen |
| *Eulonchus sapphirinus* | 009338 | unknown | CAS | USA, California, El Dorado County, Blodgett Forest Research Station, 20.9 km east of Georgetown, [38.909, -120.661], 1300 m, 29.VI.1967, W. Turner |
| *Eulonchus sapphirinus* | 009339 | unknown | CAS | USA, California, El Dorado County, Blodgett Forest Research Station, 20.9 km east of Georgetown, [38.909, -120.661], 1300 m, 29.VI.1967, W. Turner |
| *Eulonchus sapphirinus* | 009340 | unknown | CAS | USA, California, El Dorado County, Blodgett Forest Research Station, 20.9 km east of Georgetown, [38.909, -120.661], 1300 m, 29.VI.1967, W. Turner |
| *Eulonchus sapphirinus* | 009341 | unknown | CAS | USA, California, El Dorado County, Blodgett Forest Research Station, 20.9 km east of Georgetown, [38.909, -120.661], 1300 m, 29.VI.1967, W. Turner |
| *Eulonchus sapphirinus* | 009342 | unknown | CAS | USA, California, El Dorado County, Blodgett Forest Research Station, 20.9 km east of Georgetown, [38.909, -120.661], 1300 m, 29.VI.1967, W. Turner |
| *Eulonchus sapphirinus* | 009343 | unknown | CAS | USA, California, El Dorado County, Blodgett Forest Research Station, 20.9 km east of Georgetown, [38.909, -120.661], 1300 m, 19.VI.1967, J. Powell |
| *Eulonchus sapphirinus* | 009344 | unknown | CAS | USA, California, El Dorado County, Blodgett Forest Research Station, 20.9 km east of Georgetown, [38.909, -120.661], 1300 m, 24.VI.1967, S.R. Kutcher |
| *Eulonchus sapphirinus* | 009345 | unknown | CAS | USA, California, El Dorado County, Blodgett Forest Research Station, 20.9 km east of Georgetown, [38.909, -120.661], 1300 m, 24.VI.1967, M.O. Way |
| *Eulonchus sapphirinus* | 009346 | unknown | CAS | USA, California, El Dorado County, Blodgett Forest Research Station, 20.9 km east of Georgetown, [38.909, -120.661], 1300 m, 30.VI.1967, W. Turner |
| *Eulonchus sapphirinus* | 009347 | unknown | CAS | USA, California, El Dorado County, Blodgett Forest Research Station, 20.9 km east of Georgetown, [38.909, -120.661], 1300 m, 30.VI.1967, W. Turner |
| *Eulonchus sapphirinus* | 009348 | unknown | CAS | USA, California, El Dorado County, Blodgett Forest Research Station, 20.9 km east of Georgetown, [38.909, -120.661], 1300 m, 30.VI.1967, W. Turner |
| *Eulonchus sapphirinus* | 009349 | unknown | CAS | USA, California, El Dorado County, Blodgett Forest Research Station, 20.9 km east of Georgetown, [38.909, -120.661], 1300 m, 30.VI.1967, W. Turner |
| *Eulonchus sapphirinus* | 009350 | unknown | CAS | USA, California, El Dorado County, Blodgett Forest Research Station, 20.9 km east of Georgetown, [38.909, -120.661], 1300 m, 30.VI.1967, W. Turner |
| *Eulonchus sapphirinus* | 009351 | unknown | CAS | USA, California, El Dorado County, Blodgett Forest Research Station, 20.9 km east of Georgetown, [38.909, -120.661], 1300 m, 30.VI.1967, W. Turner |
| *Eulonchus sapphirinus* | 009352 | unknown | CAS | USA, California, El Dorado County, Blodgett Forest Research Station, 20.9 km east of Georgetown, [38.909, -120.661], 1300 m, 30.VI.1967, W. Turner |
| *Eulonchus sapphirinus* | 009353 | unknown | CAS | USA, California, El Dorado County, Blodgett Forest Research Station, 20.9 km east of Georgetown, [38.909, -120.661], 1300 m, 30.VI.1967, W. Turner |
| *Eulonchus sapphirinus* | 009354 | unknown | CAS | USA, California, El Dorado County, Blodgett Forest Research Station, 20.9 km east of Georgetown, [38.909, -120.661], 1300 m, 30.VI.1967, W. Turner |
| *Eulonchus sapphirinus* | 009355 | unknown | CAS | USA, California, El Dorado County, Blodgett Forest Research Station, 20.9 km east of Georgetown, [38.909, -120.661], 1300 m, 30.VI.1967, W. Turner |
| *Eulonchus sapphirinus* | 009356 | unknown | CAS | USA, California, El Dorado County, Blodgett Forest Research Station, 20.9 km east of Georgetown, [38.909, -120.661], 1300 m, 30.VI.1967, W. Turner |
| *Eulonchus sapphirinus* | 009357 | unknown | CAS | USA, California, El Dorado County, Blodgett Forest Research Station, 20.9 km east of Georgetown, [38.909, -120.661], 1300 m, 30.VI.1967, W. Turner |
| *Eulonchus sapphirinus* | 009358 | unknown | CAS | USA, California, El Dorado County, Blodgett Forest Research Station, 20.9 km east of Georgetown, [38.909, -120.661], 1300 m, 30.VI.1967, W. Turner |
| *Eulonchus sapphirinus* | 009359 | unknown | CAS | USA, California, El Dorado County, Blodgett Forest Research Station, 20.9 km east of Georgetown, [38.909, -120.661], 1300 m, 30.VI.1967, W. Turner |
| *Eulonchus sapphirinus* | 009360 | unknown | CAS | USA, California, El Dorado County, Blodgett Forest Research Station, 20.9 km east of Georgetown, [38.909, -120.661], 1300 m, 30.VI.1967, W. Turner |
| *Eulonchus sapphirinus* | 009361 | unknown | CAS | USA, California, El Dorado County, Blodgett Forest Research Station, 20.9 km east of Georgetown, [38.909, -120.661], 1300 m, 30.VI.1967, W. Turner |
| *Eulonchus sapphirinus* | 009362 | unknown | CAS | USA, California, El Dorado County, Blodgett Forest Research Station, 20.9 km east of Georgetown, [38.909, -120.661], 1300 m, 30.VI.1967, W. Turner |
| *Eulonchus sapphirinus* | 009363 | unknown | CAS | USA, California, El Dorado County, Blodgett Forest Research Station, 20.9 km east of Georgetown, [38.909, -120.661], 1300 m, 30.VI.1967, W. Turner |
| *Eulonchus sapphirinus* | 009364 | unknown | CAS | USA, California, El Dorado County, Blodgett Forest Research Station, 20.9 km east of Georgetown, [38.909, -120.661], 1300 m, 30.VI.1967, W. Turner |
| *Eulonchus sapphirinus* | 009365 | unknown | CAS | USA, California, El Dorado County, Blodgett Forest Research Station, 20.9 km east of Georgetown, [38.909, -120.661], 1300 m, 30.VI.1967, W. Turner |
| *Eulonchus sapphirinus* | 009366 | unknown | CAS | USA, California, El Dorado County, Blodgett Forest Research Station, 20.9 km east of Georgetown, [38.909, -120.661], 1300 m, 30.VI.1967, W. Turner |
| *Eulonchus sapphirinus* | 009367 | unknown | CAS | USA, California, El Dorado County, Blodgett Forest Research Station, 20.9 km east of Georgetown, [38.909, -120.661], 1300 m, 29.VI.1967, W. Turner |
| *Eulonchus sapphirinus* | 009368 | unknown | CAS | USA, California, El Dorado County, Blodgett Forest Research Station, 20.9 km east of Georgetown, [38.909, -120.661], 1300 m, 29.VI.1967, W. Turner |
| *Eulonchus sapphirinus* | 009369 | unknown | CAS | USA, California, El Dorado County, Blodgett Forest Research Station, 20.9 km east of Georgetown, [38.909, -120.661], 1300 m, 29.VI.1967, W. Turner |
| *Eulonchus sapphirinus* | 009370 | unknown | CAS | USA, California, El Dorado County, Blodgett Forest Research Station, 20.9 km east of Georgetown, [38.909, -120.661], 1300 m, 29.VI.1967, W. Turner |
| *Eulonchus sapphirinus* | 009371 | unknown | CAS | USA, California, El Dorado County, Blodgett Forest Research Station, 20.9 km east of Georgetown, [38.909, -120.661], 1300 m, 29.VI.1967, W. Turner |
| *Eulonchus sapphirinus* | 009372 | unknown | CAS | USA, California, El Dorado County, Blodgett Forest Research Station, 20.9 km east of Georgetown, [38.909, -120.661], 1300 m, 29.VI.1967, W. Turner |
| *Eulonchus sapphirinus* | 009373 | unknown | CAS | USA, California, El Dorado County, Blodgett Forest Research Station, 20.9 km east of Georgetown, [38.909, -120.661], 1300 m, 29.VI.1967, W. Turner |
| *Eulonchus sapphirinus* | 009374 | unknown | CAS | USA, California, El Dorado County, Blodgett Forest Research Station, 20.9 km east of Georgetown, [38.909, -120.661], 1300 m, 29.VI.1967, W. Turner |
| *Eulonchus sapphirinus* | 009375 | unknown | CAS | USA, California, El Dorado County, Blodgett Forest Research Station, 20.9 km east of Georgetown, [38.909, -120.661], 1300 m, 29.VI.1967, W. Turner |
| *Eulonchus sapphirinus* | 009376 | unknown | CAS | USA, California, El Dorado County, Blodgett Forest Research Station, 20.9 km east of Georgetown, [38.909, -120.661], 1300 m, 29.VI.1967, W. Turner |
| *Eulonchus sapphirinus* | 009377 | unknown | CAS | USA, California, El Dorado County, Blodgett Forest Research Station, 20.9 km east of Georgetown, [38.909, -120.661], 1300 m, 29.VI.1967, W. Turner |
| *Eulonchus sapphirinus* | 009378 | unknown | CAS | USA, California, El Dorado County, Blodgett Forest Research Station, 20.9 km east of Georgetown, [38.909, -120.661], 1300 m, 29.VI.1967, W. Turner |
| *Eulonchus sapphirinus* | 009379 | unknown | CAS | USA, California, El Dorado County, Blodgett Forest Research Station, 20.9 km east of Georgetown, [38.909, -120.661], 1300 m, 29.VI.1967, W. Turner |
| *Eulonchus sapphirinus* | 009380 | unknown | CAS | USA, California, El Dorado County, Blodgett Forest Research Station, 20.9 km east of Georgetown, [38.909, -120.661], 1300 m, 29.VI.1967, W. Turner |
| *Eulonchus sapphirinus* | 009381 | unknown | CAS | USA, California, El Dorado County, Blodgett Forest Research Station, 20.9 km east of Georgetown, [38.909, -120.661], 1300 m, 29.VI.1967, W. Turner |
| *Eulonchus sapphirinus* | 009382 | unknown | CAS | USA, California, El Dorado County, Blodgett Forest Research Station, 20.9 km east of Georgetown, [38.909, -120.661], 1300 m, 30.VI.1967, W. Turner |
| *Eulonchus sapphirinus* | 009394 | unknown | CAS | USA, Washington, Chinook Pass, [46.872, -121.516], 29.VII.1949, L. D. Beamer |
| *Eulonchus sapphirinus* | 009395 | unknown | CAS | USA, Utah, Summit County, Park City, [40.647, -111.498], 11.VI.1952, C.H. Winer |
| *Eulonchus sapphirinus* | 009396 | unknown | CAS | USA, Washington, Packwood, [46.607, -121.671], 22.VII.1949, L. D. Beamer |
| *Eulonchus sapphirinus* | 009397 | unknown | CAS | USA, Washington, American River, 29.VII.1949, L. D. Beamer |
| *Eulonchus sapphirinus* | 009415 | unknown | CAS | USA, California, Tehama-Butte County Line, Highway 32, [40.175, -121.552], 21.VI.1959, R. W. Thorp |
| *Eulonchus sapphirinus* | 009416 | unknown | CAS | USA, California, Tehama-Butte County Line, Highway 32, [40.175, -121.552], 21.VI.1959, R. W. Thorp |
| *Eulonchus sapphirinus* | 009417 | unknown | CAS | USA, Oregon, Josephine County, N. Fork Dunn Creek, Siskiyou Springs, [42.001, -123.538], 24.VII.1969, J. Powell |
| *Eulonchus sapphirinus* | 009418 | unknown | CAS | USA, Oregon, Josephine County, N. Fork Dunn Creek, Siskiyou Springs, [42.001, -123.538], 24.VII.1969, J. Powell |
| *Eulonchus sapphirinus* | 009419 | unknown | CAS | USA, California, Mendocino County, Mouth of Gualala River, [38.768, -123.514], 30.V.1958, E. G. Linsley |
| *Eulonchus sapphirinus* | 009420 | unknown | CAS | USA, California, Marin County, Mill Valley, =, [37.906, -122.545], 28.IV.1959, C. W. O’Brien |
| *Eulonchus sapphirinus* | 009421 | unknown | CAS | USA, California, Marin County, Mill Valley, =, [37.906, -122.545], 28.IV.1959, C. W. O’Brien |
| *Eulonchus sapphirinus* | 009424 | unknown | CAS | USA, California, Nevada County, Sagehen near Hobart Mills, [39.434, -120.248], 25.VI.1954, J. A. Powell |
| *Eulonchus sapphirinus* | 009425 | unknown | CAS | USA, California, Siskiyou County, 1.6 km. NW of Bartle, [41.272, -121.842], 20.VII.1966, Opler, P. A. |
| *Eulonchus sapphirinus* | 009426 | unknown | CAS | USA, California, Siskiyou County, 1.6 km. NW of Bartle, [41.272, -121.842], 20.VII.1966, Opler, P. A. |
| *Eulonchus sapphirinus* | 009427 | unknown | CAS | USA, California, Siskiyou County, 1.6 km. NW of Bartle, [41.272, -121.842], 20.VII.1966, Opler, P. A. |
| *Eulonchus sapphirinus* | 009429 | unknown | CAS | USA, California, Siskiyou County, 1.6 km. NW of Bartle, [41.272, -121.842], 20.VII.1966, Opler, P. A. |
| *Eulonchus sapphirinus* | 009430 | unknown | CAS | USA, California, Siskiyou County, 1.6 km. NW of Bartle, [41.272, -121.842], 20.VII.1966, P. Rude |
| *Eulonchus sapphirinus* | 009432 | unknown | CAS | USA, California, Lassen County, 45.1 km E of Shasta, Hwy 44, [41.407, -121.656], 3.VII.1967, J. Wilcox |
| *Eulonchus sapphirinus* | 009433 | unknown | CAS | USA, California, Trinity County, 16.1 km North of Coffee Creek Ranger Station, [41.233, -122.706], 15.VII.1955, J. W. MacSwain |
| *Eulonchus sapphirinus* | 009434 | unknown | CAS | USA, California, Trinity County, 16.1 km North of Coffee Creek Ranger Station, [41.233, -122.706], 15.VII.1955, J. W. MacSwain |
| *Eulonchus sapphirinus* | 009435 | unknown | CAS | USA, California, Trinity County, 16.1 km North of Coffee Creek Ranger Station, [41.233, -122.706], 15.VII.1955, J. W. MacSwain |
| *Eulonchus sapphirinus* | 009436 | unknown | CAS | USA, California, Trinity County, 16.1 km North of Coffee Creek Ranger Station, [41.233, -122.706], 15.VII.1955, J. W. MacSwain |
| *Eulonchus sapphirinus* | 009437 | unknown | CAS | USA, California, Trinity County, 16.1 km North of Coffee Creek Ranger Station, [41.233, -122.706], 15.VII.1955, J. W. MacSwain |
| *Eulonchus sapphirinus* | 010248 | unknown | CAS | USA, Oregon, Linn County, 32.2 km E. of Cascadia, [44.397, -122.082], 25.VI.1954, E. I. Schlinger |
| *Eulonchus sapphirinus* | 010249 | unknown | CAS | USA, Oregon, Linn County, 32.2 km E. of Cascadia, [44.397, -122.082], 25.VI.1954, E. I. Schlinger |
| *Eulonchus sapphirinus* | 010250 | unknown | CAS | USA, Oregon, Linn County, 32.2 km E. of Cascadia, [44.397, -122.082], 25.VI.1954, E. I. Schlinger |
| *Eulonchus sapphirinus* | 010251 | unknown | CAS | USA, Oregon, Linn County, 32.2 km E. of Cascadia, [44.397, -122.082], 25.VI.1954, E. I. Schlinger |
| *Eulonchus sapphirinus* | 010252 | unknown | CAS | USA, Oregon, Linn County, 32.2 km E. of Cascadia, [44.397, -122.082], 25.VI.1954, E. I. Schlinger |
| *Eulonchus sapphirinus* | 010253 | unknown | CAS | USA, Oregon, Linn County, 32.2 km E. of Cascadia, [44.397, -122.082], 25.VI.1954, E. I. Schlinger |
| *Eulonchus sapphirinus* | 010254 | unknown | CAS | USA, Oregon, Linn County, 32.2 km E. of Cascadia, [44.397, -122.082], 25.VI.1954, E. I. Schlinger |
| *Eulonchus sapphirinus* | 010255 | unknown | CAS | USA, Oregon, Linn County, 32.2 km E. of Cascadia, [44.397, -122.082], 25.VI.1954, E. I. Schlinger |
| *Eulonchus sapphirinus* | 010256 | unknown | CAS | USA, Oregon, Linn County, 32.2 km E. of Cascadia, [44.397, -122.082], 25.VI.1954, E. I. Schlinger |
| *Eulonchus sapphirinus* | 010257 | unknown | CAS | USA, Oregon, Linn County, 32.2 km E. of Cascadia, [44.397, -122.082], 25.VI.1954, E. I. Schlinger |
| *Eulonchus sapphirinus* | 010258 | unknown | CAS | USA, Oregon, Linn County, 32.2 km E. of Cascadia, [44.397, -122.082], 25.VI.1954, E. I. Schlinger |
| *Eulonchus sapphirinus* | 010259 | unknown | CAS | USA, Oregon, Linn County, 32.2 km E. of Cascadia, [44.397, -122.082], 25.VI.1954, J. C. Downey |
| *Eulonchus sapphirinus* | 010260 | unknown | CAS | USA, Oregon, Linn County, 32.2 km E. of Cascadia, [44.397, -122.082], 25.VI.1954, J. C. Downey |
| *Eulonchus sapphirinus* | 010261 | unknown | CAS | USA, Oregon, Linn County, 32.2 km E. of Cascadia, [44.397, -122.082], 25.VI.1954, J. C. Downey |
| *Eulonchus sapphirinus* | 010262 | unknown | CAS | USA, Oregon, Linn County, 32.2 km E. of Cascadia, [44.397, -122.082], 25.VI.1954, E. I. Schlinger |
| *Eulonchus sapphirinus* | 010263 | unknown | CAS | USA, Oregon, Linn County, 32.2 km E. of Cascadia, [44.397, -122.082], 25.VI.1954, E. I. Schlinger |
| *Eulonchus sapphirinus* | 010264 | unknown | CAS | USA, Oregon, Linn County, 32.2 km E. of Cascadia, [44.397, -122.082], 25.VI.1954, E. I. Schlinger |
| *Eulonchus sapphirinus* | 010265 | unknown | CAS | USA, Oregon, Linn County, 32.2 km E. of Cascadia, [44.397, -122.082], 25.VI.1954, A. A. Grigarick |
| *Eulonchus sapphirinus* | 010266 | unknown | CAS | USA, Oregon, Linn County, 32.2 km E. of Cascadia, [44.397, -122.082], 25.VI.1954, A. A. Grigarick |
| *Eulonchus sapphirinus* | 010267 | unknown | CAS | USA, Oregon, Linn County, 32.2 km E. of Cascadia, [44.397, -122.082], 25.VI.1954, A. A. Grigarick |
| *Eulonchus sapphirinus* | 010268 | unknown | CAS | USA, Oregon, Linn County, 32.2 km E. of Cascadia, [44.397, -122.082], 25.VI.1954, A. A. Grigarick |
| *Eulonchus sapphirinus* | 010269 | unknown | CAS | USA, Oregon, Linn County, 32.2 km E. of Cascadia, [44.397, -122.082], 25.VI.1954, A. A. Grigarick |
| *Eulonchus sapphirinus* | 010270 | female | CAS | USA, Oregon, Linn County, 32.2 km E. of Cascadia, [44.397, -122.082], 25.VI.1954, A. A. Grigarick |
| *Eulonchus sapphirinus* | 010271 | unknown | CAS | USA, Oregon, Linn County, 32.2 km E. of Cascadia, [44.397, -122.082], 25.VI.1954, A. A. Grigarick |
| *Eulonchus sapphirinus* | 010272 | unknown | CAS | USA, Oregon, Linn County, 32.2 km E. of Cascadia, [44.397, -122.082], 25.VI.1954, A. A. Grigarick |
| *Eulonchus sapphirinus* | 010298 | unknown | OSUC | USA, Oregon, Marion County, Breitenbush Spring, [44.781, -121.975], 14.VI.1942, R. E. Rieder |
| *Eulonchus sapphirinus* | 010299 | unknown | OSUC | USA, Oregon, Marion County, Breitenbush Spring, [44.781, -121.975], 14.VI.1942, R. E. Rieder |
| *Eulonchus sapphirinus* | 010300 | unknown | OSUC | USA, Oregon, Marion County, Breitenbush Spring, [44.781, -121.975], 14.VI.1942, R. E. Rieder |
| *Eulonchus sapphirinus* | 010301 | unknown | OSUC | USA, Oregon, Marion County, Breitenbush Spring, [44.781, -121.975], 14.VI.1942, R. E. Rieder |
| *Eulonchus sapphirinus* | 010302 | unknown | OSUC | USA, Oregon, Marion County, Breitenbush Spring, [44.781, -121.975], 14.VI.1942, R. E. Rieder |
| *Eulonchus sapphirinus* | 010322 | unknown | OSUC | USA, California, Mendocino County, Van Damme State Park, 18.V.1947 |
| *Eulonchus sapphirinus* | 010323 | unknown | OSUC | USA, California, Mendocino County, Van Damme State Park, 18.V.1947 |
| *Eulonchus sapphirinus* | 010324 | unknown | OSUC | USA, California, Mendocino County, Van Damme State Park, 18.V.1947 |
| *Eulonchus sapphirinus* | 010325 | unknown | OSUC | USA, California, Mendocino County, Van Damme State Park, 18.V.1947 |
| *Eulonchus sapphirinus* | 010333 | unknown | CAS | USA, Oregon, Columbia River, Starvation Creek State Park, [45.688, -121.691], 17.VI.1957, E. I. Schlinger |
| *Eulonchus sapphirinus* | 010334 | unknown | CAS | USA, Oregon, Columbia River, Starvation Creek State Park, [45.688, -121.691], 17.VI.1957, E. I. Schlinger |
| *Eulonchus sapphirinus* | 010379 | unknown | OSUC | USA, California, Humboldt County, Korbel - Maple Creek, 24.VI.1907, J. C. Bradley |
| *Eulonchus sapphirinus* | 010380 | unknown | OSUC | USA, California, Humboldt County, Korbel - Maple Creek, 24.VI.1907, J. C. Bradley |
| *Eulonchus sapphirinus* | 010406 | unknown | CAS | USA, Oregon, Linn County, Monument Peak, [44.695, -122.322], 1372 m, 2.VIII.1953, P.O. Ritcher |
| *Eulonchus sapphirinus* | 010407 | unknown | CAS | USA, Oregon, Linn County, Monument Peak, [44.695, -122.322], 1372 m, 2.VIII.1953, P.O. Ritcher |
| *Eulonchus sapphirinus* | 010408 | unknown | CAS | USA, Oregon, Linn County, Monument Peak, [44.695, -122.322], 1372 m, 2.VIII.1953, P.O. Ritcher |
| *Eulonchus sapphirinus* | 010409 | unknown | CAS | USA, Oregon, Linn County, Monument Peak, [44.695, -122.322], 1372 m, 2.VIII.1953, P.O. Ritcher |
| *Eulonchus sapphirinus* | 010410 | unknown | CAS | USA, Oregon, Linn County, Monument Peak, [44.695, -122.322], 1372 m, 2.VIII.1953, P.O. Ritcher |
| *Eulonchus sapphirinus* | 010411 | unknown | OSUC | USA, Oregon, Clackamas County, 9.7 km SE of Oak Grove Ranger Station, Shellrock Campground, [45.029, -121.932], 671 m, 19.VI.1955, M. T. James |
| *Eulonchus sapphirinus* | 010412 | unknown | OSUC | USA, Oregon, Clackamas County, 9.7 km SE of Oak Grove Ranger Station, Shellrock Campground, [45.029, -121.932], 671 m, 19.VI.1955, M. T. James |
| *Eulonchus sapphirinus* | 010413 | unknown | OSUC | USA, Oregon, Clackamas County, 9.7 km SE of Oak Grove Ranger Station, Shellrock Campground, [45.029, -121.932], 671 m, 19.VI.1955, M. T. James |
| *Eulonchus sapphirinus* | 011742 | unknown | CAS | USA, Oregon, Jefferson Co., Mt. Jefferson, Permelia L., [44.674, -121.799], 914 m, 16.VII.1907, J.C. Bridwell |
| *Eulonchus sapphirinus* | 011752 | unknown | CAS | USA, Oregon, Calacamas Co., Mt. Hood, [45.373, -121.696], .VII.1929, F. J. Spruijt |
| *Eulonchus sapphirinus* | 011789 | unknown | CAS | USA, Oregon, Grant County, Field’s Peak, [44.339, -119.258], .VII.1941, H. E. Cott |
| *Eulonchus sapphirinus* | 011887 | unknown | CAS | USA, California, Nevada County, 5 miles south of Washington, [39.287, -120.801], 31.V.1977, E. I. Schlinger |
| *Eulonchus sapphirinus* | 011888 | unknown | CAS | USA, California, Nevada County, 5 miles south of Washington, [39.287, -120.801], 31.V.1977, E. I. Schlinger |
| *Eulonchus sapphirinus* | 011889 | unknown | CAS | USA, California, Nevada County, 5 miles south of Washington, [39.287, -120.801], 31.V.1977, E. I. Schlinger |
| *Eulonchus sapphirinus* | 011890 | unknown | CAS | USA, California, Nevada County, 5 miles south of Washington, [39.287, -120.801], 31.V.1977, E. I. Schlinger |
| *Eulonchus sapphirinus* | 011891 | unknown | CAS | USA, California, Nevada County, 5 miles south of Washington, [39.287, -120.801], 31.V.1977, E. I. Schlinger |
| *Eulonchus sapphirinus* | 011892 | unknown | CAS | USA, California, Nevada County, 5 miles south of Washington, [39.287, -120.801], 31.V.1977, E. I. Schlinger |
| *Eulonchus sapphirinus* | 011893 | unknown | CAS | USA, California, Nevada County, 5 miles south of Washington, [39.287, -120.801], 31.V.1977, E. I. Schlinger |
| *Eulonchus sapphirinus* | 011894 | unknown | CAS | USA, California, Nevada County, 5 miles south of Washington, [39.287, -120.801], 31.V.1977, E. I. Schlinger |
| *Eulonchus sapphirinus* | 011895 | unknown | CAS | USA, California, Nevada County, 5 miles south of Washington, [39.287, -120.801], 31.V.1977, E. I. Schlinger |
| *Eulonchus sapphirinus* | 011896 | unknown | CAS | USA, California, Nevada County, 5 miles south of Washington, [39.287, -120.801], 31.V.1977, E. I. Schlinger |
| *Eulonchus sapphirinus* | 011897 | unknown | CAS | USA, California, Nevada County, 5 miles south of Washington, [39.287, -120.801], 31.V.1977, E. I. Schlinger |
| *Eulonchus sapphirinus* | 011898 | unknown | CAS | USA, California, Nevada County, 5 miles south of Washington, [39.287, -120.801], 31.V.1977, E. I. Schlinger |
| *Eulonchus sapphirinus* | 011899 | unknown | CAS | USA, California, Nevada County, 5 miles south of Washington, [39.287, -120.801], 31.V.1977, E. I. Schlinger |
| *Eulonchus sapphirinus* | 011900 | unknown | CAS | USA, California, Nevada County, 5 miles south of Washington, [39.287, -120.801], 31.V.1977, E. I. Schlinger |
| *Eulonchus sapphirinus* | 011901 | unknown | CAS | USA, California, Nevada County, 5 miles south of Washington, [39.287, -120.801], 31.V.1977, E. I. Schlinger |
| *Eulonchus sapphirinus* | 011902 | unknown | CAS | USA, California, Nevada County, 5 miles south of Washington, [39.287, -120.801], 31.V.1977, E. I. Schlinger |
| *Eulonchus sapphirinus* | 011903 | unknown | CAS | USA, California, Nevada County, 5 miles south of Washington, [39.287, -120.801], 31.V.1977, E. I. Schlinger |
| *Eulonchus sapphirinus* | 011904 | unknown | CAS | USA, California, Nevada County, 5 miles south of Washington, [39.287, -120.801], 31.V.1977, E. I. Schlinger |
| *Eulonchus sapphirinus* | 011905 | unknown | CAS | USA, California, Nevada County, 5 miles south of Washington, [39.287, -120.801], 31.V.1977, E. I. Schlinger |
| *Eulonchus sapphirinus* | 011906 | unknown | CAS | USA, California, Nevada County, 5 miles south of Washington, [39.287, -120.801], 31.V.1977, E. I. Schlinger |
| *Eulonchus sapphirinus* | 011907 | unknown | CAS | USA, California, Nevada County, 5 miles south of Washington, [39.287, -120.801], 31.V.1977, E. I. Schlinger |
| *Eulonchus sapphirinus* | 011908 | unknown | CAS | USA, California, Nevada County, 5 miles south of Washington, [39.287, -120.801], 31.V.1977, E. I. Schlinger |
| *Eulonchus sapphirinus* | 011909 | unknown | CAS | USA, California, Nevada County, 5 miles south of Washington, [39.287, -120.801], 31.V.1977, E. I. Schlinger |
| *Eulonchus sapphirinus* | 011910 | unknown | CAS | USA, California, Nevada County, 5 miles south of Washington, [39.287, -120.801], 31.V.1977, E. I. Schlinger |
| *Eulonchus sapphirinus* | 011911 | unknown | CAS | USA, California, Nevada County, 5 miles south of Washington, [39.287, -120.801], 31.V.1977, E. I. Schlinger |
| *Eulonchus sapphirinus* | 011912 | unknown | CAS | USA, California, Nevada County, 5 miles south of Washington, [39.287, -120.801], 31.V.1977, E. I. Schlinger |
| *Eulonchus sapphirinus* | 011913 | unknown | CAS | USA, California, Nevada County, 5 miles south of Washington, [39.287, -120.801], 31.V.1977, E. I. Schlinger |
| *Eulonchus sapphirinus* | 011914 | unknown | CAS | USA, California, Nevada County, 5 miles south of Washington, [39.287, -120.801], 31.V.1977, E. I. Schlinger |
| *Eulonchus sapphirinus* | 011915 | unknown | CAS | USA, California, Nevada County, 5 miles south of Washington, [39.287, -120.801], 31.V.1977, E. I. Schlinger |
| *Eulonchus sapphirinus* | 011916 | unknown | CAS | USA, California, Nevada County, 5 miles south of Washington, [39.287, -120.801], 31.V.1977, E. I. Schlinger |
| *Eulonchus sapphirinus* | 011920 | unknown | CAS | USA, California, Nevada County, 5 miles south of Washington, [39.287, -120.801], 31.V.1977, E. I. Schlinger |
| *Eulonchus sapphirinus* | 011923 | unknown | CAS | USA, California, Nevada County, 5 miles south of Washington, [39.287, -120.801], 31.V.1977, E. I. Schlinger |
| *Eulonchus sapphirinus* | 011924 | unknown | CAS | USA, California, Nevada County, 5 miles south of Washington, [39.287, -120.801], 31.V.1977, E. I. Schlinger |
| *Eulonchus sapphirinus* | 011925 | unknown | CAS | USA, California, Nevada County, 5 miles south of Washington, [39.287, -120.801], 31.V.1977, E. I. Schlinger |
| *Eulonchus sapphirinus* | 011930 | unknown | CAS | USA, California, Nevada County, 5 miles south of Washington, [39.287, -120.801], 31.V.1977, E. I. Schlinger |
| *Eulonchus sapphirinus* | 011932 | unknown | CAS | USA, California, Nevada County, 5 miles south of Washington, [39.287, -120.801], 31.V.1977, E. I. Schlinger |
| *Eulonchus sapphirinus* | 012262 | male | CAS | USA, California, Tuolumne Co., Buck’s Meadow, Mather site, [37.813, -120.065], ..1970 to ..1971, A. R. Moldenke |
| *Eulonchus sapphirinus* | 013068 | unknown | CAS | USA, California, Mendocino County, NCCRP (Northern California Coast Range Preserve), 4.8 km North of Branscomb, [39.699, -123.627], time of day: midafternoon, 20.V.1985 to 24.V.1985, N. J. Atkinson |
| *Eulonchus sapphirinus* | 013070 | unknown | CAS | USA, California, Mendocino County, NCCRP (Northern California Coast Range Preserve), 4.8 km North of Branscomb, [39.699, -123.627], time of day: midafternoon, 20.V.1985 to 24.V.1985, N. J. Atkinson |
| *Eulonchus sapphirinus* | 013101 | unknown | CAS | USA, California, Monterey County, Big Creek Reserve, 8.1 km. N. of Lucia, Landels-Hill, [36.093, -121.549], time of day: midafternoon, 4.VI.1982 to 6.VI.1982, E. I. Schlinger |
| *Eulonchus sapphirinus* | 013108 | unknown | CAS | USA, California, Mendocino County, NCCRP (Northern California Coast Range Preserve), 4.8 km N of Branscomb, [39.697, -123.626], 427 m, time of day: midafternoon, 21.V.1982 to 23.V.1982, E. I. Schlinger |
| *Eulonchus sapphirinus* | 013143 | unknown | CAS | USA, Nevada, Elko County, Jarbridge, [41.875, -115.431], time of day: midafternoon, 16.VI.1979, R.W. Rust |
| *Eulonchus sapphirinus* | 013144 | unknown | CAS | USA, Nevada, Humbolt Co., Hinkey Summit, [41.667, -117.541], 2454 m, time of day: midafternoon, 1.VII.1981, R. C. Bechtel |
| *Eulonchus sapphirinus* | 013145 | unknown | CAS | USA, Nevada, Humbolt Co., Hinkey Summit, [41.667, -117.541], 2454 m, time of day: midafternoon, 1.VII.1981, R. C. Bechtel |
| *Eulonchus sapphirinus* | 013146 | unknown | CAS | USA, Nevada, Elko Co., Saval Ranch, [41.297, -115.911], time of day: midafternoon, 1979 |
| *Eulonchus sapphirinus* | 013147 | unknown | CAS | USA, Nevada, Elko Co., Saval Ranch, [41.297, -115.911], time of day: midafternoon, 1979 |
| *Eulonchus sapphirinus* | 013148 | unknown | CAS | USA, Nevada, Elko Co., Saval Ranch, [41.297, -115.911], time of day: midafternoon, 1979 |
| *Eulonchus sapphirinus* | 013149 | unknown | CAS | USA, Nevada, Washoe County, Little Valley, [39.807, -119.952], 22.VI.1979 |
| *Eulonchus sapphirinus* | 013159 | unknown | TAIU | USA, California, Hat Creek, [40.831, -121.514], time of day: midafternoon, 3.VII.1946, W. F. Chamberlain |
| *Eulonchus sapphirinus* | 013788 | unknown | CAS | USA, California, Tuolumne Co., Stawberry, [38.198, -120.009], 18.VI.1951, P. D. Ashlock |
| *Eulonchus sapphirinus* | 013807 | unknown | CAS | USA, Washington, Olympic National Park, Hoh River Rainforest, [47.861, -123.935], 7.VII.1968, W. W. Wirth |
| *Eulonchus sapphirinus* | 013808 | unknown | CAS | USA, Washington, Clallam Co., Port Angeles, [48.119, -123.431], 18.VI.1967, C. W. Sabrosky |
| *Eulonchus sapphirinus* | 013907 | unknown | CAS | USA, California, Butte County, Province Creek, 18.VI.1962, C. N. Slobodchikoff |
| *Eulonchus sapphirinus* | 013908 | unknown | CAS | USA, California, Sonoma County, 1 mile NE Plantation, [38.602, -123.3], 9.VI.1971, G. R. Noonan |
| *Eulonchus sapphirinus* | 013922 | unknown | CAS | USA, Washington, Mason Co., Lake Cushman, [47.481, -123.253], 2.VII.1919: 79, FM Galge |
| *Eulonchus sapphirinus* | 013923 | unknown | CAS | USA, Washington, Mason Co., Lake Cushman, [47.481, -123.253], 2.VII.1919: 79, FM Galge |
| *Eulonchus sapphirinus* | 013924 | unknown | CAS | USA, Washington, Mason Co., Lake Cushman, [47.481, -123.253], 2.VII.1919: 79, FM Galge |
| *Eulonchus sapphirinus* | 013925 | unknown | CAS | USA, Washington, Mason Co., Lake Cushman, [47.481, -123.253], 20.VI.1919: 79, FM Galge |
| *Eulonchus sapphirinus* | 013926 | unknown | CAS | USA, Utah, Juab Co., Eureka, [39.954, -122.12], 3.VI.1920, T. Spalding |
| *Eulonchus sapphirinus* | 013927 | unknown | CAS | USA, Utah, Juab County, Granite Creek, [39.729, -113.731], 7.IV.1920 |
| *Eulonchus sapphirinus* | 013928 | unknown | CAS | USA, Utah, Juab Co., Eureka, [39.954, -122.12], 7.VI.1920, T. Spalding |
| *Eulonchus sapphirinus* | 013930 | unknown | CAS | USA, Utah, Juab Co., Eureka, [39.954, -122.12], 5.VI.1920, T. Spalding |
| *Eulonchus sapphirinus* | 013931 | unknown | CAS | USA, Utah, Juab Co., Eureka, [39.954, -122.12], 14.VI.1920, T. Spalding |
| *Eulonchus sapphirinus* | 013932 | unknown | CAS | USA, Utah, Juab Co., Eureka, [39.954, -122.12], 14.VI.1920, T. Spalding |
| *Eulonchus sapphirinus* | 013933 | unknown | CAS | USA, Utah, Juab Co., Eureka, [39.954, -122.12], 8.VI.1920, T. Spalding |
| *Eulonchus sapphirinus* | 013934 | unknown | CAS | USA, Washington, Grays Harbor Co., Quinault, [47.467, -123.845], 8.VII.1960, D. J. Knull, J. N. Knull |
| *Eulonchus sapphirinus* | 013936 | unknown | CAS | USA, Washington, Grays Harbor Co., Quinault, [47.467, -123.845], 8.VII.1960, D. J. Knull, J. N. Knull |
| *Eulonchus sapphirinus* | 013937 | unknown | CAS | USA, Washington, Grays Harbor Co., Quinault, [47.467, -123.845], 8.VII.1960, D. J. Knull, J. N. Knull |
| *Eulonchus sapphirinus* | 013938 | unknown | CAS | USA, Washington, Grays Harbor Co., Quinault, [47.467, -123.845], 8.VII.1960, D. J. Knull, J. N. Knull |
| *Eulonchus sapphirinus* | 013939 | unknown | CAS | USA, California, El Dorado Co., Ice House, [38.832, -120.358], 27.VI.1964, S. G. Seminoff |
| *Eulonchus sapphirinus* | 013940 | unknown | CAS | USA, Washington, Grays Harbor Co., Quinault, [47.467, -123.845], 14.VII.1960, D. J. Knull, J. N. Knull |
| *Eulonchus sapphirinus* | 013949 | unknown | CAS | USA, California, El Dorado County, Pino Grande, NW of Lake Edson, [38.899, -120.607], 29.VI.1967, hand netted, W. J. Turner |
| *Eulonchus sapphirinus* | 013950 | unknown | CAS | USA, California, El Dorado County, Pino Grande, NW of Lake Edson, [38.899, -120.607], 29.VI.1967, hand netted, W. J. Turner |
| *Eulonchus sapphirinus* | 013951 | unknown | CAS | USA, California, El Dorado County, Pino Grande, NW of Lake Edson, [38.899, -120.607], 29.VI.1967, hand netted, W. J. Turner |
| *Eulonchus sapphirinus* | 013952 | unknown | CAS | USA, California, El Dorado County, Pino Grande, NW of Lake Edson, [38.899, -120.607], 29.VI.1967, hand netted, W. J. Turner |
| *Eulonchus sapphirinus* | 013953 | unknown | CAS | USA, California, Lassen Co., Lassen National Forest, 1/4 mile Hat Creek Campground, [40.67, -121.45], 1890 m, 3.VII.1971, hand netted, P. Loggins |
| *Eulonchus sapphirinus* | 013972 | unknown | CAS | USA, Utah, Cache County, Logan Canyon, [41.74, -111.794], 1829 m, .VI.1933 |
| *Eulonchus sapphirinus* | 013973 | unknown | CAS | USA, California, Sonoma County, [38.292, -122.458] |
| *Eulonchus sapphirinus* | 013975 | unknown | CAS | USA, California, Marin Co, Lagunitas, [38.011, -122.702], 27.IV.1958, D. C. F. Rentz |
| *Eulonchus sapphirinus* | 014060 | unknown | CAS | USA, California, Mendocino Co., 17.8 km W. Navarro, mm 318 Hwy 128, [39.151, -123.736], 12.VI.1968, D. Calvert |
| *Eulonchus sapphirinus* | 014063 | unknown | CAS | USA, Oregon, Jackson Co., Ashland, [42.195, -122.71], 3.VII.1925, H. A. Scullen |
| *Eulonchus sapphirinus* | 014064 | unknown | CAS | USA, Oregon, Jackson Co., Ashland, [42.195, -122.71], 3.VII.1925, H. A. Scullen |
| *Eulonchus sapphirinus* | 014065 | unknown | CAS | USA, Washington, Kitsap Co.,, Illahee, [47.595, -122.594], .VI.1956, D. Frechin |
| *Eulonchus sapphirinus* | 014115 | unknown | CAS | USA, California, Del Norte County, Gasquet, [41.846, -123.97], 6.VII.1960, T. R. Haig |
| *Eulonchus sapphirinus* | 014116 | unknown | CAS | USA, California, Del Norte County, Gasquet, [41.846, -123.97], 6.VII.1960, T. R. Haig |
| *Eulonchus sapphirinus* | 014489 | unknown | CAS | USA, California, Shasta County, Hat Creek, USFS Insect Lab T34N R4E Sec16, [40.831, -121.514], 25.V.1992, M. A. Valenti |
| *Eulonchus sapphirinus* | 014535 | unknown | CAS | USA, California, Placer Co., Sierra Nevada, Alta, off of I-80, [39.207, -120.811], 1220 m, 28.VI.1987, E. S. Ross |
| *Eulonchus sapphirinus* | 014575 | unknown | CAS | USA, California, Monterey County, 3.2 km E Hwy 1, on Nacimiento Road, [35.796, -120.757], 28.VI.1996, E. I. Schlinger |
| *Eulonchus sapphirinus* | 014576 | unknown | CAS | USA, California, Monterey County, 3.2 km E Hwy 1, on Nacimiento Road, [35.796, -120.757], 28.VI.1996, E. I. Schlinger |
| *Eulonchus sapphirinus* | 014677 | unknown | CAS | USA, California, Monterey County, 11 km N Lucia, [36.12, -121.548], 8.VII.1963, P. H. Arnaud, Jr. |
| *Eulonchus sapphirinus* | 014678 | unknown | CAS | USA, California, Monterey County, 11 km N Lucia, [36.12, -121.548], 8.VII.1963, P. H. Arnaud, Jr. |
| *Eulonchus sapphirinus* | 014679 | unknown | CAS | USA, California, Monterey County, 11 km N Lucia, [36.12, -121.548], 8.VII.1963, P. H. Arnaud, Jr. |
| *Eulonchus sapphirinus* | 014680 | unknown | CAS | USA, California, Monterey County, 11 km N Lucia, [36.12, -121.548], 8.VII.1963, P. H. Arnaud, Jr. |
| *Eulonchus sapphirinus* | 014681 | unknown | CAS | USA, California, Monterey County, 11 km N Lucia, [36.12, -121.548], 8.VII.1963, P. H. Arnaud, Jr. |
| *Eulonchus sapphirinus* | 014684 | unknown | CAS | USA, California, San Mateo County, Portola Valley, Alpine Road, Corte Madera Creek, [37.401, -122.238], 7.V.1960, P. H. Arnaud, Jr. |
| *Eulonchus sapphirinus* | 014685 | unknown | CAS | USA, California, San Mateo County, Portola Valley, Alpine Road, Corte Madera Creek, [37.401, -122.238], 7.V.1960, P. H. Arnaud, Jr. |
| *Eulonchus sapphirinus* | 014711 | unknown | CAS | USA, Oregon, Lane County, Whittaker Creek, Bounds Creek Road, [43.966, -123.717], 270 m, 29.VI.1999, hand netted, K. C. Holston |
| *Eulonchus sapphirinus* | 014712 | unknown | CAS | USA, Oregon, Lane County, Whittaker Creek, Bounds Creek Road, [43.966, -123.717], 270 m, 29.VI.1999, hand netted, K. C. Holston |
| *Eulonchus sapphirinus* | 014716 | unknown | CAS | USA, Oregon, Lane County, Whittaker Creek, Bounds Creek Road, [43.966, -123.717], 270 m, 29.VI.1999, hand netted, K. C. Holston |
| *Eulonchus sapphirinus* | 014718 | unknown | CAS | USA, Oregon, Lane County, Whittaker Creek, Bounds Creek Road, [43.966, -123.717], 270 m, 29.VI.1999, hand netted, K. C. Holston |
| *Eulonchus sapphirinus* | 014721 | unknown | CAS | USA, Oregon, Lane County, Whittaker Creek, Bounds Creek Road, [43.966, -123.717], 270 m, 29.VI.1999, hand netted, K. C. Holston |
| *Eulonchus sapphirinus* | 014722 | unknown | CAS | USA, Oregon, Lane County, Whittaker Creek, Bounds Creek Road, [43.966, -123.717], 270 m, 29.VI.1999, hand netted, K. C. Holston |
| *Eulonchus sapphirinus* | 014746 | unknown | CAS | USA, California, Lassen County, Susan River Camp, [40.494, -121.093], 10.VII.1949, R. G. Howell |
| *Eulonchus sapphirinus* | 014747 | unknown | CAS | USA, California, Lassen County, Susan River Camp, [40.494, -121.093], 10.VII.1949, L. Andres |
| *Eulonchus sapphirinus* | 014749 | unknown | CAS | USA, California, Lassen County, Bridge Creek Camp, [45.785, -115.213], 9.VII.1949, L. Andres |
| *Eulonchus sapphirinus* | 014789 | unknown | CAS | USA, California, Sonoma County, Salt Point State Park, [38.567, -123.325], 10.VI.1990, R. Robertson, J. K. Robertson |
| *Eulonchus sapphirinus* | 014791 | unknown | CAS | USA, California, Sonoma County, Salt Point State Park, [38.567, -123.325], 10.VI.1990, R. Robertson, J. K. Robertson |
| *Eulonchus sapphirinus* | 014792 | unknown | CAS | USA, California, Sonoma County, Salt Point State Park, [38.567, -123.325], 10.VI.1990, R. Robertson, J. K. Robertson |
| *Eulonchus sapphirinus* | 014793 | unknown | CAS | USA, California, Sonoma County, Salt Point State Park, [38.567, -123.325], 10.VI.1990, R. Robertson, J. K. Robertson |
| *Eulonchus sapphirinus* | 014794 | unknown | CAS | USA, California, Sonoma County, Salt Point State Park, [38.567, -123.325], 10.VI.1990, R. Robertson, J. K. Robertson |
| *Eulonchus sapphirinus* | 014795 | unknown | CAS | USA, California, Sonoma County, Plantation, [38.591, -123.309], 10.VI.1990, R. Robertson, J. K. Robertson |
| *Eulonchus sapphirinus* | 014797 | unknown | CAS | USA, California, Sonoma County, Plantation, [38.591, -123.309], 10.VI.1990, R. Robertson, J. K. Robertson |
| *Eulonchus sapphirinus* | 014841 | unknown | CAS | USA, California, Mendocino County, University of California Angelo Coast Range Reserve, Skunk Creek, [39.719, -123.653], 21.V.1984, M. B. Schlinger |
| *Eulonchus sapphirinus* | 014842 | unknown | CAS | USA, California, Mendocino County, University of California Angelo Coast Range Reserve, Skunk Creek, [39.719, -123.653], 21.V.1984, M. B. Schlinger |
| *Eulonchus sapphirinus* | 014843 | unknown | CAS | USA, California, Mendocino County, University of California Angelo Coast Range Reserve, Skunk Creek, [39.719, -123.653], 21.V.1984, M. B. Schlinger |
| *Eulonchus sapphirinus* | 014844 | unknown | CAS | USA, California, Mendocino County, University of California Angelo Coast Range Reserve, Skunk Creek, [39.719, -123.653], 21.V.1984, M. B. Schlinger |
| *Eulonchus sapphirinus* | 014845 | unknown | CAS | USA, California, Mendocino County, University of California Angelo Coast Range Reserve, Skunk Creek, [39.719, -123.653], 21.V.1984, M. B. Schlinger |
| *Eulonchus sapphirinus* | 014847 | unknown | CAS | USA, California, Mendocino County, University of California Angelo Coast Range Reserve, Skunk Creek, [39.719, -123.653], 21.V.1984, M. B. Schlinger |
| *Eulonchus sapphirinus* | 014848 | unknown | CAS | USA, California, Mendocino County, University of California Angelo Coast Range Reserve, Skunk Creek, [39.719, -123.653], 21.V.1984, M. B. Schlinger |
| *Eulonchus sapphirinus* | 014850 | unknown | CAS | USA, California, Mendocino County, University of California Angelo Coast Range Reserve, Skunk Creek, [39.719, -123.653], 21.V.1984, M. B. Schlinger |
| *Eulonchus sapphirinus* | 014851 | unknown | CAS | USA, California, Mendocino County, University of California Angelo Coast Range Reserve, Skunk Creek, [39.719, -123.653], 21.V.1984, M. B. Schlinger |
| *Eulonchus sapphirinus* | 015477 | unknown | CAS | USA, California, Sonoma County, 5 mi E. Kenwood, [38.413, -122.454], 30.V.1989, R. Robertson, J. K. Robertson |
| *Eulonchus sapphirinus* | 015478 | unknown | CAS | USA, California, Sonoma County, 5 mi E. Kenwood, [38.413, -122.454], 30.V.1989, R. Robertson, J. K. Robertson |
| *Eulonchus sapphirinus* | 015479 | unknown | CAS | USA, California, Sonoma County, Plantation, [38.591, -123.309], 27.V.1989, R. Robertson, J. K. Robertson |
| *Eulonchus sapphirinus* | 015480 | unknown | CAS | USA, California, Sonoma County, Plantation, [38.591, -123.309], 27.V.1989, R. Robertson, J. K. Robertson |
| *Eulonchus sapphirinus* | 015481 | unknown | CAS | USA, California, Sonoma County, Plantation, [38.591, -123.309], 27.V.1989, R. Robertson, J. K. Robertson |
| *Eulonchus sapphirinus* | 015482 | unknown | CAS | USA, California, Sonoma County, Plantation, [38.591, -123.309], 27.V.1989, R. Robertson, J. K. Robertson |
| *Eulonchus sapphirinus* | 015483 | unknown | CAS | USA, California, Sonoma County, Plantation, [38.591, -123.309], 27.V.1989, R. Robertson, J. K. Robertson |
| *Eulonchus sapphirinus* | 015484 | unknown | CAS | USA, California, Sonoma County, Plantation, [38.591, -123.309], 27.V.1989, R. Robertson, J. K. Robertson |
| *Eulonchus sapphirinus* | 015485 | unknown | CAS | USA, California, Sonoma County, Plantation, [38.591, -123.309], 17.V.1987, R. Robertson, J. K. Robertson |
| *Eulonchus sapphirinus* | 015486 | unknown | CAS | USA, California, Sonoma County, 8.1 km E Santa Rosa, [38.44, -122.622], 2.VI.1989, R. Robertson |
| *Eulonchus sapphirinus* | 015502 | unknown | CAS | USA, California, El Dorado County, 1.6 km E Pacific House, [38.759, -120.487], 20.V.1992, flume debris trap, W. D. Shepard |
| *Eulonchus sapphirinus* | 015503 | unknown | CAS | USA, California, El Dorado County, 1.6 km E Pacific House, [38.759, -120.487], 20.V.1992, flume debris trap, W. D. Shepard |
| *Eulonchus sapphirinus* | 015504 | unknown | CAS | USA, California, El Dorado County, 1.6 km E Pacific House, [38.759, -120.487], 9.V.1992, flume debris trap, W. D. Shepard |
| *Eulonchus sapphirinus* | 015505 | unknown | CAS | USA, California, El Dorado County, 1.6 km E Pacific House, [38.759, -120.487], 9.V.1992, flume debris trap, W. D. Shepard |
| *Eulonchus sapphirinus* | 015523 | unknown | CAS | USA, Washington, Olympic National Park, Elwha, [48.061, -123.593], 1.VII.1956, C. B. Philip |
| *Eulonchus sapphirinus* | 015524 | unknown | CAS | USA, Nevada, Storey County, Geiger Summit, [39.351, -119.654], 2069 m, 10.VII.1995, R. L. Langston |
| *Eulonchus sapphirinus* | 015571 | unknown | CAS | USA, California, Nevada County, Sagehen near Hobart Mills, [39.434, -120.248], 25.VI.1954, J. A. Powell |
| *Eulonchus sapphirinus* | 015572 | unknown | CAS | USA, California, Nevada County, Sagehen near Hobart Mills, [39.434, -120.248], 25.VI.1954, J. A. Powell |
| *Eulonchus sapphirinus* | 015573 | unknown | CAS | USA, California, Nevada County, Sagehen near Hobart Mills, [39.434, -120.248], 25.VI.1954, J. A. Powell |
| *Eulonchus sapphirinus* | 015574 | unknown | CAS | USA, California, Nevada County, Sagehen near Hobart Mills, [39.434, -120.248], 25.VI.1954, J. A. Powell |
| *Eulonchus sapphirinus* | 015575 | unknown | CAS | USA, California, Nevada County, Sagehen near Hobart Mills, [39.434, -120.248], 25.VI.1954, J. A. Powell |
| *Eulonchus sapphirinus* | 015576 | unknown | CAS | USA, California, Nevada County, Sagehen near Hobart Mills, [39.434, -120.248], 25.VI.1954, J. A. Powell |
| *Eulonchus sapphirinus* | 015577 | unknown | CAS | USA, California, Nevada County, Sagehen near Hobart Mills, [39.434, -120.248], 25.VI.1954, J. A. Powell |
| *Eulonchus sapphirinus* | 015578 | unknown | CAS | USA, California, Nevada County, Sagehen near Hobart Mills, [39.434, -120.248], 25.VI.1954, J. A. Powell |
| *Eulonchus sapphirinus* | 015579 | unknown | CAS | USA, California, Nevada County, Sagehen near Hobart Mills, [39.434, -120.248], 25.VI.1954, J. A. Powell |
| *Eulonchus sapphirinus* | 015580 | unknown | CAS | USA, California, Nevada County, Sagehen near Hobart Mills, [39.434, -120.248], 2.VII.1954, J. A. Powell |
| *Eulonchus sapphirinus* | 015581 | unknown | CAS | USA, California, Tehama County, Lassen National Park, NE corner of county, [40.347, -121.614], 2027 m, 14.VII.1965, R. L. Langston |
| *Eulonchus sapphirinus* | 015667 | unknown | CAS | USA, California, Tuolumne County, vicinity of Sonora Peak, [38.335, -119.655], 1.VII.1961, D. C. F. Rentz |
| *Eulonchus sapphirinus* | 015668 | unknown | CAS | USA, California, Tuolumne County, vicinity of Sonora Peak, [38.335, -119.655], 1.VII.1961, D. C. F. Rentz |
| *Eulonchus sapphirinus* | 015669 | unknown | CAS | USA, California, Siskiyou County, 12.9 mi E of Bartle, [41.259, -121.668], 22.VII.1962, D. C. F. Rentz, C. D. MacNeill |
| *Eulonchus sapphirinus* | 015670 | unknown | CAS | USA, California, Siskiyou County, 12.9 mi E of Bartle, [41.259, -121.668], 22.VII.1962, D. C. F. Rentz, C. D. MacNeill |
| *Eulonchus sapphirinus* | 015671 | unknown | CAS | USA, California, Siskiyou County, 12.9 mi E of Bartle, [41.259, -121.668], 22.VII.1962, D. C. F. Rentz, C. D. MacNeill |
| *Eulonchus sapphirinus* | 015672 | unknown | CAS | USA, California, Tuolumne County, vicinity of Sonora Peak, [38.335, -119.655], 23.VI.1960, D. C. F. Rentz |
| *Eulonchus sapphirinus* | 015673 | unknown | CAS | USA, California, Tuolumne County, vicinity of Sonora Peak, [38.335, -119.655], 23.VI.1960, D. C. F. Rentz |
| *Eulonchus sapphirinus* | 015674 | unknown | CAS | USA, California, Siskiyou County, 6.4 km NE Mount Shasta, McBride Springs, [41.354, -122.285], 4.VII.1963, V. B. Whitehead |
| *Eulonchus sapphirinus* | 015773 | unknown | CAS | USA, California, Trinity County, 12.9 air-km W Hayfork, Buttercreek Meadows, [40.553, -123.335], 21.V.1973, J. A. Powell |
| *Eulonchus sapphirinus* | 015864 | unknown | CAS | USA, California, El Dorado County, Pino Grande, NW of Lake Edson, [38.899, -120.607], 29.VI.1967, hand netted, A. J. Gilbert |
| *Eulonchus sapphirinus* | 015865 | unknown | CAS | USA, California, El Dorado County, Pino Grande, NW of Lake Edson, [38.899, -120.607], 29.VI.1967, hand netted, A. J. Gilbert |
| *Eulonchus sapphirinus* | 015866 | unknown | CAS | USA, California, El Dorado County, Pino Grande, NW of Lake Edson, [38.899, -120.607], 29.VI.1967, hand netted, A. J. Gilbert |
| *Eulonchus sapphirinus* | 015867 | unknown | CAS | USA, California, El Dorado County, Pino Grande, NW of Lake Edson, [38.899, -120.607], 29.VI.1967, hand netted, A. J. Gilbert |
| *Eulonchus sapphirinus* | 015868 | unknown | CAS | USA, California, El Dorado County, Pino Grande, NW of Lake Edson, [38.899, -120.607], 29.VI.1967, hand netted, A. J. Gilbert |
| *Eulonchus sapphirinus* | 015869 | unknown | CAS | USA, California, El Dorado County, Blodgett Forest Research Station, 20.9 km east of Georgetown, [38.909, -120.661], 1300 m, 24.VI.1967, A. J. Gilbert |
| *Eulonchus sapphirinus* | 015870 | unknown | CAS | USA, California, El Dorado County, Blodgett Forest Research Station, 20.9 km east of Georgetown, [38.909, -120.661], 1300 m, 24.VI.1967, A. J. Gilbert |
| *Eulonchus sapphirinus* | 015872 | unknown | CAS | USA, California, El Dorado County, Blodgett Forest Research Station, 20.9 km east of Georgetown, [38.909, -120.661], 1300 m, 13.VII.1967, A. J. Gilbert |
| *Eulonchus sapphirinus* | 015873 | unknown | CAS | USA, California, Lassen County, Lassen Volcanic National Park, 3.2 km S Snag Lake, [40.475, -121.311], 9.VII.1964, T. R. Haig |
| *Eulonchus sapphirinus* | 015874 | unknown | CAS | USA, California, El Dorado County, Pino Grande, [38.87, -120.625], 22.V.1966, hand netted, P. E. Adams |
| *Eulonchus sapphirinus* | 015875 | unknown | CAS | USA, California, El Dorado County, Pino Grande, [38.87, -120.625], 22.V.1966, hand netted, P. E. Adams |
| *Eulonchus sapphirinus* | 015877 | unknown | CAS | USA, California, El Dorado County, El Dorado National Forest, Stumpy Meadows, [38.904, -120.593], 26.V.1966, hand netted, P. E. Adams |
| *Eulonchus sapphirinus* | 015878 | unknown | CAS | USA, California, El Dorado County, El Dorado National Forest, Stumpy Meadows, [38.904, -120.593], 26.V.1966, hand netted, P. E. Adams |
| *Eulonchus sapphirinus* | 015879 | unknown | CAS | USA, California, Amador County, 8.1 km E Jackson, [38.349, -120.682], 26.VI.1960, |
| *Eulonchus sapphirinus* | 015883 | unknown | CAS | USA, California, Tuolumne County, 4 km S. Mather, Middle Fork Tuolumne River, Middle Fork Campground, [37.846, -119.854], 24.V.1969, P. H. Arnaud, Jr. |
| *Eulonchus sapphirinus* | 015885 | unknown | CAS | USA, California, Tuolumne County, 4 km S. Mather, Middle Fork Tuolumne River, Middle Fork Campground, [37.846, -119.854], 24.V.1969, P. H. Arnaud, Jr. |
| *Eulonchus sapphirinus* | 015886 | unknown | CAS | USA, California, Tuolumne County, 4 km S. Mather, Middle Fork Tuolumne River, Middle Fork Campground, [37.846, -119.854], 24.V.1969, P. H. Arnaud, Jr. |
| *Eulonchus sapphirinus* | 015887 | unknown | CAS | USA, California, Tuolumne County, 4 km S. Mather, Middle Fork Tuolumne River, Middle Fork Campground, [37.846, -119.854], 24.V.1969, P. H. Arnaud, Jr. |
| *Eulonchus sapphirinus* | 015894 | unknown | CAS | USA, California, Nevada County, White Cloud Campground, [39.321, -120.846], 30.V.1964, P. H. Arnaud, Jr. |
| *Eulonchus sapphirinus* | 015956 | unknown | CAS | USA, California, Trinity County, 12.9 km W Hayfork, Buttercreek Meadow, [40.553, -123.335], 1143 m, 20.V.1973, R. E. Dietz |
| *Eulonchus sapphirinus* | 015989 | unknown | CAS | USA, Oregon, Jackson County, Rogue River National Forest, 3.2 km N Bull Gap, [42.125, -122.685], 3.VII.1970, J. Dietz, P. A. Rude |
| *Eulonchus sapphirinus* | 015990 | unknown | CAS | USA, California, Marin County, Bear Valley Trail., [38.013, -122.801], 30 m, 18.V.1986, hand netted, E. I. Schlinger |
| *Eulonchus sapphirinus* | 015991 | unknown | CAS | USA, California, Marin County, Bear Valley Trail., [38.013, -122.801], 30 m, 18.V.1986, hand netted, E. I. Schlinger |
| *Eulonchus sapphirinus* | 015992 | unknown | CAS | USA, California, Marin County, Bear Valley Trail., [38.013, -122.801], 30 m, 18.V.1986, hand netted, E. I. Schlinger |
| *Eulonchus sapphirinus* | 015993 | unknown | CAS | USA, California, Marin County, Bear Valley Trail., [38.013, -122.801], 30 m, 18.V.1986, hand netted, E. I. Schlinger |
| *Eulonchus sapphirinus* | 015994 | unknown | CAS | USA, California, Marin County, Bear Valley Trail., [38.013, -122.801], 30 m, 18.V.1986, hand netted, E. I. Schlinger |
| *Eulonchus sapphirinus* | 016348 | unknown | CAS | USA, Oregon, Klamath County, Lake of the Woods, [42.366, -122.213], 23.VI.1951, C. Fitch |
| *Eulonchus sapphirinus* | 016350 | unknown | CAS | USA, Oregon, Klamath County, Lake of the Woods, [42.366, -122.213], 23.VI.1951, C. Fitch |
| *Eulonchus sapphirinus* | 016355 | female | CAS | USA, Oregon, Klamath County, Lake of the Woods, [42.366, -122.213], 23.VI.1951, M. F. McClay |
| *Eulonchus sapphirinus* | 016356 | male | CAS | USA, Oregon, Klamath County, Lake of the Woods, [42.366, -122.213], 23.VI.1951, M. F. McClay |
| *Eulonchus sapphirinus* | 016357 | unknown | CAS | USA, Oregon, Klamath County, Lake of the Woods, [42.366, -122.213], 23.VI.1951, M. F. McClay |
| *Eulonchus sapphirinus* | 016358 | unknown | CAS | USA, Oregon, Klamath County, Lake of the Woods, [42.366, -122.213], 23.VI.1951, M. F. McClay |
| *Eulonchus sapphirinus* | 016359 | unknown | CAS | USA, Oregon, Klamath County, Lake of the Woods, [42.366, -122.213], 23.VI.1951, M. F. McClay |
| *Eulonchus sapphirinus* | 016360 | unknown | CAS | USA, Oregon, Klamath County, Lake of the Woods, [42.366, -122.213], 23.VI.1951, M. F. McClay |
| *Eulonchus sapphirinus* | 016361 | unknown | CAS | USA, Oregon, Klamath County, Lake of the Woods, [42.366, -122.213], 23.VI.1951, M. F. McClay |
| *Eulonchus sapphirinus* | 016362 | unknown | CAS | USA, Oregon, Klamath County, Lake of the Woods, [42.366, -122.213], 23.VI.1951, M. F. McClay |
| *Eulonchus sapphirinus* | 016363 | unknown | CAS | USA, Oregon, Klamath County, Lake of the Woods, [42.366, -122.213], 23.VI.1951, M. F. McClay |
| *Eulonchus sapphirinus* | 016364 | unknown | CAS | USA, Oregon, Klamath County, Lake of the Woods, [42.366, -122.213], 23.VI.1951, M. F. McClay |
| *Eulonchus sapphirinus* | 016365 | unknown | CAS | USA, Oregon, Klamath County, Lake of the Woods, [42.366, -122.213], 23.VI.1951, M. F. McClay |
| *Eulonchus sapphirinus* | 016366 | unknown | CAS | USA, Oregon, Klamath County, Lake of the Woods, [42.366, -122.213], 23.VI.1951, M. F. McClay |
| *Eulonchus sapphirinus* | 016367 | unknown | CAS | USA, Oregon, Klamath County, Lake of the Woods, [42.366, -122.213], 23.VI.1951, M. F. McClay |
| *Eulonchus sapphirinus* | 016377 | unknown | CAS | USA, California, Siskiyou County, 29.V.1911, F. W. Nunenmacher |
| *Eulonchus sapphirinus* | 016378 | unknown | CAS | USA, California, Siskiyou County, 29.V.1911, F. W. Nunenmacher |
| *Eulonchus sapphirinus* | 016379 | unknown | CAS | USA, California, Siskiyou County, 29.V.1911, F. W. Nunenmacher |
| *Eulonchus sapphirinus* | 016380 | unknown | CAS | USA, California, Siskiyou County, 29.V.1911, F. W. Nunenmacher |
| *Eulonchus sapphirinus* | 016381 | unknown | CAS | USA, California, Siskiyou County, 29.V.1911, F. W. Nunenmacher |
| *Eulonchus sapphirinus* | 016382 | unknown | CAS | USA, California, Siskiyou County, 29.V.1911, F. W. Nunenmacher |
| *Eulonchus sapphirinus* | 016383 | unknown | CAS | USA, California, Siskiyou County, 29.V.1911, F. W. Nunenmacher |
| *Eulonchus sapphirinus* | 016384 | unknown | CAS | USA, California, Siskiyou County, 29.V.1911, F. W. Nunenmacher |
| *Eulonchus sapphirinus* | 016385 | unknown | CAS | USA, California, C. W. Johnson |
| *Eulonchus sapphirinus* | 016386 | unknown | CAS | USA, Oregon, Crater Lake National Park, near Bald top, [42.805, -122.214], 10.VII.1951, D. C. Lowrie |
| *Eulonchus sapphirinus* | 016387 | unknown | CAS | USA, Oregon, Crater Lake National Park, near Bald top, [42.805, -122.214], 10.VII.1951, D. C. Lowrie |
| *Eulonchus sapphirinus* | 016388 | unknown | CAS | USA, Oregon, Jackson Co., Ashland, [42.195, -122.71], 2.VI.1984, C. Fitch |
| *Eulonchus sapphirinus* | 016389 | unknown | CAS | USA, Oregon, Jackson Co., Ashland, [42.195, -122.71], 2.VI.1984, C. Fitch |
| *Eulonchus sapphirinus* | 016390 | unknown | CAS | USA, Oregon, Jackson Co., Ashland, [42.195, -122.71], 2.VI.1984, C. Fitch |
| *Eulonchus sapphirinus* | 016391 | unknown | CAS | USA, Oregon, Jackson Co., Ashland, [42.195, -122.71], 6.VI.1916, F. P. Keen, F. D. Sergent |
| *Eulonchus sapphirinus* | 016392 | unknown | CAS | USA, Oregon, Jackson County, Griffin Creek, [42.34, -122.924], 17.VI.1954, C. Fitch |
| *Eulonchus sapphirinus* | 016393 | unknown | CAS | USA, Oregon, Jackson County, Griffin Creek, [42.34, -122.924], 17.VI.1954, C. Fitch |
| *Eulonchus sapphirinus* | 016491 | unknown | CAS | USA, Washington, Clallam County, Forks, [47.951, -124.385], 1.VII.1920, E. P. Van Duzee |
| *Eulonchus sapphirinus* | 016496 | unknown | CAS | USA, Washington, Clallam County, Forks, [47.951, -124.385], 1.VII.1920, E. P. Van Duzee |
| *Eulonchus sapphirinus* | 016547 | male | UCDC | USA, Arizona, North Rim Grand Canyon, 5.VI.1946, R. M. Bohart |
| *Eulonchus sapphirinus* | 016548 | male | UCDC | USA, Arizona, North Rim Grand Canyon, 5.VI.1946, R. M. Bohart |
| *Eulonchus sapphirinus* | 016549 | unknown | UCDC | USA, Arizona, North Rim Grand Canyon, 5.VI.1946, R. M. Bohart |
| *Eulonchus sapphirinus* | 016550 | unknown | UCDC | USA, Arizona, North Rim Grand Canyon, 5.VI.1946, R. M. Bohart |
| *Eulonchus sapphirinus* | 016551 | unknown | UCDC | USA, Arizona, North Rim Grand Canyon, 5.VI.1946, R. M. Bohart |
| *Eulonchus sapphirinus* | 016552 | unknown | UCDC | USA, Arizona, North Rim Grand Canyon, 5.VI.1946, R. M. Bohart |
| *Eulonchus sapphirinus* | 016553 | unknown | UCDC | USA, Arizona, North Rim Grand Canyon, 5.VI.1946, R. M. Bohart |
| *Eulonchus sapphirinus* | 016554 | male | UCDC | USA, Arizona, North Rim Grand Canyon, 5.VI.1946, R. M. Bohart |
| *Eulonchus sapphirinus* | 016555 | unknown | UCDC | USA, Arizona, North Rim Grand Canyon, 5.VI.1946, R. M. Bohart |
| *Eulonchus sapphirinus* | 016556 | unknown | UCDC | USA, Arizona, North Rim Grand Canyon, 5.VI.1946, R. M. Bohart |
| *Eulonchus sapphirinus* | 016557 | unknown | UCDC | USA, Arizona, North Rim Grand Canyon, 5.VI.1946, R. M. Bohart |
| *Eulonchus sapphirinus* | 016558 | unknown | UCDC | USA, Arizona, North Rim Grand Canyon, 5.VI.1946, R. M. Bohart |
| *Eulonchus sapphirinus* | 016559 | unknown | UCDC | USA, Arizona, North Rim Grand Canyon, 5.VI.1946, R. M. Bohart |
| *Eulonchus sapphirinus* | 016560 | unknown | UCDC | USA, Arizona, North Rim Grand Canyon, 5.VI.1946, R. M. Bohart |
| *Eulonchus sapphirinus* | 016561 | unknown | UCDC | USA, Arizona, North Rim Grand Canyon, 5.VI.1946, R. M. Bohart |
| *Eulonchus sapphirinus* | 016562 | unknown | UCDC | USA, Arizona, North Rim Grand Canyon, 5.VI.1946, R. M. Bohart |
| *Eulonchus sapphirinus* | 016563 | unknown | UCDC | USA, Arizona, North Rim Grand Canyon, 5.VI.1946, R. M. Bohart |
| *Eulonchus sapphirinus* | 016564 | unknown | UCDC | USA, Arizona, North Rim Grand Canyon, 5.VI.1946, R. M. Bohart |
| *Eulonchus sapphirinus* | 016565 | unknown | UCDC | USA, Arizona, North Rim Grand Canyon, 5.VI.1946, R. M. Bohart |
| *Eulonchus sapphirinus* | 016566 | unknown | UCDC | USA, Arizona, North Rim Grand Canyon, 5.VI.1946, R. M. Bohart |
| *Eulonchus sapphirinus* | 016567 | unknown | UCDC | USA, Arizona, North Rim Grand Canyon, 5.VI.1946, R. M. Bohart |
| *Eulonchus sapphirinus* | 016568 | unknown | UCDC | USA, Arizona, North Rim Grand Canyon, 5.VI.1946, R. M. Bohart |
| *Eulonchus sapphirinus* | 016569 | unknown | UCDC | USA, Arizona, North Rim Grand Canyon, 5.VI.1946, R. M. Bohart |
| *Eulonchus sapphirinus* | 016570 | female | UCDC | USA, Arizona, North rim Grand Canyon, 2438 m, 1.VI.1946, R. M. Bohart |
| *Eulonchus sapphirinus* | 016571 | male | UCDC | USA, Arizona, North rim Grand Canyon, 2438 m, 1.VI.1946, R. M. Bohart |
| *Eulonchus sapphirinus* | 016572 | unknown | CAS | USA, Utah, Washington County, Pintura |
| *Eulonchus sapphirinus* | 016573 | unknown | CAS | USA, Arizona, Coconino County, Jacob Lake, 18.VI.1949, G. F. Knowlton |
| *Eulonchus sapphirinus* | 016593 | unknown | CAS | USA, Washington, Thurston County, Olympia, [47.038, -122.901], 6.VII.1933, C. H. Martin, D. Martin |
| *Eulonchus sapphirinus* | 016595 | unknown | CAS | USA, Washington, Thurston County, Olympia, [47.038, -122.901], 6.VII.1933, C. H. Martin, D. Martin |
| *Eulonchus sapphirinus* | 016596 | unknown | CAS | USA, Washington, Thurston County, Olympia, [47.038, -122.901], 6.VII.1933, C. H. Martin, D. Martin |
| *Eulonchus sapphirinus* | 016597 | unknown | CAS | USA, Washington, Thurston County, Olympia, [47.038, -122.901], 6.VII.1933, C. H. Martin, D. Martin |
| *Eulonchus sapphirinus* | 016598 | unknown | CAS | USA, Washington, Thurston County, Olympia, [47.038, -122.901], 6.VII.1933, C. H. Martin, D. Martin |
| *Eulonchus sapphirinus* | 016599 | unknown | CAS | USA, Washington, Thurston County, Olympia, [47.038, -122.901], 15.VII.1933, C. H. Martin |
| *Eulonchus sapphirinus* | 016600 | unknown | CAS | USA, Washington, Thurston County, Olympia, [47.038, -122.901], 28.V.1932, C. H. Martin |
| *Eulonchus sapphirinus* | 016601 | unknown | CAS | USA, Washington, C. W. Johnson |
| *Eulonchus sapphirinus* | 016602 | unknown | CAS | USA, Washington Territory |
| *Eulonchus sapphirinus* | 016603 | unknown | CAS | USA, Washington, Snohomish County, Penn Mines |
| *Eulonchus sapphirinus* | 016604 | unknown | CAS | USA, West Washington Territory, H.K. Morrison |
| *Eulonchus sapphirinus* | 016605 | unknown | CAS | USA, West Washington Territory, H.K. Morrison |
| *Eulonchus sapphirinus* | 016606 | unknown | CAS | USA, Washington, Mason Co., Lake Cushman, [47.481, -123.253], 26.VI.1922, P. G. Putnam |
| *Eulonchus sapphirinus* | 016608 | male | CAS | USA, Washington, King County, Seattle, [47.608, -122.332], 17.VI.1901 |
| *Eulonchus sapphirinus* | 016609 | unknown | CAS | USA, Washington, King County, Seattle, [47.608, -122.332], 19.VI.1920, E. P. Van Duzee |
| *Eulonchus sapphirinus* | 016610 | unknown | CAS | USA, Washington, Chinook Pass, [46.872, -121.516], 29.VII.1949, L. D. Beamer |
| *Eulonchus sapphirinus* | 016705 | female | CAS | USA, California, Trinity County, Coffee Creek Ranch, 914 m, 19.VI.1972, D. P. Levin |
| *Eulonchus sapphirinus* | 017548 | unknown | CAS | USA, Oregon, Crater Lake National Park, [42.871, -122.168], 7.VIII.1963, Schuh, Hansen, Miller |
| *Eulonchus sapphirinus* | 017549 | unknown | CAS | USA, Oregon, Crater Lake National Park, [42.871, -122.168], 7.VIII.1963, Schuh, Hansen, Miller |
| *Eulonchus sapphirinus* | 017550 | unknown | CAS | USA, Oregon, Crater Lake National Park, [42.871, -122.168], 7.VIII.1963, Schuh, Hansen, Miller |
| *Eulonchus sapphirinus* | 017560 | unknown | CAS | USA, Washington, Clallam County, Olympic National Park, Lake Crescent, [43.475, -121.986], 18.VI.1967, B. A. Freeman |
| *Eulonchus sapphirinus* | 017571 | unknown | CAS | USA, Oregon, Josephine County, Oregon Mountain, [42.001, -123.798], 4.VII.1942, R. E. Rieder |
| *Eulonchus sapphirinus* | 017799 | unknown | CAS | USA, California, El Dorado County, Pollock Pines, [38.761, -120.587], 28.V.1970, D. P. Levin |
| *Eulonchus sapphirinus* | 017802 | unknown | CAS | USA, California, Shasta County, Hat Creek, [40.831, -121.514], 21.VI.1955, C. L. Hogue |
| *Eulonchus sapphirinus* | 017803 | unknown | CAS | USA, Washington, Clallam County, Olympic National Park, Sol Duc Ranger Station, [47.972, -123.864], 15.VI.1965, D. Rice |
| *Eulonchus sapphirinus* | 017822 | unknown | CAS | USA, California, Marin County, Inverness Ridge, [38.102, -122.887], 15.V.1970, J. Scott |
| *Eulonchus sapphirinus* | 017823 | unknown | CAS | USA, California, Marin County, Inverness Ridge, [38.102, -122.887], 15.V.1970, J. Scott |
| *Eulonchus sapphirinus* | 017824 | unknown | CAS | USA, California, Marin County, Inverness Ridge, [38.102, -122.887], 15.V.1970, J. Scott |
| *Eulonchus sapphirinus* | 017947 | unknown | CAS | USA, California, Siskiyou County, 8.1 to 16.1 km N.E. of Bartle, [41.262, -121.818], 20.VI.1954, hand netted, E. I. Schlinger |
| *Eulonchus sapphirinus* | 017948 | unknown | CAS | USA, California, Siskiyou County, 8.1 to 16.1 km N.E. of Bartle, [41.262, -121.818], 20.VI.1954, hand netted, E. I. Schlinger |
| *Eulonchus sapphirinus* | 017949 | unknown | CAS | USA, California, Siskiyou County, 8.1 to 16.1 km N.E. of Bartle, [41.262, -121.818], 20.VI.1954, hand netted, E. I. Schlinger |
| *Eulonchus sapphirinus* | 017950 | unknown | CAS | USA, California, Siskiyou County, 8.1 to 16.1 km N.E. of Bartle, [41.262, -121.818], 20.VI.1954, hand netted, E. I. Schlinger |
| *Eulonchus sapphirinus* | 017951 | unknown | CAS | USA, California, Siskiyou County, 8.1 to 16.1 km N.E. of Bartle, [41.262, -121.818], 20.VI.1954, hand netted, A. A. Grigarick |
| *Eulonchus sapphirinus* | 018331 | female | CAS | USA, California, Sierra County, Tahoe National Forest, Lake of the Woods, [39.504, -120.392], 4.VII.1959, W. E. Simonds |
| *Eulonchus sapphirinus* | 018468 | unknown | CAS | USA, California, Placer County, East of Auburn, Foresthill, [39.021, -120.818], 15.IV.1980, E. L. Klee |
| *Eulonchus sapphirinus* | 018475 | unknown | CAS | USA, California, Nevada County, 8.VI.1933 |
| *Eulonchus sapphirinus* | 018476 | unknown | CAS | USA, California, El Dorado County, Kyburz, [38.775, -120.297], 6.VI.1981 to 13.VI.1981, Malaise trap, S. C. Kuba |
| *Eulonchus sapphirinus* | 018515 | unknown | CAS | USA, California, El Dorado County, El Dorado National Forest, Leonardi Spring, [38.909, -120.547], 1341 m, 7.VI.1970, hand netted, J. L. Kipping |
| *Eulonchus sapphirinus* | 018516 | unknown | CAS | USA, California, El Dorado County, El Dorado National Forest, Leonardi Spring, [38.909, -120.547], 1341 m, 6.VI.1970, hand netted, J. L. Kipping |
| *Eulonchus sapphirinus* | 018517 | unknown | CAS | USA, California, El Dorado County, El Dorado National Forest, Leonardi Spring, [38.909, -120.547], 1341 m, 6.VI.1970, hand netted, J. L. Kipping |
| *Eulonchus sapphirinus* | 018539 | unknown | CAS | USA, Idaho, Bear Lake County, above Home Canyon, Fox Springs, [42.382, -111.229], 20.VI.1966, R. L. Westcott |
| *Eulonchus sapphirinus* | 018540 | unknown | CAS | USA, Idaho, Bear Lake County, above Home Canyon, Fox Springs, [42.382, -111.229], 20.VI.1966, R. L. Westcott |
| *Eulonchus sapphirinus* | 018541 | unknown | CAS | USA, Idaho, Bear Lake County, above Home Canyon, Fox Springs, [42.382, -111.229], 20.VI.1966, R. L. Westcott |
| *Eulonchus sapphirinus* | 018542 | unknown | CAS | USA, Idaho, Bear Lake County, above Home Canyon, Fox Springs, [42.382, -111.229], 20.VI.1966, R. L. Westcott |
| *Eulonchus sapphirinus* | 018543 | unknown | CAS | USA, Oregon, Curry County, Kalmiopsis Wilderness, Vulcan Lake trail, [42.283, -123.965], 5.VII.1969, R. L. Westcott |
| *Eulonchus sapphirinus* | 018544 | unknown | CAS | USA, Oregon, Curry County, Kalmiopsis Wilderness, Vulcan Lake trail, [42.283, -123.965], 5.VII.1969, R. L. Westcott |
| *Eulonchus sapphirinus* | 018545 | unknown | CAS | USA, Oregon, Curry County, Kalmiopsis Wilderness, Vulcan Lake trail, [42.283, -123.965], 5.VII.1969, R. L. Westcott |
| *Eulonchus sapphirinus* | 018546 | unknown | CAS | USA, Oregon, Curry County, Kalmiopsis Wilderness, Vulcan Lake trail, [42.283, -123.965], 5.VII.1969, R. L. Westcott |
| *Eulonchus sapphirinus* | 018547 | unknown | CAS | USA, Oregon, Curry County, Kalmiopsis Wilderness, Vulcan Lake trail, [42.283, -123.965], 5.VII.1969, R. L. Westcott |
| *Eulonchus sapphirinus* | 018548 | unknown | CAS | USA, Oregon, Curry County, Kalmiopsis Wilderness, Vulcan Lake trail, [42.283, -123.965], 5.VII.1969, R. L. Westcott |
| *Eulonchus sapphirinus* | 018549 | unknown | CAS | USA, Oregon, Curry County, Kalmiopsis Wilderness, Vulcan Lake trail, [42.283, -123.965], 5.VII.1969, R. L. Westcott |
| *Eulonchus sapphirinus* | 018550 | unknown | CAS | USA, Oregon, Curry County, Kalmiopsis Wilderness, Vulcan Lake trail, [42.283, -123.965], 5.VII.1969, R. L. Westcott |
| *Eulonchus sapphirinus* | 018551 | unknown | CAS | USA, Oregon, Curry County, Kalmiopsis Wilderness, Vulcan Lake trail, [42.283, -123.965], 5.VII.1969, R. L. Westcott |
| *Eulonchus sapphirinus* | 018552 | unknown | CAS | USA, Oregon, Curry County, Kalmiopsis Wilderness, Vulcan Lake trail, [42.283, -123.965], 5.VII.1969, R. L. Westcott |
| *Eulonchus sapphirinus* | 018554 | unknown | CAS | USA, Oregon, Curry County, Kalmiopsis Wilderness, Vulcan Lake trail, [42.283, -123.965], 5.VII.1969, R. L. Westcott |
| *Eulonchus sapphirinus* | 018555 | unknown | CAS | USA, Oregon, Curry County, Kalmiopsis Wilderness, Vulcan Lake trail, [42.283, -123.965], 5.VII.1969, R. L. Westcott |
| *Eulonchus sapphirinus* | 018556 | unknown | CAS | USA, Oregon, Curry County, Kalmiopsis Wilderness, Vulcan Lake trail, [42.283, -123.965], 5.VII.1969, R. L. Westcott |
| *Eulonchus sapphirinus* | 018557 | unknown | CAS | USA, Oregon, Curry County, Kalmiopsis Wilderness, Vulcan Lake trail, [42.283, -123.965], 5.VII.1969, R. L. Westcott |
| *Eulonchus sapphirinus* | 018558 | unknown | CAS | USA, Oregon, Curry County, Kalmiopsis Wilderness, Vulcan Lake trail, [42.283, -123.965], 5.VII.1969, R. L. Westcott |
| *Eulonchus sapphirinus* | 018559 | unknown | CAS | USA, Oregon, Curry County, Kalmiopsis Wilderness, Vulcan Lake trail, [42.283, -123.965], 5.VII.1969, R. L. Westcott |
| *Eulonchus sapphirinus* | 018568 | unknown | CAS | USA, Washington, Kittitas County, Fish Lake, [47.524, -121.073], 27.VII.1969, D. S. Horning |
| *Eulonchus sapphirinus* | 018569 | unknown | CAS | USA, Washington, Kittitas County, Fish Lake, [47.524, -121.073], 27.VII.1969, D. S. Horning |
| *Eulonchus sapphirinus* | 018570 | unknown | CAS | USA, Washington, Kittitas County, Fish Lake, [47.524, -121.073], 27.VII.1969, D. S. Horning |
| *Eulonchus sapphirinus* | 018571 | unknown | CAS | USA, Washington, Kittitas County, Fish Lake, [47.524, -121.073], 27.VII.1969, D. S. Horning |
| *Eulonchus sapphirinus* | 018572 | unknown | CAS | USA, Washington, Kittitas County, Fish Lake, [47.524, -121.073], 27.VII.1969, D. S. Horning |
| *Eulonchus sapphirinus* | 018573 | unknown | CAS | USA, Washington, Kittitas County, Fish Lake, [47.524, -121.073], 27.VII.1969, D. S. Horning |
| *Eulonchus sapphirinus* | 018575 | unknown | CAS | USA, Washington, Kittitas County, Fish Lake, [47.524, -121.073], 27.VII.1969, D. S. Horning |
| *Eulonchus sapphirinus* | 018578 | unknown | CAS | USA, Oregon, Clatsop County, Saddle Mountain, [45.969, -123.685], 14.VI.1969, R. L. Westcott |
| *Eulonchus sapphirinus* | 018579 | unknown | CAS | USA, Oregon, Clatsop County, Saddle Mountain, [45.969, -123.685], 14.VI.1969, R. L. Westcott |
| *Eulonchus sapphirinus* | 018580 | unknown | CAS | USA, Oregon, Clatsop County, Saddle Mountain, [45.969, -123.685], 14.VI.1969, R. L. Westcott |
| *Eulonchus sapphirinus* | 018581 | unknown | CAS | USA, Oregon, Clatsop County, Saddle Mountain, [45.969, -123.685], 14.VI.1969, R. L. Westcott |
| *Eulonchus sapphirinus* | 018582 | unknown | CAS | USA, Oregon, Clatsop County, Saddle Mountain, [45.969, -123.685], 14.VI.1969, R. L. Westcott |
| *Eulonchus sapphirinus* | 018929 | unknown | CAS | USA, California, Marin County, 0.8 km W Alpine Lake, [37.95, -122.638], 24.V.1985 |
| *Eulonchus sapphirinus* | 018981 | unknown | CAS | USA, California, Mendocino County, NCCRP (Northern California Coast Range Preserve), 8.1 km. N. of Branscomb, [39.77, -123.628], 26.V.1976, J. A. Powell |
| *Eulonchus sapphirinus* | 019003 | unknown | CAS | USA, California, El Dorado County, Blodgett Forest Research Station, 19.3 km east of Georgetown, [38.909, -120.661], 1300 m, 30.V.1974, E. I. Schlinger |
| *Eulonchus sapphirinus* | 019007 | unknown | CAS | USA, California, Santa Cruz County, Big Basin State Park, [37.172, -122.223], 30.IV.1978, C. Montllor |
| *Eulonchus sapphirinus* | 019022 | unknown | CAS | USA, California, Mendocino County, NCCRP (Northern California Coast Range Preserve), 8.1 km. N. of Branscomb, [39.77, -123.628], 25.V.1976, J. A. Chemsak |
| *Eulonchus sapphirinus* | 019042 | unknown | CAS | USA, California, El Dorado County, Blodgett Forest Research Station, 12.9 km E of Georgetown, [38.906, -120.69], 6.VII.1967, C. O. Dudley |
| *Eulonchus sapphirinus* | 019047 | unknown | CAS | USA, California, El Dorado County, Blodgett Forest Research Station, 19.3 km east of Georgetown, [38.909, -120.661], 1300 m, 26.V.1973, R. Wharton |
| *Eulonchus sapphirinus* | 019048 | unknown | CAS | USA, California, Siskiyou County, 14.5 km E McCloud, Ash Creek Ranger Station, [41.256, -121.967], 1067 m, 7.VI.1974 to 9.VI.1974, hand netted, R. Coville |
| *Eulonchus sapphirinus* | 019053 | unknown | CAS | USA, California, El Dorado County, Blodgett Forest Research Station, 16.1 km E Georgetown, [38.908, -120.654], 10.VI.1965, G. Buckingham |
| *Eulonchus sapphirinus* | 019054 | unknown | CAS | USA, California, El Dorado County, Blodgett Forest Research Station, 16.1 km E Georgetown, [38.908, -120.654], 10.VI.1965, G. Buckingham |
| *Eulonchus sapphirinus* | 019055 | unknown | CAS | USA, California, El Dorado County, Blodgett Forest Research Station, 16.1 km E Georgetown, [38.908, -120.654], 10.VI.1965, G. Buckingham |
| *Eulonchus sapphirinus* | 019081 | unknown | CAS | USA, California, Alpine County, Ebbetts Pass, [38.544, -119.812], 2661 m, 18.VII.1980, J. A. Powell |
| *Eulonchus sapphirinus* | 019082 | unknown | CAS | USA, California, Alpine County, Ebbetts Pass, [38.544, -119.812], 2661 m, 18.VII.1980, J. A. Powell |
| *Eulonchus sapphirinus* | 019083 | unknown | CAS | USA, California, El Dorado County, Blodgett Forest Research Station, 20.9 km E Georgetown, Bache Meadow, [38.907, -120.598], 28.V.1977 to 29.V.1977, Malaise trap, J. Yoakley |
| *Eulonchus sapphirinus* | 019084 | unknown | CAS | USA, California, El Dorado County, Blodgett Forest Research Station, 20.9 km E Georgetown, Bache Meadow, [38.907, -120.598], 28.V.1977 to 29.V.1977, Malaise trap, J. Yoakley |
| *Eulonchus sapphirinus* | 019085 | unknown | CAS | USA, California, El Dorado County, Blodgett Forest Research Station, 20.9 km E Georgetown, Bache Meadow, [38.907, -120.598], 28.V.1977 to 29.V.1977, Malaise trap, J. Yoakley |
| *Eulonchus sapphirinus* | 019087 | unknown | CAS | USA, California, Marin County, 3.2 km south east of Inverness, Inverness Ridge, [38.086, -122.825], 244 to 317 m, 10.V.1974, D. S. Green |
| *Eulonchus sapphirinus* | 019100 | unknown | CAS | USA, California, El Dorado County, Blodgett Forest Research Station, 20.9 km east of Georgetown, [38.909, -120.661], 1300 m, 4.VII.1981, J. A. Powell |
| *Eulonchus sapphirinus* | 019117 | unknown | CAS | USA, California, Mendocino County, NCCRP (Northern California Coast Range Preserve), 8.1 km. N. of Branscomb, [39.77, -123.628], 26.V.1976, J. A. Powell |
| *Eulonchus sapphirinus* | 019118 | unknown | CAS | USA, California, Mendocino County, NCCRP (Northern California Coast Range Preserve), 8.1 km. N. of Branscomb, [39.77, -123.628], 26.V.1976, J. A. Powell |
| *Eulonchus sapphirinus* | 019119 | unknown | CAS | USA, California, Mendocino County, NCCRP (Northern California Coast Range Preserve), 8.1 km. N. of Branscomb, [39.77, -123.628], 26.V.1976, J. A. Powell |
| *Eulonchus sapphirinus* | 019120 | unknown | CAS | USA, California, Mendocino County, NCCRP (Northern California Coast Range Preserve), 8.1 km. N. of Branscomb, [39.77, -123.628], 26.V.1976, J. A. Powell |
| *Eulonchus sapphirinus* | 019121 | unknown | CAS | USA, California, Mendocino County, NCCRP (Northern California Coast Range Preserve), 8.1 km. N. of Branscomb, [39.77, -123.628], 26.V.1976, J. A. Powell |
| *Eulonchus sapphirinus* | 019126 | unknown | CAS | USA, California, Mendocino County, NCCRP (Northern California Coast Range Preserve), 8.1 km. N. of Branscomb, [39.77, -123.628], 26.V.1976, J. A. Powell |
| *Eulonchus sapphirinus* | 019127 | unknown | CAS | USA, California, Mendocino County, NCCRP (Northern California Coast Range Preserve), 8.1 km. N. of Branscomb, [39.77, -123.628], 26.V.1976, J. A. Powell |
| *Eulonchus sapphirinus* | 019128 | unknown | CAS | USA, California, Mendocino County, NCCRP (Northern California Coast Range Preserve), 8.1 km. N. of Branscomb, [39.77, -123.628], 26.V.1976, J. A. Powell |
| *Eulonchus sapphirinus* | 019129 | unknown | CAS | USA, California, Mendocino County, NCCRP (Northern California Coast Range Preserve), 8.1 km. N. of Branscomb, [39.77, -123.628], 26.V.1976, J. A. Powell |
| *Eulonchus sapphirinus* | 019131 | unknown | CAS | USA, California, Mendocino County, NCCRP (Northern California Coast Range Preserve), 8.1 km. N. of Branscomb, [39.77, -123.628], 26.V.1976, J. A. Powell |
| *Eulonchus sapphirinus* | 019132 | unknown | CAS | USA, California, Mendocino County, NCCRP (Northern California Coast Range Preserve), 8.1 km. N. of Branscomb, [39.77, -123.628], 26.V.1976, J. A. Powell |
| *Eulonchus sapphirinus* | 019133 | unknown | CAS | USA, California, Mendocino County, NCCRP (Northern California Coast Range Preserve), 8.1 km. N. of Branscomb, [39.77, -123.628], 26.V.1976, J. A. Powell |
| *Eulonchus sapphirinus* | 019134 | unknown | CAS | USA, California, Mendocino County, NCCRP (Northern California Coast Range Preserve), 8.1 km. N. of Branscomb, [39.77, -123.628], 26.V.1976, J. A. Powell |
| *Eulonchus sapphirinus* | 019135 | unknown | CAS | USA, California, Mendocino County, NCCRP (Northern California Coast Range Preserve), 8.1 km. N. of Branscomb, [39.77, -123.628], 26.V.1976, J. A. Powell |
| *Eulonchus sapphirinus* | 019136 | unknown | CAS | USA, California, Mendocino County, NCCRP (Northern California Coast Range Preserve), 8.1 km. N. of Branscomb, [39.77, -123.628], 26.V.1976, J. A. Powell |
| *Eulonchus sapphirinus* | 019137 | unknown | CAS | USA, California, Mendocino County, NCCRP (Northern California Coast Range Preserve), 8.1 km. N. of Branscomb, [39.77, -123.628], 26.V.1976, J. A. Powell |
| *Eulonchus sapphirinus* | 019138 | unknown | CAS | USA, California, Mendocino County, NCCRP (Northern California Coast Range Preserve), 8.1 km. N. of Branscomb, [39.77, -123.628], 26.V.1976, J. A. Powell |
| *Eulonchus sapphirinus* | 019139 | unknown | CAS | USA, California, Mendocino County, NCCRP (Northern California Coast Range Preserve), 8.1 km. N. of Branscomb, [39.77, -123.628], 26.V.1976, J. A. Powell |
| *Eulonchus sapphirinus* | 019140 | unknown | CAS | USA, California, Mendocino County, NCCRP (Northern California Coast Range Preserve), 8.1 km. N. of Branscomb, [39.77, -123.628], 26.V.1976, J. A. Powell |
| *Eulonchus sapphirinus* | 019141 | unknown | CAS | USA, California, Mendocino County, NCCRP (Northern California Coast Range Preserve), 8.1 km. N. of Branscomb, [39.77, -123.628], 26.V.1976, J. A. Powell |
| *Eulonchus sapphirinus* | 019142 | unknown | CAS | USA, California, Mendocino County, NCCRP (Northern California Coast Range Preserve), 8.1 km. N. of Branscomb, [39.77, -123.628], 26.V.1976, J. A. Powell |
| *Eulonchus sapphirinus* | 019143 | unknown | CAS | USA, California, Mendocino County, NCCRP (Northern California Coast Range Preserve), 8.1 km. N. of Branscomb, [39.77, -123.628], 26.V.1976, J. A. Powell |
| *Eulonchus sapphirinus* | 019144 | unknown | CAS | USA, California, Mendocino County, NCCRP (Northern California Coast Range Preserve), 8.1 km. N. of Branscomb, [39.77, -123.628], 26.V.1976, J. A. Powell |
| *Eulonchus sapphirinus* | 019150 | unknown | CAS | USA, California, Humboldt County, 3.2 km W Briceland, [40.108, -123.938], 21.V.1976, J. A. Chemsak |
| *Eulonchus sapphirinus* | 019205 | unknown | CAS | USA, California, Mendocino County, NCCRP (Northern California Coast Range Preserve), 4.8 km N of Branscomb, [39.737, -123.626], 18.V.1984 to 21.V.1984, E. I. Schlinger |
| *Eulonchus sapphirinus* | 019206 | unknown | CAS | USA, California, Mendocino County, NCCRP (Northern California Coast Range Preserve), 4.8 km N of Branscomb, [39.737, -123.626], 18.V.1984 to 21.V.1984, E. I. Schlinger |
| *Eulonchus sapphirinus* | 019416 | unknown | CAS | USA, California, El Dorado County, Pino Grande, NW of Lake Edson, [38.899, -120.607], 29.VI.1967, hand netted, S.R. Kutcher |
| *Eulonchus sapphirinus* | 019417 | unknown | CAS | USA, California, El Dorado County, Pino Grande, NW of Lake Edson, [38.899, -120.607], 29.VI.1967, hand netted, S.R. Kutcher |
| *Eulonchus sapphirinus* | 019418 | unknown | CAS | USA, California, El Dorado County, Pino Grande, NW of Lake Edson, [38.899, -120.607], 29.VI.1967, hand netted, S.R. Kutcher |
| *Eulonchus sapphirinus* | 019419 | unknown | CAS | USA, California, El Dorado County, Blodgett Forest Research Station, 20.9 km east of Georgetown, [38.909, -120.661], 1300 m, 24.VI.1967, S.R. Kutcher |
| *Eulonchus sapphirinus* | 019420 | unknown | CAS | USA, California, El Dorado County, Blodgett Forest Research Station, 20.9 km east of Georgetown, [38.909, -120.661], 1300 m, 24.VI.1967, S.R. Kutcher |
| *Eulonchus sapphirinus* | 019421 | unknown | CAS | USA, California, El Dorado County, Blodgett Forest Research Station, 20.9 km east of Georgetown, [38.909, -120.661], 1300 m, 24.VI.1967, S.R. Kutcher |
| *Eulonchus sapphirinus* | 019422 | unknown | CAS | USA, California, El Dorado County, Blodgett Forest Research Station, 20.9 km east of Georgetown, [38.909, -120.661], 1300 m, 24.VI.1967, S.R. Kutcher |
| *Eulonchus sapphirinus* | 019423 | unknown | CAS | USA, California, El Dorado County, Blodgett Forest Research Station, 20.9 km east of Georgetown, [38.909, -120.661], 1300 m, 24.VI.1967, S.R. Kutcher |
| *Eulonchus sapphirinus* | 019424 | unknown | CAS | USA, California, El Dorado County, Blodgett Forest Research Station, 20.9 km east of Georgetown, [38.909, -120.661], 1300 m, 24.VI.1967, S.R. Kutcher |
| *Eulonchus sapphirinus* | 019425 | unknown | CAS | USA, California, El Dorado County, Blodgett Forest Research Station, 20.9 km east of Georgetown, [38.909, -120.661], 1300 m, 24.VI.1967, S.R. Kutcher |
| *Eulonchus sapphirinus* | 019426 | unknown | CAS | USA, California, El Dorado County, Blodgett Forest Research Station, 20.9 km east of Georgetown, [38.909, -120.661], 1300 m, 24.VI.1967, S.R. Kutcher |
| *Eulonchus sapphirinus* | 019427 | unknown | CAS | USA, California, El Dorado County, Blodgett Forest Research Station, 20.9 km east of Georgetown, [38.909, -120.661], 1300 m, 24.VI.1967, S.R. Kutcher |
| *Eulonchus sapphirinus* | 019428 | unknown | CAS | USA, California, El Dorado County, Blodgett Forest Research Station, 20.9 km east of Georgetown, [38.909, -120.661], 1300 m, 20.VI.1967, M.O. Way |
| *Eulonchus sapphirinus* | 019589 | unknown | CAS | USA, Washington, Clallam County, Bogachiel, Bogachiel River, [47.902, -124.195], 29.VI.1974, P. H. Arnaud, Jr. |
| *Eulonchus sapphirinus* | 019590 | unknown | CAS | USA, Washington, Clallam County, Bogachiel, Bogachiel River, [47.902, -124.195], 29.VI.1974, P. H. Arnaud, Jr. |
| *Eulonchus sapphirinus* | 019591 | unknown | CAS | USA, Washington, Clallam County, Bogachiel, Bogachiel River, [47.902, -124.195], 29.VI.1974, P. H. Arnaud, Jr. |
| *Eulonchus sapphirinus* | 019595 | unknown | CAS | USA, California, Del Norte County, Gasquet, [41.846, -123.97], 20.VI.1974, P. H. Arnaud, Jr. |
| *Eulonchus sapphirinus* | 019597 | unknown | CAS | USA, California, El Dorado County, Blodgett Forest Research Station, 19.3 km east of Georgetown, [38.909, -120.661], 1300 m, 20.V.1973, Wharton |
| *Eulonchus sapphirinus* | 019599 | unknown | CAS | USA, California, El Dorado County, Blodgett Forest Research Station, 12.9 km E of Georgetown, [38.906, -120.69], 30.VI.1967, C. O. Dudley |
| *Eulonchus sapphirinus* | 019715 | unknown | CAS | USA, Washington, Jefferson County, Olympic National Park, Dose Forks, [47.744, -123.226], 469 m, 24.VII.1953, C. P. Alexander |
| *Eulonchus sapphirinus* | 019716 | unknown | CAS | USA, Washington, Jefferson County, Olympic National Park, Dose Forks, [47.744, -123.226], 469 m, 24.VII.1953, C. P. Alexander |
| *Eulonchus sapphirinus* | 019730 | unknown | CAS | USA, Washington, Snohomish County, Miner's Ridge, [48.214, -121.011], 3.VIII.1965, L. E. Eighme |
| *Eulonchus sapphirinus* | 019731 | unknown | CAS | USA, Washington, Snohomish County, Miner's Ridge, [48.214, -121.011], 3.VIII.1965, L. E. Eighme |
| *Eulonchus sapphirinus* | 019732 | unknown | CAS | USA, Washington, Snohomish County, Miner's Ridge, [48.214, -121.011], 3.VIII.1965, L. E. Eighme |
| *Eulonchus sapphirinus* | 019737 | unknown | CAS | USA, California, Trinity County, Shasta National Forest, Swift Creek, [40.367, -123.383], 914 m, 30.V.1972, T. Griswold |
| *Eulonchus sapphirinus* | 019738 | unknown | CAS | USA, California, Trinity County, Shasta National Forest, Swift Creek, [40.367, -123.383], 914 m, 30.V.1972, T. Griswold |
| *Eulonchus sapphirinus* | 019739 | unknown | CAS | USA, California, Trinity County, Shasta National Forest, Swift Creek, [40.367, -123.383], 1798 m, 26.VI.1972, hand netted, T. Griswold |
| *Eulonchus sapphirinus* | 019740 | unknown | CAS | USA, California, Trinity County, Shasta National Forest, Swift Creek, [40.367, -123.383], 1798 m, 26.VI.1972, hand netted, T. Griswold |
| *Eulonchus sapphirinus* | 019741 | unknown | CAS | USA, California, Trinity County, Shasta National Forest, Swift Creek, [40.367, -123.383], 1798 m, 26.VI.1972, hand netted, T. Griswold |
| *Eulonchus sapphirinus* | 019742 | unknown | CAS | USA, California, Trinity County, Shasta National Forest, Gibson Meadow, [40.954, -122.849], 1768 m, 27.VII.1973, T. Griswold |
| *Eulonchus sapphirinus* | 019743 | unknown | CAS | USA, California, Trinity County, Shasta National Forest, Gibson Meadow, [40.954, -122.849], 1768 m, 27.VII.1973, T. Griswold |
| *Eulonchus sapphirinus* | 019744 | unknown | CAS | USA, California, Trinity County, Shasta National Forest, Gibson Meadow, [40.954, -122.849], 1768 m, 27.VII.1973, T. Griswold |
| *Eulonchus sapphirinus* | 019745 | unknown | CAS | USA, California, Trinity County, Shasta National Forest, Gibson Meadow, [40.954, -122.849], 1768 m, 27.VII.1973, T. Griswold |
| *Eulonchus sapphirinus* | 019746 | unknown | CAS | USA, California, Trinity County, Shasta National Forest, Gibson Meadow, [40.954, -122.849], 1768 m, 27.VII.1973, T. Griswold |
| *Eulonchus sapphirinus* | 019747 | unknown | CAS | USA, California, Trinity County, Shasta National Forest, Gibson Meadow, [40.954, -122.849], 1768 m, 27.VII.1973, T. Griswold |
| *Eulonchus sapphirinus* | 019748 | unknown | CAS | USA, California, Siskiyou County, Klamath National Forest, Bell Echo Camp, [41.875, -123.622], 1676 m, 1.VIII.1971, J. Kraemer |
| *Eulonchus sapphirinus* | 019749 | unknown | CAS | USA, California, Siskiyou County, Klamath National Forest, Bell Echo Camp, [41.875, -123.622], 1676 m, 1.VIII.1971, J. Kraemer |
| *Eulonchus sapphirinus* | 019750 | unknown | CAS | USA, California, Siskiyou County, Klamath National Forest, Bell Echo Camp, [41.875, -123.622], 1676 m, 1.VIII.1971, J. Kraemer |
| *Eulonchus sapphirinus* | 019751 | unknown | CAS | USA, California, Siskiyou County, Klamath National Forest, Bell Echo Camp, [41.875, -123.622], 1676 m, 1.VIII.1971, J. Kraemer |
| *Eulonchus sapphirinus* | 019752 | unknown | CAS | USA, California, Siskiyou County, Klamath National Forest, Bell Echo Camp, [41.875, -123.622], 1676 m, 1.VIII.1971, J. Kraemer |
| *Eulonchus sapphirinus* | 019753 | unknown | CAS | USA, California, Klamath National Forest, Young's Valley, [41.875, -123.635], 1402 m, 6.VIII.1970, M. Butler |
| *Eulonchus sapphirinus* | 019754 | unknown | CAS | USA, California, Trinity County, Shasta National Forest, Granite Creek, [40.953, -122.853], 1524 m, 27.VII.1973, T. Griswold |
| *Eulonchus sapphirinus* | 019756 | unknown | CAS | USA, California, Trinity County, Trinity National Forest, Swift Creek, [40.369, -123.382], 1890 m, 16.VII.1972, T. Griswold |
| *Eulonchus sapphirinus* | 019757 | unknown | CAS | USA, California, Trinity County, Trinity National Forest, Trinity River, Stuart Fork, [40.959, -122.951], 1067 m, 12.VI.1974, T. Griswold |
| *Eulonchus sapphirinus* | 019758 | unknown | CAS | USA, California, Trinity County, Trinity National Forest, Swift Creek, [40.367, -123.383], 2743 m, 30.V.1972, T. Griswold |
| *Eulonchus sapphirinus* | 019806 | female | CAS | USA, California, Shasta County, Junction hwy 89 & 299, [40.937, -121.614], 18.VI.1960, hand netted, A. Edwards, J. G. Edwards |
| *Eulonchus sapphirinus* | 019807 | male | CAS | USA, California, Shasta County, Junction hwy 89 & 299, [40.937, -121.614], 18.VI.1960, hand netted, A. Edwards, J. G. Edwards |
| *Eulonchus sapphirinus* | 019841 | unknown | CAS | USA, California, Marin County, Alpine Lake, [37.946, -122.629], 30.V.1972, hand netted, D. Tilles |
| *Eulonchus sapphirinus* | 019842 | unknown | CAS | USA, California, Marin County, Alpine Lake, [37.946, -122.629], 30.V.1972, hand netted, D. Tilles |
| *Eulonchus sapphirinus* | 019847 | unknown | CAS | USA, California, Sierra County, Downieville, [39.56, -120.828], 29.V.1965, H. G. Real |
| *Eulonchus sapphirinus* | 019881 | unknown | CAS | USA, Utah, Salt Lake County, City Creek Canyon, [40.776, -111.885], 13.VI.1951, Y. Sedman |
| *Eulonchus sapphirinus* | 020063 | unknown | CAS | USA, California, El Dorado County, Sugar Pine Point State Park, 2 mi. west Lake Tahoe, General Creek, General Creek, [39.044, -120.147], 1.VII.1993 to 2.VII.1993 |
| *Eulonchus sapphirinus* | 020064 | unknown | CAS | USA, California, El Dorado County, Sugar Pine Point State Park, 2 mi. west Lake Tahoe, General Creek, General Creek, [39.044, -120.147], 1.VII.1993 to 2.VII.1993 |
| *Eulonchus sapphirinus* | 020065 | unknown | CAS | USA, California, El Dorado County, Sugar Pine Point State Park, 2 mi. west Lake Tahoe, General Creek, General Creek, [39.044, -120.147], 1.VII.1993 to 2.VII.1993 |
| *Eulonchus sapphirinus* | 020066 | unknown | CAS | USA, California, El Dorado County, Sugar Pine Point State Park, 2 mi. west Lake Tahoe, General Creek, General Creek, [39.044, -120.147], 1.VII.1993 to 2.VII.1993 |
| *Eulonchus sapphirinus* | 020067 | unknown | CAS | USA, California, El Dorado County, Sugar Pine Point State Park, 2 mi. west Lake Tahoe, General Creek, General Creek, [39.044, -120.147], 1.VII.1993 to 2.VII.1993 |
| *Eulonchus sapphirinus* | 020068 | unknown | CAS | USA, California, El Dorado County, Sugar Pine Point State Park, 2 mi. west Lake Tahoe, General Creek, General Creek, [39.044, -120.147], 1.VII.1993 to 2.VII.1993 |
| *Eulonchus sapphirinus* | 020073 | unknown | CAS | USA, California, El Dorado County, 1.2 Km of Highway 50, Wrights Lake Road, [38.787, -120.211], 1700 m, 24.VI.2006 |
| *Eulonchus sapphirinus* | 020074 | unknown | CAS | USA, California, El Dorado County, 1.2 Km of Highway 50, Wrights Lake Road, [38.787, -120.211], 1700 m, 24.VI.2006 |
| *Eulonchus sapphirinus* | 020076 | unknown | CAS | USA, California, El Dorado County, Big Trees State Park, south grove, Beaver Creek, beaver creek, [38.262, -120.258], 22.V.2007 to 11.V.2007 |
| *Eulonchus sapphirinus* | 020077 | unknown | CAS | USA, California, Del Norte, Six Rivers National Forest, Darlingtonia (Highway 199), [41.839, -123.946], 120 m, 1.VI.2009 |
| *Eulonchus sapphirinus* | 020078 | unknown | CAS | USA, California, Amador County, Indian Grinding Rock State Park, firebreak near envtl camp, [38.419, -120.635], 24.IV.2007 to 24.V.2007 |
| *Eulonchus sapphirinus* | 020086 | unknown | CAS | USA, California, Amador County, 12.9 km East of Ham's Station, [38.543, -120.524], 7.VII.1991 |
| *Eulonchus sapphirinus* | 020088 | unknown | CAS | USA, California, El Dorado County, 4.7 km S of Kyburz, China Flat, [38.732, -120.298], 1280 m, 16.VI.1989 |
| *Eulonchus sapphirinus* | 020089 | unknown | CAS | USA, California, El Dorado County, El Dorado National Forest, Wright's Lake entry road, [38.811, -120.244], 2002 m, 20.VI.2006 |
| *Eulonchus sapphirinus* | 020141 | unknown | CAS | USA, Oregon, Marion, 4.8 km East of Idanha, [44.702, -122.018], 8.VI.1969 |
| *Eulonchus sapphirinus* | 020156 | unknown | CAS | USA, Orgeon, Clatsop County, South slope of Saddle Mountain, [44.516, -121.884], 762 to 1000 m, 14.VI.1969 |
| *Eulonchus sapphirinus* | 020157 | unknown | CAS | USA, Orgeon, Clatsop County, South slope of Saddle Mountain, [44.516, -121.884], 762 to 1000 m, 14.VI.1969 |
| *Eulonchus sapphirinus* | 020158 | unknown | CAS | USA, Orgeon, Clatsop County, South slope of Saddle Mountain, [44.516, -121.884], 762 to 1000 m, 14.VI.1969 |
| *Eulonchus sapphirinus* | 020160 | unknown | CAS | USA, Orgeon, Clatsop County, South slope of Saddle Mountain, [44.516, -121.884], 762 to 1000 m, 14.VI.1969 |
| *Eulonchus sapphirinus* | 020161 | unknown | CAS | USA, Orgeon, Clatsop County, South slope of Saddle Mountain, [44.516, -121.884], 762 to 1000 m, 14.VI.1969 |
| *Eulonchus sapphirinus* | 020162 | unknown | CAS | USA, Orgeon, Clatsop County, South slope of Saddle Mountain, [44.516, -121.884], 762 to 1000 m, 14.VI.1969 |
| *Eulonchus sapphirinus* | 020169 | unknown | CAS | USA, Oregon, Lane County, Whittaker Creek, Bounds Creek Road, [43.967, -123.683], 270 m, 29.VII.1999 |
| *Eulonchus sapphirinus* | 020188 | unknown | CAS | USA, Washington, Yakima County, Hemlock Forest, 8 km SSW of Goose Prairie, 1 km South of Junction of 1800, Forest Road 1808, [46.827, -121.306], 1090 m, 14.VII.2004, C.J. Borkent |
| *Eulonchus sapphirinus* | 020190 | unknown | CAS | USA, Washington, Clallam County, Olympic National Park, 0.5km North East of Fairholm, near entrance of Fairholm Campground, [48.07, -123.919], 200 m, 24.VI.2005, C.J. Borkent |
| *Eulonchus sapphirinus* | 020210 | unknown | CAS | USA, California, Del Norte County, Six Rivers National Forest, Near Bear Basin Outlook, forest rout 16N02, [41.802, -123.737], 6 m, 3.VI.2009 to 24.VII.2009 |
| *Eulonchus sapphirinus* | 020211 | female | CAS | USA, Utah, Wasatch County, Uinta National Forest, Currant Creek Valley, [401.329, -111.069], 2438 m, 24.VI.1917 |
| *Eulonchus sapphirinus* | 020212 | unknown | CAS | USA, California, Del Norte County, Six Rivers National Forest, Near Bear Basin Outlook, forest rout 16N02, [41.802, -123.737], 6 m, 3.VI.2009 to 24.VII.2009 |
| *Eulonchus sapphirinus* | 020213 | unknown | CAS | USA, California, Calaveras County, Calaveras Big Trees State Park, [38.275, -120.31], 25.V.2010 to 26.VI.2010 |
| *Eulonchus sapphirinus* | 020214 | unknown | CAS | USA, California |
| *Eulonchus sapphirinus* | 020215 | female | CAS | USA, Utah, Wasatch County, Uinta National Forest, Currant Creek Valley, [401.329, -111.069], 2438 m, 24.VI.1917 |
| *Eulonchus sapphirinus* | 020216 | unknown | CAS | USA, Utah, Uinta County, Uinta National Forest, Currant Creek Valley, 2438 m, 25.V.1917 |
| *Eulonchus sapphirinus* | 020217 | female | CAS | USA, Oregon, Lincoln County, Mary's Peak, [44.504, -123.553], 1.VII.1982 |
| *Eulonchus sapphirinus* | 020219 | female | CAS | USA, Washington, Yakima County, Indian Flat Campground, [46.983, -121.15], 823 m, 25.VII.1996 |
| *Eulonchus sapphirinus* | 020220 | female | CAS | USA, Washington, Yakima County, Indian Flat Campground, [46.983, -121.15], 823 m, 25.VII.1996 |
| *Eulonchus sapphirinus* | 012263 | female | CAS | USA, California, Sierra Co., GOLD Lake, Gold Lake, [39.678, -120.657], 1.VII.1949, L.W. Issak |
| *Eulonchus sapphirinus* | 012264 | female | CAS | USA, California, Plumas County, Meadow Valley, [39.93, -121.061], 1067 to 1219 m, 5.VI.1924, E. C. Van Dyke |
| *Eulonchus sapphirinus* | 012265 | female | CAS | USA, California, Nevada County, Sagehen near Hobart Mills, [39.407, -120.19], 21.VI.1954, P. D. Hurd |
| *Eulonchus sapphirinus* | 012266 | female | CAS | USA, California, Nevada County, Sagehen near Hobart Mills, [39.407, -120.19], 21.VI.1954, P. D. Hurd |
| *Eulonchus sapphirinus* | 012267 | female | CAS | USA, California, Plumas Co., 13.VI.1913, F. W. Nunenmacher |
| *Eulonchus sapphirinus* | 012268 | female | CAS | USA, California, Plumas Co, 6.4 km West of Quincy, 19.VI.1949, L. L. Jensen |
| *Eulonchus sapphirinus* | 012269 | female | UCDC | USA, California, Nevada County, Sagehen near Hobart Mills, [39.407, -120.19], 25.VI.1954, R. M. Bohart |
| *Eulonchus sapphirinus* | 012270 | female | CAS | USA, California, Shasta County, 15.VII.1935, W. H. Lange |
| *Eulonchus sapphirinus* | 012271 | female | UCDC | USA, California, Nevada County, Sagehen near Hobart Mills, [39.407, -120.19], 25.VI.1954, R. M. Bohart |
| *Eulonchus sapphirinus* | 012272 | female | UCDC | USA, California, Nevada County, Sagehen near Hobart Mills, [39.407, -120.19], 25.VI.1954, R. M. Bohart |
| *Eulonchus sapphirinus* | 012273 | female | UCDC | USA, California, Nevada County, Sagehen near Hobart Mills, [39.407, -120.19], 25.VI.1954, R. M. Bohart |
| *Eulonchus sapphirinus* | 012274 | female | UCDC | USA, California, Nevada County, Sagehen near Hobart Mills, [39.407, -120.19], 25.VI.1954, R. M. Bohart |
| *Eulonchus sapphirinus* | 012275 | female | UCDC | USA, California, Nevada County, Sagehen near Hobart Mills, [39.407, -120.19], 25.VI.1954, R. M. Bohart |
| *Eulonchus sapphirinus* | 012276 | female | UCDC | USA, California, Nevada County, Sagehen near Hobart Mills, [39.407, -120.19], 25.VI.1954, R. M. Bohart |
| *Eulonchus sapphirinus* | 012277 | female | UCDC | USA, California, Nevada County, Sagehen near Hobart Mills, [39.407, -120.19], 25.VI.1954, R. M. Bohart |
| *Eulonchus sapphirinus* | 012278 | female | UCDC | USA, California, Nevada County, Sagehen near Hobart Mills, [39.407, -120.19], 25.VI.1954, R. M. Bohart |
| *Eulonchus sapphirinus* | 012279 | female | UCDC | USA, California, Nevada County, Sagehen near Hobart Mills, [39.407, -120.19], 25.VI.1954, R. M. Bohart |
| *Eulonchus sapphirinus* | 012280 | female | UCDC | USA, California, Nevada County, Sagehen near Hobart Mills, [39.407, -120.19], 25.VI.1954, R. M. Bohart |
| *Eulonchus sapphirinus* | 012281 | female | CAS | USA, California, Lassen County, Bridge Creek Camp, [45.785, -115.213], 9.VII.1949, E. I. Schlinger |
| *Eulonchus sapphirinus* | 012283 | female | CAS | USA, California, El Dorado Co., China Flat, 28.VI.1948, C. D. MacNeill |
| *Eulonchus sapphirinus* | 012284 | female | CAS | USA, California, Lassen County, Susan River Camp, [40.494, -121.093], 10.VII.1949, E. I. Schlinger |
| *Eulonchus sapphirinus* | 012285 | female | CAS | USA, California, Lassen County, Susan River Camp, [40.494, -121.093], 10.VII.1949, E. I. Schlinger |
| *Eulonchus sapphirinus* | 012286 | female | CAS | USA, California, Plumas County, Buck’s Lake, [39.868, -121.174], 1.VII.1949, E. I. Schlinger |
| *Eulonchus sapphirinus* | 012287 | female | CAS | USA, California, Plumas County, Buck’s Lake, [39.868, -121.174], 1.VII.1949, E. I. Schlinger |
| *Eulonchus sapphirinus* | 012288 | female | CAS | USA, California, Plumas County, Buck’s Lake, [39.868, -121.174], 1.VII.1949, E. I. Schlinger |
| *Eulonchus sapphirinus* | 012289 | female | CAS | USA, California, Sierra County, Gold Lake, [39.678, -120.657], 14.VII.1921, C. L. Fox |
| *Eulonchus sapphirinus* | 012290 | female | CAS | USA, California, Lassen Co. , Norval Flats, 20.VI.1920, J. O. Martin |
| *Eulonchus sapphirinus* | 012291 | female | CAS | USA, California, El Dorado Co, Strawberry, 7.VI.1948 |
| *Eulonchus sapphirinus* | 012292 | female | CAS | USA, California, El Dorado Co, Strawberry, 7.VI.1948 |
| *Eulonchus sapphirinus* | 012293 | female | CAS | USA, California, Shasta County, Moose Camp, [40.859, -121.847], 19.VI.1954, E. I. Schlinger |
| *Eulonchus sapphirinus* | 012294 | female | CAS | USA, California, Shasta County, 13.VII.1935, W. H. Lange |
| *Eulonchus sapphirinus* | 012295 | female | CAS | USA, California, Plumas County, Meadow Valley, [39.929, -121.061], 2.VI.1924 |
| *Eulonchus sapphirinus* | 012296 | female | CAS | USA, California, Plumas Co., Onion Valley, [39.793, -120.879], 7.VII.1949, E. I. Schlinger |
| *Eulonchus sapphirinus* | 012297 | female | CAS | USA, California, Shasta County, Lake Eiler, [40.727, -121.568], 9.VII.1947, R. L. Usinger |
| *Eulonchus sapphirinus* | 012298 | female | CAS | USA, Oregon, Steen mountains, Blitzen River, 25.VI.1903, G. P. Engelhardt |
| *Eulonchus sapphirinus* | 012299 | male | CAS | USA, California, Lassen County, Bridge Creek Camp, [45.785, -115.213], 9.VII.1949, R. C. Bechtel |
| *Eulonchus sapphirinus* | 012300 | male | CAS | USA, California, Lassen County, Bridge Creek Camp, [45.785, -115.213], 9.VII.1949, R. C. Bechtel |
| *Eulonchus sapphirinus* | 012301 | male | CAS | USA, California, Lassen County, Bridge Creek Camp, [45.785, -115.213], 9.VII.1949, R. C. Bechtel |
| *Eulonchus sapphirinus* | 012302 | male | CAS | USA, California, Lassen County, Bridge Creek Camp, [45.785, -115.213], 9.VII.1949, R. C. Bechtel |
| *Eulonchus sapphirinus* | 012303 | male | CAS | USA, California, Lassen County, Bridge Creek Camp, [45.785, -115.213], 9.VII.1949, R. C. Bechtel |
| *Eulonchus sapphirinus* | 012304 | male | CAS | USA, California, Plumas County, Buck’s Lake, [39.868, -121.174], 1.VII.1949, E. I. Schlinger |
| *Eulonchus sapphirinus* | 012305 | male | CAS | USA, California, Plumas County, Buck’s Lake, [39.868, -121.174], 1.VII.1949, E. I. Schlinger |
| *Eulonchus sapphirinus* | 012306 | male | CAS | USA, California, Plumas County, Buck’s Lake, [39.868, -121.174], 1.VII.1949, E. I. Schlinger |
| *Eulonchus sapphirinus* | 012307 | male | CAS | USA, California, Plumas County, Buck’s Lake, [39.868, -121.174], 1.VII.1949, E. I. Schlinger |
| *Eulonchus sapphirinus* | 012308 | male | CAS | USA, California, Shasta County, Moose Camp, [40.859, -121.847], 19.VI.1954, E. I. Schlinger |
| *Eulonchus sapphirinus* | 012309 | male | CAS | USA, California, Shasta County, Moose Camp, [40.859, -121.847], 19.VI.1954, E. I. Schlinger |
| *Eulonchus sapphirinus* | 012310 | male | CAS | USA, California, Shasta County, Moose Camp, [40.859, -121.847], 19.VI.1954, E. I. Schlinger |
| *Eulonchus sapphirinus* | 012311 | male | CAS | USA, California, Shasta County, 15.VII.1935, W. H. Lange |
| *Eulonchus sapphirinus* | 012312 | male | CAS | USA, California, Lassen Co, 24.VI.1935, W. H. Lange |
| *Eulonchus sapphirinus* | 012313 | male | CAS | USA, California, Lassen County, Bridge Creek Camp, [45.785, -115.213], 12.VII.1954, R. C. Bechtel |
| *Eulonchus sapphirinus* | 012314 | male | CAS | USA, California, Plumas County, Meadow Valley, [39.93, -121.061], 1067 to 1219 m, 5.VI.1924, E. C. Van Dyke |
| *Eulonchus sapphirinus* | 012315 | male | CAS | USA, California, Truckee, [39.328, -120.184], 21.VI.1937, Van Duzee |
| *Eulonchus sapphirinus* | 012316 | male | CAS | USA, California, Sierra Co., GOLD Lake, Gold Lake, [39.678, -120.657], 1.VII.1949, L.W. Issak |
| *Eulonchus sapphirinus* | 012317 | male | CAS | USA, California, Sierra Co., GOLD Lake, Gold Lake, [39.678, -120.657], 1.VII.1949, L.W. Issak |
| *Eulonchus sapphirinus* | 012318 | male | CAS | USA, California, Sierra County, Gold Lake, [39.678, -120.657], 10.VII.1921, C. L. Fox |
| *Eulonchus sapphirinus* | 012319 | male | UCDC | USA, California, Nevada County, Sagehen near Hobart Mills, [39.407, -120.19], 25.VI.1954, R. M. Bohart |
| *Eulonchus sapphirinus* | 012320 | male | UCDC | USA, California, Nevada County, Sagehen near Hobart Mills, [39.407, -120.19], 25.VI.1954, R. M. Bohart |
| *Eulonchus sapphirinus* | 012321 | male | UCDC | USA, California, Nevada County, Sagehen near Hobart Mills, [39.407, -120.19], 25.VI.1954, R. M. Bohart |
| *Eulonchus sapphirinus* | 012322 | male | UCDC | USA, California, Nevada County, Sagehen near Hobart Mills, [39.407, -120.19], 25.VI.1954, R. M. Bohart |
| *Eulonchus sapphirinus* | 012323 | male | UCDC | USA, California, Nevada County, Sagehen near Hobart Mills, [39.407, -120.19], 25.VI.1954, R. M. Bohart |
| *Eulonchus sapphirinus* | 012324 | male | UCDC | USA, California, Nevada County, Sagehen near Hobart Mills, [39.407, -120.19], 25.VI.1954, R. M. Bohart |
| *Eulonchus sapphirinus* | 012325 | male | UCDC | USA, California, Nevada County, Sagehen near Hobart Mills, [39.407, -120.19], 25.VI.1954, R. M. Bohart |
| *Eulonchus sapphirinus* | 012326 | male | UCDC | USA, California, Nevada County, Sagehen near Hobart Mills, [39.407, -120.19], 25.VI.1954, R. M. Bohart |
| *Eulonchus sapphirinus* | 012327 | male | UCDC | USA, California, Nevada County, Sagehen near Hobart Mills, [39.407, -120.19], 25.VI.1954, R. M. Bohart |
| *Eulonchus sapphirinus* | 012328 | male | UCDC | USA, California, Nevada County, Sagehen near Hobart Mills, [39.407, -120.19], 25.VI.1954, R. M. Bohart |
| *Eulonchus sapphirinus* | 012329 | male | UCDC | USA, California, Nevada County, Sagehen near Hobart Mills, [39.407, -120.19], 25.VI.1954, R. M. Bohart |
| *Eulonchus sapphirinus* | 012330 | male | UCDC | USA, California, Nevada County, Sagehen near Hobart Mills, [39.407, -120.19], 25.VI.1954, R. M. Bohart |
| *Eulonchus sapphirinus* | 012331 | male | UCDC | USA, California, Nevada County, Sagehen near Hobart Mills, [39.407, -120.19], 25.VI.1954, R. M. Bohart |
| *Eulonchus sapphirinus* | 012332 | male | UCDC | USA, California, Nevada County, Sagehen near Hobart Mills, [39.407, -120.19], 25.VI.1954, R. M. Bohart |
| *Eulonchus sapphirinus* | 012333 | male | UCDC | USA, California, Nevada County, Sagehen near Hobart Mills, [39.407, -120.19], 25.VI.1954, R. M. Bohart |
| *Eulonchus sapphirinus* | 012334 | male | UCDC | USA, California, Nevada County, Sagehen near Hobart Mills, [39.407, -120.19], 25.VI.1954, R. M. Bohart |
| *Eulonchus sapphirinus* | 012335 | male | UCDC | USA, California, Nevada County, Sagehen near Hobart Mills, [39.407, -120.19], 25.VI.1954, R. M. Bohart |
| *Eulonchus sapphirinus* | 012336 | male | UCDC | USA, California, Nevada County, Sagehen near Hobart Mills, [39.407, -120.19], 25.VI.1954, R. M. Bohart |
| *Eulonchus sapphirinus* | 012337 | male | UCDC | USA, California, Nevada County, Sagehen near Hobart Mills, [39.407, -120.19], 25.VI.1954, R. M. Bohart |
| *Eulonchus sapphirinus* | 012338 | male | UCDC | USA, California, Nevada County, Sagehen near Hobart Mills, [39.407, -120.19], 25.VI.1954, R. M. Bohart |
| *Eulonchus sapphirinus* | 012339 | male | UCDC | USA, California, Nevada County, Sagehen near Hobart Mills, [39.407, -120.19], 25.VI.1954, R. M. Bohart |
| *Eulonchus sapphirinus* | 012340 | male | UCDC | USA, California, Nevada County, Sagehen near Hobart Mills, [39.407, -120.19], 25.VI.1954, R. M. Bohart |
| *Eulonchus sapphirinus* | 012341 | male | UCDC | USA, California, Nevada County, Sagehen near Hobart Mills, [39.407, -120.19], 25.VI.1954, R. M. Bohart |
| *Eulonchus sapphirinus* | 012342 | male | UCDC | USA, California, Nevada County, Sagehen near Hobart Mills, [39.407, -120.19], 25.VI.1954, R. M. Bohart |
| *Eulonchus sapphirinus* | 012353 | male | UCDC | USA, California, Nevada County, Sagehen near Hobart Mills, [39.407, -120.19], 25.VI.1954, R. M. Bohart |
| *Eulonchus sapphirinus* | 012354 | male | UCDC | USA, California, Nevada County, Sagehen near Hobart Mills, [39.407, -120.19], 25.VI.1954, R. M. Bohart |
| *Eulonchus sapphirinus* | 012355 | male | UCDC | USA, California, Nevada County, Sagehen near Hobart Mills, [39.407, -120.19], 25.VI.1954, R. M. Bohart |
| *Eulonchus sapphirinus* | 012356 | male | CAS | USA, California, Plumas Co., 13.VI.1913, F. W. Nunenmacher |
| *Eulonchus sapphirinus* | 012357 | male | CAS | USA, California, Plumas Co., 13.VI.1913, F. W. Nunenmacher |
| *Eulonchus sapphirinus* | 012358 | male | CAS | USA, California, Plumas Co., 13.VI.1913, F. W. Nunenmacher |
| *Eulonchus sapphirinus* | 012359 | male | CAS | USA, California, Shasta County, Lake Eiler, [40.727, -121.568], 9.VII.1947, R. L. Usinger |
| *Eulonchus sapphirinus* | 012360 | male | CAS | USA, California, Lassen County, Susan River Camp, [40.494, -121.093], 10.VII.1949, R. G. Howell |
| *Eulonchus sapphirinus* | 012361 | male | CAS | USA, California, Placer Co, Robertson Flat (Robinsons Flat), [39.156, -120.501], 4.VII.1956, R. E. Darby |
| *Eulonchus sapphirinus* | 012362 | male | CAS | USA, California, Placer Co, Robertson Flat (Robinsons Flat), [39.156, -120.501], 4.VII.1956, R. E. Darby |
| *Eulonchus sapphirinus* | 012363 | male | CAS | USA, California, Placer Co, Robertson Flat (Robinsons Flat), [39.156, -120.501], 4.VII.1956, R. E. Darby |
| *Eulonchus sapphirinus* | 012364 | male | CAS | USA, California, Plumas Co., 13.VI.1913, F. W. Nunenmacher |
| *Eulonchus sapphirinus* | 012365 | male | CAS | USA, California, Plumas Co., 13.VI.1913, F. W. Nunenmacher |
| *Eulonchus sapphirinus* | 012366 | male | CAS | USA, California, Caribou, Feather River, [40.073, -121.166], 1.VI.1946, B. J. Adelson |
| *Eulonchus sapphirinus* | 012367 | male | CAS | USA, California, Lassen National Park, Hat Lake, [40.509, -121.466], 14.VI.1941 |
| *Eulonchus sapphirinus* | 020222 | male | USNM | USA, California, Sierra Co., GOLD Lake, Gold Lake, [39.678, -120.657], 20.VII.1921 |
| *Eulonchus sapphirinus* | – | male | TCAC | USA, California, Calaveras State Park, [], 23.V-12.VI.1990, B. Quelvog. |
| *Eulonchus smaragdinus* | 000001 | male | CAS | USA, California, Monrovia Canyon, [34.174, -117.99], 26.V.1957, F. R. Cole |
| *Eulonchus smaragdinus* | 000002 | male | CAS | USA, California, Monrovia Canyon, [34.174, -117.99], 26.V.1957, F. R. Cole |
| *Eulonchus smaragdinus* | 000003 | male | CAS | USA, California, Monrovia Canyon, [34.174, -117.99], 26.V.1957, F. R. Cole |
| *Eulonchus smaragdinus* | 000004 | male | CAS | USA, California, Monrovia Canyon, [34.174, -117.99], 26.V.1957, F. R. Cole |
| *Eulonchus smaragdinus* | 000005 | male | CAS | USA, California, Monrovia Canyon, [34.174, -117.99], 26.V.1957, F. R. Cole |
| *Eulonchus smaragdinus* | 000006 | male | CAS | USA, California, Monterey County, Carmel, [36.555, -121.924], 22.VI.1924, L. S. Slevin |
| *Eulonchus smaragdinus* | 000007 | male | CAS | 28.VI.1892 |
| *Eulonchus smaragdinus* | 000008 | male | CAS | USA, California, San Francisco, [37.774, -122.42], .V., F. X. Williams |
| *Eulonchus smaragdinus* | 000009 | male | CAS | USA, California, Monterey County, Paraiso Springs, [36.331, -121.369], 27.V.1927, J. R. Slevin |
| *Eulonchus smaragdinus* | 000010 | male | CAS | USA, California, Monrovia Canyon, [34.174, -117.99] |
| *Eulonchus smaragdinus* | 000011 | male | CAS | USA, California, San Francisco, [37.774, -122.42], .V.1905, F. X. Williams |
| *Eulonchus smaragdinus* | 000012 | male | CAS | USA, California, Monrovia Canyon, [34.174, -117.99], 26.V.1957, F. R. Cole |
| *Eulonchus smaragdinus* | 000013 | male | CAS | USA, California, Monrovia Canyon, [34.174, -117.99], 26.V.1957, F. R. Cole |
| *Eulonchus smaragdinus* | 000014 | male | CAS | USA, California, Monrovia Canyon, [34.174, -117.99], 26.V.1957, F. R. Cole |
| *Eulonchus smaragdinus* | 000015 | male | CAS | USA, California, between De Haven and Latenville, [39.665, -123.628], 24.VI.1922 |
| *Eulonchus smaragdinus* | 000016 | male | CAS | USA, California, Claremont, [34.095, -117.72], .VII.1922, Baker |
| *Eulonchus smaragdinus* | 000017 | male | CAS | USA, California, Berkeley, [37.871, -122.273], .VI.1922 |
| *Eulonchus smaragdinus* | 000018 | male | CAS | USA, California, The Gavilan, [33.442, -117.213], 31.V.1937, P. H. Timberlake |
| *Eulonchus smaragdinus* | 000019 | male | CAS | USA, California, Monterey County, Paraiso Springs, [36.331, -121.369], 27.V.1927, L. S. Slevin |
| *Eulonchus smaragdinus* | 000020 | male | CAS | USA, California, San Francisco, [37.774, -122.42], .V., F. X. Williams |
| *Eulonchus smaragdinus* | 000021 | male | CAS | USA, California, Los Angeles County, Crystal Lake, [34.319, -117.847], 29.VI.1950, J. W. MacSwain |
| *Eulonchus smaragdinus* | 000022 | male | CAS | USA, California, Alameda County, Oakland, Diamond Canyon, Diamond Boy Scout Camp, [37.821, -122.2], 26.VI.1929, |
| *Eulonchus smaragdinus* | 000023 | male | CAS | USA, California, Los Angeles County, Tanbark Flat, [34.204, -117.761], 30.VI.1950, R. O. Schuster |
| *Eulonchus smaragdinus* | 000024 | male | CAS | USA, California, Inyo County, Whitney Portal, [36.589, -118.227], 3.VII.1953, J. W. MacSwain |
| *Eulonchus smaragdinus* | 000025 | male | CAS | USA, California, Inyo County, Whitney Portal, [36.589, -118.227], 3.VII.1953, J. W. MacSwain |
| *Eulonchus smaragdinus* | 000026 | male | CAS | USA, California, Inyo County, Whitney Portal, [36.589, -118.227], 3.VII.1953, J. W. MacSwain |
| *Eulonchus smaragdinus* | 000027 | male | CAS | USA, California, Inyo County, Whitney Portal, [36.589, -118.227], 3.VII.1953, J. W. MacSwain |
| *Eulonchus smaragdinus* | 000028 | male | CAS | USA, California, Monterey County, Monterey, [36.6, -121.895], 18.IX.1951, W. K. Dayton |
| *Eulonchus smaragdinus* | 000029 | male | CAS | USA, California, Inyo County, Whitney Portal, [36.589, -118.227], 3.VII.1953, J. W. MacSwain |
| *Eulonchus smaragdinus* | 000030 | male | CAS | USA, California, Inyo County, Whitney Portal, [36.589, -118.227], 3.VII.1953, J. W. MacSwain |
| *Eulonchus smaragdinus* | 000031 | male | CAS | USA, California, Inyo County, Whitney Portal, [36.589, -118.227], 3.VII.1953, J. W. MacSwain |
| *Eulonchus smaragdinus* | 000032 | male | CAS | USA, California, Inyo County, Whitney Portal, [36.589, -118.227], 3.VII.1953, J. W. MacSwain |
| *Eulonchus smaragdinus* | 000033 | male | CAS | USA, California, Los Angeles County, Crystal Lake, [34.319, -117.847], 29.VI.1950, J. W. MacSwain |
| *Eulonchus smaragdinus* | 000034 | male | CAS | USA, California, Los Angeles County, Tanbark Flat, [34.204, -117.761], 28.VI.1950, J. W. MacSwain |
| *Eulonchus smaragdinus* | 000035 | male | CAS | USA, California, Los Angeles County, Tanbark Flat, [34.204, -117.761], 22.VI.1950, J. W. MacSwain |
| *Eulonchus smaragdinus* | 000036 | male | CAS | MEXICO, Baja California, 43.5 km south San Vicente, [31.298, -116.241], 25.IV.1950, R. L. Langston |
| *Eulonchus smaragdinus* | 000037 | male | CAS | USA, California, Los Angeles County, Tanbark Flat, [34.204, -117.761], 22.VI.1950, J. W. MacSwain |
| *Eulonchus smaragdinus* | 000038 | male | CAS | USA, California, Los Angeles County, Tanbark Flat, [34.204, -117.761], 22.VI.1950, J. W. MacSwain |
| *Eulonchus smaragdinus* | 000039 | male | CAS | USA, California, Los Angeles County, Tanbark Flat, [34.204, -117.761], 21.VI.1956, C. L. Wiley |
| *Eulonchus smaragdinus* | 000040 | male | CAS | USA, California, San Bernardino County, near Upland, Cucamonga Canyon, [34.161, -117.637], 21.V.1950 |
| *Eulonchus smaragdinus* | 000041 | male | CAS | USA, California, Riverside County, Idyllwild, [33.746, -116.716], 27.VI.1956, M. S. Wasbauer |
| *Eulonchus smaragdinus* | 000042 | male | CAS | USA, California, San Jacinto Mountains, Idyllwild, [33.814, -116.679], 19.VI.1951, R. C. Bechtel |
| *Eulonchus smaragdinus* | 000043 | male | CAS | USA, California, San Jacinto Mountains, Idyllwild, [33.814, -116.679], 19.VI.1951, R. C. Bechtel |
| *Eulonchus smaragdinus* | 000044 | male | CAS | USA, California, San Jacinto Mountains, Idyllwild, [33.814, -116.679], 19.VI.1951, R. C. Bechtel |
| *Eulonchus smaragdinus* | 000045 | male | CAS | USA, California, Monterey County, Pebble Beach, [36.566, -121.946], 27.V.1921, E. C. Van Dyke |
| *Eulonchus smaragdinus* | 000046 | male | CAS | USA, California, San Luis Obispo County, near Morro Bay, [35.352, -120.838], 17.VI.1950, E. I. Schlinger |
| *Eulonchus smaragdinus* | 000047 | male | CAS | USA, California, Los Angeles County, Tanbark Flat, [34.204, -117.761], 19.VI.1956, B. M. Bartosh |
| *Eulonchus smaragdinus* | 000048 | male | CAS | USA, California, San Luis Obispo County, near Morro Bay, [35.352, -120.838], 17.VI.1950, E. I. Schlinger |
| *Eulonchus smaragdinus* | 000049 | male | UCDC | USA, California, Monterey County, Arroyo Seco Campground, [36.232, -121.485], 19.V.1964, R. M. Bohart |
| *Eulonchus smaragdinus* | 000050 | male | CAS | USA, California, Los Angeles County, Tanbark Flat, [34.204, -117.761], 19.VI.1956, B. M. Bartosh |
| *Eulonchus smaragdinus* | 000051 | male | CAS | USA, California, Riverside County, The Gavilan, [33.442, -117.213], 17.V.1951, E. I. Schlinger |
| *Eulonchus smaragdinus* | 000052 | male | CAS | USA, California, Los Angeles County, Tanbark Flat, [34.204, -117.761], 20.VI.1956, R. C. Bechtel |
| *Eulonchus smaragdinus* | 000053 | male | CAS | USA, California, Lake County |
| *Eulonchus smaragdinus* | 000054 | male | CAS | USA, California, Placer County, Emigrant Gap, [39.297, -120.673], 13.VI.1939 |
| *Eulonchus smaragdinus* | 000055 | male | CAS | USA, California, Inyo County, Whitney Portal, [36.589, -118.227], 3.VII.1953, E. Nakakihara |
| *Eulonchus smaragdinus* | 000056 | male | CAS | USA, California, San Bernardino County, Seven Oaks, [34.186, -116.914], 15.X.1934, A. C. Browne |
| *Eulonchus smaragdinus* | 000057 | male | CAS | USA, California, San Bernardino County, Seven Oaks, [34.186, -116.914], 15.X.1934, A. C. Browne |
| *Eulonchus smaragdinus* | 000058 | male | CAS | USA, California, Los Angeles County, San Gabriel Mountains, Fish Canyon, [34.158, -117.924], 27.IV.1949, E. I. Schlinger |
| *Eulonchus smaragdinus* | 000059 | male | CAS | USA, California, Los Angeles County, Tanbark Flat, [34.204, -117.761], 21.VI.1950, J. C. Hall |
| *Eulonchus smaragdinus* | 000060 | male | CAS | USA, California, Los Angeles County, Tanbark Flat, [34.204, -117.761], 20.VI.1956, L. W. Shainberg |
| *Eulonchus smaragdinus* | 000061 | male | CAS | USA, California, Los Angeles County, Tanbark Flat, [34.204, -117.761], 21.VI.1956, H. R. Moffitt |
| *Eulonchus smaragdinus* | 000062 | male | CAS | USA, California, Los Angeles County, Tanbark Flat, [34.204, -117.761], 20.VI.1956, L. W. Shainberg |
| *Eulonchus smaragdinus* | 000063 | male | CAS | USA, California, San Luis Obispo County, 16.1 km east Morro Bay, [35.372, -120.818], 30.VI.1953, J. C. Downey |
| *Eulonchus smaragdinus* | 000064 | male | CAS | USA, California, San Luis Obispo County, 16.1 km east Morro Bay, [35.372, -120.818], 30.VI.1953, J. C. Downey |
| *Eulonchus smaragdinus* | 000065 | male | CAS | USA, California, Santa Cruz County, Highland District, [37.081, -122.081], 5.VII.1958, S. M. Fidel |
| *Eulonchus smaragdinus* | 000066 | male | CAS | USA, California, Inyo County, Whitney Portal, [36.589, -118.227], 3.VII.1953, J. W. MacSwain |
| *Eulonchus smaragdinus* | 000067 | male | CAS | USA, California, Los Angeles County, San Gabriel Mountains, Fish Canyon, [34.158, -117.924], 27.IV.1949, E. I. Schlinger |
| *Eulonchus smaragdinus* | 000068 | male | CAS | USA, California, Inyo County, Whitney Portal, [36.589, -118.227], 3.VII.1953, J. W. MacSwain |
| *Eulonchus smaragdinus* | 000069 | male | CAS | USA, California, Lakeport, [39.043, -122.916], 27.VI.1957, S. M. Fidel |
| *Eulonchus smaragdinus* | 000070 | male | CAS | USA, California, Monrovia Canyon, [34.174, -117.99], 20.VII.1930, C. H. Martin, D. Martin |
| *Eulonchus smaragdinus* | 000071 | male | CAS | MEXICO, Baja California, 43.5 km south San Vicente, [31.298, -116.241], 25.IV.1950, R. L. Langston |
| *Eulonchus smaragdinus* | 000072 | male | CAS | USA, California, Lakeport, [39.043, -122.916], 27.VI.1957, S. M. Fidel |
| *Eulonchus smaragdinus* | 000073 | male | CAS | USA, California, Mount Diablo, [37.882, -121.914], 610 m, 26.VI.1951, F. X. Williams |
| *Eulonchus smaragdinus* | 000074 | male | CAS | USA, California, Lakeport, [39.043, -122.916], 27.VI.1957, S. M. Fidel |
| *Eulonchus smaragdinus* | 000075 | male | CAS | USA, California, Los Angeles County, Beverly Glen, [34.108, -118.442], 30.VI.1952, R. X. Schick |
| *Eulonchus smaragdinus* | 000076 | male | CAS | USA, California, Los Angeles County, Beverly Glen, [34.108, -118.442], 4.VII.1952, W. A. MacDonald |
| *Eulonchus smaragdinus* | 000077 | male | CAS | USA, California, Napa County, Samuel Springs, [38.604, -122.311], 30.V.1953, R. C. Bechtel |
| *Eulonchus smaragdinus* | 000078 | male | CAS | USA, California, Napa County, Samuel Springs, [38.604, -122.311], 7.VI.1953, R. C. Bechtel |
| *Eulonchus smaragdinus* | 000079 | male | UCDC | USA, California, Santa Barbara County, Santa Ynez Mountains, [34.549, -120.029], 24.VI.1959, R. M. Bohart |
| *Eulonchus smaragdinus* | 000080 | male | CAS | USA, California, Santa Barbara County, Santa Ynez Mountains, [34.549, -120.029], 24.VI.1929, M. Bruck |
| *Eulonchus smaragdinus* | 000081 | male | CAS | USA, California, Santa Barbara County, Santa Ynez Mountains, [34.549, -120.029], 24.VI.1959, R. W. Spore |
| *Eulonchus smaragdinus* | 000082 | male | CAS | USA, California, Santa Barbara County, Santa Ynez Mountains, [34.549, -120.029], 24.VI.1959, A. E. Menke |
| *Eulonchus smaragdinus* | 000083 | male | CAS | USA, California, Lake County, Hopland Grade, [38.997, -122.949], 10.VI.1959, S. M. Fidel |
| *Eulonchus smaragdinus* | 000085 | male | CAS | USA, California, Riverside County, The Gavilan, [33.442, -117.213], 17.V.1951, R. C. Bechtel |
| *Eulonchus smaragdinus* | 000086 | male | CAS | USA, California, Riverside County, The Gavilan, [33.442, -117.213], 18.V.1951, R. C. Bechtel |
| *Eulonchus smaragdinus* | 000087 | male | CAS | USA, California, San Jacinto Mountains, Idyllwild, [33.814, -116.679], 19.VI.1951, R. C. Bechtel |
| *Eulonchus smaragdinus* | 000088 | male | CAS | USA, California, Los Angeles County, San Gabriel Mountains, Fish Canyon, [34.158, -117.924], 27.IV.1949, E. I. Schlinger |
| *Eulonchus smaragdinus* | 000089 | male | EMEC | USA, California, Sonoma County, [38.292, -122.458] |
| *Eulonchus smaragdinus* | 000090 | male | UCDC | USA, California, Los Angeles County, Tanbark Flat, [34.204, -117.761], 8.VII.1952, R. M. Bohart |
| *Eulonchus smaragdinus* | 000091 | male | CAS | USA, California, Riverside County, The Gavilan, [33.442, -117.213], 17.V.1951, R. C. Bechtel |
| *Eulonchus smaragdinus* | 000092 | female | CAS | USA, California, Riverside County, The Gavilan, [33.442, -117.213], 17.V.1951, R. C. Bechtel |
| *Eulonchus smaragdinus* | 000093 | male | CAS | USA, California, Yolo County, Putah Canyon, [38.513, -122.101], 2.VI.1962, M. E. Irwin |
| *Eulonchus smaragdinus* | 000094 | female | CAS | USA, California, Yolo County, Putah Canyon, [38.513, -122.101], 2.VI.1962, M. E. Irwin |
| *Eulonchus smaragdinus* | 000095 | male | CAS | USA, California, Oldenberg |
| *Eulonchus smaragdinus* | 000096 | male | CAS | USA, California, Los Angeles County, Tanbark Flat, [34.204, -117.761], 22.VI.1950, J. W. MacSwain |
| *Eulonchus smaragdinus* | 000097 | male | CAS | USA, California, San Jacinto Mountains, Idyllwild, [33.814, -116.679], 17.VI.1940, C. D. Michener |
| *Eulonchus smaragdinus* | 000098 | male | CAS | USA, California, Alameda County, Oakland, Diamond Canyon, Diamond Boy Scout Camp, [37.821, -122.2], 26.VI.1929 |
| *Eulonchus smaragdinus* | 000099 | male | CAS | USA, California, Los Angeles County, Tanbark Flat, [34.204, -117.761], 22.VI.1956, C. L. Wiley |
| *Eulonchus smaragdinus* | 000100 | male | CAS | USA, California, San Jacinto Mountains, Idyllwild, [33.814, -116.679], 17.VI.1940 |
| *Eulonchus smaragdinus* | 000101 | male | CAS | USA, California, San Jacinto Mountains, Herkey Creek, [33.675, -116.682], 20.VI.1940 |
| *Eulonchus smaragdinus* | 000102 | male | CAS | USA, California, Santa Clara County, Mount Madonna, [37.012, -121.705], 4.VII.1941, K. Frick |
| *Eulonchus smaragdinus* | 000103 | male | CAS | USA, California, Los Angeles County, Crystal Lake, [34.319, -117.847], 29.VI.1950, J. W. MacSwain |
| *Eulonchus smaragdinus* | 000104 | male | CAS | USA, California, Los Angeles County, Crystal Lake, [34.319, -117.847], 29.VI.1950, J. W. MacSwain |
| *Eulonchus smaragdinus* | 000105 | male | CAS | USA, California, Los Angeles County, Crystal Lake, [34.319, -117.847], 29.VI.1950, J. W. MacSwain |
| *Eulonchus smaragdinus* | 000106 | male | CAS | USA, California, Los Angeles County, Tanbark Flat, [34.204, -117.761], 20.VI.1956, R. C. Bechtel |
| *Eulonchus smaragdinus* | 000107 | male | CAS | USA, California, Los Angeles County, Tanbark Flat, [34.204, -117.761], 21.VI.1956, R. C. Bechtel |
| *Eulonchus smaragdinus* | 000108 | male | CAS | USA, California, Los Angeles County, Crystal Lake, [34.319, -117.847], 29.VI.1950, J. W. MacSwain |
| *Eulonchus smaragdinus* | 000109 | male | CAS | USA, California, Los Angeles County, Tanbark Flat, [34.204, -117.761], 19.VI.1956, B. M. Bartosh |
| *Eulonchus smaragdinus* | 000110 | male | CAS | USA, California, Los Angeles County, Tanbark Flat, [34.204, -117.761], 23.VI.1956, R. C. Bechtel |
| *Eulonchus smaragdinus* | 000111 | male | CAS | USA, California, Los Angeles County, Tanbark Flat, [34.204, -117.761], 20.VI.1956, L. W. Shainberg |
| *Eulonchus smaragdinus* | 000112 | male | CAS | USA, California, Los Angeles County, Tanbark Flat, [34.204, -117.761], 20.VI.1956, L. W. Shainberg |
| *Eulonchus smaragdinus* | 000113 | male | CAS | USA, California, Marin County, Point Reyes National Seashore, Point Reyes, 0.2 km E. McCLure Beach, [38.188, -122.956], 12.VII.1969, P. A. Rauch |
| *Eulonchus smaragdinus* | 000114 | male | CAS | USA, California, San Diego Co., Mount Palomar, Crestline Picnic Area, [33.323, -116.879], 1615 m, 22.VII.1965, R. E. Somerby, B. Ruge |
| *Eulonchus smaragdinus* | 000115 | male | CAS | USA, California, Marin County, Point Reyes National Seashore, Point Reyes, 0.2 km E. McCLure Beach, [38.188, -122.956], 12.VII.1969, P. A. Rauch |
| *Eulonchus smaragdinus* | 000116 | male | CAS | USA, California, San Jacinto Mountains, Idyllwild, [33.814, -116.679], 19.VI.1951, R. C. Bechtel |
| *Eulonchus smaragdinus* | 000117 | male | CAS | USA, California, Tuolumne County, Strawberry, [38.198, -120.009], 20.VI.1957, J. W. MacSwain |
| *Eulonchus smaragdinus* | 000118 | male | CAS | USA, California, San Diego Co., Mount Palomar, Crestline Picnic Area, [33.323, -116.879], 1615 m, 22.VII.1965, R. E. Somerby, B. Ruge |
| *Eulonchus smaragdinus* | 000119 | male | CAS | USA, California, San Diego Co., Mount Palomar, Crestline Picnic Area, [33.323, -116.879], 1615 m, 22.VII.1965, R. E. Somerby, B. Ruge |
| *Eulonchus smaragdinus* | 000120 | male | CAS | USA, California, San Diego Co., Mount Palomar, Crestline Picnic Area, [33.323, -116.879], 1615 m, 22.VII.1965, R. E. Somerby, B. Ruge |
| *Eulonchus smaragdinus* | 000121 | male | CAS | USA, California, Yolo County, Putah Canyon, [38.513, -122.101], 2.VI.1962, M. E. Irwin |
| *Eulonchus smaragdinus* | 000122 | male | CAS | USA, California, Santa Barbara County, Santa Ynez Mountains, [34.549, -120.029], 24.VI.1959, P. E. Paige |
| *Eulonchus smaragdinus* | 000123 | male | CAS | USA, California, Santa Barbara County, Santa Ynez Mountains, [34.549, -120.029], 24.VI.1959, J. L. Bath |
| *Eulonchus smaragdinus* | 000124 | male | CAS | USA, California, Santa Barbara County, Santa Ynez Mountains, [34.549, -120.029], 24.VI.1959, P. E. Paige |
| *Eulonchus smaragdinus* | 000125 | male | CAS | USA, California, Santa Barbara County, Santa Ynez Mountains, [34.549, -120.029], 24.VI.1959, P. E. Paige |
| *Eulonchus smaragdinus* | 000126 | male | CAS | USA, California, Santa Barbara County, San Rafael Mountains, Bluff Camp, [34.685, -119.666], 29.VI.1959, F. D. Parker |
| *Eulonchus smaragdinus* | 000127 | male | CAS | USA, California, San Diego Co., Mount Laguna, [32.867, -116.419], 21.VI.1963, T. Bolton |
| *Eulonchus smaragdinus* | 000128 | male | CAS | USA, California, San Diego Co., Mount Palomar, Crestline Picnic Area, [33.323, -116.879], 1615 m, 22.VII.1965, R. E. Somerby, B. Ruge |
| *Eulonchus smaragdinus* | 000129 | male | CAS | USA, California, Los Angeles County, Tanbark Flat, [34.204, -117.761], 18.VI.1956, R. C. Bechtel |
| *Eulonchus smaragdinus* | 000130 | male | CAS | USA, California, Los Angeles County, Tanbark Flat, [34.204, -117.761], 18.VI.1956, R. C. Bechtel |
| *Eulonchus smaragdinus* | 000131 | male | CAS | USA, California, Los Angeles County, Tanbark Flat, [34.204, -117.761], 23.VI.1956, R. C. Bechtel |
| *Eulonchus smaragdinus* | 000132 | male | CAS | USA, California, Los Angeles County, Tanbark Flat, [34.204, -117.761], 20.VI.1956, R. C. Bechtel |
| *Eulonchus smaragdinus* | 000133 | male | CAS | USA, California, Los Angeles County, Tanbark Flat, [34.204, -117.761], 22.VI.1956, R. W. Bushing |
| *Eulonchus smaragdinus* | 000134 | male | CAS | USA, California, Los Angeles County, Tanbark Flat, [34.204, -117.761], 20.VI.1956, R. C. Bechtel |
| *Eulonchus smaragdinus* | 000135 | male | CAS | USA, California, Los Angeles County, Tanbark Flat, [34.204, -117.761], 20.VI.1956, R. C. Bechtel |
| *Eulonchus smaragdinus* | 000136 | male | CAS | USA, California, Marin County, Mill Valley, [37.906, -122.545], 110 m, 13.VII.1965 to 15.VII.1965, P. H. Arnaud, Jr. |
| *Eulonchus smaragdinus* | 000137 | male | CAS | USA, California, Los Angeles County, Camp Baldy, [34.239, -117.669], 11.VII.1950, D. Newby |
| *Eulonchus smaragdinus* | 000138 | male | CAS | USA, California, Monterey County, Partington Ridge Road, 14.V.1964, R. M. Brown |
| *Eulonchus smaragdinus* | 000139 | male | CAS | USA, California, Santa Cruz County, 8.1 km east Glenwood, [37.109, -121.896], 2.VII.1956, D. J. Burdick |
| *Eulonchus smaragdinus* | 000140 | male | CAS | USA, California, San Luis Obispo County, 12.9 km west Atascadero, [35.491, -120.813], 3.VII.1956, P. D. Hurd |
| *Eulonchus smaragdinus* | 000141 | male | CAS | USA, California, Santa Cruz County, 8.1 km east Glenwood, [37.109, -121.896], 2.VII.1956, D. J. Burdick |
| *Eulonchus smaragdinus* | 000142 | male | CAS | USA, California, San Luis Obispo County, 12.9 km west Atascadero, [35.491, -120.813], 3.VII.1956, P. D. Hurd |
| *Eulonchus smaragdinus* | 000143 | male | CAS | USA, California, Santa Cruz County, 8.1 km east Glenwood, [37.109, -121.896], 2.VII.1956, D. J. Burdick |
| *Eulonchus smaragdinus* | 000144 | male | CAS | USA, California, San Gabriel Mountains, Switzer’s Trail, [34.259, -118.155], 1067 m, 11.VI.1910, F. Grinnell |
| *Eulonchus smaragdinus* | 000145 | male | CAS | USA, California, San Diego County, The Willows, [32.835, -116.723], 14.VI.1957, C. L. Hogue |
| *Eulonchus smaragdinus* | 000146 | male | CAS | USA, California, San Bernardino County, Miller Canyon, [34.284, -117.331], 1.VII.1956, A. Menke |
| *Eulonchus smaragdinus* | 000147 | male | CAS | USA, California, Los Angeles County, Tanbark Flat, [34.204, -117.761], 22.VI.1956, A. Menke |
| *Eulonchus smaragdinus* | 000148 | male | CAS | USA, California, Claremont, [34.095, -117.72], Baker |
| *Eulonchus smaragdinus* | 000149 | male | CAS | USA, California, Santa Clara County, San Antonio Valley, [37.355, -121.92], 13.VI.1950, H. E. Cott |
| *Eulonchus smaragdinus* | 000150 | male | CAS | USA, California, Santa Clara County, San Antonio Valley, [37.355, -121.92], 13.VI.1950, H. E. Cott |
| *Eulonchus smaragdinus* | 000151 | male | CAS | USA, California, Mendocino County, 3.2 km north Howard Lake, [39.909, -122.993], 1128 m, 11.VI.1972, S. L. Szerlip |
| *Eulonchus smaragdinus* | 000152 | male | CAS | USA, California, Mendocino County, 3.2 km north Howard Lake, [39.909, -122.993], 1128 m, 11.VI.1972, S. L. Szerlip |
| *Eulonchus smaragdinus* | 000153 | male | CAS | USA, California, Mendocino County, 3.2 km north Howard Lake, [39.909, -122.993], 1128 m, 11.VI.1972, S. L. Szerlip |
| *Eulonchus smaragdinus* | 000154 | male | CAS | USA, California, Mendocino County, 3.2 km north Howard Lake, [39.909, -122.993], 1128 m, 11.VI.1972, S. L. Szerlip |
| *Eulonchus smaragdinus* | 000155 | male | CAS | USA, California, Mendocino County, 3.2 km north Howard Lake, [39.909, -122.993], 1128 m, 11.VI.1972, S. L. Szerlip |
| *Eulonchus smaragdinus* | 000156 | female | CAS | USA, California, Santa Cruz County, Mount Hermon, [37.051, -122.059], 6.VI.1947, W. D. Hazeltine |
| *Eulonchus smaragdinus* | 000157 | female | CAS | USA, California, San Jacinto Mountains, Idyllwild, [33.814, -116.679], 14.VI.1940 |
| *Eulonchus smaragdinus* | 000158 | female | CAS | USA, California, Monrovia Canyon, [34.174, -117.99], 4.VII.1930 |
| *Eulonchus smaragdinus* | 000159 | female | CAS | USA, California, San Bernardino County |
| *Eulonchus smaragdinus* | 000161 | female | CAS | USA, California, Los Angeles County, Tanbark Flat, [34.204, -117.761], 22.VI.1956, C. L. Wiley |
| *Eulonchus smaragdinus* | 000162 | female | CAS | USA, California, The Gavilan, [33.442, -117.213], 31.V.1937, P. H. Timberlake |
| *Eulonchus smaragdinus* | 000164 | female | CAS | USA, California, Monterey Co., Bixby Creek, [36.372, -121.9], 1.VIII.1949, M. S. Wasbauer |
| *Eulonchus smaragdinus* | 000165 | female | CAS | USA, California, Monrovia Canyon, [34.174, -117.99], 4.VII.1930 |
| *Eulonchus smaragdinus* | 000166 | female | CAS | USA, California, Los Angeles County, Tanbark Flat, [34.204, -117.761], 23.VI.1956, C. L. Wiley |
| *Eulonchus smaragdinus* | 000167 | female | CAS | USA, California, Los Angeles County, Crystal Lake, [34.319, -117.847], 29.VI.1950, J. W. MacSwain |
| *Eulonchus smaragdinus* | 000168 | female | CAS | USA, California, Los Angeles County, Tanbark Flat, [34.204, -117.761], 23.VI.1956, C. L. Wiley |
| *Eulonchus smaragdinus* | 000169 | female | CAS | USA, California, Riverside County, The Gavilan, [33.442, -117.213], 18.V.1951, E. I. Schlinger |
| *Eulonchus smaragdinus* | 000170 | female | CAS | USA, California, Mono County, Sardine Creek, Levitt Creek, [38.307, -119.589], .VII.1951, B. Baker |
| *Eulonchus smaragdinus* | 000171 | female | CAS | USA, California, Napa County, Samuel Springs, [38.604, -122.311], 7.VI.1953, R. C. Bechtel |
| *Eulonchus smaragdinus* | 000172 | female | CAS | USA, California, San Diego Co., Mount Palomar, Crestline Picnic Area, [33.323, -116.879], 1615 m, 22.VII.1965, R. E. Somerby, B. Ruge |
| *Eulonchus smaragdinus* | 000173 | female | CAS | USA, California, Yolo County, Putah Canyon, [38.513, -122.101], 3.VI.1962, M. E. Irwin |
| *Eulonchus smaragdinus* | 000174 | female | CAS | USA, California, Orange County, Santiago Canyon, [33.76, -117.703], 10.VI.1962, M. E. Irwin |
| *Eulonchus smaragdinus* | 000175 | female | CAS | USA, California, Los Angeles County, Tanbark Flat, [34.204, -117.761], 19.VI.1956, B. M. Bartosh |
| *Eulonchus smaragdinus* | 000176 | female | CAS | USA, California, Pacific Grove, [36.618, -121.917], 2.VI.1904 |
| *Eulonchus smaragdinus* | 000177 | female | CAS | USA, California, Inyo County, Whitney Portal, [36.589, -118.227], 3.VII.1953, J. W. MacSwain |
| *Eulonchus smaragdinus* | 000178 | female | CAS | USA, California, Los Angeles County, Tanbark Flat, [34.204, -117.761], 22.VI.1956, R. W. Bushing |
| *Eulonchus smaragdinus* | 000179 | female | CAS | USA, California, Napa County, Samuel Springs, [38.604, -122.311], 30.V.1953, R. C. Bechtel |
| *Eulonchus smaragdinus* | 000180 | female | CAS | USA, California, Los Angeles County, Tanbark Flat, [34.204, -117.761], 21.VI.1956, R. C. Bechtel |
| *Eulonchus smaragdinus* | 000181 | female | CAS | USA, California, Monterey County, Arroyo Seco Campground, [36.232, -121.485], 5.VI.1956, R. C. Bechtel |
| *Eulonchus smaragdinus* | 000182 | female | CAS | USA, California, Santa Cruz County, Highland District, [37.081, -122.081], 4.VII.1956, S. M. Fidel |
| *Eulonchus smaragdinus* | 000183 | female | CAS | USA, California, San Bernardino County, Camp Baldy, 29.VI.1956, H. R. Moffitt |
| *Eulonchus smaragdinus* | 000184 | female | CAS | USA, California, Santa Cruz County, Highland District, [37.081, -122.081], 4.VII.1956, S. M. Fidel |
| *Eulonchus smaragdinus* | 000185 | female | CAS | USA, California, Inyo County, Whitney Portal, [36.589, -118.227], 3.VII.1953, J. W. MacSwain |
| *Eulonchus smaragdinus* | 000186 | female | CAS | USA, California, San Jacinto Mountains, Idyllwild, [33.814, -116.679], 19.VI.1951, G. C. Bechtel |
| *Eulonchus smaragdinus* | 000187 | female | CAS | USA, California, Santa Barbara County, Santa Ynez Mountains, [34.549, -120.029], 24.VI.1959, A. E. Menke |
| *Eulonchus smaragdinus* | 000188 | female | CAS | USA, California, Napa County, Samuel Springs, [38.604, -122.311], 7.VI.1953, R. C. Bechtel |
| *Eulonchus smaragdinus* | 000189 | female | CAS | USA, California, San Francisco, [37.774, -122.42], 21.V.1922, C. L. Fox |
| *Eulonchus smaragdinus* | 000190 | female | CAS | USA, California, San Jacinto Mountains, Idyllwild, [33.814, -116.679], 19.VI.1951, R. C. Bechtel |
| *Eulonchus smaragdinus* | 000191 | female | CAS | USA, California, Solano County, Green Valley, [38.252, -122.163], 19.VI.1953, E. I. Schlinger |
| *Eulonchus smaragdinus* | 000192 | female | CAS | USA, California, San Diego County, Cleveland National Forest, Indian Flats Campground, [33.349, -116.659], 1158 m, 26.V.1968, J. A. Honey |
| *Eulonchus smaragdinus* | 000193 | female | CAS | MEXICO, Baja California, San Quintin, [30.56, -115.943], 30.III.1972, C. H. Bruemmer |
| *Eulonchus smaragdinus* | 000194 | female | CAS | USA, California, Butte County, Feather Falls, [39.642, -121.275], 549 m, 29.VI.1963, E. E. Ball |
| *Eulonchus smaragdinus* | 000195 | female | CAS | USA, California, Marin County, Mill Valley, [37.906, -122.545], 110 m, 13.VII.1965 to 15.VII.1965, P. H. Arnaud, Jr. |
| *Eulonchus smaragdinus* | 000219 | male | CAS | USA, California, Riverside County, San Gorgonio Pass, [33.917, -116.751], 28.V.1941, E. C. Van Dyke |
| *Eulonchus smaragdinus* | 000220 | male | CAS | USA, California, San Diego County, Kearny Mesa, [32.829, -117.144], 160 m, 30.IV.1955, S. E. Olson |
| *Eulonchus smaragdinus* | 000273 | male | CAS | USA, California, Los Angeles County, Tanbark Flat, [34.204, -117.761], 19.VI.1956, B. M. Bartosh |
| *Eulonchus smaragdinus* | 000457 | male | CAS | USA, California, Monrovia Canyon, [34.174, -117.99], .VII., F. R. Cole |
| *Eulonchus smaragdinus* | 000458 | male | CAS | USA, California, Los Angeles County, Tanbark Flat, [34.204, -117.761], 20.VI.1956, J. I. Stage |
| *Eulonchus smaragdinus* | 000459 | male | CAS | USA, California, Marin County, McCLure’s Beach, [38.189, -122.958], 8.VII.1961, C. A. or K. Toschi |
| *Eulonchus smaragdinus* | 000460 | male | CAS | USA, California, Santa Barbara County, Santa Ynez Mountains, [34.549, -120.029], 24.VI.1953, R. D. Gehring |
| *Eulonchus smaragdinus* | 000461 | male | CAS | USA, California, Contra Costa County, Mount Diablo, [37.882, -121.914], 18.VI.1963, D. H. Janzen |
| *Eulonchus smaragdinus* | 000462 | male | CAS | USA, California, Marin County, McCLure’s Beach, [38.189, -122.958], 8.VI.1957, A. E. Michelbacher |
| *Eulonchus smaragdinus* | 000463 | male | CAS | USA, California, Los Angeles County, Tanbark Flat, [34.204, -117.761], 20.VI.1956, J. I. Stage |
| *Eulonchus smaragdinus* | 000464 | male | CAS | USA, California, Los Angeles County, Tanbark Flat, [34.204, -117.761], 20.VI.1956, J. I. Stage |
| *Eulonchus smaragdinus* | 000465 | male | CAS | USA, California, San Diego Co., Mount Laguna, [32.867, -116.419], 5.VII.1963, J. A. Powell |
| *Eulonchus smaragdinus* | 000466 | male | CAS | USA, California, Santa Barbara County, Santa Ynez Mountains, [34.549, -120.029], 24.VI.1959, C. A. Campbell |
| *Eulonchus smaragdinus* | 000467 | male | CAS | USA, California, San Luis Obispo County, 9.7 km northeast Santa Margarita, [35.459, -120.543], 22.VI.1958, E. G. Linsley |
| *Eulonchus smaragdinus* | 000468 | male | CAS | USA, California, San Luis Obispo County, 9.7 km northeast Santa Margarita, [35.459, -120.543], 22.VI.1958, E. G. Linsley |
| *Eulonchus smaragdinus* | 000469 | male | CAS | USA, California, Inyo County, Whitney Portal, [36.589, -118.227], 3.VII.1953, D. D. Linsdale |
| *Eulonchus smaragdinus* | 000470 | male | CAS | USA, California, Santa Barbara County, Santa Ynez Mountains, [34.549, -120.029], 24.VI.1953, R. D. Gehring |
| *Eulonchus smaragdinus* | 000471 | male | CAS | USA, California, Santa Barbara County, Santa Ynez Mountains, [34.549, -120.029], 24.VI.1959, C. A. Campbell |
| *Eulonchus smaragdinus* | 000472 | male | CAS | USA, California, Santa Barbara County, Santa Ynez Mountains, [34.549, -120.029], 24.VI.1953, R. D. Gehring |
| *Eulonchus smaragdinus* | 000473 | male | CAS | USA, California, Santa Barbara County, Canon del Refugio, 8.1 km E Hwy 101, [34.502, -120.066], 25.VI.1959, R. D. Gehring |
| *Eulonchus smaragdinus* | 000474 | male | CAS | USA, California, San Diego, [32.719, -117.156], 2.VII.1953, J. A. Powell |
| *Eulonchus smaragdinus* | 000475 | male | CAS | USA, California, San Bernardino County, Camp Baldy Road, [34.252, -117.641], 1981 m, 7.VII.1956, L. W. Shainberg |
| *Eulonchus smaragdinus* | 000476 | male | CAS | USA, California, Santa Barbara County, Santa Ynez Mountains, [34.549, -120.029], 24.VI.1959, C. A. Campbell |
| *Eulonchus smaragdinus* | 000477 | male | CAS | USA, California, Monterey County, Arroyo Seco Campground, [36.232, -121.485], 27.V.1956, R. C. Bechtel |
| *Eulonchus smaragdinus* | 000478 | male | CAS | USA, California, Marin County, McCLure’s Beach, [38.189, -122.958], 24.V.1962, A. E. Michelbacher |
| *Eulonchus smaragdinus* | 000479 | male | CAS | USA, California, Santa Barbara County, Santa Ynez Mountains, [34.549, -120.029], 24.VI.1959, W. A. Steffan |
| *Eulonchus smaragdinus* | 000480 | male | CAS | USA, California, Marin County, McCLure’s Beach, [38.189, -122.958], 31.V.1962, A. E. Michelbacher |
| *Eulonchus smaragdinus* | 000481 | male | CAS | USA, California, Marin County, McCLure’s Beach, [38.189, -122.958], 24.V.1962, A. E. Michelbacher |
| *Eulonchus smaragdinus* | 000482 | male | CAS | USA, California, Marin County, McCLure’s Beach, [38.189, -122.958], 24.V.1962, A. E. Michelbacher |
| *Eulonchus smaragdinus* | 000483 | male | CAS | USA, California, Santa Barbara County, Santa Ynez Mountains, [34.549, -120.029], 4.VII.1959, R. D. Gehring |
| *Eulonchus smaragdinus* | 000484 | male | CAS | USA, California, Inyo County, Whitney Portal, [36.589, -118.227], 3.VII.1953, D. D. Linsdale |
| *Eulonchus smaragdinus* | 000485 | male | CAS | USA, California, Santa Barbara County, Canon del Refugio, 8.1 km E Hwy 101, [34.502, -120.066], 25.VI.1959, R. D. Gehring |
| *Eulonchus smaragdinus* | 000486 | male | CAS | USA, California, Santa Barbara County, Santa Ynez Mountains, [34.549, -120.029], 24.VI.1959, W. A. Steffan |
| *Eulonchus smaragdinus* | 000487 | male | CAS | USA, California, Marin County, McCLure’s Beach, [38.189, -122.958], 31.V.1962, A. E. Michelbacher |
| *Eulonchus smaragdinus* | 000488 | male | CAS | USA, California, Marin County, McCLure’s Beach, [38.189, -122.958], 31.V.1962, A. E. Michelbacher |
| *Eulonchus smaragdinus* | 000489 | male | CAS | USA, California, Marin County, McCLure’s Beach, [38.189, -122.958], 31.V.1962, A. E. Michelbacher |
| *Eulonchus smaragdinus* | 000490 | male | CAS | USA, California, Marin County, McCLure’s Beach, [38.189, -122.958], 31.V.1962, A. E. Michelbacher |
| *Eulonchus smaragdinus* | 000491 | male | CAS | USA, California, Santa Barbara County, Santa Ynez Mountains, [34.549, -120.029], 24.VI.1959, R. D. Gehring |
| *Eulonchus smaragdinus* | 000492 | male | CAS | USA, California, Santa Barbara County, Santa Ynez Mountains, [34.549, -120.029], 24.VI.1959, W. A. Steffan |
| *Eulonchus smaragdinus* | 000493 | male | CAS | USA, California, Santa Barbara County, Santa Ynez Mountains, [34.549, -120.029], 24.VI.1959, R. D. Gehring |
| *Eulonchus smaragdinus* | 000494 | male | CAS | USA, California, Santa Barbara County, San Rafael Mountains, Bluff Camp, [34.685, -119.666], 29.VI.1959, R. D. Gehring |
| *Eulonchus smaragdinus* | 000495 | male | CAS | USA, California, San Luis Obispo County, 9.7 km northeast Santa Margarita, [35.459, -120.543], 12.VI.1963, J. W. MacSwain |
| *Eulonchus smaragdinus* | 000496 | male | CAS | USA, California, San Luis Obispo County, 9.7 km northeast Santa Margarita, [35.459, -120.543], 12.VI.1963, R. W. Thorp |
| *Eulonchus smaragdinus* | 000497 | male | CAS | USA, California, San Luis Obispo County, 9.7 km northeast Santa Margarita, [35.459, -120.543], 12.VI.1963, R. W. Thorp |
| *Eulonchus smaragdinus* | 000499 | male | CAS | USA, California, San Luis Obispo County, 9.7 km northeast Santa Margarita, [35.459, -120.543], 12.VI.1963, J. W. MacSwain |
| *Eulonchus smaragdinus* | 000500 | male | CAS | USA, California, Santa Barbara County, Santa Ynez Mountains, [34.549, -120.029], 24.VI.1959, W. A. Steffan |
| *Eulonchus smaragdinus* | 000501 | male | CAS | USA, California, San Luis Obispo County, 9.7 km northeast Santa Margarita, [35.459, -120.543], 12.VI.1963, J. W. MacSwain |
| *Eulonchus smaragdinus* | 000502 | male | CAS | USA, California, San Luis Obispo County, 9.7 km northeast Santa Margarita, [35.459, -120.543], 12.VI.1963, J. W. MacSwain |
| *Eulonchus smaragdinus* | 000503 | male | CAS | USA, California, San Luis Obispo County, 9.7 km northeast Santa Margarita, [35.459, -120.543], 12.VI.1963, J. W. MacSwain |
| *Eulonchus smaragdinus* | 000504 | male | CAS | USA, California, San Luis Obispo County, 9.7 km northeast Santa Margarita, [35.459, -120.543], 12.VI.1963, J. W. MacSwain |
| *Eulonchus smaragdinus* | 000505 | male | CAS | USA, California, San Luis Obispo County, 9.7 km northeast Santa Margarita, [35.459, -120.543], 12.VI.1963, J. W. MacSwain |
| *Eulonchus smaragdinus* | 000506 | male | CAS | USA, California, San Luis Obispo County, 9.7 km northeast Santa Margarita, [35.459, -120.543], 12.VI.1963, R. W. Thorp |
| *Eulonchus smaragdinus* | 000507 | male | CAS | USA, California, Inyo County, Whitney Portal, [36.589, -118.227], 3.VII.1953, D. D. Linsdale |
| *Eulonchus smaragdinus* | 000508 | male | CAS | USA, California, Santa Barbara County, Santa Ynez Mountains, [34.549, -120.029], 24.VI.1959, R. D. Gehring |
| *Eulonchus smaragdinus* | 000509 | male | CAS | USA, California, Santa Barbara County, Santa Ynez Mountains, [34.549, -120.029], 24.VI.1959, W. A. Steffan |
| *Eulonchus smaragdinus* | 000510 | male | CAS | USA, California, Santa Barbara County, Santa Ynez Mountains, [34.549, -120.029], 24.VI.1959, W. A. Steffan |
| *Eulonchus smaragdinus* | 000511 | male | CAS | USA, California, Marin County, McCLure’s Beach, [38.189, -122.958], 8.VI.1957, A. E. Michelbacher |
| *Eulonchus smaragdinus* | 000512 | male | CAS | USA, California, Marin County, McCLure’s Beach, [38.189, -122.958], 8.VI.1957, A. E. Michelbacher |
| *Eulonchus smaragdinus* | 000513 | male | CAS | USA, California, Marin County, McCLure’s Beach, [38.189, -122.958], 8.VI.1957, A. E. Michelbacher |
| *Eulonchus smaragdinus* | 000514 | male | CAS | USA, California, Marin County, McCLure’s Beach, [38.189, -122.958], 8.VI.1957, A. E. Michelbacher |
| *Eulonchus smaragdinus* | 000515 | male | CAS | USA, California, Contra Costa County, Richmond, Point San Pablo, [37.964, -122.428], 5.VI.1964, P. A. Rude |
| *Eulonchus smaragdinus* | 000516 | male | CAS | USA, California, Marin County, McCLure’s Beach, [38.189, -122.958], 8.VI.1957, A. E. Michelbacher |
| *Eulonchus smaragdinus* | 000517 | male | CAS | USA, California, Contra Costa County, Richmond, Point San Pablo, [37.964, -122.428], 5.VI.1964, P. A. Rude |
| *Eulonchus smaragdinus* | 000518 | male | CAS | USA, California, Contra Costa County, Richmond, Point San Pablo, [37.964, -122.428], 5.VI.1964, P. A. Rude |
| *Eulonchus smaragdinus* | 000519 | male | CAS | USA, California, Contra Costa County, Richmond, Point San Pablo, [37.964, -122.428], 5.VI.1964, P. A. Rude |
| *Eulonchus smaragdinus* | 000520 | male | CAS | USA, California, Contra Costa County, Richmond, Point San Pablo, [37.964, -122.428], 5.VI.1964, P. A. Rude |
| *Eulonchus smaragdinus* | 000521 | male | CAS | USA, California, Contra Costa County, Richmond, Point San Pablo, [37.964, -122.428], 12.VI.1964, R. L. Langston |
| *Eulonchus smaragdinus* | 000522 | male | CAS | USA, California, Contra Costa County, Richmond, Point San Pablo, [37.964, -122.428], 12.VI.1964, R. L. Langston |
| *Eulonchus smaragdinus* | 000523 | male | CAS | USA, California, Contra Costa County, Richmond, Point San Pablo, [37.964, -122.428], 12.VI.1964, R. L. Langston |
| *Eulonchus smaragdinus* | 000524 | male | CAS | USA, California, Contra Costa County, Richmond, Point San Pablo, [37.964, -122.428], 3.VI.1964, P. A. Rude |
| *Eulonchus smaragdinus* | 000525 | male | CAS | USA, California, Contra Costa County, Richmond, Point San Pablo, [37.964, -122.428], 3.VI.1964, P. A. Rude |
| *Eulonchus smaragdinus* | 000526 | male | CAS | USA, California, Contra Costa County, Richmond, Point San Pablo, [37.964, -122.428], 3.VI.1964, P. A. Rude |
| *Eulonchus smaragdinus* | 000527 | male | CAS | USA, California, Contra Costa County, Richmond, Point San Pablo, [37.964, -122.428], 3.VI.1964, R. L. Langston |
| *Eulonchus smaragdinus* | 000528 | male | CAS | USA, California, Contra Costa County, Richmond, Point San Pablo, [37.964, -122.428], 12.VI.1964, R. L. Langston |
| *Eulonchus smaragdinus* | 000529 | male | CAS | USA, California, Contra Costa County, Richmond, Point San Pablo, [37.964, -122.428], 12.VI.1964, R. L. Langston |
| *Eulonchus smaragdinus* | 000530 | male | CAS | USA, California, Contra Costa County, Richmond, Point San Pablo, [37.964, -122.428], 12.VI.1964, R. L. Langston |
| *Eulonchus smaragdinus* | 000531 | male | CAS | USA, California, San Luis Obispo County, 9.7 km northeast Santa Margarita, [35.459, -120.543], 12.VI.1963, J. W. MacSwain |
| *Eulonchus smaragdinus* | 000532 | male | CAS | USA, California, San Luis Obispo County, 8.1 km northeast Santa Margarita, [35.446, -120.552], 9.VI.1962, P. D. Hurd |
| *Eulonchus smaragdinus* | 000533 | male | CAS | USA, California, San Luis Obispo County, 8.1 km northeast Santa Margarita, [35.446, -120.552], 9.VI.1962, P. D. Hurd |
| *Eulonchus smaragdinus* | 000534 | male | CAS | USA, California, San Luis Obispo County, 8.1 km northeast Santa Margarita, [35.446, -120.552], 10.VI.1962, P. D. Hurd |
| *Eulonchus smaragdinus* | 000535 | male | CAS | USA, California, San Luis Obispo County, 8.1 km northeast Santa Margarita, [35.446, -120.552], 10.VI.1962, P. D. Hurd |
| *Eulonchus smaragdinus* | 000536 | male | CAS | USA, California, San Luis Obispo County, 8.1 km northeast Santa Margarita, [35.446, -120.552], 10.VI.1962, P. D. Hurd |
| *Eulonchus smaragdinus* | 000537 | male | CAS | USA, California, San Luis Obispo County, 8.1 km northeast Santa Margarita, [35.446, -120.552], 10.VI.1962, P. D. Hurd |
| *Eulonchus smaragdinus* | 000538 | male | CAS | USA, California, Point Reyes Beach, [38.089, -122.957], 24.V.1969, P. A. Opler |
| *Eulonchus smaragdinus* | 000539 | male | CAS | USA, California, Monterey County, Arroyo Seco Campground, [36.232, -121.485], 5.V.1957 |
| *Eulonchus smaragdinus* | 000540 | male | CAS | USA, California, Arroyo Seco, [36.232, -121.485], 15.V.1960, T. N. Seeno |
| *Eulonchus smaragdinus* | 000541 | male | CAS | USA, California, Point Reyes Beach, [38.089, -122.957], 24.V.1969, P. A. Opler |
| *Eulonchus smaragdinus* | 000542 | male | CAS | USA, California, Point Reyes Beach, [38.089, -122.957], 24.V.1969, P. A. Opler |
| *Eulonchus smaragdinus* | 000543 | male | CAS | USA, California, San Luis Obispo County, 9.7 km northeast Santa Margarita, [35.459, -120.543], 12.VI.1963, J. W. MacSwain |
| *Eulonchus smaragdinus* | 000544 | male | CAS | USA, California, San Luis Obispo County, 9.7 km northeast Santa Margarita, [35.459, -120.543], 22.VI.1958, E. G. Linsley |
| *Eulonchus smaragdinus* | 000545 | male | CAS | USA, California, Point Reyes Beach, [38.089, -122.957], 24.V.1969, P. A. Opler |
| *Eulonchus smaragdinus* | 000546 | male | CAS | USA, California, Point Reyes Beach, [38.089, -122.957], 24.V.1969, P. A. Opler |
| *Eulonchus smaragdinus* | 000547 | male | CAS | USA, California, Santa Barbara County, Santa Ynez Mountains, [34.549, -120.029], 24.VI.1959, C. A. Campbell |
| *Eulonchus smaragdinus* | 000548 | male | CAS | USA, California, Arroyo Seco, [36.232, -121.485], 15.V.1960, T. N. Seeno |
| *Eulonchus smaragdinus* | 000549 | male | CAS | USA, California, Santa Barbara County, Santa Ynez Mountains, [34.549, -120.029], 24.VI.1959, C. A. Campbell |
| *Eulonchus smaragdinus* | 000550 | male | CAS | USA, California, Monterey County, 14.5 km south Big Sur, Partington Canyon, [36.176, -121.697], 14.V.1966, R. L. Langston |
| *Eulonchus smaragdinus* | 000551 | male | CAS | USA, California, Monterey County, 14.5 km south Big Sur, Partington Canyon, [36.176, -121.697], 15.V.1966, R. L. Langston |
| *Eulonchus smaragdinus* | 000552 | male | CAS | USA, California, Trinity County, 9.7 km west Zenia, [40.205, -123.501], 19.V.1966, J. A. Powell |
| *Eulonchus smaragdinus* | 000553 | male | CAS | USA, California, Santa Barbara County, Los Prietos, [34.541, -119.801], 12.VII.1965, J. A. Powell |
| *Eulonchus smaragdinus* | 000554 | male | CAS | USA, California, San Diego County, 8.1 km northeast Jamul, Lawson Valley Road, [32.747, -116.79], 26.VI.1965, R. L. Langston |
| *Eulonchus smaragdinus* | 000555 | male | CAS | USA, California, Monterey Co., Bixby Creek, [36.372, -121.9], 31.V.1954, M. S. Wasbauer |
| *Eulonchus smaragdinus* | 000556 | male | CAS | USA, California, Marin County, Point Reyes, [38.07, -122.812], 28.V.1967, P. A. Opler |
| *Eulonchus smaragdinus* | 000557 | male | CAS | USA, California, San Luis Obispo County, Morro Rock, 26.IV.1968, J. A. Powell |
| *Eulonchus smaragdinus* | 000558 | male | CAS | USA, California, Trinity County, 9.7 km west Zenia, [40.205, -123.501], 19.V.1966, J. A. Powell |
| *Eulonchus smaragdinus* | 000559 | male | CAS | USA, California, Monterey County, Los Padres National Forest, 4.8 km southeast Tassajara Springs, [36.23, -121.541], 18.IV.1971, L. Carpenter |
| *Eulonchus smaragdinus* | 000560 | male | CAS | USA, California, Trinity County, 9.7 km west Zenia, [40.205, -123.501], 19.V.1966, J. A. Powell |
| *Eulonchus smaragdinus* | 000561 | male | CAS | USA, California, Contra Costa County, Richmond, Point San Pablo, [37.964, -122.428], 12.VI.1964, R. L. Langston |
| *Eulonchus smaragdinus* | 000562 | male | CAS | USA, California, Contra Costa County, Richmond, Point San Pablo, [37.964, -122.428], 12.VI.1964, R. L. Langston |
| *Eulonchus smaragdinus* | 000563 | male | CAS | USA, California, Monterey County, Arroyo Seco Campground, [36.232, -121.485], 6.VI.1957, G. I. Stage |
| *Eulonchus smaragdinus* | 000564 | female | CAS | USA, California, Monterey County, Arroyo Seco Campground, [36.232, -121.485], 6.VI.1957, G. I. Stage |
| *Eulonchus smaragdinus* | 000565 | female | CAS | USA, California, Monterey County, Arroyo Seco Campground, [36.232, -121.485], 25.V.1958, T. N. Seeno |
| *Eulonchus smaragdinus* | 000566 | female | CAS | USA, California, Monterey County, Arroyo Seco Campground, [36.232, -121.485], 15.V.1969, T. N. Seeno |
| *Eulonchus smaragdinus* | 000567 | female | CAS | USA, California, Point Reyes Beach, [38.089, -122.957], 24.V.1969, P. A. Opler |
| *Eulonchus smaragdinus* | 000568 | female | CAS | USA, California, Point Reyes Beach, [38.089, -122.957], 24.V.1969, P. A. Opler |
| *Eulonchus smaragdinus* | 000569 | female | CAS | USA, California, Point Reyes Beach, [38.089, -122.957], 24.V.1969, P. A. Opler |
| *Eulonchus smaragdinus* | 000570 | female | CAS | USA, California, San Luis Obispo County, 8.1 km northeast Santa Margarita, [35.446, -120.552], 10.VI.1962, P. D. Hurd |
| *Eulonchus smaragdinus* | 000571 | female | CAS | USA, California, Contra Costa County, Richmond, Point San Pablo, [37.964, -122.428], 3.VI.1964, R. L. Langston |
| *Eulonchus smaragdinus* | 000572 | female | CAS | USA, California, Contra Costa County, Richmond, Point San Pablo, [37.964, -122.428], 12.VI.1964, R. L. Langston |
| *Eulonchus smaragdinus* | 000573 | female | CAS | USA, California, Marin County, McCLure’s Beach, [38.189, -122.958], 8.VI.1957, A. E. Michelbacher |
| *Eulonchus smaragdinus* | 000574 | female | CAS | USA, California, San Luis Obispo County, 9.7 km northeast Santa Margarita, [35.459, -120.543], 12.VI.1963, J. W. MacSwain |
| *Eulonchus smaragdinus* | 000575 | female | CAS | USA, California, Marin County, McCLure’s Beach, [38.189, -122.958], 31.V.1962, A. E. Michelbacher |
| *Eulonchus smaragdinus* | 000576 | female | CAS | USA, California, Marin County, McCLure’s Beach, [38.189, -122.958], 31.V.1962, A. E. Michelbacher |
| *Eulonchus smaragdinus* | 000577 | female | CAS | USA, California, Marin County, McCLure’s Beach, [38.189, -122.958], 31.V.1962, A. E. Michelbacher |
| *Eulonchus smaragdinus* | 000578 | female | CAS | USA, California, Siskiyou County, 8.1 km east McCloud, [41.256, -122.044], 14.VII.1962, J. A. Powell |
| *Eulonchus smaragdinus* | 000579 | female | CAS | USA, California, Los Angeles County, Tanbark Flat, [34.204, -117.761], 20.VI.1956, J. I. Stage |
| *Eulonchus smaragdinus* | 000580 | female | CAS | USA, California, Contra Costa County, Mount Diablo, [37.882, -121.914], 18.VI.1963, D. H. Janzen |
| *Eulonchus smaragdinus* | 000581 | female | CAS | USA, California, Kern County, 5.6 km east Alta Sierra, [39.142, -121.047], 4.VI.1965, P. H. Raven |
| *Eulonchus smaragdinus* | 000582 | female | CAS | USA, California, Santa Barbara County, Santa Ynez Mountains, [34.549, -120.029], 24.VI.1959, C. A. Campbell |
| *Eulonchus smaragdinus* | 000583 | female | CAS | USA, California, Marin County, Point Reyes, [38.07, -122.812], 28.V.1967, P. A. Opler |
| *Eulonchus smaragdinus* | 002227 | male | CAS | USA, California, Los Angeles, Westwood, [40.306, -121.003], E. G. Linsley |
| *Eulonchus smaragdinus* | 002228 | male | UCDC | USA, California, Cisco, [39.302, -120.547], 13.VI.1939, G. E. Bohart |
| *Eulonchus smaragdinus* | 002229 | male | UCDC | USA, California, Cisco, [39.302, -120.547], 13.VI.1939, G. E. Bohart |
| *Eulonchus smaragdinus* | 002230 | male | UCDC | USA, California, Cisco, [39.302, -120.547], 13.VI.1939, G. E. Bohart |
| *Eulonchus smaragdinus* | 002231 | male | UCDC | USA, California, Alameda County, Oakland, [37.804, -122.273], 12.VI.1933, G. E. Bohart |
| *Eulonchus smaragdinus* | 002232 | male | UCDC | USA, California, Mount Diablo, [37.905, -121.949], 10.V.1941, G. E. Bohart |
| *Eulonchus smaragdinus* | 002286 | male | CAS | USA, California, San Luis Obispo, [35.283, -120.659], 14.VI.1934, E. D. Ball |
| *Eulonchus smaragdinus* | 002287 | male | CAS | USA, California, San Luis Obispo, [35.283, -120.659], 14.VI.1934, E. D. Ball |
| *Eulonchus smaragdinus* | 004132 | unknown | CAS | USA, California, Santa Barbara County, 8.1 km west Ventucopa, Alamo Canyon, [34.83, -119.558], 29.V.1993, P. F. Torchio, G. Trostle, D. J. Burdick |
| *Eulonchus smaragdinus* | 004133 | unknown | CAS | USA, California, Santa Barbara County, 8.1 km west Ventucopa, Alamo Canyon, [34.83, -119.558], 29.V.1993, P. F. Torchio, G. Trostle, D. J. Burdick |
| *Eulonchus smaragdinus* | 004396 | male | CAS | MEXICO, Baja California, Ensenada, [31.859, -116.607], 6.IV.1966, S. Rubinkan |
| *Eulonchus smaragdinus* | 006744 | male | CAS | USA, California, Marin County, Mill Valley, =, [37.906, -122.545], 6.V.1965 to 10.V.1965, P. H. Arnaud, Jr. |
| *Eulonchus smaragdinus* | 006745 | male | CAS | USA, California, Marin County, Mill Valley, =, [37.906, -122.545], 28.V.1965, P. H. Arnaud, Jr. |
| *Eulonchus smaragdinus* | 006746 | male | CAS | USA, California, Marin County, Mill Valley, =, [37.906, -122.545], 19.VII.1965 to 22.VII.1965, P. H. Arnaud, Jr. |
| *Eulonchus smaragdinus* | 006747 | male | CAS | USA, California, Marin County, Mill Valley, =, [37.906, -122.545], 23.VII.1965 to 25.VII.1965, P. H. Arnaud, Jr. |
| *Eulonchus smaragdinus* | 006748 | male | CAS | USA, California, Marin County, Mill Valley, [37.906, -122.545], 110 m, .VIII.1965, flight trapP. H. Arnaud, Jr. |
| *Eulonchus smaragdinus* | 006749 | male | CAS | USA, California, Marin County, Mill Valley, =, [37.906, -122.545], 28.V.1965, P. H. Arnaud, Jr. |
| *Eulonchus smaragdinus* | 006750 | female | CAS | USA, California, Marin County, Mill Valley, [37.906, -122.545], 110 m, .VIII.1965, flight trapP. H. Arnaud, Jr. |
| *Eulonchus smaragdinus* | 006751 | male | CAS | USA, California, Marin County, Mill Valley, =, [37.906, -122.545], 19.VII.1965 to 22.VII.1965, P. H. Arnaud, Jr. |
| *Eulonchus smaragdinus* | 006752 | male | CAS | USA, California, Marin County, Mill Valley, =, [37.906, -122.545], 19.VII.1965 to 22.VII.1965, P. H. Arnaud, Jr. |
| *Eulonchus smaragdinus* | 006753 | male | CAS | USA, California, Marin County, Mill Valley, =, [37.906, -122.545], 28.V.1965, P. H. Arnaud, Jr. |
| *Eulonchus smaragdinus* | 006754 | male | CAS | USA, California, Marin County, Mill Valley, [37.906, -122.545], 110 m, .VIII.1965, flight trapP. H. Arnaud, Jr. |
| *Eulonchus smaragdinus* | 006755 | male | CAS | USA, California, Marin County, Mill Valley, =, [37.906, -122.545], 19.VII.1965 to 22.VII.1965, P. H. Arnaud, Jr. |
| *Eulonchus smaragdinus* | 006756 | male | CAS | USA, California, Marin County, Mill Valley, =, [37.906, -122.545], 19.VII.1965 to 22.VII.1965, P. H. Arnaud, Jr. |
| *Eulonchus smaragdinus* | 006757 | male | CAS | USA, California, Marin County, Mill Valley, =, [37.906, -122.545], 23.VII.1965 to 25.VII.1965, P. H. Arnaud, Jr. |
| *Eulonchus smaragdinus* | 006758 | male | CAS | USA, California, Marin County, Lake Lagunitas, [37.947, -122.596], 24.VI.1958, D. C. Rentz |
| *Eulonchus smaragdinus* | 006759 | male | CAS | USA, California, Marin County, Lake Lagunitas, [37.947, -122.596], 24.VI.1958, D. C. Rentz |
| *Eulonchus smaragdinus* | 006760 | male | CAS | USA, California, Marin County, Mill Valley, =, [37.906, -122.545], 28.V.1965, P. H. Arnaud, Jr. |
| *Eulonchus smaragdinus* | 006761 | male | CAS | USA, California, Marin County, Mill Valley, =, [37.906, -122.545], 19.VII.1965 to 22.VII.1965, P. H. Arnaud, Jr. |
| *Eulonchus smaragdinus* | 006762 | male | CAS | USA, California, Marin County, Mill Valley, [37.906, -122.545], 110 m, 9.VII.1965 to 12.VII.1965, P. H. Arnaud, Jr. |
| *Eulonchus smaragdinus* | 006763 | male | CAS | USA, California, Marin County, Mill Valley, [37.906, -122.545], 110 m, 9.VII.1965 to 12.VII.1965, P. H. Arnaud, Jr. |
| *Eulonchus smaragdinus* | 006764 | male | CAS | USA, California, Marin County, Mill Valley, [37.906, -122.545], 110 m, 9.VII.1965 to 12.VII.1965, P. H. Arnaud, Jr. |
| *Eulonchus smaragdinus* | 006765 | male | CAS | USA, California, Marin County, Mill Valley, [37.906, -122.545], 110 m, 9.VII.1965 to 12.VII.1965, P. H. Arnaud, Jr. |
| *Eulonchus smaragdinus* | 006766 | male | CAS | USA, California, Marin County, Mill Valley, [37.906, -122.545], 110 m, 9.VII.1965 to 12.VII.1965, P. H. Arnaud, Jr. |
| *Eulonchus smaragdinus* | 006767 | male | CAS | USA, California, Marin County, Mill Valley, [37.906, -122.545], 110 m, 9.VII.1965 to 12.VII.1965, P. H. Arnaud, Jr. |
| *Eulonchus smaragdinus* | 006768 | male | CAS | USA, California, Marin County, Mill Valley, [37.906, -122.545], 110 m, 9.VII.1965 to 12.VII.1965, P. H. Arnaud, Jr. |
| *Eulonchus smaragdinus* | 006769 | female | CAS | USA, California, Marin County, Mill Valley, [37.906, -122.545], 110 m, 13.VII.1965 to 15.VII.1965, P. H. Arnaud, Jr. |
| *Eulonchus smaragdinus* | 006770 | male | CAS | USA, California, Marin County, Mill Valley, [37.906, -122.545], 110 m, 9.VII.1965 to 12.VII.1965, P. H. Arnaud, Jr. |
| *Eulonchus smaragdinus* | 006771 | male | CAS | USA, California, Marin County, Mill Valley, [37.906, -122.545], 110 m, 9.VII.1965 to 12.VII.1965, P. H. Arnaud, Jr. |
| *Eulonchus smaragdinus* | 006772 | male | CAS | USA, California, Marin County, Mill Valley, [37.906, -122.545], 110 m, 9.VII.1965 to 12.VII.1965, P. H. Arnaud, Jr. |
| *Eulonchus smaragdinus* | 006773 | male | CAS | USA, California, Marin County, Mill Valley, [37.906, -122.545], 110 m, 13.VII.1965 to 15.VII.1965, P. H. Arnaud, Jr. |
| *Eulonchus smaragdinus* | 006774 | male | CAS | USA, California, Marin County, Mill Valley, [37.906, -122.545], 110 m, 13.VII.1965 to 15.VII.1965, P. H. Arnaud, Jr. |
| *Eulonchus smaragdinus* | 006775 | male | CAS | USA, California, Marin County, Mill Valley, [37.906, -122.545], 110 m, 9.VII.1965 to 12.VII.1965, P. H. Arnaud, Jr. |
| *Eulonchus smaragdinus* | 006776 | male | CAS | USA, California, Marin County, Mill Valley, [37.906, -122.545], 110 m, 9.VII.1965 to 12.VII.1965, P. H. Arnaud, Jr. |
| *Eulonchus smaragdinus* | 006777 | male | CAS | USA, California, Marin County, Mill Valley, [37.906, -122.545], 110 m, 13.VII.1965 to 15.VII.1965, P. H. Arnaud, Jr. |
| *Eulonchus smaragdinus* | 006778 | male | CAS | USA, California, Marin County, Mill Valley, [37.906, -122.545], 110 m, 13.VII.1965 to 15.VII.1965, P. H. Arnaud, Jr. |
| *Eulonchus smaragdinus* | 006779 | male | CAS | USA, California, Marin County, Mill Valley, [37.906, -122.545], 110 m, 13.VII.1965 to 15.VII.1965, P. H. Arnaud, Jr. |
| *Eulonchus smaragdinus* | 006780 | male | CAS | USA, California, Marin County, Mill Valley, [37.906, -122.545], 110 m, 13.VII.1965 to 15.VII.1965, P. H. Arnaud, Jr. |
| *Eulonchus smaragdinus* | 006781 | male | CAS | USA, California, Marin County, Mill Valley, [37.906, -122.545], 110 m, 9.VII.1965 to 12.VII.1965, P. H. Arnaud, Jr. |
| *Eulonchus smaragdinus* | 006782 | male | CAS | USA, California, Marin County, Mill Valley, [37.906, -122.545], 110 m, 9.VII.1965 to 12.VII.1965, P. H. Arnaud, Jr. |
| *Eulonchus smaragdinus* | 006783 | male | CAS | USA, California, Marin County, Mill Valley, [37.906, -122.545], 110 m, 9.VII.1965 to 12.VII.1965, P. H. Arnaud, Jr. |
| *Eulonchus smaragdinus* | 006784 | male | CAS | USA, California, Marin County, Mill Valley, [37.906, -122.545], 110 m, 13.VII.1965 to 15.VII.1965, P. H. Arnaud, Jr. |
| *Eulonchus smaragdinus* | 006785 | male | CAS | USA, California, Marin County, Mill Valley, [37.906, -122.545], 110 m, 13.VII.1965 to 15.VII.1965, P. H. Arnaud, Jr. |
| *Eulonchus smaragdinus* | 006786 | female | CAS | USA, California, Marin County, Mill Valley, [37.906, -122.545], 110 m, 13.VII.1965 to 15.VII.1965, P. H. Arnaud, Jr. |
| *Eulonchus smaragdinus* | 006787 | female | CAS | USA, California, Marin County, Mill Valley, [37.906, -122.545], 110 m, 22.V.1965 to 24.V.1965, flight trapP. H. Arnaud, Jr. |
| *Eulonchus smaragdinus* | 006788 | female | CAS | USA, California, Marin County, Mill Valley, =, [37.906, -122.545], 19.VII.1965 to 22.VII.1965, P. H. Arnaud, Jr. |
| *Eulonchus smaragdinus* | 006789 | female | CAS | USA, California, Fairfax, [37.987, -122.589], 18.VI.1939, E. C. Van Dyke |
| *Eulonchus smaragdinus* | 006790 | male | CAS | USA, California, Monterey County, Pebble Beach, [36.566, -121.946], 27.V.1921, E. C. Van Dyke |
| *Eulonchus smaragdinus* | 006791 | male | CAS | USA, California, Monterey County, Pebble Beach, [36.566, -121.946], 27.V.1921, E. C. Van Dyke |
| *Eulonchus smaragdinus* | 006792 | male | CAS | USA, California, Monterey County, Pebble Beach, [36.566, -121.946], 27.V.1921, E. C. Van Dyke |
| *Eulonchus smaragdinus* | 006793 | female | CAS | USA, California, Monterey County, Pebble Beach, [36.566, -121.946], 27.V.1921, E. C. Van Dyke |
| *Eulonchus smaragdinus* | 006794 | female | CAS | USA, California, Monterey County, Pebble Beach, [36.566, -121.946], 27.V.1921, E. C. Van Dyke |
| *Eulonchus smaragdinus* | 006795 | male | CAS | USA, California, Monterey County, Pebble Beach, [36.566, -121.946], 27.V.1921, E. C. Van Dyke |
| *Eulonchus smaragdinus* | 006796 | male | CAS | USA, California, Monterey County, Carmel, [36.555, -121.924], 22.VI.1924, L. S. Slevin |
| *Eulonchus smaragdinus* | 006797 | male | CAS | USA, California, Monterey County, Carmel, [36.555, -121.924], 22.VI.1924, L. S. Slevin |
| *Eulonchus smaragdinus* | 006798 | female | CAS | USA, California, Monterey County, Carmel, [36.555, -121.924], 15.V.1908, L. S. Slevin |
| *Eulonchus smaragdinus* | 006799 | male | CAS | USA, California, San Francisco, [37.774, -122.42], .V.1905, F. X. Williams |
| *Eulonchus smaragdinus* | 006800 | male | CAS | USA, California, San Francisco, [37.774, -122.42], .V.1905, F. X. Williams |
| *Eulonchus smaragdinus* | 006801 | male | CAS | USA, California, San Francisco, [37.774, -122.42], .V.1905, F. X. Williams |
| *Eulonchus smaragdinus* | 006802 | male | CAS | USA, California, San Francisco, [37.774, -122.42], .V.1905, F. X. Williams |
| *Eulonchus smaragdinus* | 006803 | male | CAS | USA, California, San Francisco, [37.774, -122.42], .V.1905, F. X. Williams |
| *Eulonchus smaragdinus* | 006804 | male | CAS | USA, California, San Francisco, [37.774, -122.42], .V.1905, F. X. Williams |
| *Eulonchus smaragdinus* | 006805 | male | CAS | USA, California, Marin County, Mill Valley, =, [37.906, -122.545], 19.VI.1950, R. E. Leech |
| *Eulonchus smaragdinus* | 006806 | male | CAS | USA, California, Marin County, Mill Valley, =, [37.906, -122.545], 25.VI.1950, R. E. Leech |
| *Eulonchus smaragdinus* | 006807 | male | CAS | USA, California, Marin County, Mill Valley, =, [37.906, -122.545], 30.VI.1950, R. E. Leech |
| *Eulonchus smaragdinus* | 006808 | male | CAS | USA, California, Marin County, Mount Tamalpais, [37.924, -122.596], 20.VI.1909, E. C. Van Dyke |
| *Eulonchus smaragdinus* | 006809 | male | CAS | USA, California, Pt. Reyes Peninsula, AT&T station, [38.082, -122.955], 18.VI.1966, D. Wielgus |
| *Eulonchus smaragdinus* | 006810 | male | CAS | USA, California, Monterey County, 14.5 km south Big Sur, Partington Canyon, [36.176, -121.697], 14.V.1964, R. M. Brown |
| *Eulonchus smaragdinus* | 006811 | male | CAS | USA, California, San Luis Obispo, [35.283, -120.659], .VI.1938, I. McCracken |
| *Eulonchus smaragdinus* | 006812 | male | CAS | USA, California, San Luis Obispo, [35.283, -120.659], .VI.1938, I. McCracken |
| *Eulonchus smaragdinus* | 006813 | male | CAS | USA, California, San Luis Obispo County, Baywood Park, Morro Bay, [35.366, -120.85], 9.VI.1963, G. I. Stage |
| *Eulonchus smaragdinus* | 006814 | male | CAS | USA, California, Mount Diablo, [37.882, -121.914], 610 m, 26.VI.1951, F. X. Williams |
| *Eulonchus smaragdinus* | 006815 | male | CAS | USA, California, Mount Diablo, [37.882, -121.914], 610 m, 26.VI.1951, F. X. Williams |
| *Eulonchus smaragdinus* | 006816 | male | CAS | USA, California, Mount Diablo, [37.882, -121.914], 610 m, 26.VI.1951, F. X. Williams |
| *Eulonchus smaragdinus* | 006817 | male | CAS | USA, California, Mount Diablo, [37.882, -121.914], 610 m, 18.VI.1951, F. X. Williams |
| *Eulonchus smaragdinus* | 006818 | male | CAS | USA, California, Mount Diablo, [37.882, -121.914], 610 m, 18.VI.1951, F. X. Williams |
| *Eulonchus smaragdinus* | 006819 | male | CAS | USA, California, Mount Diablo, [37.882, -121.914], 610 m, 18.VI.1951, F. X. Williams |
| *Eulonchus smaragdinus* | 006820 | male | CAS | USA, California, Mount Diablo, [37.882, -121.914], 610 m, 18.VI.1951, F. X. Williams |
| *Eulonchus smaragdinus* | 006821 | male | CAS | USA, California, Mount Diablo, [37.882, -121.914], 610 m, 14.VI.1949, F. X. Williams |
| *Eulonchus smaragdinus* | 006822 | male | CAS | USA, California, Santa Monica, [34.02, -118.491], 20.VI.1935, E. G. Linsley |
| *Eulonchus smaragdinus* | 006823 | male | CAS | USA, California, San Bernardino County, Forest Home, [34.101, -116.997], 19.VI.1928, E. C. Van Dyke |
| *Eulonchus smaragdinus* | 006824 | male | CAS | USA, California, Sonoma County, [38.292, -122.458] |
| *Eulonchus smaragdinus* | 006825 | male | CAS | USA, California, Sonoma County, [38.292, -122.458] |
| *Eulonchus smaragdinus* | 006826 | male | CAS | USA, California, Sonoma County, Sobre Vista, [38.333, -122.511], .VII.1911, A. Kusche |
| *Eulonchus smaragdinus* | 006827 | male | CAS | USA, California, Sonoma County, [38.292, -122.458] |
| *Eulonchus smaragdinus* | 006831 | male | CAS | USA, California, San Francisco County, [37.776, -122.42], 7.V.1911, J. A. Kusche |
| *Eulonchus smaragdinus* | 006832 | male | CAS | USA, California, San Francisco, [37.774, -122.42], 26.IV.1927, J. A. Kusche |
| *Eulonchus smaragdinus* | 006833 | male | CAS | USA, California, San Francisco, [37.774, -122.42], 26.IV.1925, J. A. Kusche |
| *Eulonchus smaragdinus* | 006834 | male | CAS | USA, California, San Francisco County, [37.776, -122.42], F. E. Blaisdell |
| *Eulonchus smaragdinus* | 006835 | male | CAS | USA, California, San Francisco County, [37.776, -122.42], 7.V.1911, J. A. Kusche |
| *Eulonchus smaragdinus* | 006836 | male | CAS | USA, California, San Francisco, [37.774, -122.42], .V.1905, F. X. Williams |
| *Eulonchus smaragdinus* | 006837 | male | CAS | USA, California, San Francisco County, [37.776, -122.42], F. E. Blaisdell |
| *Eulonchus smaragdinus* | 006838 | male | CAS | USA, California, San Francisco, [37.774, -122.42], 21.V.1922, C. L. Fox |
| *Eulonchus smaragdinus* | 006839 | male | CAS | USA, California, south San Fransisco, [37.725, -122.434], 4.VI.1922, C. L. Fox |
| *Eulonchus smaragdinus* | 006840 | female | CAS | USA, California, San Francisco County, [37.776, -122.42], 7.V.1911, J. A. Kusche |
| *Eulonchus smaragdinus* | 006841 | male | CAS | USA, California, San Francisco, [37.774, -122.42], .V.1905, F. X. Williams |
| *Eulonchus smaragdinus* | 006842 | male | CAS | USA, California, south San Fransisco, [37.725, -122.434], 4.VI.1922, C. L. Fox |
| *Eulonchus smaragdinus* | 006843 | male | CAS | USA, California, San Francisco, [37.774, -122.42], 30.IV.1911, J. A. Kusche |
| *Eulonchus smaragdinus* | 006844 | male | CAS | USA, California, San Francisco County, [37.776, -122.42], 7.V.1911, J. A. Kusche |
| *Eulonchus smaragdinus* | 006845 | male | CAS | USA, California, San Francisco County, [37.776, -122.42], .V.1920, F. E. Blaisdell |
| *Eulonchus smaragdinus* | 006846 | male | CAS | USA, California, San Francisco, [37.774, -122.42], 26.IV.1925, J. A. Kusche |
| *Eulonchus smaragdinus* | 006847 | male | CAS | USA, California, San Francisco County, [37.776, -122.42], 7.V.1911, J. A. Kusche |
| *Eulonchus smaragdinus* | 006848 | male | CAS | USA, California, San Francisco, Lone Mountain, [37.778, -122.454], .VI.1925, F. X. Williams |
| *Eulonchus smaragdinus* | 006849 | female | CAS | USA, California, San Francisco County, [37.776, -122.42], F. E. Blaisdell |
| *Eulonchus smaragdinus* | 006850 | male | CAS | USA, California, San Francisco County, [37.776, -122.42], 7.V.1911, J. A. Kusche |
| *Eulonchus smaragdinus* | 006851 | male | CAS | USA, California, Los Gatos, [37.236, -121.963], 27.VII.1933, J. A. Kusche |
| *Eulonchus smaragdinus* | 006852 | male | CAS | USA, California, Mount Diablo, [37.882, -121.914], 610 m, 18.VI.1951, F. X. Williams |
| *Eulonchus smaragdinus* | 006853 | male | CAS | USA, California, Mount Diablo, [37.882, -121.914], 610 m, 18.VI.1951, F. X. Williams |
| *Eulonchus smaragdinus* | 006854 | male | CAS | USA, California, Mount Diablo, [37.882, -121.914], 610 m, 18.VI.1951, F. X. Williams |
| *Eulonchus smaragdinus* | 006855 | male | CAS | USA, California, Mount Diablo, [37.882, -121.914], 610 m, 18.VI.1951, F. X. Williams |
| *Eulonchus smaragdinus* | 006856 | male | CAS | USA, California, Mount Diablo, [37.882, -121.914], 610 m, 18.VI.1951, F. X. Williams |
| *Eulonchus smaragdinus* | 006857 | male | CAS | USA, California, Mount Diablo, [37.882, -121.914], 610 m, 14.VI.1949, F. X. Williams |
| *Eulonchus smaragdinus* | 006858 | male | CAS | USA, California, Riverside County, Gavilan Hills, [33.804, -117.376], 26.V.1935, A. J. Basinger |
| *Eulonchus smaragdinus* | 006859 | male | CAS | USA, California, Riverside County, The Gavilan, [33.442, -117.213], 26.V.1935 |
| *Eulonchus smaragdinus* | 006860 | female | CAS | USA, California, Sonoma County, [38.292, -122.458] |
| *Eulonchus smaragdinus* | 006861 | male | CAS | MEXICO, Baja California, San Quintin, [30.56, -115.943], 30.III.1972, C. H. Bruemmer |
| *Eulonchus smaragdinus* | 006862 | male | CAS | USA, California, Banning, [33.926, -116.877], 30.V.1928, E. C. Van Dyke |
| *Eulonchus smaragdinus* | 007865 | male | CAS | USA, California, Santa Barbara County, Live Oak Park, Cachuma Lake, [34.579, -119.948], 15.VI.1996, E. I. Schlinger |
| *Eulonchus smaragdinus* | 008336 | male | CAS | USA, California, Inyo County, Whitney Portal, [36.589, -118.227], 3.VII.1953, J. W. MacSwain |
| *Eulonchus smaragdinus* | 008714 | unknown | CAS | USA, California, Monrovia Canyon, [34.174, -117.99], 12.VII.1930, C. H. Martin, D. Martin |
| *Eulonchus smaragdinus* | 008715 | unknown | CAS | USA, California, Monrovia Canyon, [34.174, -117.99], 4.VII.1930, C. H. Martin |
| *Eulonchus smaragdinus* | 008716 | unknown | CAS | MEXICO, Baja California, 11.5 km N. of El Rosario, [30.074, -115.761], 7.V.1977, R. L. Westcott |
| *Eulonchus smaragdinus* | 009019 | unknown | CAS | USA, California, Monrovia Canyon, [34.174, -117.99], .VII., Cole |
| *Eulonchus smaragdinus* | 009070 | unknown | CAS | USA, California, Mendocino County, NCCRP (Northern California Coast Range Preserve), 4.8 km N of Branscomb, [39.697, -123.626], 427 m, time of day: midafternoon, 21.V.1982 to 23.V.1982, E. I. Schlinger |
| *Eulonchus smaragdinus* | 009071 | unknown | CAS | USA, California, Mendocino County, NCCRP (Northern California Coast Range Preserve), 4.8 km N of Branscomb, [39.697, -123.626], 427 m, time of day: midafternoon, 21.V.1982 to 23.V.1982, E. I. Schlinger |
| *Eulonchus smaragdinus* | 009072 | unknown | CAS | USA, California, Mendocino County, NCCRP (Northern California Coast Range Preserve), 4.8 km N of Branscomb, [39.697, -123.626], 427 m, time of day: midafternoon, 21.V.1982 to 23.V.1982, E. I. Schlinger |
| *Eulonchus smaragdinus* | 009168 | unknown | CAS | USA, California, Monrovia Canyon, [34.174, -117.99], 4.VII.1930 |
| *Eulonchus smaragdinus* | 009428 | unknown | CAS | USA, California, Siskiyou County, 1.6 km. NW of Bartle, [41.272, -121.842], 20.VII.1966, Opler, P. A. |
| *Eulonchus smaragdinus* | 011560 | unknown | CAS | USA, California, Marin, Mt. Tamalpais, [37.924, -122.596], 7.II.1953 |
| *Eulonchus smaragdinus* | 012199 | male | CAS | USA, California, San Mateo Co., Campus Experimental Area, [37.405, -122.242], 26.IV.1969, A. R. Moldenke |
| *Eulonchus smaragdinus* | 012200 | female | CAS | USA, California, San Mateo Co., Campus Experimental Area, [37.405, -122.242], 26.IV.1969, A. R. Moldenke |
| *Eulonchus smaragdinus* | 012201 | male | CAS | USA, California, Marin County, Point Reyes National Seashore, Sand dunes, [38.105, -122.959], 0 to 100 m, ..1970, A. R. Moldenke |
| *Eulonchus smaragdinus* | 012739 | male | CAS | USA, California, Napa County, 11 miles east of Rutherford, [38.459, -122.412], 30.V.1968, P. Welies |
| *Eulonchus smaragdinus* | 012740 | male | CAS | USA, California, Napa County, 11 miles east of Rutherford, [38.459, -122.412], 30.V.1968, P. Welies |
| *Eulonchus smaragdinus* | 012741 | male | CAS | USA, California, Napa County, 11 miles east of Rutherford, [38.459, -122.412], 30.V.1968, P. Welies |
| *Eulonchus smaragdinus* | 012742 | male | CAS | USA, California, Napa County, 11 miles east of Rutherford, [38.459, -122.412], 30.V.1968, P. Welies |
| *Eulonchus smaragdinus* | 012743 | male | CAS | USA, California, Napa County, 11 miles east of Rutherford, [38.459, -122.412], 30.V.1968, P. Welies |
| *Eulonchus smaragdinus* | 012744 | male | CAS | USA, California, Napa County, 11 miles east of Rutherford, [38.459, -122.412], 30.V.1968, P. Welies |
| *Eulonchus smaragdinus* | 012745 | male | CAS | USA, California, Napa County, 11 miles east of Rutherford, [38.459, -122.412], 30.V.1968, P. Welies |
| *Eulonchus smaragdinus* | 012746 | male | CAS | USA, California, Napa County, 11 miles east of Rutherford, [38.459, -122.412], 30.V.1968, P. Welies |
| *Eulonchus smaragdinus* | 012750 | male | CAS | USA, California, Napa County, Conn Dam, 30.V.1968, P. Welies |
| *Eulonchus smaragdinus* | 012751 | male | CAS | USA, California, Napa County, Conn Dam, 30.V.1968, P. Welies |
| *Eulonchus smaragdinus* | 012752 | male | CAS | USA, California, Napa County, Conn Dam, 30.V.1968, P. Welies |
| *Eulonchus smaragdinus* | 012753 | male | CAS | USA, California, Napa County, Conn Dam, 30.V.1968, P. Welies |
| *Eulonchus smaragdinus* | 012754 | male | CAS | USA, California, Napa County, Conn Dam, 30.V.1968, P. Welies |
| *Eulonchus smaragdinus* | 012755 | male | CAS | USA, California, Napa County, Conn Dam, 30.V.1968, P. Welies |
| *Eulonchus smaragdinus* | 012756 | male | CAS | USA, California, Napa County, Conn Dam, 30.V.1968, P. Welies |
| *Eulonchus smaragdinus* | 012757 | male | CAS | USA, California, Napa County, Conn Dam, 30.V.1968, P. Welies |
| *Eulonchus smaragdinus* | 012758 | male | CAS | USA, California, Napa County, Conn Dam, 30.V.1968, P. Welies |
| *Eulonchus smaragdinus* | 012759 | male | CAS | USA, California, Napa County, Conn Dam, 30.V.1968, P. Welies |
| *Eulonchus smaragdinus* | 012760 | male | CAS | USA, California, Napa County, Conn Dam, 30.V.1968, P. Welies |
| *Eulonchus smaragdinus* | 012761 | male | CAS | USA, California, Napa County, Conn Dam, 30.V.1968, P. Welies |
| *Eulonchus smaragdinus* | 012762 | male | CAS | USA, California, Napa County, Conn Dam, 30.V.1968, P. Welies |
| *Eulonchus smaragdinus* | 012763 | male | CAS | USA, California, Napa County, Conn Dam, 30.V.1968, P. Welies |
| *Eulonchus smaragdinus* | 012764 | female | CAS | USA, California, Napa County, Conn Dam, 30.V.1968, P. Welies |
| *Eulonchus smaragdinus* | 012765 | female | CAS | USA, California, Napa County, Conn Dam, 30.V.1968, P. Welies |
| *Eulonchus smaragdinus* | 012966 | female | CAS | USA, California, Napa County, 7 miles east of Conn Dam, [38.489, -122.376], 12.VI.1964, R. W. Thorp |
| *Eulonchus smaragdinus* | 012967 | female | CAS | USA, California, Napa County, 7 miles east of Conn Dam, [38.489, -122.376], 12.VI.1964, R. W. Thorp |
| *Eulonchus smaragdinus* | 012968 | female | CAS | USA, California, Napa County, 7 miles east of Conn Dam, [38.489, -122.376], 12.VI.1964, R. W. Thorp |
| *Eulonchus smaragdinus* | 012969 | male | CAS | USA, California, Napa County, 7 miles east of Conn Dam, [38.489, -122.376], 12.VI.1964, R. W. Thorp |
| *Eulonchus smaragdinus* | 012970 | male | CAS | USA, California, Napa County, 7 miles east of Conn Dam, [38.489, -122.376], 12.VI.1964, R. W. Thorp |
| *Eulonchus smaragdinus* | 012971 | male | CAS | USA, California, Napa County, 7 miles east of Conn Dam, [38.489, -122.376], 12.VI.1964, R. W. Thorp |
| *Eulonchus smaragdinus* | 012972 | male | CAS | USA, California, Napa County, 7 miles east of Conn Dam, [38.489, -122.376], 12.VI.1964, R. W. Thorp |
| *Eulonchus smaragdinus* | 012973 | male | CAS | USA, California, Napa County, 7 miles east of Conn Dam, [38.489, -122.376], 12.VI.1964, R. W. Thorp |
| *Eulonchus smaragdinus* | 012985 | male | CAS | USA, California, San Luis Obispo Co., 5 miles North East of Santa Margarita, [35.394, -120.606], 9.VI.1965, J. W. MacSwain |
| *Eulonchus smaragdinus* | 012986 | male | CAS | USA, California, San Luis Obispo Co., 5 miles North East of Santa Margarita, [35.394, -120.606], 9.VI.1965, J. W. MacSwain |
| *Eulonchus smaragdinus* | 012987 | male | CAS | USA, California, San Luis Obispo Co., 5 miles North East of Santa Margarita, [35.394, -120.606], 9.VI.1965, J. W. MacSwain |
| *Eulonchus smaragdinus* | 012988 | female | CAS | USA, California, San Luis Obispo Co., 5 miles North East of Santa Margarita, [35.394, -120.606], 9.VI.1965, J. W. MacSwain |
| *Eulonchus smaragdinus* | 012989 | male | CAS | USA, California, Napa County, 11 miles east of Rutherford, [38.459, -122.412], 15.VI.1965, R. W. Thorp |
| *Eulonchus smaragdinus* | 012990 | male | CAS | USA, California, Napa County, 11 miles east of Rutherford, [38.459, -122.412], 15.VI.1965, R. W. Thorp |
| *Eulonchus smaragdinus* | 012991 | male | CAS | USA, California, Kern Co., near Caliente, oiler Canyon, [35.308, -118.584], 4.VI.1965, P. H. Raven |
| *Eulonchus smaragdinus* | 012992 | male | CAS | USA, California, Kern Co., near Caliente, oiler Canyon, [35.308, -118.584], 4.VI.1965, P. H. Raven |
| *Eulonchus smaragdinus* | 012993 | male | CAS | USA, California, Kern Co., near Caliente, oiler Canyon, [35.308, -118.584], 4.VI.1965, P. H. Raven |
| *Eulonchus smaragdinus* | 013067 | unknown | CAS | USA, California, Mendocino County, NCCRP (Northern California Coast Range Preserve), 4.8 km North of Branscomb, [39.699, -123.627], time of day: midafternoon, 20.V.1985 to 24.V.1985, N. J. Atkinson |
| *Eulonchus smaragdinus* | 013069 | unknown | CAS | USA, California, Mendocino County, NCCRP (Northern California Coast Range Preserve), 4.8 km North of Branscomb, [39.699, -123.627], time of day: midafternoon, 20.V.1985 to 24.V.1985, N. J. Atkinson |
| *Eulonchus smaragdinus* | 013073 | unknown | CAS | USA, California, Mendocino County, NCCRP (Northern California Coast Range Preserve), 4.8 km North of Branscomb, [39.699, -123.627], time of day: midafternoon, 20.V.1985 to 24.V.1985, N. J. Atkinson |
| *Eulonchus smaragdinus* | 013074 | unknown | CAS | USA, California, Mendocino County, NCCRP (Northern California Coast Range Preserve), 4.8 km North of Branscomb, [39.699, -123.627], time of day: midafternoon, 20.V.1985 to 24.V.1985, N. J. Atkinson |
| *Eulonchus smaragdinus* | 013084 | unknown | CAS | USA, California, Mendocino County, NCCRP (Northern California Coast Range Preserve), 4.8 km North of Branscomb, [39.699, -123.627], time of day: midafternoon, 20.V.1985 to 24.V.1985, N. J. Atkinson |
| *Eulonchus smaragdinus* | 013085 | unknown | CAS | USA, California, Mendocino County, NCCRP (Northern California Coast Range Preserve), 4.8 km North of Branscomb, [39.699, -123.627], time of day: midafternoon, 20.V.1985 to 24.V.1985, N. J. Atkinson |
| *Eulonchus smaragdinus* | 013102 | unknown | CAS | USA, California, Monterey County, Big Creek Reserve, 8.1 km. N. of Lucia, Landels-Hill, [36.093, -121.549], time of day: midafternoon, 4.VI.1982 to 6.VI.1982, E. I. Schlinger |
| *Eulonchus smaragdinus* | 013103 | unknown | CAS | USA, California, Monterey County, Big Creek Reserve, 8.1 km. N. of Lucia, Landels-Hill, [36.093, -121.549], time of day: midafternoon, 4.VI.1982 to 6.VI.1982, E. I. Schlinger |
| *Eulonchus smaragdinus* | 013104 | unknown | CAS | USA, California, Monterey County, Big Creek Reserve, 8.1 km. N. of Lucia, Landels-Hill, [36.093, -121.549], time of day: midafternoon, 4.VI.1982 to 6.VI.1982, E. I. Schlinger |
| *Eulonchus smaragdinus* | 013105 | unknown | CAS | USA, California, Monterey County, Big Creek Reserve, 8.1 km. N. of Lucia, Landels-Hill, [36.093, -121.549], time of day: midafternoon, 4.VI.1982 to 6.VI.1982, E. I. Schlinger |
| *Eulonchus smaragdinus* | 013106 | unknown | CAS | USA, California, Mendocino County, NCCRP (Northern California Coast Range Preserve), 4.8 km N of Branscomb, [39.697, -123.626], 427 m, time of day: midafternoon, 21.V.1982 to 23.V.1982, E. I. Schlinger |
| *Eulonchus smaragdinus* | 013115 | unknown | CAS | USA, California, Mendocino County, NCCRP (Northern California Coast Range Preserve), 4.8 km N of Branscomb, [39.697, -123.626], 427 m, time of day: midafternoon, 21.V.1982 to 23.V.1982, E. I. Schlinger |
| *Eulonchus smaragdinus* | 013165 | unknown | CAS | USA, California, Mendocino County, NCCRP (Northern California Coast Range Preserve), 4.8 km N of Branscomb, [39.697, -123.626], 427 m, time of day: midafternoon, 21.V.1982 to 23.V.1982, E. I. Schlinger |
| *Eulonchus smaragdinus* | 013897 | male | CAS | USA, California, Monterey County, Point Lobos Reserve, Cypress Point, [36.523, -121.952], 22.V.1978, hand netted, A. E. Hajek, M. E. Buegler |
| *Eulonchus smaragdinus* | 013898 | male | CAS | USA, California, Monterey County, Point Lobos Reserve, Cypress Point, [36.523, -121.952], 22.V.1978, hand netted, A. E. Hajek, M. E. Buegler |
| *Eulonchus smaragdinus* | 013899 | male | CAS | USA, California, Monterey County, Point Lobos Reserve, Cypress Point, [36.523, -121.952], 22.V.1978, hand netted, A. E. Hajek, M. E. Buegler |
| *Eulonchus smaragdinus* | 014075 | unknown | CAS | USA, California, Marin County, Mill Valley, =, [37.906, -122.545], 3.VII.1965 to 6.VII.1965, flight trap P. H. Arnaud, Jr. |
| *Eulonchus smaragdinus* | 014076 | unknown | CAS | USA, California, Marin County, Mill Valley, =, [37.906, -122.545], 3.VII.1965 to 6.VII.1965, flight trap P. H. Arnaud, Jr. |
| *Eulonchus smaragdinus* | 014077 | unknown | CAS | USA, California, Marin County, Mill Valley, =, [37.906, -122.545], 3.VII.1965 to 6.VII.1965, flight trap P. H. Arnaud, Jr. |
| *Eulonchus smaragdinus* | 014078 | unknown | CAS | USA, California, Marin County, Mill Valley, =, [37.906, -122.545], 3.VII.1965 to 6.VII.1965, flight trap P. H. Arnaud, Jr. |
| *Eulonchus smaragdinus* | 014079 | unknown | CAS | USA, California, Marin County, Mill Valley, =, [37.906, -122.545], 3.VII.1965 to 6.VII.1965, flight trap P. H. Arnaud, Jr. |
| *Eulonchus smaragdinus* | 014080 | unknown | CAS | USA, California, Marin County, Mill Valley, =, [37.906, -122.545], 3.VII.1965 to 6.VII.1965, flight trap P. H. Arnaud, Jr. |
| *Eulonchus smaragdinus* | 014081 | unknown | CAS | USA, California, Marin County, Mill Valley, =, [37.906, -122.545], 3.VII.1965 to 6.VII.1965, flight trap P. H. Arnaud, Jr. |
| *Eulonchus smaragdinus* | 014082 | unknown | CAS | USA, California, Marin County, Mill Valley, =, [37.906, -122.545], 3.VII.1965 to 6.VII.1965, flight trap P. H. Arnaud, Jr. |
| *Eulonchus smaragdinus* | 014083 | unknown | CAS | USA, California, Marin County, Mill Valley, =, [37.906, -122.545], 3.VII.1965 to 6.VII.1965, flight trap P. H. Arnaud, Jr. |
| *Eulonchus smaragdinus* | 014084 | unknown | CAS | USA, California, Marin County, Mill Valley, =, [37.906, -122.545], 3.VII.1965 to 6.VII.1965, flight trap P. H. Arnaud, Jr. |
| *Eulonchus smaragdinus* | 014086 | unknown | CAS | USA, California, Marin County, Mill Valley, =, [37.906, -122.545], 3.VII.1965 to 6.VII.1965, flight trap P. H. Arnaud, Jr. |
| *Eulonchus smaragdinus* | 014096 | unknown | CAS | USA, California, Marin County, Mill Valley, =, [37.906, -122.545], 3.VII.1965 to 6.VII.1965, flight trap P. H. Arnaud, Jr. |
| *Eulonchus smaragdinus* | 014097 | unknown | CAS | USA, California, Marin County, Mill Valley, =, [37.906, -122.545], 3.VII.1965 to 6.VII.1965, flight trap P. H. Arnaud, Jr. |
| *Eulonchus smaragdinus* | 014103 | unknown | CAS | USA, California, Marin County, Mill Valley, =, [37.906, -122.545], 7.VII.1965 to 8.VII.1965, flight trap P. H. Arnaud, Jr. |
| *Eulonchus smaragdinus* | 014104 | unknown | CAS | USA, California, Marin County, Mill Valley, =, [37.906, -122.545], 7.VII.1965 to 8.VII.1965, flight trap P. H. Arnaud, Jr. |
| *Eulonchus smaragdinus* | 014105 | unknown | CAS | USA, California, Marin County, Mill Valley, =, [37.906, -122.545], 7.VII.1965 to 8.VII.1965, flight trap P. H. Arnaud, Jr. |
| *Eulonchus smaragdinus* | 014106 | unknown | CAS | USA, California, Marin County, Mill Valley, =, [37.906, -122.545], 7.VII.1965 to 8.VII.1965, flight trap P. H. Arnaud, Jr. |
| *Eulonchus smaragdinus* | 014107 | unknown | CAS | USA, California, Marin County, Mill Valley, =, [37.906, -122.545], 7.VII.1965 to 8.VII.1965, flight trap P. H. Arnaud, Jr. |
| *Eulonchus smaragdinus* | 014110 | unknown | CAS | USA, California, Marin County, Mill Valley, [37.906, -122.545], 110 m, 26.V.1965 to 27.V.1965, flight trap P. H. Arnaud, Jr. |
| *Eulonchus smaragdinus* | 014111 | unknown | CAS | USA, California, Marin County, Mill Valley, [37.906, -122.545], 110 m, 26.V.1965 to 27.V.1965, flight trap P. H. Arnaud, Jr. |
| *Eulonchus smaragdinus* | 014112 | unknown | CAS | USA, California, Los Angeles County, nr. Glendora, Big Dalton Canyon, [34.152, -117.836], 1.V.1936, H. L. McKenzie |
| *Eulonchus smaragdinus* | 014118 | unknown | CAS | USA, California, Riverside County, Box Springs Mountain, [33.962, -117.281], 28.V.1966, R. Sunderman |
| *Eulonchus smaragdinus* | 014121 | unknown | CAS | USA, California, Marin County, Point Reyes National Seashore, [38.043, -122.788], 30 m, 16.V.1958, J. A. Powell |
| *Eulonchus smaragdinus* | 014122 | unknown | CAS | USA, California, San Bernardino, San Antonio Can., [34.213, -117.663], 19.VI.1959, D. L. Tiemann |
| *Eulonchus smaragdinus* | 014123 | unknown | CAS | USA, California, San Bernardino County, Wildwood Canyon, [34.024, -116.994], 11.VI.1958, H. R. Moffitt |
| *Eulonchus smaragdinus* | 014124 | unknown | CAS | USA, California, Santa Barbara County, Santa Ynez Mountains, [34.549, -120.029], 24.VI.1959, J. L. Bath |
| *Eulonchus smaragdinus* | 014125 | unknown | CAS | USA, California, Santa Barbara County, Santa Ynez Mountains, [34.549, -120.029], 24.VI.1959, J. L. Bath |
| *Eulonchus smaragdinus* | 014126 | unknown | CAS | USA, California, Santa Barbara County, Santa Ynez Mountains, [34.549, -120.029], 24.VI.1959, J. L. Bath |
| *Eulonchus smaragdinus* | 014127 | unknown | CAS | USA, California, Santa Barbara County, Santa Ynez Mountains, [34.549, -120.029], 24.VI.1959, J. L. Bath |
| *Eulonchus smaragdinus* | 014128 | male | CAS | USA, California, Riverside County, Gavilan Hills, [33.804, -117.376], 18.V.1939, hand netted, P. H. Timberlake |
| *Eulonchus smaragdinus* | 014129 | male | CAS | USA, California, Riverside County, Gavilan Hills, [33.804, -117.376], 2.VI.1938, P. H. Timberlake |
| *Eulonchus smaragdinus* | 014130 | male | CAS | USA, California, Riverside County, Gavilan Hills, [33.804, -117.376], 26.V.1935, hand netted, C. M. Dammulis (?) |
| *Eulonchus smaragdinus* | 014131 | male | CAS | USA, California, Riverside County, Gavilan Hills, [33.804, -117.376], 26.V.1935, hand netted, C. M. Dammulis (?) |
| *Eulonchus smaragdinus* | 014132 | male | CAS | USA, California, Riverside County, Gavilan Hills, [33.804, -117.376], 26.V.1935, hand netted, C. M. Dammulis (?) |
| *Eulonchus smaragdinus* | 014133 | female | CAS | USA, California, San Francisco Co., San Francisco, [37.78, -122.42], .VI.1904, F. X. Williams |
| *Eulonchus smaragdinus* | 014134 | male | CAS | USA, California, Riverside County, 1 1/2 mi. N. of Perris, [33.789, -117.229], 27..1938, hand netted, P. H. Timberlake |
| *Eulonchus smaragdinus* | 014135 | male | CAS | USA, California, Tamalpais, [37.905, -122.604], 20.VI.1936, hand netted, P. H. Timberlake |
| *Eulonchus smaragdinus* | 014136 | male | CAS | USA, California, Tamalpais, [37.905, -122.604], 20.VI.1936, hand netted, P. H. Timberlake |
| *Eulonchus smaragdinus* | 014137 | male | CAS | USA, California, Tamalpais, [37.905, -122.604], 20.VI.1936, hand netted, P. H. Timberlake |
| *Eulonchus smaragdinus* | 014138 | female | CAS | USA, California, Riverside County, Gavilan Hills, [33.804, -117.376], 18.V.1939, hand netted, P. H. Timberlake |
| *Eulonchus smaragdinus* | 014139 | female | CAS | USA, California, Riverside County, Gavilan Hills, [33.804, -117.376], 18.V.1939, hand netted, P. H. Timberlake |
| *Eulonchus smaragdinus* | 014140 | female | CAS | USA, California, Riverside County, Gavilan Hills, [33.804, -117.376], 18.V.1939, hand netted, P. H. Timberlake |
| *Eulonchus smaragdinus* | 014141 | female | CAS | USA, California, Riverside County, Gavilan Hills, [33.804, -117.376], 31.V.1937, hand netted, P. H. Timberlake |
| *Eulonchus smaragdinus* | 014546 | unknown | CAS | USA, California, Solano County, 8 Km WNW Vacaville, Gates Cyn, nr. merge of Alamo Creek & S. Fork Alamo Creek, [38.382, -122.038], 335 m, 14.VI.1994 to 20.VI.1994, Malaise trap, S. D. Gaimari |
| *Eulonchus smaragdinus* | 014572 | unknown | CAS | USA, California, Santa Barbara County, La Purisima Mission State Park, Lompoc, Sand Dunes, [34.678, -120.433], 26.VI.1996, hand netted, M. B. Schlinger |
| *Eulonchus smaragdinus* | 014573 | unknown | CAS | USA, California, Santa Barbara County, La Purisima Mission State Park, Lompoc, Sand Dunes, [34.678, -120.433], 26.VI.1996, hand netted, M. B. Schlinger |
| *Eulonchus smaragdinus* | 014574 | unknown | CAS | USA, California, Santa Barbara County, Gaviota State Park, area around Gaviota pass , [34.485, -120.229], 23.VI.1996, hand netted, M. B. Schlinger |
| *Eulonchus smaragdinus* | 014604 | male | CAS | USA, California, Riverside County, Santa Rosa Mountains, 2.VI.1987, K. W. Cooper |
| *Eulonchus smaragdinus* | 014605 | male | CAS | USA, California, Riverside County, Santa Rosa Mountains, 2.VI.1987, J. C. Hall |
| *Eulonchus smaragdinus* | 014606 | male | CAS | USA, California, Riverside County, Santa Rosa Mountains, 2.VI.1987, J. C. Hall |
| *Eulonchus smaragdinus* | 014607 | male | CAS | USA, California, Riverside County, Santa Rosa Mountains, 2.VI.1987, J. C. Hall |
| *Eulonchus smaragdinus* | 014608 | male | CAS | USA, California, Riverside County, Santa Rosa Mountains, 2.VI.1987, J. C. Hall |
| *Eulonchus smaragdinus* | 014609 | male | CAS | USA, California, Riverside County, Santa Rosa Mountains, 2.VI.1987, J. C. Hall |
| *Eulonchus smaragdinus* | 014611 | male | CAS | USA, California, Riverside County, Santa Rosa Mountains, 2.VI.1987, K. W. Cooper |
| *Eulonchus smaragdinus* | 014612 | female | CAS | USA, California, Los Angeles County, Burbank, 7.VIII.1957, F. P. Sala |
| *Eulonchus smaragdinus* | 014613 | female | CAS | USA, California, Monterey County, San Simeon, Hwy route 1, 30.V.1955, F. P. Sala |
| *Eulonchus smaragdinus* | 014614 | female | CAS | USA, California, Riverside County, T7N R5E S28, 28.V.1985, M. Narog |
| *Eulonchus smaragdinus* | 014615 | male | CAS | USA, California, Kern County, Pinyon Mountain, 4.VI.1987, G. R. Ballmer |
| *Eulonchus smaragdinus* | 014616 | male | CAS | USA, California, Kern County, Pinyon Mountain, 4.VI.1987, G. R. Ballmer |
| *Eulonchus smaragdinus* | 014617 | male | CAS | USA, California, Monterey County, Marina Dunes, [36.698, -121.807], 9.IV.1992, G. R. Ballmer |
| *Eulonchus smaragdinus* | 014618 | male | CAS | USA, California, Riverside County, San Bernardino National Forest, San Jacinto Mountains, Thomas Mountain, [33.62, -116.68], 14.VI.1984, G. R. Ballmer |
| *Eulonchus smaragdinus* | 014619 | male | CAS | USA, California, Los Angeles County, Chilao, 6.VI.1960, |
| *Eulonchus smaragdinus* | 014668 | unknown | CAS | USA, California, Monterey County, Los Padres National Forest, 1.6 km W Nacimiento , Summit Campground, [34.597, -119.511], 8.VII.1963, P. H. Arnaud, Jr. |
| *Eulonchus smaragdinus* | 014670 | unknown | CAS | USA, California, Monterey County, Los Padres National Forest, 1.6 km W Nacimiento , Summit Campground, [34.597, -119.511], 8.VII.1963, P. H. Arnaud, Jr. |
| *Eulonchus smaragdinus* | 014671 | unknown | CAS | USA, California, Monterey County, Los Padres National Forest, 1.6 km W Nacimiento , Summit Campground, [34.597, -119.511], 8.VII.1963, P. H. Arnaud, Jr. |
| *Eulonchus smaragdinus* | 014672 | unknown | CAS | USA, California, Monterey County, Los Padres National Forest, 1.6 km W Nacimiento , Summit Campground, [34.597, -119.511], 8.VII.1963, P. H. Arnaud, Jr. |
| *Eulonchus smaragdinus* | 014673 | unknown | CAS | USA, California, Monterey County, Los Padres National Forest, 1.6 km W Nacimiento , Summit Campground, [34.597, -119.511], 8.VII.1963, P. H. Arnaud, Jr. |
| *Eulonchus smaragdinus* | 014674 | unknown | CAS | USA, California, Monterey County, Los Padres National Forest, 1.6 km W Nacimiento , Summit Campground, [34.597, -119.511], 8.VII.1963, P. H. Arnaud, Jr. |
| *Eulonchus smaragdinus* | 014675 | unknown | CAS | USA, California, Monterey County, Los Padres National Forest, 1.6 km W Nacimiento , Summit Campground, [34.597, -119.511], 8.VII.1963, P. H. Arnaud, Jr. |
| *Eulonchus smaragdinus* | 014676 | unknown | CAS | USA, California, Ventura County, Wagon Road, No. 2 Campground, [34.694, -119.254], 1585 m, 5.VII.1968, P. H. Arnaud, Jr. |
| *Eulonchus smaragdinus* | 014770 | unknown | CAS | USA, California, Marin County, Mill Valley, [37.906, -122.545], 110 m, 24.V.1965 to 25.V.1965, Malaise trap, P. H. Arnaud, Jr. |
| *Eulonchus smaragdinus* | 014772 | unknown | CAS | USA, California, Marin County, Mill Valley, [37.906, -122.545], 110 m, 24.V.1965 to 25.V.1965, Malaise trap, P. H. Arnaud, Jr. |
| *Eulonchus smaragdinus* | 014773 | unknown | CAS | USA, California, Marin County, Mill Valley, [37.906, -122.545], 110 m, 24.V.1965 to 25.V.1965, Malaise trap, P. H. Arnaud, Jr. |
| *Eulonchus smaragdinus* | 014790 | unknown | CAS | USA, California, Monterey County, Arroyo Seco, The Lakes, [36.231, -121.486], 1280 m, 12.IV.1997, Malaise trap, P. H. Arnaud, Jr., M. M. Arnaud |
| *Eulonchus smaragdinus* | 014796 | unknown | CAS | USA, California, Sonoma County, Bodega Bay, dunes, [38.344, -123.058], 15.V.1990, R. Robertson, J. K. Robertson |
| *Eulonchus smaragdinus* | 015519 | male | CAS | USA, California, Napa County, Samuel Springs, [38.604, -122.311], 7.VI.1953, R. C. Bechtel |
| *Eulonchus smaragdinus* | 015531 | unknown | CAS | USA, California, Mendocino County, Mendocino National Forest, at Middle Fork Eel River, Rattlesnake Creek, [39.823, -123.655], 19.VI.1976, D. D. Wilder |
| *Eulonchus smaragdinus* | 015618 | unknown | CAS | USA, California, Monterey County, Horse Bridge, 2.4 air-km SW Arroyo Seco Guard Station, [36.214, -121.501], 396 m, 7.V.1975, J. A. Chemsak |
| *Eulonchus smaragdinus* | 015619 | unknown | CAS | USA, California, Monterey County, Horse Bridge, 2.4 air-km SW Arroyo Seco Guard Station, [36.214, -121.501], 396 m, 7.V.1975, J. A. Chemsak |
| *Eulonchus smaragdinus* | 015620 | unknown | CAS | USA, California, Monterey County, Horse Bridge, 2.4 air-km SW Arroyo Seco Guard Station, [36.214, -121.501], 396 m, 7.V.1975, J. A. Chemsak |
| *Eulonchus smaragdinus* | 015621 | unknown | CAS | USA, California, Monterey County, Horse Bridge, 2.4 air-km SW Arroyo Seco Guard Station, [36.214, -121.501], 396 m, 7.V.1975, J. A. Chemsak |
| *Eulonchus smaragdinus* | 015627 | unknown | CAS | USA, California, Monterey County, Indiana Road, 4.8 air-km S Arroyo Seco Guard Station, [36.189, -121.482], 823 m, 7.V.1975, J. A. Powell |
| *Eulonchus smaragdinus* | 015628 | unknown | CAS | USA, California, Monterey County, Horse Bridge, 2.4 air-km SW Arroyo Seco Guard Station, [36.214, -121.501], 396 m, 6.V.1975, hand netted, R. Wharton |
| *Eulonchus smaragdinus* | 015629 | unknown | CAS | USA, California, Monterey County, Horse Bridge, 2.4 air-km SW Arroyo Seco Guard Station, [36.214, -121.501], 396 m, 6.V.1975, hand netted, R. Wharton |
| *Eulonchus smaragdinus* | 015630 | unknown | CAS | USA, California, Monterey County, Horse Bridge, 2.4 air-km SW Arroyo Seco Guard Station, [36.214, -121.501], 396 m, 6.V.1975, hand netted, R. Wharton |
| *Eulonchus smaragdinus* | 015631 | unknown | CAS | USA, California, San Luis Obispo County, 4.8 km S. Oceano, Dune Lakes, [35.056, -120.611], 12.VII.1973, hand netted, R. Coville |
| *Eulonchus smaragdinus* | 015634 | unknown | CAS | USA, California, Monterey County, Horse Bridge, 2.4 air-km SW Arroyo Seco Guard Station, [36.214, -121.501], 396 m, 6.V.1975, J. A. Chemsak |
| *Eulonchus smaragdinus* | 015635 | unknown | CAS | USA, California, Monterey County, Horse Bridge, 2.4 air-km SW Arroyo Seco Guard Station, [36.214, -121.501], 396 m, 6.V.1975, J. A. Chemsak |
| *Eulonchus smaragdinus* | 015808 | unknown | CAS | USA, California, Mendocino County, 1.6 km N Eel River Ranger Station, [39.842, -123.083], 853 to 945 m, 13.VI.1972, hand netted, J. T. Doyen |
| *Eulonchus smaragdinus* | 015809 | unknown | CAS | USA, California, Mendocino County, 1.6 km N Eel River Ranger Station, [39.842, -123.083], 853 to 945 m, 13.VI.1972, hand netted, J. T. Doyen |
| *Eulonchus smaragdinus* | 015811 | unknown | CAS | USA, California, Los Angeles County, Angeles National Forest, San Gabriel Mountains, Millard Canyon, [34.216, -118.146], 24.VI.1958, R. H. Crandall |
| *Eulonchus smaragdinus* | 015812 | unknown | CAS | USA, California, Los Angeles County, Angeles National Forest, San Gabriel Mountains, Millard Canyon, [34.216, -118.146], 24.VI.1958, R. H. Crandall |
| *Eulonchus smaragdinus* | 015813 | unknown | CAS | USA, California, Los Angeles County, Angeles National Forest, San Gabriel Mountains, Millard Canyon, [34.216, -118.146], 24.VI.1958, R. H. Crandall |
| *Eulonchus smaragdinus* | 015939 | unknown | CAS | USA, California, Mendocino County, 1.6 km N Eel River Ranger Station, [39.842, -123.083], 853 to 945 m, 13.VI.1972, hand netted, J. T. Doyen |
| *Eulonchus smaragdinus* | 015940 | unknown | CAS | USA, California, Mendocino County, 1.6 km N Eel River Ranger Station, [39.842, -123.083], 853 to 945 m, 13.VI.1972, hand netted, J. T. Doyen |
| *Eulonchus smaragdinus* | 015941 | unknown | CAS | USA, California, Mendocino County, 4 km N Eel River Ranger Station, [39.863, -123.082], 1341 m, 14.VI.1972, J. A. Chemsak |
| *Eulonchus smaragdinus* | 015942 | unknown | CAS | USA, California, Mendocino County, 3.2 air-km N Howard Lake, [39.909, -122.989], 1143 m, 10.VI.1972, J. A. Powell |
| *Eulonchus smaragdinus* | 015943 | unknown | CAS | USA, California, Mendocino County, 3.2 air-km N Howard Lake, [39.909, -122.989], 1143 m, 10.VI.1972, J. A. Powell |
| *Eulonchus smaragdinus* | 015944 | unknown | CAS | USA, California, Mendocino County, 3.2 air-km N Howard Lake, [39.909, -122.989], 1143 m, 10.VI.1972, J. A. Powell |
| *Eulonchus smaragdinus* | 015945 | unknown | CAS | USA, California, Mendocino County, 3.2 air-km N Howard Lake, [39.909, -122.989], 1143 m, 10.VI.1972, J. A. Powell |
| *Eulonchus smaragdinus* | 015946 | unknown | CAS | USA, California, Mendocino County, 3.2 air-km N Howard Lake, [39.909, -122.989], 1143 m, 10.VI.1972, J. A. Powell |
| *Eulonchus smaragdinus* | 015969 | unknown | CAS | USA, California, Mendocino County, 4.8 km SE Paul M. Dimmick State Park, Navarro River, [39.155, -123.643], 25.VII.1971 to 30.VII.1971, P. A. Rude |
| *Eulonchus smaragdinus* | 015970 | unknown | CAS | USA, California, Mendocino County, 4.8 km SE Paul M. Dimmick State Park, Navarro River, [39.155, -123.643], 25.VII.1971 to 30.VII.1971, P. A. Rude |
| *Eulonchus smaragdinus* | 015972 | unknown | CAS | USA, California, Mendocino County, 1.6 air-km N Eel River Ranger Station, [39.841, -123.084], 853 to 945 m, 13.VI.1972, hand netted, J. T. Doyen |
| *Eulonchus smaragdinus* | 015974 | unknown | CAS | USA, California, Mendocino County, 6.4 air-km N Eel River Ranger Station, Ham Pass, [39.885, -123.083], 1341 m, 14.VI.1972, hand netted, S. L. Szerlip |
| *Eulonchus smaragdinus* | 015975 | unknown | CAS | USA, California, Mendocino County, 6.4 air-km N Eel River Ranger Station, Ham Pass, [39.885, -123.083], 1341 m, 14.VI.1972, hand netted, S. L. Szerlip |
| *Eulonchus smaragdinus* | 015976 | unknown | CAS | USA, California, Mendocino County, 6.4 air-km N Eel River Ranger Station, Ham Pass, [39.885, -123.083], 1341 m, 15.VI.1972, hand netted, J. A. Powell |
| *Eulonchus smaragdinus* | 015978 | unknown | CAS | USA, California, Mendocino County, 11.3 mi NE Eel River Ranger Station, [39.905, -122.998], 1097 m, 11.VI.1972, hand netted, R. Coville |
| *Eulonchus smaragdinus* | 015979 | unknown | CAS | USA, California, Mendocino County, 1.6 air-km N Eel River Ranger Station, [39.841, -123.084], 853 to 945 m, 13.VI.1972, hand netted, R. Coville |
| *Eulonchus smaragdinus* | 015980 | unknown | CAS | USA, California, Mendocino County, 1.6 air-km N Eel River Ranger Station, [39.841, -123.084], 853 to 945 m, 13.VI.1972, hand netted, R. Coville |
| *Eulonchus smaragdinus* | 015981 | unknown | CAS | USA, California, Mendocino County, 1.6 km N Eel River Ranger Station, [39.842, -123.083], 853 to 945 m, 13.VI.1972, hand netted, J. A. Chemsak |
| *Eulonchus smaragdinus* | 015982 | unknown | CAS | USA, California, Mendocino County, 1.6 km N Eel River Ranger Station, [39.842, -123.083], 853 to 945 m, 13.VI.1972, hand netted, J. A. Chemsak |
| *Eulonchus smaragdinus* | 015983 | unknown | CAS | USA, California, Mendocino County, 1.6 km N Eel River Ranger Station, [39.842, -123.083], 853 to 945 m, 13.VI.1972, hand netted, J. A. Chemsak |
| *Eulonchus smaragdinus* | 015984 | unknown | CAS | USA, California, Mendocino County, 11.3 mi NE Eel River Ranger Station, [39.905, -122.998], 1097 m, 11.VI.1972, hand netted, J. A. Chemsak |
| *Eulonchus smaragdinus* | 015985 | unknown | CAS | USA, California, Mendocino County, 11.3 mi NE Eel River Ranger Station, [39.905, -122.998], 1097 m, 11.VI.1972, hand netted, J. A. Chemsak |
| *Eulonchus smaragdinus* | 015986 | unknown | CAS | USA, California, Mendocino County, 11.3 mi NE Eel River Ranger Station, [39.905, -122.998], 1097 m, 11.VI.1972, hand netted, J. A. Chemsak |
| *Eulonchus smaragdinus* | 015987 | unknown | CAS | USA, California, Mendocino County, 11.3 mi NE Eel River Ranger Station, [39.905, -122.998], 1097 m, 11.VI.1972, hand netted, R. Coville |
| *Eulonchus smaragdinus* | 015988 | unknown | CAS | USA, California, Mendocino County, 11.3 mi NE Eel River Ranger Station, [39.905, -122.998], 1097 m, 11.VI.1972, hand netted, R. Coville |
| *Eulonchus smaragdinus* | 015996 | male | CAS | USA, California, Tulare County, Kaweah, Ash Mountain, Powerhouse #3, 4.VI.1983, hand netted, R.D. Haines |
| *Eulonchus smaragdinus* | 015997 | male | CAS | USA, California, Tulare County, Kaweah, Ash Mountain, Powerhouse #3, 4.VI.1983, hand netted, R.D. Haines |
| *Eulonchus smaragdinus* | 015998 | male | CAS | USA, California, Tulare County, Kaweah, Ash Mountain, Powerhouse #3, 4.VI.1983, hand netted, R.D. Haines |
| *Eulonchus smaragdinus* | 015999 | unknown | CAS | USA, California, San Luis Obispo County, Montana de Oro, 14.VII.1973, hand netted, R.D. Haines |
| *Eulonchus smaragdinus* | 016000 | unknown | CAS | USA, California, San Luis Obispo County, Montana de Oro, 14.VII.1973, hand netted, R.D. Haines |
| *Eulonchus smaragdinus* | 016001 | unknown | CAS | USA, California, San Luis Obispo County, Montana de Oro, 14.VII.1973, hand netted, R.D. Haines |
| *Eulonchus smaragdinus* | 016002 | unknown | CAS | USA, California, Santa Clara County, Los Altos Hills, 26.VI.1983, hand netted, R.D. Haines |
| *Eulonchus smaragdinus* | 016003 | unknown | CAS | USA, California, Santa Clara County, Los Altos Hills, 26.VI.1983, hand netted, R.D. Haines |
| *Eulonchus smaragdinus* | 016004 | unknown | CAS | USA, California, Santa Clara County, Los Altos Hills, 26.VI.1983, hand netted, R.D. Haines |
| *Eulonchus smaragdinus* | 016695 | male | CAS | USA, California, Los Angeles County, Charmlee Park, [34.05, -118.88], 381 m, 27.VI.1996 to 6.VII.1996, Malaise trap, B. V. Brown |
| *Eulonchus smaragdinus* | 016696 | unknown | CAS | USA, California, Riverside County, 19.3 km S Corona, Indian Truck Trail, [33.74, -117.46], 410 m, 20.V.1996, hand netted, R. R. Snelling |
| *Eulonchus smaragdinus* | 016697 | unknown | CAS | USA, California, Riverside County, 19.3 km S Corona, Indian Truck Trail, [33.74, -117.46], 410 m, 20.V.1996, hand netted, R. R. Snelling |
| *Eulonchus smaragdinus* | 016698 | unknown | CAS | USA, California, Riverside County, 19.3 km S Corona, Indian Truck Trail, [33.74, -117.46], 410 m, 20.V.1996, hand netted, R. R. Snelling |
| *Eulonchus smaragdinus* | 016699 | unknown | CAS | USA, California, Riverside County, 19.3 km S Corona, Indian Truck Trail, [33.74, -117.46], 410 m, 20.V.1996, hand netted, R. R. Snelling |
| *Eulonchus smaragdinus* | 017871 | unknown | CAS | USA, California, Tuolumne County, Stanislaus National Forest, between Kennedy Meadows and Relief Reservoir, [38.237, -120.065], 2.VII.1979, hand netted, R. Kelson |
| *Eulonchus smaragdinus* | 017952 | unknown | CAS | USA, California, Siskiyou County, 8.1 to 16.1 km N.E. of Bartle, [41.262, -121.818], 20.VI.1954, hand netted, J. C. Downey |
| *Eulonchus smaragdinus* | 018329 | female | CAS | USA, California, San Diego County, Vista, [33.2, -117.243], 16.VI.1966, Metcalf |
| *Eulonchus smaragdinus* | 018330 | male | CAS | USA, California, San Diego County, Vista, [33.2, -117.243], 16.VI.1966, Metcalf |
| *Eulonchus smaragdinus* | 018401 | unknown | CAS | USA, California, Riverside County, Riverside, Box Spring Canyon, [33.954, -117.397], 30.V.1976, J. Trager, C. W. Young |
| *Eulonchus smaragdinus* | 018436 | unknown | CAS | USA, California, Marin County, Point Reyes National Seashore, Point Reyes, 0.2 km E. McCLure Beach, [38.188, -122.956], 5.VI.1979, R. W. Brooks |
| *Eulonchus smaragdinus* | 018437 | unknown | CAS | USA, California, Marin County, Point Reyes National Seashore, Point Reyes, 0.2 km E. McCLure Beach, [38.188, -122.956], 5.VI.1979, R. W. Brooks |
| *Eulonchus smaragdinus* | 018438 | unknown | CAS | USA, California, Marin County, Point Reyes National Seashore, Point Reyes, 0.2 km E. McCLure Beach, [38.188, -122.956], 5.VI.1979, R. W. Brooks |
| *Eulonchus smaragdinus* | 018439 | unknown | CAS | USA, California, Marin County, Point Reyes National Seashore, Point Reyes, 0.2 km E. McCLure Beach, [38.188, -122.956], 5.VI.1979, R. W. Brooks |
| *Eulonchus smaragdinus* | 018445 | unknown | CAS | USA, California, Riverside County, Murrieta, [33.554, -117.214], 14.VI.1983, |
| *Eulonchus smaragdinus* | 018460 | unknown | CAS | USA, California, Ventura County, 17.7 km E Camp Ozena, [34.694, -119.328], 2.VII.1965, J. R. Stephenson |
| *Eulonchus smaragdinus* | 018613 | unknown | CAS | USA, California, Tulare County, Smokey Valley Canyon, [35.917, -118.154], 1829 m, 12.VI.1945 |
| *Eulonchus smaragdinus* | 018679 | male | CAS | USA, California, Los Angeles County, North of Ontario, San Antonio Canyon, 1204 m, 20.VI.1931, H. A. Scullen |
| *Eulonchus smaragdinus* | 018680 | male | CAS | USA, California, Los Angeles County, North of Ontario, San Antonio Canyon, 1204 m, 20.VI.1931, H. A. Scullen |
| *Eulonchus smaragdinus* | 018686 | male | CAS | USA, California, Los Angeles County, North of Ontario, San Antonio Canyon, 1204 m, 20.VI.1931, H. A. Scullen |
| *Eulonchus smaragdinus* | 018711 | male | CAS | USA, California, Los Angeles County, Santa Monica Mountains, 2.VI.1962, M. Thompson |
| *Eulonchus smaragdinus* | 018844 | female | CAS | USA, California, Riverside County, San Jacinto Mountains, Ribbonwood, 22.V.1940, hand netted, |
| *Eulonchus smaragdinus* | 018845 | male | CAS | USA, California, Monterey County, Carmel, [36.555, -121.924], 22.VI.1924, L. S. Slevin |
| *Eulonchus smaragdinus* | 018846 | male | CAS | USA, California, San Jacinto Mountains, Herkey Creek, [33.675, -116.682], 20.VI.1940 |
| *Eulonchus smaragdinus* | 018847 | male | CAS | USA, California, Monterey County, Pebble Beach, [36.566, -121.946], 27.V.1921, E. C. Van Dyke |
| *Eulonchus smaragdinus* | 018930 | unknown | CAS | MEXICO, Baja California, San Quintin, south end dunes, [30.56, -115.942], 25.V.1988, R. Mattoni |
| *Eulonchus smaragdinus* | 018931 | unknown | CAS | MEXICO, Baja California, San Quintin, south end dunes, [30.56, -115.942], 17.V.1992, R. Mattoni |
| *Eulonchus smaragdinus* | 018933 | unknown | CAS | USA, California, Tulare County, summit of Bald Mountain, near lookout tower, [41.609, -120.27], 17.VI.1989, R. Rogers |
| *Eulonchus smaragdinus* | 018934 | unknown | CAS | USA, California, Tulare County, 11.3 km N Bald Mountain, Beach Creek Trail, [41.625, -120.271], 26.VI.1993, R. Rogers |
| *Eulonchus smaragdinus* | 018935 | unknown | CAS | USA, California, Tulare County, near summit of Bald Mountain, [41.609, -120.267], 26.VI.1993, R. Rogers |
| *Eulonchus smaragdinus* | 018936 | unknown | CAS | USA, California, Santa Barbara County, North of Lompoc, Harris Grade Road, 1.6 km N. of Burton Mesa Blvd., [34.693, -120.446], 24.V.1996, B. Harris |
| *Eulonchus smaragdinus* | 018937 | unknown | CAS | USA, California, Los Angeles County, Charmlee Park, Encinal Canyon Road, [34.059, -118.88], 3.VI.1993, R. Rogers |
| *Eulonchus smaragdinus* | 018938 | unknown | CAS | USA, California, Marin County, 14.5 km NE Point Reyes, Lighthouse, [38.084, -122.802], 28.V.1967, G. A. Gorelick |
| *Eulonchus smaragdinus* | 018939 | unknown | CAS | USA, California, Santa Barbara County, Tunnel Road Trail, [34.472, -119.705], 27.V.1984, hand netted, J.A. Calderwood |
| *Eulonchus smaragdinus* | 018940 | unknown | CAS | USA, California, Los Angeles County, Angeles National Forest, San Gabriel Mountains, Millard Canyon, [34.216, -118.146], 20.VI.1962, R. H. Crandall |
| *Eulonchus smaragdinus* | 018941 | unknown | CAS | USA, California, Los Angeles County, Angeles National Forest, San Gabriel Mountains, Millard Canyon, [34.216, -118.146], 20.VI.1962, R. H. Crandall |
| *Eulonchus smaragdinus* | 018942 | unknown | CAS | USA, California, Inyo County, Whitney Portal, [36.589, -118.227], 12.VI.1983, L. Muller |
| *Eulonchus smaragdinus* | 018943 | male | CAS | USA, California, Los Angeles County, Hollywood Hills, Wonderview Drive, [34.178, -118.259], 28.V.1972, R. Rogers |
| *Eulonchus smaragdinus* | 018944 | unknown | CAS | USA, California, Santa Barbara County, Camino Cielo Rd., [34.498, -119.242], 20.V.1989, R. Rogers |
| *Eulonchus smaragdinus* | 018982 | unknown | CAS | USA, California, Mendocino County, NCCRP (Northern California Coast Range Preserve), 8.1 km. N. of Branscomb, [39.77, -123.628], 24.V.1976 to 25.V.1976, R. Wharton |
| *Eulonchus smaragdinus* | 019023 | unknown | CAS | USA, California, Mendocino County, NCCRP (Northern California Coast Range Preserve), 8.1 km. N. of Branscomb, [39.77, -123.628], 24.V.1976 to 25.V.1975, R. Coville |
| *Eulonchus smaragdinus* | 019025 | unknown | CAS | USA, California, Mendocino County, NCCRP (Northern California Coast Range Preserve), 8.1 km. N. of Branscomb, [39.77, -123.628], 24.V.1976 to 25.V.1975, R. Coville |
| *Eulonchus smaragdinus* | 019027 | unknown | CAS | USA, California, Mendocino County, NCCRP (Northern California Coast Range Preserve), 8.1 km. N. of Branscomb, [39.77, -123.628], 24.V.1976 to 25.V.1975, R. Coville |
| *Eulonchus smaragdinus* | 019029 | unknown | CAS | USA, California, Mendocino County, NCCRP (Northern California Coast Range Preserve), 8.1 km. N. of Branscomb, [39.77, -123.628], 24.V.1976 to 25.V.1975, R. Coville |
| *Eulonchus smaragdinus* | 019045 | unknown | CAS | USA, California, San Mateo County, Belmont, 17.VI.1972, C. W. O’Brien |
| *Eulonchus smaragdinus* | 019050 | unknown | CAS | USA, California, Mendocino County, 1.6 km N Piercy, [39.981, -123.795], 20.V.1976 to 23.V.1976, flight trapJ. A. Chemsak |
| *Eulonchus smaragdinus* | 019074 | unknown | CAS | USA, California, Stanislaus County, Frank Raines Park, Del Puerto Canyon, [37.423, -121.375], 366 m, 24.V.1980, E. I. Schlinger |
| *Eulonchus smaragdinus* | 019088 | unknown | CAS | USA, California, Mendocino County, NCCRP (Northern California Coast Range Preserve), 4.8 km N of Branscomb, [39.737, -123.626], 30.V.1980 to 1.VI.1980, A. E. Hajek |
| *Eulonchus smaragdinus* | 019089 | unknown | CAS | USA, California, Mendocino County, NCCRP (Northern California Coast Range Preserve), 4.8 km N of Branscomb, [39.737, -123.626], 30.V.1980 to 1.VI.1980, A. E. Hajek |
| *Eulonchus smaragdinus* | 019090 | unknown | CAS | USA, California, Mendocino County, NCCRP (Northern California Coast Range Preserve), 4.8 km N of Branscomb, [39.737, -123.626], 30.V.1980 to 1.VI.1980, A. E. Hajek |
| *Eulonchus smaragdinus* | 019094 | unknown | CAS | USA, California, Mendocino County, NCCRP (Northern California Coast Range Preserve), 4.8 km N of Branscomb, [39.737, -123.626], 30.V.1980 to 1.VI.1980, hand netted, C. Parisek |
| *Eulonchus smaragdinus* | 019096 | unknown | CAS | USA, California, Contra Costa County, 16.VI.1973, G. Liu |
| *Eulonchus smaragdinus* | 019102 | unknown | CAS | USA, California, Mendocino County, NCCRP (Northern California Coast Range Preserve), 8.1 km. N. of Branscomb, [39.77, -123.628], 25.V.1976, J. A. Powell |
| *Eulonchus smaragdinus* | 019104 | unknown | CAS | USA, California, Mendocino County, NCCRP (Northern California Coast Range Preserve), 8.1 km. N. of Branscomb, [39.77, -123.628], 25.V.1976, J. A. Powell |
| *Eulonchus smaragdinus* | 019105 | unknown | CAS | USA, California, Mendocino County, NCCRP (Northern California Coast Range Preserve), 8.1 km. N. of Branscomb, [39.77, -123.628], 25.V.1976, J. A. Powell |
| *Eulonchus smaragdinus* | 019110 | unknown | CAS | USA, California, Mendocino County, NCCRP (Northern California Coast Range Preserve), 8.1 km. N. of Branscomb, [39.77, -123.628], 25.V.1976, J. A. Powell |
| *Eulonchus smaragdinus* | 019111 | unknown | CAS | USA, California, Mendocino County, NCCRP (Northern California Coast Range Preserve), 8.1 km. N. of Branscomb, [39.77, -123.628], 25.V.1976, J. A. Powell |
| *Eulonchus smaragdinus* | 019112 | unknown | CAS | USA, California, Mendocino County, NCCRP (Northern California Coast Range Preserve), 8.1 km. N. of Branscomb, [39.77, -123.628], 25.V.1976, J. A. Powell |
| *Eulonchus smaragdinus* | 019113 | unknown | CAS | USA, California, Mendocino County, NCCRP (Northern California Coast Range Preserve), 8.1 km. N. of Branscomb, [39.77, -123.628], 25.V.1976, J. A. Powell |
| *Eulonchus smaragdinus* | 019115 | unknown | CAS | USA, California, Mendocino County, NCCRP (Northern California Coast Range Preserve), 8.1 km. N. of Branscomb, [39.77, -123.628], 25.V.1976, J. A. Powell |
| *Eulonchus smaragdinus* | 019130 | unknown | CAS | USA, California, Mendocino County, NCCRP (Northern California Coast Range Preserve), 8.1 km. N. of Branscomb, [39.77, -123.628], 26.V.1976, J. A. Powell |
| *Eulonchus smaragdinus* | 019145 | unknown | CAS | USA, California, San Luis Obispo County, Reservoir Canyon, [35.281, -120.605], 2.VI.1968, R. Wharton |
| *Eulonchus smaragdinus* | 019151 | unknown | CAS | USA, California, Mendocino County, NCCRP (Northern California Coast Range Preserve), 8.1 km. N. of Branscomb, [39.77, -123.628], 24.V.1976 to 25.V.1975, R. Coville |
| *Eulonchus smaragdinus* | 019152 | unknown | CAS | USA, California, Mendocino County, NCCRP (Northern California Coast Range Preserve), 8.1 km. N. of Branscomb, [39.77, -123.628], 24.V.1976 to 25.V.1975, R. Coville |
| *Eulonchus smaragdinus* | 019154 | unknown | CAS | USA, California, Mendocino County, NCCRP (Northern California Coast Range Preserve), 8.1 km. N. of Branscomb, [39.77, -123.628], 24.V.1976 to 25.V.1975, R. Coville |
| *Eulonchus smaragdinus* | 019204 | unknown | CAS | USA, California, Mendocino County, NCCRP (Northern California Coast Range Preserve), 4.8 km N of Branscomb, [39.737, -123.626], 18.V.1984 to 21.V.1984, E. I. Schlinger |
| *Eulonchus smaragdinus* | 019411 | female | CAS | USA, California, Los Angeles County, 3.2 km S Tarzana, [34.148, -118.551], 6.VI.1968, hand netted, S.R. Kutcher |
| *Eulonchus smaragdinus* | 019413 | male | CAS | USA, California, Los Angeles County, 3.2 km S Tarzana, [34.148, -118.551], 30.V.1968, hand netted, S.R. Kutcher |
| *Eulonchus smaragdinus* | 019414 | male | CAS | USA, California, Los Angeles County, 3.2 km S Tarzana, [34.148, -118.551], 6.VI.1968, hand netted, S.R. Kutcher |
| *Eulonchus smaragdinus* | 019415 | male | CAS | USA, California, Ventura County, 12.9 km W Lake Sherwood, [34.14, -118.848], 7.VII.1969, S.R. Kutcher |
| *Eulonchus smaragdinus* | 019453 | unknown | CAS | USA, California, Monterey County, Marina Dunes, [36.698, -121.807], 22.IV.1970, E. I. Schlinger |
| *Eulonchus smaragdinus* | 019486 | unknown | CAS | USA, California, Marin County, Mill Valley, [37.906, -122.545], 110 m, 14.VI.1965 to 16.VI.1965, P. H. Arnaud, Jr. |
| *Eulonchus smaragdinus* | 019497 | unknown | CAS | USA, California, Marin County, Mill Valley, Blithedale Ridge, Lee Street, [37.921, -122.553], 110 m, 24.VI.1965 to 28.VI.1965, Malaise trap, P. H. Arnaud, Jr. |
| *Eulonchus smaragdinus* | 019498 | unknown | CAS | USA, California, Marin County, Mill Valley, Blithedale Ridge, Lee Street, [37.921, -122.553], 110 m, 24.VI.1965 to 28.VI.1965, Malaise trap, P. H. Arnaud, Jr. |
| *Eulonchus smaragdinus* | 019499 | unknown | CAS | USA, California, Marin County, Mill Valley, Blithedale Ridge, Lee Street, [37.921, -122.553], 110 m, 24.VI.1965 to 28.VI.1965, Malaise trap, P. H. Arnaud, Jr. |
| *Eulonchus smaragdinus* | 019500 | unknown | CAS | USA, California, Marin County, Mill Valley, Blithedale Ridge, Lee Street, [37.921, -122.553], 110 m, 24.VI.1965 to 28.VI.1965, Malaise trap, P. H. Arnaud, Jr. |
| *Eulonchus smaragdinus* | 019501 | unknown | CAS | USA, California, Marin County, Mill Valley, Blithedale Ridge, Lee Street, [37.921, -122.553], 110 m, 24.VI.1965 to 28.VI.1965, Malaise trap, P. H. Arnaud, Jr. |
| *Eulonchus smaragdinus* | 019502 | unknown | CAS | USA, California, Marin County, Mill Valley, Blithedale Ridge, Lee Street, [37.921, -122.553], 110 m, 24.VI.1965 to 28.VI.1965, Malaise trap, P. H. Arnaud, Jr. |
| *Eulonchus smaragdinus* | 019503 | unknown | CAS | USA, California, Marin County, Mill Valley, Blithedale Ridge, Lee Street, [37.921, -122.553], 110 m, 24.VI.1965 to 28.VI.1965, Malaise trap, P. H. Arnaud, Jr. |
| *Eulonchus smaragdinus* | 019504 | unknown | CAS | USA, California, Marin County, Mill Valley, Blithedale Ridge, Lee Street, [37.921, -122.553], 110 m, 24.VI.1965 to 28.VI.1965, Malaise trap, P. H. Arnaud, Jr. |
| *Eulonchus smaragdinus* | 019505 | unknown | CAS | USA, California, Marin County, Mill Valley, Blithedale Ridge, Lee Street, [37.921, -122.553], 110 m, 24.VI.1965 to 28.VI.1965, Malaise trap, P. H. Arnaud, Jr. |
| *Eulonchus smaragdinus* | 019506 | unknown | CAS | USA, California, Marin County, Mill Valley, Blithedale Ridge, Lee Street, [37.921, -122.553], 110 m, 24.VI.1965 to 28.VI.1965, Malaise trap, P. H. Arnaud, Jr. |
| *Eulonchus smaragdinus* | 019507 | unknown | CAS | USA, California, Marin County, Mill Valley, Blithedale Ridge, Lee Street, [37.921, -122.553], 110 m, 24.VI.1965 to 28.VI.1965, Malaise trap, P. H. Arnaud, Jr. |
| *Eulonchus smaragdinus* | 019508 | unknown | CAS | USA, California, Marin County, Mill Valley, Blithedale Ridge, Lee Street, [37.921, -122.553], 110 m, 24.VI.1965 to 28.VI.1965, Malaise trap, P. H. Arnaud, Jr. |
| *Eulonchus smaragdinus* | 019509 | unknown | CAS | USA, California, Marin County, Mill Valley, Blithedale Ridge, Lee Street, [37.921, -122.553], 110 m, 24.VI.1965 to 28.VI.1965, Malaise trap, P. H. Arnaud, Jr. |
| *Eulonchus smaragdinus* | 019510 | unknown | CAS | USA, California, Marin County, Mill Valley, Blithedale Ridge, Lee Street, [37.921, -122.553], 110 m, 24.VI.1965 to 28.VI.1965, Malaise trap, P. H. Arnaud, Jr. |
| *Eulonchus smaragdinus* | 019511 | unknown | CAS | USA, California, Marin County, Mill Valley, Blithedale Ridge, Lee Street, [37.921, -122.553], 110 m, 24.VI.1965 to 28.VI.1965, Malaise trap, P. H. Arnaud, Jr. |
| *Eulonchus smaragdinus* | 019512 | unknown | CAS | USA, California, Marin County, Mill Valley, Blithedale Ridge, Lee Street, [37.921, -122.553], 110 m, 24.VI.1965 to 28.VI.1965, Malaise trap, P. H. Arnaud, Jr. |
| *Eulonchus smaragdinus* | 019513 | unknown | CAS | USA, California, Marin County, Mill Valley, Blithedale Ridge, Lee Street, [37.921, -122.553], 110 m, 24.VI.1965 to 28.VI.1965, Malaise trap, P. H. Arnaud, Jr. |
| *Eulonchus smaragdinus* | 019514 | unknown | CAS | USA, California, Marin County, Mill Valley, Blithedale Ridge, Lee Street, [37.921, -122.553], 110 m, 24.VI.1965 to 28.VI.1965, Malaise trap, P. H. Arnaud, Jr. |
| *Eulonchus smaragdinus* | 019515 | unknown | CAS | USA, California, Marin County, Mill Valley, Blithedale Ridge, Lee Street, [37.921, -122.553], 110 m, 24.VI.1965 to 28.VI.1965, Malaise trap, P. H. Arnaud, Jr. |
| *Eulonchus smaragdinus* | 019516 | unknown | CAS | USA, California, Marin County, Mill Valley, Blithedale Ridge, Lee Street, [37.921, -122.553], 110 m, 24.VI.1965 to 28.VI.1965, Malaise trap, P. H. Arnaud, Jr. |
| *Eulonchus smaragdinus* | 019517 | unknown | CAS | USA, California, Marin County, Mill Valley, Blithedale Ridge, Lee Street, [37.921, -122.553], 110 m, 24.VI.1965 to 28.VI.1965, Malaise trap, P. H. Arnaud, Jr. |
| *Eulonchus smaragdinus* | 019518 | unknown | CAS | USA, California, Marin County, Mill Valley, Blithedale Ridge, Lee Street, [37.921, -122.553], 110 m, 24.VI.1965 to 28.VI.1965, Malaise trap, P. H. Arnaud, Jr. |
| *Eulonchus smaragdinus* | 019519 | unknown | CAS | USA, California, Marin County, Mill Valley, Blithedale Ridge, Lee Street, [37.921, -122.553], 110 m, 24.VI.1965 to 28.VI.1965, Malaise trap, P. H. Arnaud, Jr. |
| *Eulonchus smaragdinus* | 019520 | unknown | CAS | USA, California, Marin County, Mill Valley, Blithedale Ridge, Lee Street, [37.921, -122.553], 110 m, 15.V.1965 to 17.V.1965, P. H. Arnaud, Jr. |
| *Eulonchus smaragdinus* | 019521 | unknown | CAS | USA, California, Marin County, Mill Valley, Blithedale Ridge, Lee Street, [37.921, -122.553], 110 m, 20.VI.1965 to 23.VI.1965, P. H. Arnaud, Jr. |
| *Eulonchus smaragdinus* | 019522 | unknown | CAS | USA, California, Marin County, Mill Valley, Blithedale Ridge, Lee Street, [37.921, -122.553], 110 m, 20.VI.1965 to 23.VI.1965, P. H. Arnaud, Jr. |
| *Eulonchus smaragdinus* | 019523 | unknown | CAS | USA, California, Marin County, Mill Valley, Blithedale Ridge, Lee Street, [37.921, -122.553], 110 m, 20.VI.1965 to 23.VI.1965, P. H. Arnaud, Jr. |
| *Eulonchus smaragdinus* | 019524 | unknown | CAS | USA, California, Marin County, Mill Valley, Blithedale Ridge, Lee Street, [37.921, -122.553], 110 m, 20.VI.1965 to 23.VI.1965, P. H. Arnaud, Jr. |
| *Eulonchus smaragdinus* | 019525 | unknown | CAS | USA, California, Marin County, Mill Valley, Blithedale Ridge, Lee Street, [37.921, -122.553], 110 m, 9.V.1965 to 10.V.1965, P. H. Arnaud, Jr. |
| *Eulonchus smaragdinus* | 019526 | unknown | CAS | USA, California, Humboldt County, Laribee Valley, [40.442, -123.671], 30.VI.1935, B. P. Bliven |
| *Eulonchus smaragdinus* | 019537 | unknown | CAS | USA, California, Alameda County, Kilkare Road 1.4 mi. N of Sunol, 10.VII.1980, T. W. Davies |
| *Eulonchus smaragdinus* | 019546 | unknown | CAS | USA, California, Marin County, Mill Valley, [37.906, -122.545], 110 m, 29.VII.1965 to 1.VIII.1965, P. H. Arnaud, Jr. |
| *Eulonchus smaragdinus* | 019547 | unknown | CAS | USA, California, Marin County, Mill Valley, [37.906, -122.545], 110 m, 29.VII.1965 to 1.VIII.1965, P. H. Arnaud, Jr. |
| *Eulonchus smaragdinus* | 019548 | unknown | CAS | USA, California, Marin County, Mill Valley, [37.906, -122.545], 110 m, 29.VII.1965 to 1.VIII.1965, P. H. Arnaud, Jr. |
| *Eulonchus smaragdinus* | 019549 | unknown | CAS | USA, California, Marin County, Mill Valley, [37.906, -122.545], 110 m, 29.VII.1965 to 1.VIII.1965, P. H. Arnaud, Jr. |
| *Eulonchus smaragdinus* | 019550 | unknown | CAS | USA, California, Marin County, Mill Valley, [37.906, -122.545], 110 m, 29.VII.1965 to 1.VIII.1965, P. H. Arnaud, Jr. |
| *Eulonchus smaragdinus* | 019571 | unknown | CAS | USA, California, Marin County, Mill Valley, [37.906, -122.545], 110 m, 14.VI.1965 to 16.VI.1965, P. H. Arnaud, Jr. |
| *Eulonchus smaragdinus* | 019575 | unknown | CAS | USA, California, Marin County, Mill Valley, [37.906, -122.545], 110 m, 14.VI.1965 to 16.VI.1965, P. H. Arnaud, Jr. |
| *Eulonchus smaragdinus* | 019583 | unknown | CAS | USA, California, Marin County, Mill Valley, =, [37.906, -122.545], 16.VII.1965 to 18.VII.1965, P. H. Arnaud, Jr. |
| *Eulonchus smaragdinus* | 019584 | unknown | CAS | USA, California, Marin County, Mill Valley, =, [37.906, -122.545], 16.VII.1965 to 18.VII.1965, P. H. Arnaud, Jr. |
| *Eulonchus smaragdinus* | 019585 | unknown | CAS | USA, California, Marin County, Mill Valley, =, [37.906, -122.545], 16.VII.1965 to 18.VII.1965, P. H. Arnaud, Jr. |
| *Eulonchus smaragdinus* | 019586 | unknown | CAS | USA, California, Marin County, Mill Valley, =, [37.906, -122.545], 16.VII.1965 to 18.VII.1965, P. H. Arnaud, Jr. |
| *Eulonchus smaragdinus* | 019587 | unknown | CAS | USA, California, Marin County, Mill Valley, =, [37.906, -122.545], 16.VII.1965 to 18.VII.1965, P. H. Arnaud, Jr. |
| *Eulonchus smaragdinus* | 019588 | unknown | CAS | USA, California, Marin County, Mill Valley, =, [37.906, -122.545], 16.VII.1965 to 18.VII.1965, P. H. Arnaud, Jr. |
| *Eulonchus smaragdinus* | 019598 | unknown | CAS | USA, California, Mendocino County, NCCRP (Northern California Coast Range Preserve), 8.1 km. N. of Branscomb, [39.77, -123.628], 25.V.1976, J. A. Powell |
| *Eulonchus smaragdinus* | 019819 | unknown | CAS | USA, California, Marin County, Point Reyes National Seashore, North Beach, [38.044, -122.791], 30 m, 14.VI.1975, E. I. Schlinger |
| *Eulonchus smaragdinus* | 020000 | unknown | USNM | USA, California, Monterey, Pacific Grove, [36.618, -121.918], 20.V.1920, A. H. Sturtevant |
| *Eulonchus smaragdinus* | 020001 | unknown | USNM | USA, California, Monterey, Pacific Grove, [36.618, -121.918], .V.1920 to .VIII.1920 |
| *Eulonchus smaragdinus* | 020002 | unknown | USNM | USA, California, Monterey, Pacific Grove, [36.618, -121.918], 20.V.1920 to 24.V.1920 |
| *Eulonchus smaragdinus* | 020002 | unknown | USNM | USA, California, Monterey, Pacific Grove, [36.618, -121.918], 20.V.1920 to 24.V.1920 |
| *Eulonchus smaragdinus* | 020003 | unknown | USNM | USA, California, Monterey, Pacific Grove, [36.618, -121.918], 24.V.1920 |
| *Eulonchus smaragdinus* | 020004 | unknown | USNM | USA, California, Marin County, Mill Valley, [37.906, -122.55], 17.VI.1965 to 19.VI.1965, flight trap |
| *Eulonchus smaragdinus* | 020005 | unknown | USNM | USA, California, Marin County, Mill Valley, [37.906, -122.55], 17.VI.1965 to 19.VI.1965, flight trap |
| *Eulonchus smaragdinus* | 020006 | unknown | USNM | USA, California, Marin County, Mill Valley, [37.906, -122.55], 17.VI.1965 to 19.VI.1965, flight trap |
| *Eulonchus smaragdinus* | 020007 | unknown | USNM | USA, California, Marin County, Mill Valley, [37.906, -122.55], 17.VI.1965 to 19.VI.1965, flight trap |
| *Eulonchus smaragdinus* | 020008 | unknown | USNM | USA, California, Doran Beach Park, 28.V.1958 |
| *Eulonchus smaragdinus* | 020009 | unknown | USNM | USA, California, Doran Beach Park, 28.V.1958 |
| *Eulonchus smaragdinus* | 020010 | unknown | USNM | USA, California, Doran Beach Park, 28.V.1958 |
| *Eulonchus smaragdinus* | 020011 | unknown | USNM | USA, California, Marin County, Mill Valley, [37.906, -122.55], 17.VI.1965 to 19.VI.1965, flight trap |
| *Eulonchus smaragdinus* | 020012 | unknown | USNM | USA, California, Marin County, Mill Valley, [37.906, -122.55], 17.VI.1965 to 19.VI.1965, flight trap |
| *Eulonchus smaragdinus* | 020013 | unknown | USNM | USA, California, Monterey, Pacific Grove, [36.618, -121.918], 2.VI.1920 |
| *Eulonchus smaragdinus* | 020014 | unknown | CAS | USA, California, Marin County, Mill Valley, [37.906, -122.55], 17.VI.1965 to 19.VI.1965, flight trap |
| *Eulonchus smaragdinus* | 020015 | unknown | CAS | USA, California, Marin County, Mill Valley, [37.906, -122.55], 17.VI.1965 to 19.VI.1965, flight trap |
| *Eulonchus smaragdinus* | 020016 | unknown | CAS | USA, California, Marin County, Mill Valley, [37.906, -122.55], 17.VI.1965 to 19.VI.1965, flight trap |
| *Eulonchus smaragdinus* | 020017 | unknown | CAS | USA, California, Marin County, Mill Valley, [37.906, -122.55], 17.VI.1965 to 19.VI.1965, flight trap |
| *Eulonchus smaragdinus* | 020083 | unknown | CAS | USA, California, Humboldt County, On Shruberry, [40.276, -123.636], 20.VII.1978 |
| *Eulonchus smaragdinus* | 020084 | unknown | CAS | USA, California, Marin County, Point Reyes National Seashore, North Beach, [38.076, -122.977], 12.VI.1974 |
| *Eulonchus smaragdinus* | 020085 | unknown | CAS | USA, California, Riverside County, San Bernardino National Forest, Thomas Mountian, [33.619, -116.681] |
| *Eulonchus smaragdinus* | 020087 | unknown | CAS | USA, California, Napa County, 12.9 Southwest of Monticello Dam, [38.505, -122.115], 27.V.1978 |
| *Eulonchus smaragdinus* | 020167 | unknown | CAS | USA, California, Riverside County, San Bernardino National Forest, San Jacinto Mountains, Thomas Mountain, [33.62, -116.68] |
| *Eulonchus smaragdinus* | 020168 | unknown | CAS | USA, California, Riverside County, San Bernardino National Forest, San Jacinto Mountains, Thomas Mountain, [33.62, -116.68] |
| *Eulonchus smaragdinus* | 020176 | unknown | CAS | USA, California, Los Angeles County, Tanbark Flat, [34.204, -117.761], 18.VI.1956 |
| *Eulonchus smaragdinus* | 020177 | unknown | CAS | USA, California, Los Angeles County, Tanbark Flat, [34.204, -117.761], 18.VI.1956 |
| *Eulonchus smaragdinus* | 020178 | unknown | CAS | USA, California, Los Angeles County, Tanbark Flat, [34.204, -117.761], 18.VI.1956 |
| *Eulonchus smaragdinus* | 020179 | unknown | CAS | USA, California, Napa County, 12.9 Southwest of Monticello Dam, [38.505, -122.115], 27.V.1978 |
| *Eulonchus smaragdinus* | 020180 | unknown | CAS | USA, California, Napa County, 12.9 Southwest of Monticello Dam, [38.505, -122.115], 31.V.1978 |
| *Eulonchus smaragdinus* | 020181 | unknown | CAS | USA, California, Napa County, 12.9 Southwest of Monticello Dam, [38.505, -122.115], 3.VI.1978 |
| *Eulonchus smaragdinus* | 020182 | unknown | CAS | USA, California, Monterey County, Big Sur, [36.271, -121.808], 11.VI.1981 |
| *Eulonchus smaragdinus* | 020183 | unknown | CAS | USA, California, Claremont, [34.095, -117.72] |
| *Eulonchus smaragdinus* | 020187 | unknown | CAS | USA, California, Monterey County, 3 km East of Marina, Coastal Sand Dunes, [36.717, -121.811], 30 m, 6.IV.2011, hand netted, |
| *Eulonchus smaragdinus* | 020221 | male | CAS | USA, California, Marin County, Mill Valley, [37.906, -122.545], 110 m, 13.VII.1965 to 15.VII.1965, P. H. Arnaud, Jr. |
| *Eulonchus smaragdinus* | 020223 | unknown | CAS | USA, California, Marin County, Mill Valley, Blithedale Ridge, Lee Street, [37.921, -122.553], 110 m, 24.VI.1965 to 28.VI.1965, Malaise trap, P. H. Arnaud, Jr. |
| *Eulonchus smaragdinus* | 020228 | male | CAS | USA, California, Mariposa County, Yosemite National Park, Illilouette Basin, [37.687, -119.547], 1940 m, 14.VI.2014 |
| *Eulonchus tristis* | 000299 | male | CAS | USA, California, Mount Diablo, [37.905, -121.949], 29.V.1951, W. J. Wall |
| *Eulonchus tristis* | 000381 | male | CAS | USA, California, Napa County, Samuel Springs, [38.604, -122.311], 7.VI.1953, R. C. Bechtel |
| *Eulonchus tristis* | 000382 | female | CAS | USA, California, Napa County, Samuel Springs, [38.604, -122.311], 7.VI.1953, R. C. Bechtel |
| *Eulonchus tristis* | 000387 | male | CAS | USA, California, Fairfax, [37.987, -122.589], 21.V.1950, W. F. Ehrhardt |
| *Eulonchus tristis* | 000388 | male | CAS | USA, California, Santa Cruz County, Santa Cruz Mountains, [37.091, -121.844], 15.I.1922, E. O. Essig |
| *Eulonchus tristis* | 000389 | male | CAS | USA, California, Humboldt County, Blocksburg, [40.276, -123.636], 19.VI.1935, E. O. Essig |
| *Eulonchus tristis* | 000390 | male | CAS | USA, California, San Mateo County, Portola State Park, [37.253, -122.212], 7.V.1950, P. D. Hurd |
| *Eulonchus tristis* | 000391 | male | CAS | USA, California, Santa Clara County, Madera Creek, [37.942, -122.502], 22.IV.1921 |
| *Eulonchus tristis* | 000392 | male | CAS | USA, California, Berkeley, [37.871, -122.273], 16.VI.1932 |
| *Eulonchus tristis* | 000393 | male | CAS | USA, California, Siskiyou County, Lake Mountain, [41.749, -123.133], 10.VI.1951, R. P. Allen |
| *Eulonchus tristis* | 000394 | male | CAS | USA, California, Solano County, Green Valley, [38.252, -122.163], 16.VI.1953, A. A. Grigarick |
| *Eulonchus tristis* | 000395 | male | CAS | USA, California, Solano County, Green Valley, [38.252, -122.163], 16.VI.1953, R. C. Bechtel |
| *Eulonchus tristis* | 000396 | male | CAS | USA, California, Santa Cruz County, 11.3 km northwest Santa Cruz, Empire Grade Road, [37.038, -122.129], 23.V.1965, H. B. Leech |
| *Eulonchus tristis* | 000397 | male | CAS | USA, California, Santa Cruz County, 11.3 km northwest Santa Cruz, Empire Grade Road, [37.038, -122.129], 23.V.1965, H. B. Leech |
| *Eulonchus tristis* | 000398 | male | CAS | USA, California, Lake County, Hopland Grade, [38.997, -122.949], 7.VI.1960, S. M. Fidel |
| *Eulonchus tristis* | 000399 | male | CAS | USA, California, Marin County, Phoenix Lake, [37.955, -122.577], 55 m, 12.IV.1958, D. C. Rentz |
| *Eulonchus tristis* | 000400 | male | CAS | USA, California, Lake County, Hopland Grade, [38.997, -122.949], 7.VI.1960, S. M. Fidel |
| *Eulonchus tristis* | 000401 | male | CAS | USA, California, Lake County, Hopland Grade, [38.997, -122.949], 7.VI.1960, S. M. Fidel |
| *Eulonchus tristis* | 000402 | male | CAS | USA, California, Lake County, Hopland Grade, [38.997, -122.949], 7.VI.1960, S. M. Fidel |
| *Eulonchus tristis* | 000403 | male | CAS | USA, California, Lake County, Blue Lakes, [39.175, -123.015], 1.VI.1960, S. M. Fidel |
| *Eulonchus tristis* | 000404 | female | CAS | USA, California, Lake County, Blue Lakes, [39.175, -123.015], 1.VI.1960, S. M. Fidel |
| *Eulonchus tristis* | 000405 | male | CAS | USA, California, Lake County, Hopland Grade, [38.997, -122.949], 10.VI.1959, S. M. Fidel |
| *Eulonchus tristis* | 000406 | male | CAS | USA, California, Lake County, Hopland Grade, [38.997, -122.949], 17.VI.1959, S. M. Fidel |
| *Eulonchus tristis* | 000408 | male | CAS | USA, California, Lake County, Hopland Grade, [38.997, -122.949], 7.VI.1960, S. M. Fidel |
| *Eulonchus tristis* | 000409 | male | CAS | USA, California, Santa Cruz County, Santa Cruz Mountains, [37.091, -121.844], 20.VI.1956, D. Ribble |
| *Eulonchus tristis* | 000410 | male | CAS | USA, California, Lake County, Hopland Grade, [38.997, -122.949], 10.VI.1959, S. M. Fidel |
| *Eulonchus tristis* | 000411 | male | CAS | USA, California, Lake County, Hopland Grade, [38.997, -122.949], 10.VI.1959, S. M. Fidel |
| *Eulonchus tristis* | 000412 | male | CAS | USA, California, Lake County, Hopland Grade, [38.997, -122.949], 10.VI.1959, S. M. Fidel |
| *Eulonchus tristis* | 000413 | male | CAS | USA, California, Lake County, Hopland Grade, [38.997, -122.949], 17.VI.1959, S. M. Fidel |
| *Eulonchus tristis* | 000414 | male | CAS | USA, California, Lake County, Hopland Grade, [38.997, -122.949], 17.VI.1959, S. M. Fidel |
| *Eulonchus tristis* | 000415 | male | CAS | USA, California, Lake County, Hopland Grade, [38.997, -122.949], 17.VI.1959, S. M. Fidel |
| *Eulonchus tristis* | 000416 | male | CAS | USA, California, Lake County, Hopland Grade, [38.997, -122.949], 10.VI.1959, S. M. Fidel |
| *Eulonchus tristis* | 000417 | male | CAS | USA, California, Lake County, Hopland Grade, [38.997, -122.949], 17.VI.1959, S. M. Fidel |
| *Eulonchus tristis* | 000420 | male | CAS | USA, California, Lake County, Hopland Grade, [38.997, -122.949], 16.VI.1958, S. M. Fidel |
| *Eulonchus tristis* | 000421 | male | CAS | USA, California, Lake County, Hopland Grade, [38.997, -122.949], 10.VI.1959, S. M. Fidel |
| *Eulonchus tristis* | 000422 | male | CAS | USA, California, Lake County, Hopland Grade, [38.997, -122.949], 10.VI.1959, S. M. Fidel |
| *Eulonchus tristis* | 000423 | male | CAS | USA, California, Lake County, Kelseyville, [38.978, -122.84], 4.VI.1958, S. M. Fidel |
| *Eulonchus tristis* | 000425 | male | CAS | USA, California, Lake County, Hopland Grade, [38.997, -122.949], 17.VI.1959, S. M. Fidel |
| *Eulonchus tristis* | 000426 | male | CAS | USA, California, Lake County, Blue Lakes, [39.175, -123.015], 20.V.1959, S. M. Fidel |
| *Eulonchus tristis* | 000427 | male | CAS | USA, California, Lake County, Hopland Grade, [38.997, -122.949], 10.VI.1959, S. M. Fidel |
| *Eulonchus tristis* | 000428 | male | CAS | USA, California, Lake County, Hopland Grade, [38.997, -122.949], 28.V.1958, S. M. Fidel |
| *Eulonchus tristis* | 000429 | male | CAS | USA, California, Lake County, Kelseyville, [38.978, -122.84], 4.VI.1958, S. M. Fidel |
| *Eulonchus tristis* | 000430 | male | CAS | USA, California, Lake County, Hopland Grade, [38.997, -122.949], 16.VI.1958, S. M. Fidel |
| *Eulonchus tristis* | 000431 | male | CAS | USA, California, Lake County, Hopland Grade, [38.997, -122.949], 16.VI.1958, S. M. Fidel |
| *Eulonchus tristis* | 000432 | male | CAS | USA, California, Lake County, Kelseyville, [38.978, -122.84], 4.VI.1958, S. M. Fidel |
| *Eulonchus tristis* | 000433 | male | CAS | USA, California, Yolo County, Davis, [38.545, -121.741], 30.VI.1947 |
| *Eulonchus tristis* | 000434 | female | CAS | USA, California, Lake County, Kelseyville, [38.978, -122.84], 4.VI.1958, S. M. Fidel |
| *Eulonchus tristis* | 000435 | female | CAS | USA, California, San Mateo County, Portola State Park, [37.253, -122.212], 7.V.1950, P. D. Hurd |
| *Eulonchus tristis* | 000436 | female | CAS | USA, California, Lake County, Blue Lakes, [39.175, -123.015], 20.V.1959, S. M. Fidel |
| *Eulonchus tristis* | 000437 | female | CAS | USA, California, Solano County, Green Valley, [38.252, -122.163], 16.VI.1953, R. C. Bechtel |
| *Eulonchus tristis* | 000438 | female | CAS | USA, California, Siskiyou County, Lake Mountain, [41.749, -123.133], 10.VI.1951, R. P. Allen |
| *Eulonchus tristis* | 000439 | female | CAS | USA, California, Lake County, Hopland Grade, [38.997, -122.949], 17.VI.1959, S. M. Fidel |
| *Eulonchus tristis* | 000440 | female | CAS | USA, California, Lake County, Hopland Grade, [38.997, -122.949], 17.VI.1959, S. M. Fidel |
| *Eulonchus tristis* | 000441 | female | CAS | USA, California, Solano County, Green Valley, [38.252, -122.163], 16.VI.1953, A. A. Grigarick |
| *Eulonchus tristis* | 000442 | female | CAS | USA, California, Mount Diablo, [37.905, -121.949], 30.V.1951, E. I. Schlinger |
| *Eulonchus tristis* | 000443 | female | CAS | USA, California, Berkeley, [37.871, -122.273], 17.V.1958, D. L. Dahlsten |
| *Eulonchus tristis* | 000444 | female | CAS | USA, California, Marin County, Tomales Bay State Park, [38.125, -122.903], 14.IV.1961, D. Q. Cavagnaro |
| *Eulonchus tristis* | 000445 | female | CAS | USA, California, Lake County, Hopland Grade, [38.997, -122.949], 7.VI.1960, S. M. Fidel |
| *Eulonchus tristis* | 000446 | female | CAS | USA, California, Santa Cruz County, 11.3 km northwest Santa Cruz, Empire Grade Road, [37.038, -122.129], 23.V.1965, H. B. Leech |
| *Eulonchus tristis* | 000447 | female | CAS | USA, California, Solano County, Green Valley, [38.252, -122.163], 16.VI.1953, R. C. Bechtel |
| *Eulonchus tristis* | 000448 | female | CAS | USA, California, Solano County, Green Valley, [38.252, -122.163], 19.VI.1953, R. C. Bechtel |
| *Eulonchus tristis* | 000449 | female | CAS | USA, California, Solano County, Green Valley, [38.252, -122.163], 16.VI.1953, R. C. Bechtel |
| *Eulonchus tristis* | 000450 | female | CAS | USA, California, Solano County, Green Valley, [38.252, -122.163], 19.VI.1953, R. C. Bechtel |
| *Eulonchus tristis* | 000451 | female | CAS | USA, California, Fairfax, [37.987, -122.589], 1.III.1921 |
| *Eulonchus tristis* | 000452 | male | CAS | USA, California, Siskiyou County, Siskiyou Wilderness, Clear Creek Trail, 12.VI.1996, J. Cromwell |
| *Eulonchus tristis* | 000453 | male | CAS | USA, California, Siskiyou County, Devil’s Punch Bowl, [41.804, -123.672], 24.VII.1995, J. Cromwell |
| *Eulonchus tristis* | 000454 | male | CAS | USA, California, Siskiyou County, Devil’s Punch Bowl, 1524 m, 21.VI.1994, J. Cromwell |
| *Eulonchus tristis* | 000455 | female | CAS | USA, California, Siskiyou County, Devil’s Punch Bowl, [41.804, -123.672], 8.VII.1995, J. Cromwell |
| *Eulonchus tristis* | 000456 | male | CAS | USA, Oregon, Curry County, Kalmiopsis Wilderness, Babyfoot Lake, [42.283, -123.963], 7.VI.1996, J. Cromwell |
| *Eulonchus tristis* | 000498 | male | CAS | USA, California, Tulare County, 5.6 km west California Hot Springs, [35.879, -118.736], 22.VI.1963, R. W. Thorp |
| *Eulonchus tristis* | 004129 | unknown | CAS | USA, California, Tuolumne County, north of Cherry Creek, [37.902, -119.967], 28.V.1996, T. Griswold |
| *Eulonchus tristis* | 004131 | unknown | CAS | USA, California, Siskiyou County, south side Red Butte, [14.92, -123.185], 1798 m, 5.VI.1992, T. Griswold |
| *Eulonchus tristis* | 004139 | unknown | CAS | USA, California, Fresno County, south of Chawanakee, [37.504, -119.325], 26.V.1996, T. Griswold |
| *Eulonchus tristis* | 004140 | unknown | CAS | USA, California, Fresno County, south of Chawanakee, [37.504, -119.325], 26.V.1996, T. Griswold |
| *Eulonchus tristis* | 004141 | unknown | CAS | USA, California, Tuolumne County, north of Cherry Creek, [37.902, -119.967], 28.V.1996, T. Griswold |
| *Eulonchus tristis* | 004142 | unknown | CAS | USA, California, Tuolumne County, north of Cherry Creek, [37.902, -119.967], 28.V.1996, T. Griswold |
| *Eulonchus tristis* | 006863 | male | CAS | USA, California, Marin County, Mill Valley, [37.906, -122.545], 110 m, 9.VII.1965 to 12.VII.1965, P. H. Arnaud, Jr. |
| *Eulonchus tristis* | 006864 | male | CAS | USA, California, Marin County, Mill Valley, [37.906, -122.545], 110 m, 9.VII.1965 to 12.VII.1965, P. H. Arnaud, Jr. |
| *Eulonchus tristis* | 006865 | female | CAS | USA, California, Marin County, Highway 1, 2.7 km northeast Muir Beach, [38.069, -122.804], 25.IV.1964, P. H. Arnaud, Jr. |
| *Eulonchus tristis* | 006866 | male | CAS | USA, California, Marin County, Mill Valley, =, [37.906, -122.545], 2.V.1965 to 4.V.1965, P. H. Arnaud, Jr. |
| *Eulonchus tristis* | 006867 | male | CAS | USA, California, Marin County, Mill Valley, =, [37.906, -122.545], 20.IV.1965, P. H. Arnaud, Jr. |
| *Eulonchus tristis* | 006868 | female | CAS | USA, California, Marin County, Mill Valley, =, [37.906, -122.545], 27.VI.1965, P. H. Arnaud, Jr. |
| *Eulonchus tristis* | 006869 | male | CAS | USA, California, Marin County, Mill Valley, [37.906, -122.545], 110 m, 13.VII.1965 to 15.VII.1965, P. H. Arnaud, Jr. |
| *Eulonchus tristis* | 006870 | male | CAS | USA, California, Marin County, Highway 1, 2.7 km northeast Muir Beach, [38.069, -122.804], 25.IV.1964, P. H. Arnaud, Jr. |
| *Eulonchus tristis* | 006871 | male | CAS | USA, California, Marin County, Mill Valley, [37.906, -122.545], 110 m, 22.V.1965 to 24.V.1965, flight trapP. H. Arnaud, Jr. |
| *Eulonchus tristis* | 006872 | male | CAS | USA, California, Marin County, Mount Tamalpais, [37.924, -122.596], 15.V.1968 |
| *Eulonchus tristis* | 006873 | male | CAS | USA, California, Los Gatos, [37.236, -121.963], 1.VII.1954, H. Munsterman |
| *Eulonchus tristis* | 006874 | male | CAS | USA, California, Mendocino County, 7.V.1928, E. R. Leach |
| *Eulonchus tristis* | 006875 | male | CAS | USA, California, Santa Cruz County, 11.3 km northwest Santa Cruz, Empire Grade Road, [37.038, -122.129], 23.V.1965, H. B. Leech |
| *Eulonchus tristis* | 006876 | male | CAS | USA, California, Santa Cruz County, 11.3 km northwest Santa Cruz, Empire Grade Road, [37.038, -122.129], 23.V.1965, H. B. Leech |
| *Eulonchus tristis* | 006877 | male | CAS | USA, California, Santa Cruz County, 11.3 km northwest Santa Cruz, Empire Grade Road, [37.038, -122.129], 23.V.1965, H. B. Leech |
| *Eulonchus tristis* | 006878 | male | CAS | USA, California, Santa Cruz County, 11.3 km northwest Santa Cruz, Empire Grade Road, [37.038, -122.129], 23.V.1965, H. B. Leech |
| *Eulonchus tristis* | 006879 | male | CAS | USA, California, Santa Cruz County, 11.3 km northwest Santa Cruz, Empire Grade Road, [37.038, -122.129], 23.V.1965, H. B. Leech |
| *Eulonchus tristis* | 006880 | female | CAS | USA, California, Alameda County, Oakland, Golf Links Road, [37.756, -122.146], time of day: 1140-1210 hours, 15.VI.1969, P. H. Arnaud, Jr. |
| *Eulonchus tristis* | 006881 | unknown | CAS | USA, California, Calaveras County, Calaveras Big Trees State Park, [38.275, -120.31], 19.VI.1990, B. Quelvog |
| *Eulonchus tristis* | 006882 | unknown | CAS | USA, California, Calaveras County, Calaveras Big Trees State Park, [38.275, -120.31], 19.VI.1990 to 27.VI.1990, B. Quelvog |
| *Eulonchus tristis* | 006883 | unknown | CAS | USA, California, Calaveras County, Calaveras Big Trees State Park, [38.275, -120.31], 19.VI.1990, B. Quelvog |
| *Eulonchus tristis* | 006884 | unknown | CAS | USA, California, Calaveras County, Calaveras Big Trees State Park, [38.275, -120.31], 19.VI.1990, B. Quelvog |
| *Eulonchus tristis* | 006885 | unknown | CAS | USA, California, Calaveras County, Calaveras Big Trees State Park, [38.275, -120.31], 19.VI.1990, B. Quelvog |
| *Eulonchus tristis* | 006886 | unknown | CAS | USA, California, Calaveras County, Calaveras Big Trees State Park, [38.275, -120.31], 19.VI.1990, B. Quelvog |
| *Eulonchus tristis* | 006887 | unknown | CAS | USA, California, Calaveras County, Calaveras Big Trees State Park, [38.275, -120.31], 19.VI.1990, B. Quelvog |
| *Eulonchus tristis* | 006888 | unknown | CAS | USA, California, Calaveras County, Calaveras Big Trees State Park, [38.275, -120.31], 19.VI.1990 to 27.VI.1990, B. Quelvog |
| *Eulonchus tristis* | 008718 | unknown | CAS | USA, Oregon, Linn County, Monument Peak, [44.695, -122.322], 1372 m, 17.VII.1969, K.J. Goeden |
| *Eulonchus tristis* | 008719 | unknown | CAS | USA, Oregon, Linn County, Monument Peak, [44.695, -122.322], 1372 m, 17.VII.1969, K.J. Goeden |
| *Eulonchus tristis* | 008720 | unknown | CAS | USA, Oregon, Linn County, Monument Peak, [44.695, -122.322], 1372 m, 17.VII.1969, K.J. Goeden |
| *Eulonchus tristis* | 008721 | unknown | CAS | USA, Oregon, Linn County, Monument Peak, [44.695, -122.322], 1372 m, 17.VII.1969, K.J. Goeden |
| *Eulonchus tristis* | 008722 | unknown | CAS | USA, Oregon, Linn County, Monument Peak, [44.695, -122.322], 1372 m, 17.VII.1969, K.J. Goeden |
| *Eulonchus tristis* | 008723 | unknown | CAS | USA, Oregon, Linn County, Monument Peak, [44.695, -122.322], 1372 m, 17.VII.1969, K.J. Goeden |
| *Eulonchus tristis* | 008724 | unknown | CAS | USA, Oregon, Linn County, Monument Peak, [44.695, -122.322], 1372 m, 17.VII.1969, K.J. Goeden |
| *Eulonchus tristis* | 008725 | unknown | CAS | USA, Oregon, Linn County, Monument Peak, [44.695, -122.322], 1372 m, 17.VII.1969, K.J. Goeden |
| *Eulonchus tristis* | 008726 | unknown | CAS | USA, Oregon, Linn County, Monument Peak, [44.695, -122.322], 1372 m, 17.VII.1969, K.J. Goeden |
| *Eulonchus tristis* | 008727 | unknown | CAS | USA, Oregon, Linn County, Monument Peak, [44.695, -122.322], 1372 m, 17.VII.1969, K.J. Goeden |
| *Eulonchus tristis* | 008728 | unknown | CAS | USA, Oregon, Linn County, Monument Peak, [44.695, -122.322], 1372 m, 17.VII.1969, K.J. Goeden |
| *Eulonchus tristis* | 008729 | unknown | CAS | USA, Oregon, Linn County, Monument Peak, [44.695, -122.322], 1372 m, 17.VII.1969, K.J. Goeden |
| *Eulonchus tristis* | 008730 | unknown | CAS | USA, Oregon, Linn County, Santiam Pass, Hwy 20, [44.403, -121.852], 21.VII.1969, K. Goeden |
| *Eulonchus tristis* | 008731 | unknown | CAS | USA, Oregon, Linn County, 9.7 km. SE. of Gates, [44.698, -122.326], 3.VII.1971, R. L. Westcott |
| *Eulonchus tristis* | 008732 | unknown | CAS | USA, Oregon, Linn County, 9.7 km. SE. of Gates, [44.698, -122.326], 3.VII.1971, R. L. Westcott |
| *Eulonchus tristis* | 008733 | unknown | CAS | USA, Oregon, Linn County, 9.7 km. SE. of Gates, [44.698, -122.326], 3.VII.1971, R. L. Westcott |
| *Eulonchus tristis* | 008735 | unknown | CAS | USA, Oregon, Linn County, 9.7 km. SE. of Gates, [44.698, -122.326], 3.VII.1971, R. L. Westcott |
| *Eulonchus tristis* | 008736 | unknown | CAS | USA, Oregon, Linn County, 9.7 km. SE. of Gates, [44.698, -122.326], 3.VII.1971, R. L. Westcott |
| *Eulonchus tristis* | 008737 | unknown | CAS | USA, Oregon, Linn County, 9.7 km. SE. of Gates, [44.698, -122.326], 3.VII.1971, R. L. Westcott |
| *Eulonchus tristis* | 008738 | unknown | CAS | USA, Oregon, Linn County, 9.7 km. SE. of Gates, [44.698, -122.326], 3.VII.1971, R. L. Westcott |
| *Eulonchus tristis* | 008739 | unknown | CAS | USA, Oregon, Linn County, 9.7 km. SE. of Gates, [44.698, -122.326], 3.VII.1971, R. L. Westcott |
| *Eulonchus tristis* | 008740 | unknown | CAS | USA, Oregon, Linn County, 9.7 km. SE. of Gates, [44.698, -122.326], 3.VII.1971, R. L. Westcott |
| *Eulonchus tristis* | 008741 | unknown | CAS | USA, Oregon, Linn County, 9.7 km. SE. of Gates, [44.698, -122.326], 3.VII.1971, R. L. Westcott |
| *Eulonchus tristis* | 008742 | unknown | CAS | USA, Oregon, Linn County, 7.7 km. W. of Marion Forks, [44.615, -122.044], 20.VI.1972, R. L. Penrose |
| *Eulonchus tristis* | 008743 | unknown | CAS | USA, Oregon, Linn County, 7.7 km. W. of Marion Forks, [44.615, -122.044], 20.VI.1972, R. L. Penrose |
| *Eulonchus tristis* | 008744 | unknown | CAS | USA, Oregon, Linn County, 7.7 km. W. of Marion Forks, [44.615, -122.044], 20.VI.1972, R. L. Penrose |
| *Eulonchus tristis* | 008745 | unknown | CAS | USA, Oregon, Linn County, 7.7 km. W. of Marion Forks, [44.615, -122.044], 20.VI.1972, R. L. Penrose |
| *Eulonchus tristis* | 008746 | unknown | CAS | USA, Oregon, Linn County, 7.7 km. W. of Marion Forks, [44.615, -122.044], 20.VI.1972, R. L. Penrose |
| *Eulonchus tristis* | 008747 | unknown | CAS | USA, Oregon, Linn County, 7.7 km. W. of Marion Forks, [44.615, -122.044], 20.VI.1972, R. L. Penrose |
| *Eulonchus tristis* | 008748 | unknown | CAS | USA, Oregon, Linn County, 7.7 km. W. of Marion Forks, [44.615, -122.044], 20.VI.1972, R. L. Penrose |
| *Eulonchus tristis* | 008749 | unknown | CAS | USA, Oregon, Linn County, 7.7 km. W. of Marion Forks, [44.615, -122.044], 20.VI.1972, R. L. Penrose |
| *Eulonchus tristis* | 008750 | unknown | CAS | USA, Oregon, Linn County, 7.7 km. W. of Marion Forks, [44.615, -122.044], 20.VI.1972, R. L. Penrose |
| *Eulonchus tristis* | 008751 | unknown | CAS | USA, Oregon, Linn County, 7.7 km. W. of Marion Forks, [44.615, -122.044], 20.VI.1972, R. L. Penrose |
| *Eulonchus tristis* | 008752 | unknown | CAS | USA, Oregon, Linn County, 7.7 km. W. of Marion Forks, [44.615, -122.044], 20.VI.1972, R. L. Penrose |
| *Eulonchus tristis* | 008754 | unknown | CAS | USA, Oregon, Linn County, 7.7 km. W. of Marion Forks, [44.615, -122.044], 20.VI.1972, R. L. Penrose |
| *Eulonchus tristis* | 008756 | unknown | CAS | USA, Oregon, Linn County, 7.7 km. W. of Marion Forks, [44.615, -122.044], 20.VI.1972, R. L. Penrose |
| *Eulonchus tristis* | 008759 | unknown | CAS | USA, Idaho, Latah County, S. slope of Moscow Mountain, [46.793, -116.87], 6.VII.1968, R. L. Westcott |
| *Eulonchus tristis* | 008762 | unknown | CAS | USA, Oregon, Baker County, 19.6 km NW of Haines, [45.024, -118.134], 16.VII.1975, R. L. Penrose |
| *Eulonchus tristis* | 008764 | unknown | CAS | USA, Oregon, Crook County, Maury Mts., Pine Creek Campground, [44.047, -120.391], 24.VII.1976 to 26.VII.1976, R. L. Westcott |
| *Eulonchus tristis* | 008765 | unknown | CAS | USA, Oregon, Crook County, Maury Mts., Pine Creek Campground, [44.047, -120.391], 24.VII.1976 to 26.VII.1976, R. L. Westcott |
| *Eulonchus tristis* | 008768 | unknown | CAS | USA, Idaho, Latah County, S. slope of Moscow Mountain, [46.793, -116.87], 6.VII.1968, R. L. Westcott |
| *Eulonchus tristis* | 008769 | unknown | CAS | USA, Idaho, Latah County, S. slope of Moscow Mountain, [46.793, -116.87], 6.VII.1968, R. L. Westcott |
| *Eulonchus tristis* | 008770 | unknown | CAS | USA, Idaho, Latah County, S. slope of Moscow Mountain, [46.793, -116.87], 6.VII.1968, R. L. Westcott |
| *Eulonchus tristis* | 008777 | unknown | CAS | USA, Oregon, Linn County, Monument Peak, [44.695, -122.322], 1372 m, 17.VII.1969, K.J. Goeden |
| *Eulonchus tristis* | 008906 | unknown | CAS | USA, Washington, Whitman County, Pullman, [46.731, -117.178], 3.VI.1969, malaise trap, R. W. Dawson |
| *Eulonchus tristis* | 008907 | unknown | CAS | USA, Washington, Whitman County, Pullman, [46.731, -117.178], 3.VI.1969, malaise trap, R. W. Dawson |
| *Eulonchus tristis* | 008909 | unknown | CAS | USA, Washington, Whitman County, Pullman, [46.731, -117.178], 5.VI.1969, malaise trap, R. W. Dawson |
| *Eulonchus tristis* | 008916 | unknown | CAS | USA, California, Modoc County, Cedar Pass, [41.563, -120.269], 26.VI.1958, J. W. Tilden |
| *Eulonchus tristis* | 008917 | unknown | CAS | USA, Idaho, Idaho County, Moose Creek, [46.121, -114.927], 30.VIII.1962, R.S. Dewey, J. E. Dewey |
| *Eulonchus tristis* | 008921 | unknown | CAS | USA, Washington, Whitman County, Pullman, [46.731, -117.178], 8.VI.1969, Malaise trap, R. W. Dawson |
| *Eulonchus tristis* | 008926 | unknown | CAS | USA, California, Siskiyou County, McCloud, [41.256, -122.14], 14.VI.1961, S. L. Wood, J. B. Karren, D.E. Bright |
| *Eulonchus tristis* | 008929 | unknown | CAS | USA, Oregon, Klamath County, Lake of the Woods, [42.366, -122.213], 22.VI.1975, G. F. Knowlton, W. J. Hanson |
| *Eulonchus tristis* | 008946 | unknown | CAS | USA, California, Fresno County, Kings Canyon, 10 mi Cr, [36.802, -118.696], 11.VI.1972, P. F. Torchio |
| *Eulonchus tristis* | 008947 | unknown | CAS | USA, California, Fresno County, Kings Canyon, 10 mi Cr, [36.802, -118.696], 11.VI.1972, P. F. Torchio |
| *Eulonchus tristis* | 008972 | unknown | UCDC | USA, California, Ojai Pass, 15.VI.1960, G. E. Bohart |
| *Eulonchus tristis* | 008979 | unknown | CAS | USA, Oregon, Jackson County, Grizzly Mt. Rd., [42.273, -122.612], 22.VI.1975, G. F. Knowlton, W. J. Hanson |
| *Eulonchus tristis* | 009069 | unknown | CAS | USA, California, Mendocino County, NCCRP (Northern California Coast Range Preserve), 4.8 km N of Branscomb, [39.697, -123.626], 427 m, time of day: midafternoon, 21.V.1982 to 23.V.1982, E. I. Schlinger |
| *Eulonchus tristis* | 009073 | unknown | CAS | USA, California, Mendocino County, NCCRP (Northern California Coast Range Preserve), 4.8 km N of Branscomb, [39.697, -123.626], 427 m, time of day: midafternoon, 21.V.1982 to 23.V.1982, E. I. Schlinger |
| *Eulonchus tristis* | 009075 | unknown | CAS | USA, California, Mendocino County, NCCRP (Northern California Coast Range Preserve), 4.8 km N of Branscomb, [39.697, -123.626], 427 m, time of day: midafternoon, 21.V.1982 to 23.V.1982, E. I. Schlinger |
| *Eulonchus tristis* | 009077 | unknown | CAS | USA, California, Mendocino County, NCCRP (Northern California Coast Range Preserve), 4.8 km N of Branscomb, [39.697, -123.626], 427 m, time of day: midafternoon, 21.V.1982 to 23.V.1982, E. I. Schlinger |
| *Eulonchus tristis* | 009081 | unknown | CAS | USA, California, Mendocino County, NCCRP (Northern California Coast Range Preserve), 4.8 km N of Branscomb, [39.697, -123.626], 427 m, time of day: midafternoon, 21.V.1982 to 23.V.1982, E. I. Schlinger |
| *Eulonchus tristis* | 009083 | unknown | CAS | USA, California, Mendocino County, NCCRP (Northern California Coast Range Preserve), 4.8 km N of Branscomb, [39.697, -123.626], 427 m, time of day: midafternoon, 21.V.1982 to 23.V.1982, E. I. Schlinger |
| *Eulonchus tristis* | 009084 | unknown | CAS | USA, California, Mendocino County, NCCRP (Northern California Coast Range Preserve), 4.8 km N of Branscomb, [39.697, -123.626], 427 m, time of day: midafternoon, 21.V.1982 to 23.V.1982, E. I. Schlinger |
| *Eulonchus tristis* | 009166 | unknown | CAS | USA, California, El Dorado County, North of Greenwood, Road to American River, [38.907, -120.911], 30.V.1976, E. I. Schlinger, Buegler |
| *Eulonchus tristis* | 009167 | unknown | CAS | USA, California, El Dorado County, N. of Greenwood, [38.904, -120.91], 30.V.1976, E. I. Schlinger, M. E. Buegler |
| *Eulonchus tristis* | 009169 | unknown | CAS | USA, California, Alameda County, Roberts area Oakland Hills, [37.817, -122.18], 6.VII.1976, Opler |
| *Eulonchus tristis* | 009171 | unknown | CAS | USA, California, Mendocino County, NCCRP (Northern California Coast Range Preserve), 8.1 km. N. of Branscomb, [39.77, -123.628], 26.V.1976 to 27.V.1976, R. Wharton |
| *Eulonchus tristis* | 009307 | unknown | CAS | USA, California, San Rafael Mts., Bluff Camp, [34.676, -119.667], 29.VI.1959, C. A. Campbell |
| *Eulonchus tristis* | 009315 | unknown | CAS | USA, California, Marin County, Mill Valley, =, [37.906, -122.545], 16.VII.1954, H. B. Leech |
| *Eulonchus tristis* | 009316 | unknown | CAS | USA, Oregon, Linn County, Hoodoo Ski Bowl, [44.409, -121.88], 1402 m, 25.VII.1966, P. Rude |
| *Eulonchus tristis* | 009317 | unknown | CAS | USA, California, Mariposa County, 5.6 km E. El Portal, [37.675, -119.721], 16.VI.1961, J. F. Lawrence |
| *Eulonchus tristis* | 009318 | unknown | CAS | USA, California, Napa County, 22.5 km east Rutherford, near Hennesey Dam, [38.457, -122.169], 22.V.1955, J. C. Downey |
| *Eulonchus tristis* | 009319 | unknown | CAS | USA, California, Mariposa County, 5.6 km E. El Portal, [37.675, -119.721], 16.VI.1961, J. F. Lawrence |
| *Eulonchus tristis* | 009320 | unknown | CAS | USA, California, Mariposa County, 5.6 km E. El Portal, [37.675, -119.721], 16.VI.1961, J. F. Lawrence |
| *Eulonchus tristis* | 009321 | unknown | CAS | USA, Oregon, Jackson County, Laurelhurst State Park, [45.519, -122.623], 20.VI.1962, C. A. or K. Toschi |
| *Eulonchus tristis* | 009322 | unknown | CAS | USA, California, San Luis Obispo County, 4.0 km S. of Creston, [35.483, -120.524], 4.V.1962, C. A. or K. Toschi |
| *Eulonchus tristis* | 009323 | unknown | CAS | USA, California, Santa Cruz County, 9.7 km N. of Boulder Creek, [37.214, -122.122], 5.VII.1959, J. Powell |
| *Eulonchus tristis* | 009324 | unknown | CAS | USA, California, San Luis Obispo County, 8.1 km northeast Santa Margarita, [35.446, -120.552], 6.X.1962, G. I. Stage |
| *Eulonchus tristis* | 009326 | unknown | CAS | USA, California, Santa Clara County, San Antonio Valley, [37.355, -121.92], 3.VI.1954, J. G. Rozen |
| *Eulonchus tristis* | 009327 | unknown | CAS | USA, California, Siskiyou County, 9.7 km NE of Weed, [41.49, -122.314], 8.VI.1963, D. H. Janzen |
| *Eulonchus tristis* | 009328 | unknown | CAS | USA, California, Siskiyou County, 9.7 km NE of Weed, [41.49, -122.314], 8.VI.1963, D. H. Janzen |
| *Eulonchus tristis* | 009329 | unknown | CAS | USA, California, Siskiyou County, 9.7 km NE of Weed, [41.49, -122.314], 8.VI.1963, D. H. Janzen |
| *Eulonchus tristis* | 009330 | unknown | CAS | USA, California, San Bernardino County, Summit, 27.VI.1962, A. E. Michelbacher |
| *Eulonchus tristis* | 009331 | unknown | CAS | USA, California, Berkeley, [37.871, -122.273], 15.VI.1952, J. G. Rozen |
| *Eulonchus tristis* | 009332 | unknown | CAS | USA, California, Berkeley, [37.871, -122.273], 27.III.1961, J.A. Goodwin |
| *Eulonchus tristis* | 009333 | unknown | CAS | USA, California, Berkeley, [37.871, -122.273], 15.VI.1952, J. G. Rozen |
| *Eulonchus tristis* | 009334 | unknown | CAS | USA, California, Contra Costa County, Lafayette, [37.886, -122.118], 17.VI.1956, R. A. Stirton |
| *Eulonchus tristis* | 009335 | unknown | CAS | USA, California, Contra Costa County, Lafayette, Woodlands, [37.885, -122.118], 9.VI.1956, R. A. Stirton |
| *Eulonchus tristis* | 009336 | unknown | CAS | USA, California, Contra Costa County, Lafayette, Woodlands, [37.885, -122.118], 9.VI.1956, R. A. Stirton |
| *Eulonchus tristis* | 009337 | unknown | CAS | USA, California, Modoc County, Buck Creek Ranger Station, [41.872, -120.292], 1570 m, 5.VI.1970 to 7.VI.1970, J. T. Doyen |
| *Eulonchus tristis* | 009384 | unknown | CAS | USA, California, Modoc County, 16.1 km N., 9.7 km.W.of Likely, [41.373, -120.62], 13.V.1958, A.M. Barnes |
| *Eulonchus tristis* | 009385 | unknown | CAS | USA, Washington, Columbia County No. 1, Lewis and Clark Park, [46.137, -123.88], 30.VI.1959, G. W. Byers |
| *Eulonchus tristis* | 009386 | unknown | CAS | USA, Washington, Columbia County No. 1, Lewis and Clark Park, [46.137, -123.88], 30.VI.1959, G. W. Byers |
| *Eulonchus tristis* | 009387 | unknown | CAS | USA, Washington, Columbia County No. 1, Lewis and Clark Park, [46.137, -123.88], 30.VI.1959, G. W. Byers |
| *Eulonchus tristis* | 009388 | unknown | CAS | USA, Washington, Columbia County No. 1, Lewis and Clark Park, [46.137, -123.88], 30.VI.1959, G. W. Byers |
| *Eulonchus tristis* | 009389 | unknown | CAS | USA, Washington, Columbia County No. 1, Lewis and Clark Park, [46.137, -123.88], 30.VI.1959, G. W. Byers |
| *Eulonchus tristis* | 009390 | unknown | CAS | USA, Oregon, Jackson County, #1, Union Creek, Campground, [42.914, -122.408], 23.VI.1959, G. W. Byers |
| *Eulonchus tristis* | 009391 | unknown | CAS | USA, Oregon, Jackson County, #1, Union Creek, Campground, [42.914, -122.408], 23.VI.1959, G. W. Byers |
| *Eulonchus tristis* | 009392 | unknown | CAS | USA, Oregon, Jackson County, #1, Union Creek, Campground, [42.914, -122.408], 23.VI.1959, G. W. Byers |
| *Eulonchus tristis* | 009393 | unknown | CAS | USA, Idaho, Latah County #1, 9.7 km N. of Moscow, [46.82, -117.001], 1.VII.1959, D.F. Beneway |
| *Eulonchus tristis* | 009398 | unknown | CAS | USA, Oregon, Jackson County #1, 16.1 km W. of Crater Lake Park, [42.869, -122.365], 1006 m, 23.VI.1959, D.F. Beneway |
| *Eulonchus tristis* | 009399 | unknown | CAS | USA, Oregon, Jackson County #1, 16.1 km W. of Crater Lake Park, [42.869, -122.365], 1006 m, 23.VI.1959, D.F. Beneway |
| *Eulonchus tristis* | 009400 | unknown | CAS | USA, Oregon, Jackson County #1, 16.1 km W. of Crater Lake Park, [42.869, -122.365], 1006 m, 23.VI.1959, D.F. Beneway |
| *Eulonchus tristis* | 009401 | unknown | CAS | USA, Oregon, Jackson County #1, 16.1 km W. of Crater Lake Park, [42.869, -122.365], 1006 m, 23.VI.1959, D.F. Beneway |
| *Eulonchus tristis* | 009402 | unknown | CAS | USA, California, Yosemite National Park, Arch Rock, [37.449, -119.076], 22.VI.1955, M. Wasbauer |
| *Eulonchus tristis* | 009403 | unknown | CAS | USA, California, Yosemite National Park, Arch Rock, [37.449, -119.076], 22.VI.1955, M. Wasbauer |
| *Eulonchus tristis* | 009404 | unknown | CAS | USA, California, Yosemite National Park, Arch Rock, [37.449, -119.076], 22.VI.1955, M. Wasbauer |
| *Eulonchus tristis* | 009405 | unknown | CAS | USA, California, Yosemite National Park, Arch Rock, [37.449, -119.076], 22.VI.1955, M. Wasbauer |
| *Eulonchus tristis* | 009406 | unknown | CAS | USA, California, Yosemite National Park, Arch Rock, [37.449, -119.076], 22.VI.1955, M. Wasbauer |
| *Eulonchus tristis* | 009407 | unknown | CAS | USA, California, Yosemite National Park, Arch Rock, [37.449, -119.076], 22.VI.1955, M. Wasbauer |
| *Eulonchus tristis* | 009408 | unknown | CAS | USA, California, Yosemite National Park, Arch Rock, [37.449, -119.076], 22.VI.1955, M. Wasbauer |
| *Eulonchus tristis* | 009409 | unknown | CAS | USA, California, Yosemite National Park, Arch Rock, [37.449, -119.076], 22.VI.1955, M. Wasbauer |
| *Eulonchus tristis* | 009410 | unknown | CAS | USA, California, Yosemite National Park, Arch Rock, [37.449, -119.076], 22.VI.1955, M. Wasbauer |
| *Eulonchus tristis* | 009411 | unknown | CAS | USA, California, Yosemite National Park, Arch Rock, [37.449, -119.076], 22.VI.1955, M. Wasbauer |
| *Eulonchus tristis* | 009412 | unknown | CAS | USA, California, Yosemite National Park, Arch Rock, [37.449, -119.076], 22.VI.1955, M. Wasbauer |
| *Eulonchus tristis* | 009413 | unknown | CAS | USA, California, Yosemite National Park, Arch Rock, [37.449, -119.076], 22.VI.1955, M. Wasbauer |
| *Eulonchus tristis* | 009414 | unknown | CAS | USA, California, Yosemite National Park, Arch Rock, [37.449, -119.076], 22.VI.1955, M. Wasbauer |
| *Eulonchus tristis* | 009422 | unknown | CAS | USA, Oregon, Klamath County, Crater Lake National Park, Boundary Springs, [43.066, -122.23], 5.VII.1957, D.H. Huntzinger |
| *Eulonchus tristis* | 009423 | unknown | CAS | USA, California, Tulare County, 0.5 km E. of Pine flat, [35.874, -118.638], 14.VI.1961, G. I. Stage, R. A. Snelling |
| *Eulonchus tristis* | 009431 | unknown | CAS | USA, California, Sonoma County, Kenwood, [38.414, -122.546], 2.V.1954, J. Powell |
| *Eulonchus tristis* | 009581 | male | CAS | USA, California, Trinity County, Dubakella Mtn. Road, 29N55, [40.381, -123.148], 1524 m, 10.VII.1998, M.R. Mesler |
| *Eulonchus tristis* | 009582 | male | CAS | USA, California, Trinity County, Dubakella Mtn. Road, 29N55, [40.381, -123.148], 1524 m, 10.VII.1998, M.R. Mesler |
| *Eulonchus tristis* | 009584 | male | CAS | USA, California, Trinity County, Dubakella Mtn. Road, 29N55, [40.381, -123.148], 1524 m, 10.VII.1998, M.R. Mesler |
| *Eulonchus tristis* | 009585 | male | CAS | USA, California, Trinity County, Dubakella Mtn. Road, 29N55, [40.381, -123.148], 1524 m, 10.VII.1998, M.R. Mesler |
| *Eulonchus tristis* | 009592 | unknown | CAS | USA, California, Trinity County, South Fork Mtn. Road, [40.442, -123.455], 1372 to 1524 m, 10.VII.1998, M.R. Mesler |
| *Eulonchus tristis* | 009593 | unknown | CAS | USA, California, Trinity County, South Fork Mtn. Road, [40.442, -123.455], 1372 to 1524 m, 10.VII.1998, M.R. Mesler |
| *Eulonchus tristis* | 009594 | unknown | CAS | USA, California, Trinity County, South Fork Mtn. Road, [40.442, -123.455], 1372 to 1524 m, 10.VII.1998, M.R. Mesler |
| *Eulonchus tristis* | 009595 | unknown | CAS | USA, California, Trinity County, South Fork Mtn. Road, [40.442, -123.455], 1372 to 1524 m, 10.VII.1998, M.R. Mesler |
| *Eulonchus tristis* | 009596 | unknown | CAS | USA, California, Trinity County, South Fork Mtn. Road, [40.442, -123.455], 1372 to 1524 m, 10.VII.1998, M.R. Mesler |
| *Eulonchus tristis* | 009597 | unknown | CAS | USA, California, Trinity County, South Fork Mtn. Road, [40.442, -123.455], 1372 to 1524 m, 10.VII.1998, M.R. Mesler |
| *Eulonchus tristis* | 009598 | unknown | CAS | USA, California, Trinity County, South Fork Mtn. Road, [40.442, -123.455], 1372 to 1524 m, 10.VII.1998, M.R. Mesler |
| *Eulonchus tristis* | 009599 | unknown | CAS | USA, California, Trinity County, South Fork Mtn. Road, [40.442, -123.455], 1372 to 1524 m, 10.VII.1998, M.R. Mesler |
| *Eulonchus tristis* | 009600 | male | CAS | USA, California, Trinity County, Dubakella Mtn. Road, 29N55, [40.381, -123.148], 1524 m, 10.VII.1998, M.R. Mesler |
| *Eulonchus tristis* | 009601 | male | CAS | USA, California, Trinity County, Dubakella Mtn. Road, 29N55, [40.381, -123.148], 1524 m, 10.VII.1998, M.R. Mesler |
| *Eulonchus tristis* | 009602 | male | CAS | USA, California, Trinity County, Dubakella Mtn. Road, 29N55, [40.381, -123.148], 1524 m, 10.VII.1998, M.R. Mesler |
| *Eulonchus tristis* | 009603 | male | CAS | USA, California, Humboldt County, Grouse Mtn., [40.759, -123.672], 1524 m, 8.VII.1998, M.R. Mesler |
| *Eulonchus tristis* | 009604 | male | CAS | USA, California, Humboldt County, Grouse Mtn., [40.759, -123.672], 1524 m, 8.VII.1998, M.R. Mesler |
| *Eulonchus tristis* | 009605 | male | CAS | USA, California, Humboldt County, Grouse Mtn., [40.759, -123.672], 1524 m, 8.VII.1998, M.R. Mesler |
| *Eulonchus tristis* | 009606 | male | CAS | USA, California, Humboldt County, Grouse Mtn., [40.759, -123.672], 1524 m, 8.VII.1998, M.R. Mesler |
| *Eulonchus tristis* | 009607 | male | CAS | USA, California, Humboldt County, Grouse Mtn., [40.759, -123.672], 1524 m, 8.VII.1998, M.R. Mesler |
| *Eulonchus tristis* | 009608 | male | CAS | USA, California, Humboldt County, Grouse Mtn., [40.759, -123.672], 1524 m, 8.VII.1998, M.R. Mesler |
| *Eulonchus tristis* | 009609 | male | CAS | USA, California, Humboldt County, Grouse Mtn., [40.759, -123.672], 1524 m, 5.VII.1998, M.R. Mesler |
| *Eulonchus tristis* | 009610 | male | CAS | USA, California, Humboldt County, Grouse Mtn., [40.759, -123.672], 1524 m, 5.VII.1998, M.R. Mesler |
| *Eulonchus tristis* | 010273 | unknown | CAS | USA, Oregon, Linn County, 32.2 km E. of Cascadia, [44.397, -122.082], 25.VI.1954, A. A. Grigarick |
| *Eulonchus tristis* | 010274 | unknown | CAS | USA, Oregon, Linn County, 32.2 km E. of Cascadia, [44.397, -122.082], 25.VI.1954, A. A. Grigarick |
| *Eulonchus tristis* | 010275 | unknown | CAS | USA, Oregon, Linn County, 16.1 km E. of Cascadia, [44.397, -122.283], 25.VI.1954, A. A. Grigarick |
| *Eulonchus tristis* | 010276 | unknown | CAS | USA, Oregon, Linn County, 16.1 km E. of Cascadia, [44.397, -122.283], 25.VI.1954, A. A. Grigarick |
| *Eulonchus tristis* | 010277 | unknown | CAS | USA, Oregon, Linn County, 16.1 km E. of Cascadia, [44.397, -122.283], 25.VI.1954, A. A. Grigarick |
| *Eulonchus tristis* | 010278 | unknown | CAS | USA, Oregon, Linn County, 32.2 km E. of Cascadia, [44.397, -122.082], 25.VI.1954, J. C. Downey |
| *Eulonchus tristis* | 010279 | unknown | CAS | USA, Oregon, Linn County, 32.2 km E. of Cascadia, [44.397, -122.082], 25.VI.1954, J. C. Downey |
| *Eulonchus tristis* | 010280 | unknown | CAS | USA, Oregon, Linn County, N. Santiam River, 16.1 km North of Hwy. 20, [44.494, -121.927], 24.VI.1954, E. I. Schlinger |
| *Eulonchus tristis* | 010281 | unknown | CAS | USA, Oregon, Linn County, N. Santiam River, 16.1 km North of Hwy. 20, [44.494, -121.927], 24.VI.1954, E. I. Schlinger |
| *Eulonchus tristis* | 010282 | unknown | CAS | USA, Oregon, Linn County, N. Santiam River, 16.1 km North of Hwy. 20, [44.494, -121.927], 24.VI.1954, E. I. Schlinger |
| *Eulonchus tristis* | 010283 | unknown | CAS | USA, Oregon, Linn County, N. Santiam River, 16.1 km North of Hwy. 20, [44.494, -121.927], 24.VI.1954, E. I. Schlinger |
| *Eulonchus tristis* | 010284 | unknown | CAS | USA, Oregon, Linn County, N. Santiam River, 16.1 km North of Hwy. 20, [44.494, -121.927], 24.VI.1954, E. I. Schlinger |
| *Eulonchus tristis* | 010285 | unknown | CAS | USA, Oregon, Linn County, N. Santiam River, 16.1 km North of Hwy. 20, [44.494, -121.927], 24.VI.1954, E. I. Schlinger |
| *Eulonchus tristis* | 010286 | unknown | CAS | USA, Oregon, Linn County, N. Santiam River, 16.1 km North of Hwy. 20, [44.494, -121.927], 24.VI.1954, J. C. Downey |
| *Eulonchus tristis* | 010287 | unknown | CAS | USA, Oregon, Linn County, N. Santiam River, 16.1 km North of Hwy. 20, [44.494, -121.927], 24.VI.1954, J. C. Downey |
| *Eulonchus tristis* | 010288 | unknown | CAS | USA, Oregon, Linn County, N. Santiam River, 16.1 km North of Hwy. 20, [44.494, -121.927], 24.VI.1954, J. C. Downey |
| *Eulonchus tristis* | 010289 | unknown | CAS | USA, Oregon, Linn County, N. Santiam River, 16.1 km North of Hwy. 20, [44.494, -121.927], 24.VI.1954, J. C. Downey |
| *Eulonchus tristis* | 010290 | unknown | CAS | USA, Oregon, Linn County, N. Santiam River, 16.1 km North of Hwy. 20, [44.494, -121.927], 24.VI.1954, J. C. Downey |
| *Eulonchus tristis* | 010291 | female | CAS | USA, Oregon, Linn County, N. Santiam River, 16.1 km North of Hwy. 20, [44.494, -121.927], 24.VI.1954, J. C. Downey |
| *Eulonchus tristis* | 010292 | female | CAS | USA, Oregon, Linn County, N. Santiam River, 16.1 km North of Hwy. 20, [44.494, -121.927], 24.VI.1954, A. A. Grigarick |
| *Eulonchus tristis* | 010293 | unknown | OSUC | USA, Oregon, Lost Prairie, Santiam Pass, [44.402, -121.851], 1.VII.1948 |
| *Eulonchus tristis* | 010294 | unknown | OSUC | USA, Oregon, Marion County, Breitenbush Spring, [44.781, -121.975], 14.VI.1942, R. E. Rieder |
| *Eulonchus tristis* | 010295 | unknown | OSUC | USA, Oregon, Marion County, Breitenbush Spring, [44.781, -121.975], 14.VI.1942, R. E. Rieder |
| *Eulonchus tristis* | 010296 | unknown | OSUC | USA, Oregon, Marion County, Breitenbush Spring, [44.781, -121.975], 14.VI.1942, R. E. Rieder |
| *Eulonchus tristis* | 010297 | unknown | OSUC | USA, Oregon, Marion County, Breitenbush Spring, [44.781, -121.975], 14.VI.1942, R. E. Rieder |
| *Eulonchus tristis* | 010303 | unknown | OSUC | USA, Oregon, Marion County, Breitenbush Spring, [44.781, -121.975], 14.VI.1942, R. E. Rieder |
| *Eulonchus tristis* | 010304 | unknown | OSUC | USA, Oregon, Marion County, Breitenbush Spring, [44.781, -121.975], 14.VI.1942, R. E. Rieder |
| *Eulonchus tristis* | 010305 | unknown | OSUC | USA, Oregon, Marion County, Breitenbush Spring, [44.781, -121.975], 14.VI.1942, R. E. Rieder |
| *Eulonchus tristis* | 010306 | unknown | OSUC | USA, Oregon, Marion County, Breitenbush Spring, [44.781, -121.975], 14.VI.1942, R. E. Rieder |
| *Eulonchus tristis* | 010307 | unknown | OSUC | USA, Oregon, Marion County, Breitenbush Spring, [44.781, -121.975], 14.VI.1942, R. E. Rieder |
| *Eulonchus tristis* | 010308 | unknown | OSUC | USA, Oregon, Marion County, Breitenbush Spring, [44.781, -121.975], 14.VI.1942, R. E. Rieder |
| *Eulonchus tristis* | 010309 | unknown | OSUC | USA, Oregon, Swim, 2.VII.1942, Geo. Ferguson |
| *Eulonchus tristis* | 010310 | unknown | OSUC | USA, Oregon, Swim, 2.VII.1942, Geo. Ferguson |
| *Eulonchus tristis* | 010311 | unknown | OSUC | USA, Oregon, Swim, 2.VII.1942, Geo. Ferguson |
| *Eulonchus tristis* | 010312 | unknown | OSUC | USA, Oregon, Swim, 2.VII.1942, Geo. Ferguson |
| *Eulonchus tristis* | 010313 | unknown | OSUC | USA, Oregon, Swim, 2.VII.1942, Geo. Ferguson |
| *Eulonchus tristis* | 010314 | unknown | OSUC | USA, Oregon, Swim, 2.VII.1942, Geo. Ferguson |
| *Eulonchus tristis* | 010315 | unknown | OSUC | USA, Oregon, Swim, 2.VII.1942, Geo. Ferguson |
| *Eulonchus tristis* | 010316 | unknown | OSUC | USA, Oregon, Swim, 2.VII.1942, Geo. Ferguson |
| *Eulonchus tristis* | 010317 | unknown | OSUC | USA, Oregon, Swim, 2.VII.1942, Geo. Ferguson |
| *Eulonchus tristis* | 010318 | unknown | OSUC | USA, Oregon, Swim, 2.VII.1942, Geo. Ferguson |
| *Eulonchus tristis* | 010319 | unknown | OSUC | USA, Oregon, Swim, 2.VII.1942, Geo. Ferguson |
| *Eulonchus tristis* | 010320 | unknown | OSUC | USA, Oregon, Swim, 2.VII.1942, Geo. Ferguson |
| *Eulonchus tristis* | 010321 | unknown | OSUC | USA, Oregon, Swim, 2.VII.1942, Geo. Ferguson |
| *Eulonchus tristis* | 010326 | unknown | CAS | USA, Oregon, Columbia River, Starvation Creek State Park, [45.688, -121.691], 17.VI.1957, E. I. Schlinger |
| *Eulonchus tristis* | 010327 | unknown | CAS | USA, Oregon, Columbia River, Starvation Creek State Park, [45.688, -121.691], 17.VI.1957, E. I. Schlinger |
| *Eulonchus tristis* | 010328 | unknown | CAS | USA, Oregon, Columbia River, Starvation Creek State Park, [45.688, -121.691], 17.VI.1957, E. I. Schlinger |
| *Eulonchus tristis* | 010329 | unknown | CAS | USA, Oregon, Columbia River, Starvation Creek State Park, [45.688, -121.691], 17.VI.1957, E. I. Schlinger |
| *Eulonchus tristis* | 010330 | unknown | CAS | USA, Oregon, Columbia River, Starvation Creek State Park, [45.688, -121.691], 17.VI.1957, E. I. Schlinger |
| *Eulonchus tristis* | 010331 | unknown | CAS | USA, Oregon, Columbia River, Starvation Creek State Park, [45.688, -121.691], 17.VI.1957, E. I. Schlinger |
| *Eulonchus tristis* | 010332 | unknown | CAS | USA, Oregon, Columbia River, Starvation Creek State Park, [45.688, -121.691], 17.VI.1957, E. I. Schlinger |
| *Eulonchus tristis* | 010335 | unknown | CAS | USA, California, Humboldt County, Meyers Flat, [40.266, -123.87], 20.VI.1957, E. I. Schlinger |
| *Eulonchus tristis* | 010336 | unknown | CAS | USA, California, Humboldt County, Meyers Flat, [40.266, -123.87], 20.VI.1957, E. I. Schlinger |
| *Eulonchus tristis* | 010337 | unknown | CAS | USA, California, Humboldt County, Meyers Flat, [40.266, -123.87], 20.VI.1957, E. I. Schlinger |
| *Eulonchus tristis* | 010338 | unknown | CAS | USA, California, Humboldt County, Meyers Flat, [40.266, -123.87], 20.VI.1957, E. I. Schlinger |
| *Eulonchus tristis* | 010339 | unknown | CAS | USA, California, Humboldt County, Meyers Flat, [40.266, -123.87], 20.VI.1957, E. I. Schlinger |
| *Eulonchus tristis* | 010340 | unknown | CAS | USA, California, Humboldt County, Meyers Flat, [40.266, -123.87], 20.VI.1957, E. I. Schlinger |
| *Eulonchus tristis* | 010341 | unknown | CAS | USA, Oregon, Klamath County, Deschutes National Forest, Crescent Lake, [43.475, -121.986], 2.VII.1952, E.R. Jaycox |
| *Eulonchus tristis* | 010342 | unknown | CAS | USA, Oregon, Klamath County, Deschutes National Forest, Crescent Lake, [43.475, -121.986], 5.VII.1952, E.R. Jaycox |
| *Eulonchus tristis* | 010343 | unknown | CAS | USA, Oregon, Klamath County, Deschutes National Forest, Crescent Lake, [43.475, -121.986], 5.VII.1952, E.R. Jaycox |
| *Eulonchus tristis* | 010344 | unknown | CAS | USA, Oregon, Klamath County, Deschutes National Forest, Crescent Lake, [43.475, -121.986], 5.VII.1952, E.R. Jaycox |
| *Eulonchus tristis* | 010345 | unknown | CAS | USA, Oregon, Klamath County, Deschutes National Forest, Crescent Lake, [43.475, -121.986], 5.VII.1952, E.R. Jaycox |
| *Eulonchus tristis* | 010346 | unknown | CAS | USA, Oregon, Klamath County, Deschutes National Forest, Crescent Lake, [43.475, -121.986], 5.VII.1952, E.R. Jaycox |
| *Eulonchus tristis* | 010347 | unknown | CAS | USA, Oregon, Klamath County, Deschutes National Forest, Crescent Lake, [43.475, -121.986], 5.VII.1952, E.R. Jaycox |
| *Eulonchus tristis* | 010348 | unknown | CAS | USA, Oregon, Klamath County, Deschutes National Forest, Crescent Lake, [43.475, -121.986], 5.VII.1952, E.R. Jaycox |
| *Eulonchus tristis* | 010349 | unknown | CAS | USA, Oregon, Klamath County, Deschutes National Forest, Crescent Lake, [43.475, -121.986], 5.VII.1952, E.R. Jaycox |
| *Eulonchus tristis* | 010350 | unknown | CAS | USA, Oregon, Klamath County, Deschutes National Forest, Crescent Lake, [43.475, -121.986], 5.VII.1952, E.R. Jaycox |
| *Eulonchus tristis* | 010351 | unknown | CAS | USA, Oregon, Klamath County, Deschutes National Forest, Crescent Lake, [43.475, -121.986], 5.VII.1952, E.R. Jaycox |
| *Eulonchus tristis* | 010352 | unknown | CAS | USA, Oregon, Klamath County, Deschutes National Forest, Crescent Lake, [43.475, -121.986], 5.VII.1952, E.R. Jaycox |
| *Eulonchus tristis* | 010353 | unknown | CAS | USA, Oregon, Klamath County, Deschutes National Forest, Crescent Lake, [43.475, -121.986], 5.VII.1952, E.R. Jaycox |
| *Eulonchus tristis* | 010354 | unknown | CAS | USA, Oregon, Klamath County, Deschutes National Forest, Crescent Lake, [43.475, -121.986], 5.VII.1952, E.R. Jaycox |
| *Eulonchus tristis* | 010355 | unknown | CAS | USA, Oregon, Klamath County, Deschutes National Forest, Crescent Lake, [43.475, -121.986], 5.VII.1952, E.R. Jaycox |
| *Eulonchus tristis* | 010356 | unknown | CAS | USA, Oregon, Klamath County, Deschutes National Forest, Crescent Lake, [43.475, -121.986], 5.VII.1952, E.R. Jaycox |
| *Eulonchus tristis* | 010357 | unknown | CAS | USA, Oregon, Klamath County, Deschutes National Forest, Crescent Lake, [43.475, -121.986], 5.VII.1952, E.R. Jaycox |
| *Eulonchus tristis* | 010358 | unknown | CAS | USA, Oregon, Klamath County, Deschutes National Forest, Crescent Lake, [43.475, -121.986], 5.VII.1952, E.R. Jaycox |
| *Eulonchus tristis* | 010359 | unknown | CAS | USA, Oregon, Klamath County, Deschutes National Forest, Crescent Lake, [43.475, -121.986], 5.VII.1952, E.R. Jaycox |
| *Eulonchus tristis* | 010360 | unknown | CAS | USA, California, Humboldt County, Williams Grove State Park, [40.277, -123.884], 29.V.1955, E. I. Schlinger |
| *Eulonchus tristis* | 010361 | unknown | CAS | USA, California, Humboldt County, Williams Grove State Park, [40.277, -123.884], 29.V.1955, E. I. Schlinger |
| *Eulonchus tristis* | 010362 | unknown | CAS | USA, California, Humboldt County, Williams Grove State Park, [40.277, -123.884], 29.V.1955, E. I. Schlinger |
| *Eulonchus tristis* | 010363 | unknown | CAS | USA, California, Humboldt County, Williams Grove State Park, [40.277, -123.884], 29.V.1955, J. C. Downey |
| *Eulonchus tristis* | 010364 | unknown | CAS | USA, California, Humboldt County, Williams Grove State Park, [40.277, -123.884], 29.V.1955, J. C. Downey |
| *Eulonchus tristis* | 010365 | unknown | CAS | USA, California, Humboldt County, Williams Grove State Park, [40.277, -123.884], 29.V.1955, J. C. Downey |
| *Eulonchus tristis* | 010366 | unknown | CAS | USA, California, Humboldt County, Williams Grove State Park, [40.277, -123.884], 29.V.1955, J. C. Downey |
| *Eulonchus tristis* | 010367 | unknown | CAS | USA, California, Humboldt County, Williams Grove State Park, [40.277, -123.884], 29.V.1955, J. C. Downey |
| *Eulonchus tristis* | 010368 | unknown | CAS | USA, California, Humboldt County, Redcrest, [40.401, -123.95], 29.V.1955, E. I. Schlinger |
| *Eulonchus tristis* | 010369 | unknown | CAS | USA, California, Humboldt County, Redcrest, [40.401, -123.95], 29.V.1955, J. C. Downey |
| *Eulonchus tristis* | 010370 | unknown | CAS | USA, California, Humboldt County, Redcrest, [40.401, -123.95], 29.V.1955, J. C. Downey |
| *Eulonchus tristis* | 010371 | unknown | CAS | USA, California, Humboldt County, 4.8 km. E. of Blue Lake, [40.883, -123.927], 28.V.1955, E. I. Schlinger |
| *Eulonchus tristis* | 010372 | unknown | CAS | USA, California, Humboldt County, 4.8 km. E. of Blue Lake, [40.883, -123.927], 28.V.1955, E. I. Schlinger |
| *Eulonchus tristis* | 010373 | unknown | CAS | USA, California, Humboldt County, 4.8 km. E. of Blue Lake, [40.883, -123.927], 28.V.1955, J. C. Downey |
| *Eulonchus tristis* | 010374 | unknown | CAS | USA, California, Humboldt County, Fort Seward, [40.223, -123.643], 23.V.1935, F.R. Platt |
| *Eulonchus tristis* | 010375 | unknown | CAS | USA, Oregon, Odell Lake, [43.566, -121.98], 25.VI.1960, E. I. Schlinger |
| *Eulonchus tristis* | 010376 | unknown | CAS | USA, Oregon, Odell Lake, [43.566, -121.98], 25.VI.1960, E. I. Schlinger |
| *Eulonchus tristis* | 010377 | unknown | USNM | USA, California, Humboldt County, E. C. Van Dyke |
| *Eulonchus tristis* | 010378 | unknown | USNM | USA, California, Humboldt County, Bair's Ranch, Redwood Creek, 11.VI.1903, H. S. Barber |
| *Eulonchus tristis* | 010381 | unknown | CAS | USA, California, Humboldt County, Scotia, [40.482, -124.101], 12.VI.1925, J. O. Martin |
| *Eulonchus tristis* | 010382 | unknown | CAS | USA, California, Humboldt County, Scotia, [40.482, -124.101], 12.VI.1925, J. O. Martin |
| *Eulonchus tristis* | 010383 | unknown | CAS | USA, California, Humboldt County, Scotia, [40.482, -124.101], 12.VI.1925, J. O. Martin |
| *Eulonchus tristis* | 010384 | unknown | USNM | USA, California, Orlick (probably Orick), [41.286, -124.06], 21.VI.1935, A. L. Melander |
| *Eulonchus tristis* | 010385 | unknown | DEI | USA, California, Del Norte County, 29.V.1910, Oldenberg |
| *Eulonchus tristis* | 010386 | unknown | OSUC | USA, Oregon, Linn County, Trout Creek Camp, S. Santiam Pass, [44.394, -121.929], 19.V.1940, H. A. Scullen |
| *Eulonchus tristis* | 010387 | unknown | OSUC | USA, Oregon, Linn County, 9.7 km E. of Cascadia, [44.397, -122.364], 18.V.1940, H. A. Scullen |
| *Eulonchus tristis* | 010388 | unknown | UCDC | USA, Oregon, Silver Falls, [44.903, -123.585], 22.VI.1939, G. E. Bohart |
| *Eulonchus tristis* | 010389 | unknown | UCDC | USA, Oregon, Silver Falls, [44.903, -123.585], 22.VI.1939, G. E. Bohart |
| *Eulonchus tristis* | 010390 | unknown | UCDC | USA, Oregon, Silver Falls, [44.903, -123.585], 22.VI.1939, G. E. Bohart |
| *Eulonchus tristis* | 010391 | unknown | UCDC | USA, Oregon, Silver Falls, [44.903, -123.585], 22.VI.1939, G. E. Bohart |
| *Eulonchus tristis* | 010392 | unknown | UCDC | USA, Oregon, Silver Falls, [44.903, -123.585], 22.VI.1939, G. E. Bohart |
| *Eulonchus tristis* | 010393 | unknown | UCDC | USA, Oregon, Silver Falls, [44.903, -123.585], 22.VI.1939, G. E. Bohart |
| *Eulonchus tristis* | 010394 | unknown | UCDC | USA, Oregon, Silver Falls, [44.903, -123.585], 22.VI.1939, G. E. Bohart |
| *Eulonchus tristis* | 010395 | unknown | UCDC | USA, Oregon, Silver Falls, [44.903, -123.585], 22.VI.1939, G. E. Bohart |
| *Eulonchus tristis* | 010396 | unknown | UCDC | USA, Oregon, Silver Falls, [44.903, -123.585], 22.VI.1939, G. E. Bohart |
| *Eulonchus tristis* | 010397 | unknown | UCDC | USA, Oregon, Silver Falls, [44.903, -123.585], 22.VI.1939, G. E. Bohart |
| *Eulonchus tristis* | 010398 | unknown | UCDC | USA, Oregon, Silver Falls, [44.903, -123.585], 22.VI.1939, G. E. Bohart |
| *Eulonchus tristis* | 010399 | unknown | CAS | USA, Oregon, Linn County, Monument Peak, [44.695, -122.322], 1372 m, 2.VIII.1953, P.O. Ritcher |
| *Eulonchus tristis* | 010400 | unknown | CAS | USA, Oregon, Linn County, Monument Peak, [44.695, -122.322], 1372 m, 2.VIII.1953, P.O. Ritcher |
| *Eulonchus tristis* | 010401 | unknown | CAS | USA, Oregon, Linn County, Monument Peak, [44.695, -122.322], 1372 m, 2.VIII.1953, P.O. Ritcher |
| *Eulonchus tristis* | 010402 | unknown | CAS | USA, Oregon, Linn County, Monument Peak, [44.695, -122.322], 1372 m, 2.VIII.1953, P.O. Ritcher |
| *Eulonchus tristis* | 010403 | unknown | CAS | USA, Oregon, Linn County, Monument Peak, [44.695, -122.322], 1372 m, 2.VIII.1953, P.O. Ritcher |
| *Eulonchus tristis* | 010404 | unknown | CAS | USA, Oregon, Linn County, Monument Peak, [44.695, -122.322], 1372 m, 2.VIII.1953, P.O. Ritcher |
| *Eulonchus tristis* | 010405 | unknown | CAS | USA, Oregon, Linn County, Monument Peak, [44.695, -122.322], 1372 m, 2.VIII.1953, P.O. Ritcher |
| *Eulonchus tristis* | 010441 | female | CAS | USA, California, Alameda Co., Strawberry Canyon, Berkeley, fire trail, [37.874, -122.228], 27.VI.1977, M. E. Buegler |
| *Eulonchus tristis* | 010442 | female | CAS | USA, California, Alameda Co., Strawberry Canyon, Berkeley, fire trail, [37.874, -122.228], 27.VI.1977, M. E. Buegler |
| *Eulonchus tristis* | 011566 | male | CAS | USA, California, Marin County, Mill Valley, =, [37.906, -122.545], 15.V.1921 |
| *Eulonchus tristis* | 011567 | female | CAS | USA, California, San Bernardino County, Mojave Desert, near Baker, .IV.1935, I. McCracken |
| *Eulonchus tristis* | 011568 | male | CAS | USA, California, San Bernardino County, Mojave Desert, near Baker, .IV.1935, I. McCracken |
| *Eulonchus tristis* | 011573 | male | CAS | USA, California, Santa Cruz, Santa Cruz Mountains, Santa Cruz Mountains, Felton, 100 to 167 m, 15.V.1907, J. C. Bradley |
| *Eulonchus tristis* | 011574 | male | CAS | USA, California, Humbolt County, Weott, 12.VII.1929, E. C. Van Dyke |
| *Eulonchus tristis* | 011575 | male | CAS | USA, California, Alameda County, Berkeley, 21.V.1915, M.C. VanDuzee |
| *Eulonchus tristis* | 011578 | male | CAS | USA, California, Humbolt Co., Blacksburg, 22.VI.1937, I. McCracken |
| *Eulonchus tristis* | 011579 | male | CAS | USA, California, San Francisco Co., San Francisco, [37.78, -122.42], H. Edwards |
| *Eulonchus tristis* | 011580 | male | CAS | USA, California, Humboldt County, Dinsmores, [40.491, -123.607], 23.VI.1937, I. McCracken |
| *Eulonchus tristis* | 011581 | male | CAS | USA, California, Humboldt County, Dinsmores, [40.491, -123.607], 23.VI.1937, I. McCracken |
| *Eulonchus tristis* | 011582 | male | CAS | USA, California, Alameda County, Hills back of Oakland, HIlls, 8.V.1910, E. C. Van Dyke |
| *Eulonchus tristis* | 011583 | male | CAS | USA, California, Marin CO., Redwood Canon, Redwood Canon, 17.V.1908, E. T. Cresson, Cresson |
| *Eulonchus tristis* | 011584 | male | CAS | USA, California, Santa Clara County, Permanente Cr., Permanente Cr., 6.V.1917 |
| *Eulonchus tristis* | 011585 | male | CAS | USA, California, Alameda County, Berkeley, 1.V.1938, M. A. Cazier |
| *Eulonchus tristis* | 011586 | male | CAS | USA, California, Humbolt Co., Blacksburg, 22.VI.1937, I. McCracken |
| *Eulonchus tristis* | 011587 | male | CAS | USA, California, Alameda Co., Berekley Hills, 16.VI.1933, P. H. Timberlake |
| *Eulonchus tristis* | 011588 | male | CAS | USA, California, Alameda Co., Berekley Hills, 16.VI.1933, P. H. Timberlake |
| *Eulonchus tristis* | 011589 |  | CAS | USA, California, H. Edwards |
| *Eulonchus tristis* | 011590 | male | UCDC | USA, California, Solano Co., Green Valley, Green Valley, 20.III.1936, G. E. Bohart |
| *Eulonchus tristis* | 011591 | male | CAS | USA, California, Sonoma County, [38.292, -122.458] |
| *Eulonchus tristis* | 011592 | male | CAS | USA, California, Marin County, Mill Valley, =, [37.906, -122.545], 11.IV.1926 |
| *Eulonchus tristis* | 011595 | male | CAS | USA, California, Mendocino County, 12.VI.1920, R. E. Leech |
| *Eulonchus tristis* | 011596 | male | CAS | USA, California, Baron |
| *Eulonchus tristis* | 011597 | male | CAS | USA, California, Montery County, Alma College, 6.V.1951, E. L. Kessel |
| *Eulonchus tristis* | 011598 | male | CAS | USA, California, Marin Co., Muirwoods, 19.V.1915, M.C. VanDuzee |
| *Eulonchus tristis* | 011599 | male | CAS | USA, California, Santa Clara Co., Saratoga, [37.264, -122.023], 18.IV.1924, R. E. Leech |
| *Eulonchus tristis* | 011600 | male | CAS | USA, California, Sonoma County, Sobre Vista, [38.333, -122.511], 31.IV.1910, J. A. Kusche |
| *Eulonchus tristis* | 011601 | male | CAS | USA, California, Marin County, Mill Valley, =, [37.906, -122.545], 21.VII.1926, M.C. VanDuzee |
| *Eulonchus tristis* | 011602 | male | CAS | USA, California, Marin County, Mill Valley, =, [37.906, -122.545], 11.IV.1926 |
| *Eulonchus tristis* | 011603 | male | CAS | USA, California, Marin Co., Muir Woods, 23.IV.1911, F. E. Blaisdell |
| *Eulonchus tristis* | 011604 | male | CAS | USA, California, San Mateo Co., 7.V.1916, E. C. Van Dyke |
| *Eulonchus tristis* | 011609 | female | CAS | USA, California, Alameda County, Oakland, .V.1931 |
| *Eulonchus tristis* | 011610 | female | CAS | USA, California, Marin Co., Mt. Tamalpais, Mt. Tamalpais, 16.V.1920, E. C. Van Dyke |
| *Eulonchus tristis* | 011611 | female | CAS | USA, California, Mendocino County, 12.VI.1920, R. E. Leech |
| *Eulonchus tristis* | 011612 | female | CAS | USA, California, Alameda Co., Berekley Hills, 16.VI.1933, P. H. Timberlake |
| *Eulonchus tristis* | 011613 | female | CAS | USA, California, San Mateo County, Corte Madera Creek, 25.IV.1900 |
| *Eulonchus tristis* | 011614 | female | CAS | USA, California, San Mateo County, Stanford University, 19.IV.1922 |
| *Eulonchus tristis* | 011615 | female | CAS | USA, California, Alameda County, Sunol, 14.V.1922, C. L. Fox |
| *Eulonchus tristis* | 011616 | male | UCDC | USA, California, Alameda County, Berkeley, 20.V.1937, R. M. Bohart, G. E. Bohart |
| *Eulonchus tristis* | 011617 | female | CAS | USA, California, Santa Cruz County, Ben Lomond, 17.VI.1951, C. D. MacNeill |
| *Eulonchus tristis* | 011618 | female | CAS | USA, California, Trinity County, 14.V.1925, R. E. Leech |
| *Eulonchus tristis* | 011619 | female | CAS | USA, California, Redwood Co., 23.V.1915 |
| *Eulonchus tristis* | 011620 | female | CAS | USA, California, Alameda County, Berkeley, 15.V.1915 |
| *Eulonchus tristis* | 011621 | female | CAS | USA, California, Marin Co., Mt. Tamalpais, Mt. Tamalpais, 16.V.1920, E. C. Van Dyke |
| *Eulonchus tristis* | 011658 | unknown | CAS | USA, California, Trinity County, 16.1 km North of Coffee Creek Ranger Station, [41.233, -122.706], 14.VII.1955, H. M. Kimball |
| *Eulonchus tristis* | 011659 | unknown | CAS | USA, California, Lassen County, Lassen National Park, Manzanita Lake, [40.533, -121.567], 13.VI.1941, C. W. Anderson |
| *Eulonchus tristis* | 011660 | female | CAS | USA, California, Plumas Co., Onion Valley, [39.793, -120.879], 7.VII.1949, E. I. Schlinger |
| *Eulonchus tristis* | 011662 | unknown | CAS | USA, California, Plumas Co., Onion Valley, [39.793, -120.879], 7.VII.1949, W. R. Schreader |
| *Eulonchus tristis* | 011663 | unknown | CAS | USA, Oregon, Klamath County, Klamath Lake, Eagle Ridge, [42.418, -121.953], 2.VI.1924, C. L. Fox |
| *Eulonchus tristis* | 011664 | unknown | CAS | USA, California, Tulare County, Sequoia National Park, Ash Mountain, [36.496, -118.834], 9.VI.1952, R. C. Bechtel |
| *Eulonchus tristis* | 011665 | unknown | CAS | USA, California, Tulare County, Sequoia National Park, Ash Mountain, [36.496, -118.834], 9.VI.1952, R. C. Bechtel |
| *Eulonchus tristis* | 011666 | unknown | CAS | USA, California, Tulare County, Sequoia National Park, Potwisha, [36.517, -118.8], 610 to 1524 m, 13.VI.1929, E. C. Van Dyke |
| *Eulonchus tristis* | 011667 | unknown | CAS | USA, California, Tulare County, Sequoia National Park, [36.488, -118.567], 610 to 1524 m, 13.VI.1929, C. H. Martin |
| *Eulonchus tristis* | 011672 | unknown | CAS | USA, California, Tulare Co., California Hot Springs, [35.88, -118.674], 3.VI.1939, E. C. Van Dyke |
| *Eulonchus tristis* | 011673 | unknown | CAS | USA, California, Kern Co., Kern River Canyon, [35.611, -118.49], 6.V.1931, C. H. Martin |
| *Eulonchus tristis* | 011674 | unknown | CAS | USA, California, Kern Co., Kern River Canyon, [35.611, -118.49], 6.V.1931, C. H. Martin |
| *Eulonchus tristis* | 011677 | unknown | CAS | USA, California, Santa Cruz Co., In redwoods, Soquel, [36.989, -121.957], 19.VI.1950, M. T. James |
| *Eulonchus tristis* | 011678 | unknown | CAS | USA, California, Santa Cruz Co., In redwoods, Soquel, [36.989, -121.957], 23.VI.1950, M. T. James |
| *Eulonchus tristis* | 011679 | unknown | CAS | USA, California, Santa Cruz Co., In redwoods, Soquel, [36.989, -121.957], 23.VI.1950, M. T. James |
| *Eulonchus tristis* | 011681 | unknown | CAS | USA, California, Santa Cruz Co., In redwoods, Soquel, [36.989, -121.957], 23.VI.1950, M. T. James |
| *Eulonchus tristis* | 011682 | unknown | CAS | USA, California, Santa Cruz Co., In redwoods, Soquel, [36.989, -121.957], 23.VI.1950, M. T. James |
| *Eulonchus tristis* | 011683 | unknown | CAS | USA, California, Santa Cruz Co., In redwoods, Soquel, [36.989, -121.957], 23.VI.1950, M. T. James |
| *Eulonchus tristis* | 011684 | unknown | CAS | USA, California, Santa Cruz Co., In redwoods, Soquel, [36.989, -121.957], 23.VI.1950, M. T. James |
| *Eulonchus tristis* | 011685 | unknown | CAS | USA, California, Santa Cruz Co., In redwoods, Soquel, [36.989, -121.957], 23.VI.1950, M. T. James |
| *Eulonchus tristis* | 011686 | unknown | CAS | USA, California, Santa Cruz Co., 9 miles north east of Soquel, [37.087, -121.851], 4.VII.1956, S. M. Fidel |
| *Eulonchus tristis* | 011687 | unknown | CAS | USA, California, Santa Cruz Co., 9 miles north east of Soquel, [37.087, -121.851], 4.VII.1956, S. M. Fidel |
| *Eulonchus tristis* | 011688 | unknown | CAS | USA, California, Santa Cruz Co., 9 miles north east of Soquel, [37.087, -121.851], 4.VII.1956, S. M. Fidel |
| *Eulonchus tristis* | 011689 | unknown | CAS | USA, California, Santa Cruz Co., 9 miles north east of Soquel, [37.087, -121.851], 4.VII.1956, S. M. Fidel |
| *Eulonchus tristis* | 011690 | unknown | CAS | USA, California, Santa Cruz Co., 9 miles north east of Soquel, [37.087, -121.851], 4.VII.1956, S. M. Fidel |
| *Eulonchus tristis* | 011691 | unknown | CAS | USA, California, Santa Cruz Co., 9 miles north east of Soquel, [37.087, -121.851], 4.VII.1956, S. M. Fidel |
| *Eulonchus tristis* | 011692 | unknown | CAS | USA, California, Santa Cruz Co., Soquel, [36.988, -121.957], 4.VII.1954, M. T. James |
| *Eulonchus tristis* | 011693 | unknown | CAS | USA, California, Santa Cruz Co., 9 miles north east of Soquel, [37.087, -121.851], 16.VII.1956, S. M. Fidel |
| *Eulonchus tristis* | 011694 | unknown | CAS | USA, California, Santa Cruz Co., 9 miles north east of Soquel, [37.087, -121.851], 16.VII.1956, S. M. Fidel |
| *Eulonchus tristis* | 011695 | unknown | CAS | USA, California, Santa Cruz Co., 9 miles north east of Soquel, [37.087, -121.851], 16.VII.1956, S. M. Fidel |
| *Eulonchus tristis* | 011696 | unknown | CAS | USA, California, Santa Barbara County, Santa Ynez Mountains, [34.549, -120.029] |
| *Eulonchus tristis* | 011697 | unknown | CAS | USA, California, Santa Barbara County, Santa Ynez Mountains, [34.549, -120.029] |
| *Eulonchus tristis* | 011698 | unknown | CAS | USA, California, Santa Barbara County, Santa Ynez Mountains, [34.549, -120.029] |
| *Eulonchus tristis* | 011701 | unknown | CAS | USA, California, Santa Cruz County, 8.1 km east Glenwood, [37.109, -121.896], 2.VII.1956, D. J. Burdick |
| *Eulonchus tristis* | 011703 | unknown | CAS | USA, California, Santa Cruz County, Big Basin State Park, [37.172, -122.223], 21.VII.1953, W. J. Gertsch |
| *Eulonchus tristis* | 011704 | unknown | CAS | USA, California, Santa Cruz, Big Basin, [37.172, -122.223], 29.VI.1946, P.H. Arnaud |
| *Eulonchus tristis* | 011705 | unknown | CAS | USA, California, Santa Cruz County, Santa Cruz Mountains, [37.11, -121.844], C. V. Riley |
| *Eulonchus tristis* | 011706 | unknown | CAS | USA, California, Sonoma County, Sobre Vista, [38.333, -122.511], 27.IV.1910, J. A. Kusche |
| *Eulonchus tristis* | 011707 | unknown | CAS | USA, California, Santa Cruz County, Santa Cruz Mountains, [37.091, -121.844], .VI.1922, F. X. Williams |
| *Eulonchus tristis* | 011708 | unknown | CAS | USA, California, Marin County, Mill Valley, =, [37.906, -122.545], 15.VI.1959, H. B. Leech |
| *Eulonchus tristis* | 011709 | unknown | CAS | USA, California, Marin County, Mill Valley, =, [37.906, -122.545], 15.VI.1959, H. B. Leech |
| *Eulonchus tristis* | 011710 | unknown | CAS | USA, California, Marin County, Mill Valley, =, [37.906, -122.545], 15.VI.1959, H. B. Leech |
| *Eulonchus tristis* | 011711 | unknown | CAS | USA, California, Marin County, Mill Valley, =, [37.906, -122.545], 15.VI.1959, H. B. Leech |
| *Eulonchus tristis* | 011712 | unknown | CAS | USA, California, Marin County, Mill Valley, =, [37.906, -122.545], 15.VI.1959, H. B. Leech |
| *Eulonchus tristis* | 011713 | unknown | CAS | USA, California, Marin County, Mill Valley, =, [37.906, -122.545], 15.VI.1959, H. B. Leech |
| *Eulonchus tristis* | 011714 | unknown | CAS | USA, California, Marin County, Mill Valley, =, [37.906, -122.545], 15.VI.1959, H. B. Leech |
| *Eulonchus tristis* | 011715 | unknown | CAS | USA, California, Santa Cruz Co., Woodwardia, [37.056, -122.06], 31.V.1931, C. D. Duncan |
| *Eulonchus tristis* | 011718 | unknown | CAS | USA, Oregon, Clackamas County, 9.7 km SE of Oak Grove Ranger Station, Shellrock Campground, [45.029, -121.932], 671 m, 19.VI.1955, M. T. James |
| *Eulonchus tristis* | 011719 | unknown | CAS | USA, Oregon, Clackamas County, 9.7 km SE of Oak Grove Ranger Station, Shellrock Campground, [45.029, -121.932], 671 m, 19.VI.1955, M. T. James |
| *Eulonchus tristis* | 011720 | unknown | CAS | USA, Oregon, Clackamas County, 9.7 km SE of Oak Grove Ranger Station, Shellrock Campground, [45.029, -121.932], 671 m, 19.VI.1955, M. T. James |
| *Eulonchus tristis* | 011721 | unknown | CAS | USA, Oregon, Clackamas County, 9.7 km SE of Oak Grove Ranger Station, Shellrock Campground, [45.029, -121.932], 671 m, 19.VI.1955, M. T. James |
| *Eulonchus tristis* | 011723 | unknown | CAS | USA, Oregon, Clackamas County, 9.7 km SE of Oak Grove Ranger Station, Shellrock Campground, [45.029, -121.932], 671 m, 19.VI.1955, M. T. James |
| *Eulonchus tristis* | 011725 | unknown | CAS | USA, Oregon, Clackamas County, 9.7 km SE of Oak Grove Ranger Station, Shellrock Campground, [45.029, -121.932], 671 m, 19.VI.1955, M. T. James |
[truncated: 213,145 more chars]
